# Causal atlas between inflammatory bowel disease and mental disorders: a bi-directional 2-sample Mendelian randomization study

**DOI:** 10.3389/fimmu.2023.1267834

**Published:** 2023-10-13

**Authors:** Xiaorong Yang, Lejin Yang, Tongchao Zhang, Hong Zhang, Hui Chen, Xiuli Zuo

**Affiliations:** ^1^ Department of Gastroenterology, Qilu Hospital of Shandong University, Jinan, China; ^2^ Clinical Epidemiology Unit, Qilu Hospital of Shandong University, Jinan, China; ^3^ Shandong Provincial Clinical Research Center for Digestive Disease, Qilu Hospital of Shandong University, Jinan, China; ^4^ Laboratory of Translational Gastroenterology, Qilu Hospital of Shandong University, Jinan, China; ^5^ Department of Psychology, Qilu Hospital of Shandong University, Jinan, China

**Keywords:** mental disorders, Crohn’s disease, ulcerative colitis, inflammatory bowel disease, Mendelian randomization (MR)

## Abstract

**Background:**

The brain-gut axis link has attracted increasing attention, with observational studies suggesting that the relationship between common mental disorders and inflammatory bowel disease (IBD) may run in both directions. However, so far, it is not clear whether there is causality and in which direction.

**Methods:**

We conducted a bidirectional 2-sample Mendelian randomization study to investigate the relationship between IBD, including Crohn’s disease (CD) and ulcerative colitis (UC), and mental disorders, using summary-level GWAS data. The main analysis was the inverse variance weighted method. IBD (including CD and UC), and nine mental disorders were used as both exposures and outcomes.

**Results:**

We found that UC could significantly lead to obsessive-compulsive disorder, attention deficit hyperactivity disorder, and autism spectrum disorder, with odds ratio (OR) of 1.245 (95% confidence intervals [CI]: 1.069-1.450; *P*=0.008), 1.050 (95%CI: 1.023-1.077; *P*=2.42×10^-4^), and 1.041 (95%CI: 1.015-1.068; *P*=0.002) respectively. In addition, we found that bipolar disorder and schizophrenia could increase the odds of IBD, with OR values of 1.138 (95%CI: 1.084-1.194; *P*=1.9×10^-7^), and 1.115 (95%CI: 1.071-1.161; *P*=1.12×10^-7^), respectively. Our results also indicate that obsessive-compulsive disorder could lead to IBD, especially for UC, with OR values of 1.091 (95%CI: 1.024-1.162; *P*=0.009), and 1.124 (95%CI: 1.041-1.214; *P*=0.004), respectively.

**Conclusions:**

Our findings indicate that the brain-gut axis involves the association between IBD, especially UC, and some mental disorders, which guides the targeted prevention, management, and mechanism exploration of these diseases.

## 1 Introduction

Inflammatory bowel disease (IBD), containing Crohn’s disease (CD) and ulcerative colitis (UC), is a chronic systemic immune-mediated inflammatory disease that mainly affects the digestive system (1). The predominant clinical symptoms of IBD are abdominal pain and diarrhea. For UC patients, anal bleeding is also a common symptom (2). In UC, inflammation is limited to colon mucosa and rectal mucosa, but in CD, inflammation involves any transmural segment of the entire digestive tract from mouth to anus (3). IBD often occurs in early adulthood and is associated with subsequent colorectal cancer, stroke, and other diseases (3–6). IBD is widespread in Europe, North America, Oceania, and other regions with higher socio-economic status (5, 7). In Europe, one in 198 people is diagnosed with UC, and one in 310 people is diagnosed with CD. In the past three decades, the incidence rate of newly industrialized countries and territories in Asia, South America, and Africa has been rising (8). The huge disease burden makes the exploration of prevention and treatment measures for IBD a top priority.

Mental disorders are a kind of chronic diseases, mainly including attention deficit hyperactivity disorder (ADHD), anxiety disorder, autism spectrum disorder (ASD), bipolar disorder (BD), anorexia nervosa (AN), major depressive disorder (MDD), obsessive-compulsive disorder (OCD), post-traumatic stress disorder (PTSD), etc (9). The burden of diseases caused by mental disorders is heavy. In the past three decades, the number of disability-adjusted life years (DALYs) caused by mental disorders has climbed from 80.8 million to 125.3 million (9). During COVID-19, the disease burden of mental disorders may further increase (10). Some previous articles have suggested that IBD and mental disorders keep intimate bi-directional links with each other (11). Kuan-Yi Sung et al. explored the relationship between schizophrenia and IBD using the Taiwan National Health Insurance Research Database and reported that there was a significant correlation between schizophrenia and the subsequent development of IBD (12). Charles N Bernstein et al. used population-based administrative health data from Manitoba, Canada finding that the incidence rate and prevalence of mental disorders ascended in the IBD population (13). Daniele Ferrarese et al. observed a high prevalence rate of PTSD and dissociative symptoms among IBD patients (14). Maunoo Lee et al. reported that children with autism spectrum disorders have a higher prevalence rate of IBD using the data from the Military Health System database (15). Nosheen Umar et al. found that intentional self-harm, anxiety, depression, and insomnia were more likely to occur in IBD patients using the retrospective UK primary care medical database (16). However, the current studies are mostly limited to the observational correlation, and the cause and effect between IBD and mental disorders are still unknown.

The Mendelian randomization (MR) method is a common approach in causality research, which is similar to randomized controlled trials and is a natural randomized trial (17–21). The application of the MR method has three basic prerequisites: (a) instrumental variables, namely gene polymorphism, are closely related to exposure factors; (b) instrumental variables should be independent of any exposure confounders; (c) the association between instrumental variables and research outcomes only exists through exposure factors. Along with the rapid progress of sequencing technology, abundant large-scale genome-wide association studies (GWAS) data on mental disorders and IBD have been publicly reported. This provides an opportunity to use the MR method to explore and assess the bi-directional causal relationship between mental disorders and IBD based on summary-level data. In the current study, we applied a two-sample MR (2SMR) approach to evaluate the bi-directional causal relationship between nine mental disorders and IBD, (including UC and CD subtypes), in order to provide evidence for the prevention and control of the corresponding diseases.

## 2 Methods

### 2.1 Ethics considerations

This current study is based on the summary results from the published studies. Ethical approval for each of the included studies can be found in the original publication. Because our study uses summary-level data and ethical approval and informed consent were obtained in all original studies, ethical approval is exempted in our hospital.

### 2.2 Study design

**Figure 1:**
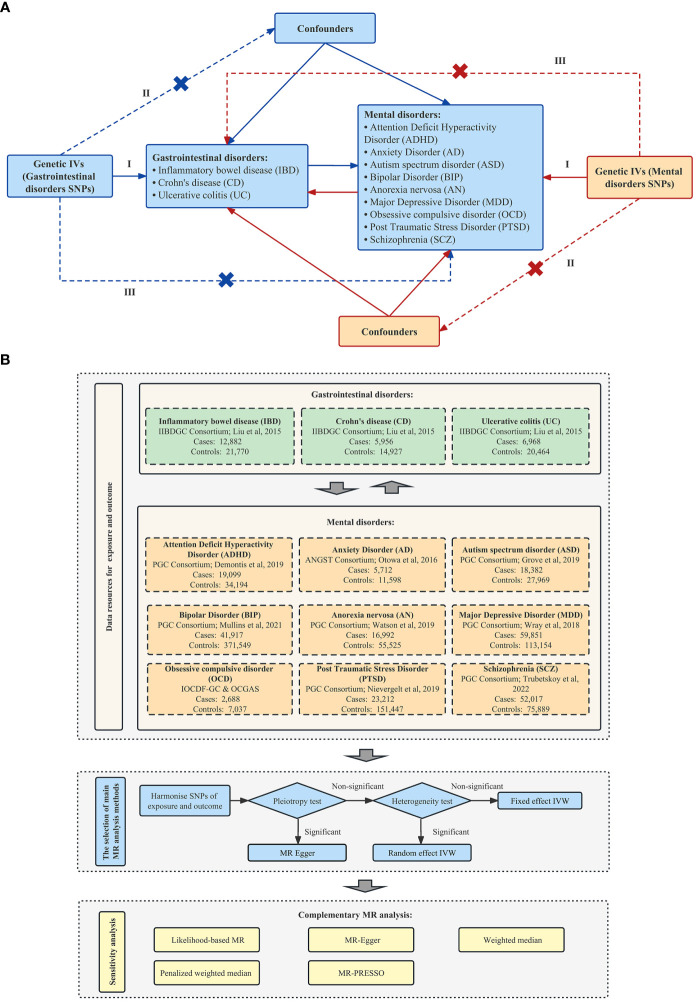
Schematic overview of this 2SMR study design. **(A)** Principles of this 2SMR study. Three principal assumptions in MR design, as follows: (I) The relevance assumption—the selected instrument is a prediction of the exposure; (II) The independence assumption—the instrument is independent of any confounders of the exposure and outcome; and (III) The exclusion-restriction assumption—the instrument is only associated with the outcome through the exposure. **(B)**The research design and framework of our study [International Obsessive Compulsive Disorder Foundation Genetics Collaborative (IOCDF-GC) and OCD Collaborative Genetics Association Studies (OCGAS) (22); Demontis et al. (23); Grove et al. (24); Liu et al. (25); Mullins et al. (26); Nievergelt et al. (27); Otowa et al. (28); Trubetskoy et al. (29); Watson et al. (30); Wray et al. (31)].

To explore and assess the association between IBD and nine mental disorders, we performed a 2SMR analysis based on summary-level GWAS, which followed the Strengthening the Reporting of Observational Studies in Epidemiology Using Mendelian Randomization (STROBE-MR) checklist (**Supplementary checklist**). The core principle of the MR method and the three prerequisites of instrumental variables in our study, namely, the relevance assumption, independence assumption, and exclusion-restriction assumption, were summarized in **Figure 1A**. The flow chart of our 2SMR analysis in this study was summarized in **Figure 1B**.

### 2.3 Data sources

GWAS data of IBD, containing CD and UC, came from European GWAS, which was structured based on up to 15 original studies on European populations. 12882 IBD cases (21770 healthy controls), 5956 CD cases (14927 healthy controls), and 6968 UC cases (20464 healthy controls) were available in this data set, with corresponding GWAS IDs of ieu-a-31, ieu-a-30, and ieu-a-32, respectively (25). GWAS data for mental disorders is from ANGST Consortium, IOCDF-GC & OCGAS, and Psychiatric Genomics Consortium (PGC, https://pgc.unc.edu). According to DSM-5, we included most mental disorders, including ADHD, anxiety disorder, ASD, BD, AN, MDD, OCD, PTSD, and schizophrenia (9). 19099 ADHD cases (34194 controls), 18382 ASD cases (27969 controls), 41917 BD cases (371549 controls), 16992 AN cases (55525 controls), 59851 MDD cases (113154 controls), 23212 PTSD cases (151447 controls), and 52017 schizophrenia cases (75889 controls) were available in PGC data set (23, 24, 26, 27, 29–31), 5712 anxiety disorder cases (11598 controls) were available in ANGST Consortium data set (28) and 2688 OCD cases (7037 controls) were available in IOCDF-GC & OCGAS data set (22). The summary information of these GWAS studies could be found in **Table 1**.

**Table 1:** The summary of instrumental variables for gastrointestinal disorders (*P* < 5 × 10^−8^) and mental disorders (*P* < 5 × 10^−5^).

Trait (reference)	Ancestry	Sample size	Cases	Controls	Number of SNPs	*P*-value	No. SNPs in MR	LD	F-statistic range	R2, %	PMID
Gastrointestinal disorders											
Inflammatory bowel disease (25)	Only Europeans	34652	12882	21770	12716084	5E-08	79	0.001	29.81-290.21	13.66	26192919
Crohn’s disease (25)	Only Europeans	20883	5956	14927	12276506	5E-08	63	0.001	29.97-272.94	17.88	26192919
Ulcerative colitis (25)	Only Europeans	27432	6968	20464	12255197	5E-08	48	0.001	29.71-226.24	11.36	26192919
Mental disorders											
ADHD (23)	Only Europeans	53293	19099	34194	8094094	0.00005	184	0.001	16.44-50.71	7.25	30478444
Anxiety disorder (28)	Only Europeans	17310	5712	11598	6330995	0.00005	86	0.001	16.45-31.94	16.74	26754954
ASD (24)	Only Europeans	46351	18382	27969	9112386	0.00005	147	0.001	16.44-35.77	6.02	30804558
BD (26)	Only Europeans	413466	41917	371549	7608183	0.00005	383	0.001	16.47-79.65	2.16	34002096
AN (30)	Only Europeans	72517	16992	55525	8219102	0.00005	191	0.001	16.44-60.70	5.16	31308545
MDD (31)	Only Europeans	173005	59851	113154	13554550	0.00005	149	0.001	16.37-44.50	1.71	29700475
OCD (22)	>85% Europeans	9725	2688	7037	8409516	0.00005	103	0.001	16.47-25.57	19.75	28761083
PTSD (27)	≥90% Europeans	174659	23212	151447	9766174	0.00005	137	0.001	16.48-35.03	1.45	31594949
Schizophrenia (29)	Only Europeans	127906	52017	75889	7659767	0.00005	536	0.001	16.38-175.28	11.77	35396580

SNPs, single nucleotide polymorphisms; MR, Mendelian randomization; LD, linkage disequilibrium; PMID, PubMed Unique Identifier; ADHD, attention deficit hyperactivity disorder; ASD, autism spectrum disorder; BD, bipolar disorder; AN, anorexia nervosa; MDD, major depressive disorder; OCD, obsessive-compulsive disorder; PTSD, post-traumatic stress disorder.

### 2.4 Instruments selection

The genetic instrumental variables of gastrointestinal disorders, namely the determined single nucleotide polymorphisms (SNPs) of IBD, CD, and UC were identified in the above-mentioned European GWAS with the following condition of *P* < 5 × 10^−8^, and linkage disequilibrium (LD): r^2 = ^0.001 and clump distance = 10,000 kb. 79 independent SNPs related to IBD disorders were finally gained on 34652 individuals of European ancestry. In addition, 63 independent SNPs related to CD were selected from 20833 individuals of European ancestry, and 48 independent SNPs related to UC were identified from 27432 European participants (25).

The genetic instrumental variables of mental disorders, namely the determined SNPs of ADHD, anxiety disorder, ASD, BD, AN, MDD, OCD, PTSD, and schizophrenia were identified in PGC, IOCDF-GC & OCGAS and ANGST Consortium with the following conditions of *P* < 5 × 10^−5^, and LD: r^2 = ^0.001 and clump distance = 10,000 kb. 184 independent SNPs for ADHD (n = 53293), 147 SNPs for ASD (n = 46351), 383 SNPs for BD (n = 413466), 191 SNPs for AN (n = 72517), 149 SNPs for MDD (n = 173005), 137 SNPs for PTSD (n = 174659), and 536 SNPs for schizophrenia (n = 127906) were obtained from European ancestry in PGC data set (23, 24, 26, 27, 29–31). A total of 86 independent SNPs related to anxiety disorder were extracted from 17310 individuals of European ancestry in the ANGST Consortium (28), and 103 independent SNPs for OCD were identified from 9725 individuals (>85% European) in IOCDF-GC & OCGAS (22). Depending on the PhennoScanner database (http://www.phenoscanner.medschl.cam.ac.uk/, accessed on 6 March 2023), the SNPs that do not achieve the whole genome significance level with confounding factors are retained in the genetic instrument (32). The variance (R^2^) in the MR study can represent the proportion of total exposure difference explained by the potential instrumental variables. The R^2^ of instrumental variables for IBD and mental disorders can be obtained from the original studies or calculated by the formula listed below according to the summary statistical data of each exposure: R^2^ = (2 * EAF * (1 − EAF) * Beta^2^)/[(2 * EAF * (1 − EAF) * Beta^2^) + (2 * EAF * (1 − EAF) * N * SE^2^)]. EAF denotes the effect of allele frequency, beta denotes the estimated genetic effect of SNP on each exposure factor, N denotes the sample size of GWAS and SE denotes the standard error of the estimated effect. We further calculated the F-statistic for each SNP by the formula listed below: Beta^2^/SE^2^. We retained the SNPs with F-statistics of more than 10 as the final genetic variables to avoid the risk of selecting weak instrumental variables. The priori statistical power was calculated using the mRnd power calculation online tool (33).

### 2.5 Statistical analyses

The existence of potential pleiotropy was evaluated by using the MR-Egger intercept test. If significant horizontal pleiotropy was observed, the MR-Egger regression was finally adopted, otherwise, the inverse-variance weighted (IVW) method was adopted. The heterogeneity was quantified by I^2^ statistic and the *P*-value of Cochran′s Q-statistics test. If significant heterogeneity was observed, the random-effect IVW model was adopted, otherwise, the fixed-effect IVW model was adopted. Furthermore, the sensitivity analyses were executed to verify the robustness of our research results. The application of MR-Egger regression, likelihood-based MR, weighted median method, penalized weighted median method, and MR Pleiotropy RESidual Sum and Outlier (MR-PRESSO) supports expounding and verifying causal relationships (34). In order to initially control the risk of potential multiple tests in our study, the *P* < 0.01 was a significant correlation, and *P* values between 0.01 and 0.05 were considered suggestive correlation. We used R software (Version 4.1.0; https://www.r-project.org/) to perform all statistical analyses and graph presentations. The R-based “TwoSampleMR” package was applied to conduct our MR analysis.

## 3 Results

### 3.1 Assessment of genetic instruments

**Table 1** shows the GWAS summary information on the included gastrointestinal disorders and mental disorders. The variance explicated by the selected genetic instruments ranged from 11.36% to 17.88% for gastrointestinal disorders (all F statistics>10) and from 1.45% to 19.75% for mental disorders (all F statistics> 10). When priori the odds ratio (OR) of correlation strength was 1.2 (0.8) or stronger, some only at 1.1 (0.9), there was sufficient statistical power to detect significant differences (**Supplementary Tables S1, S2**). The original data information for the effective evaluation of the selected SNP associated with gastrointestinal disorders and mental disorders is given in the **supplementary materials**.

### 3.2 Causal atlas of gastrointestinal disorders on mental disorders

**Figure 2:**
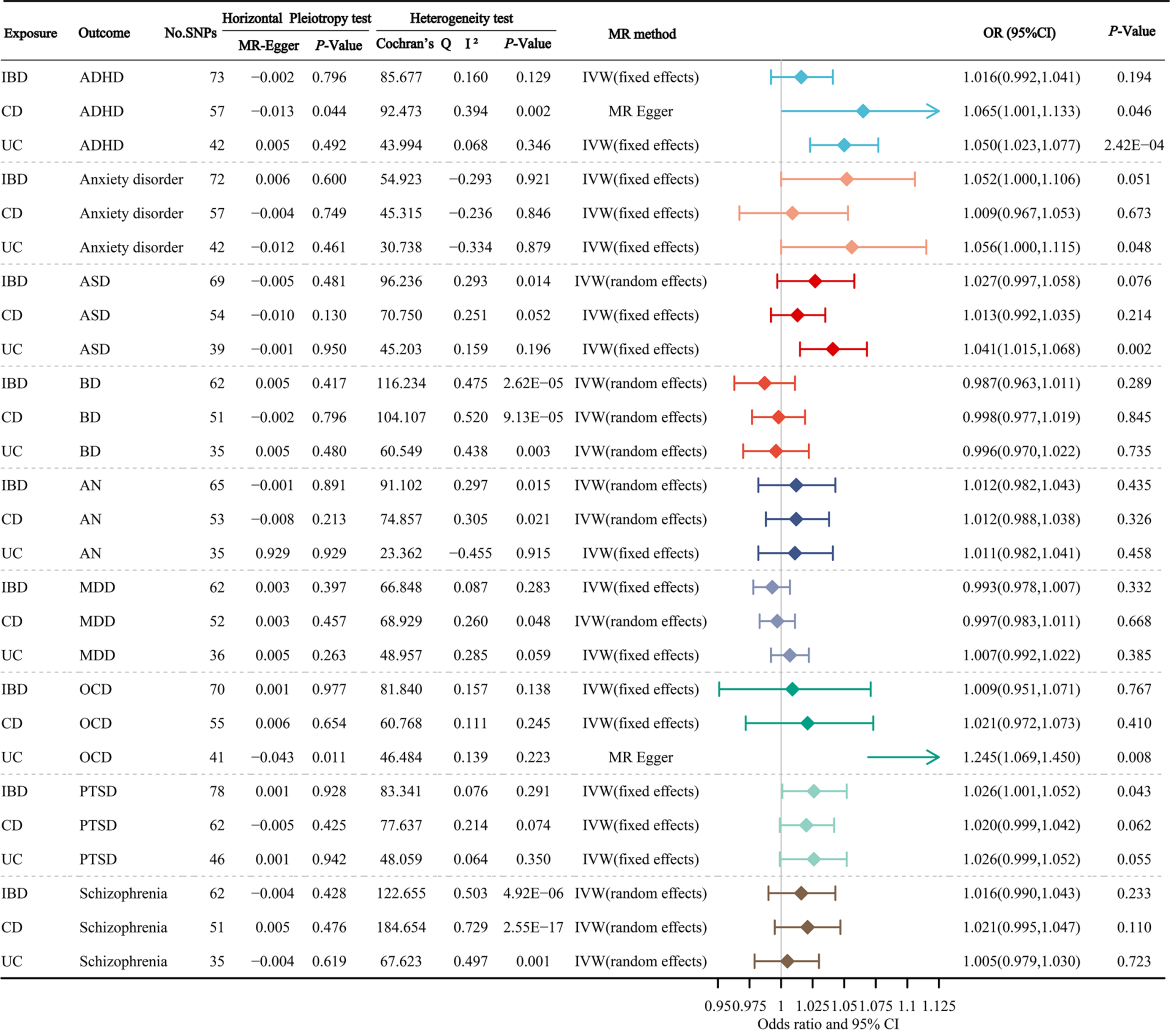
The primary results of the causal atlas of gastrointestinal disorders on mental disorders. ADHD, attention deficit hyperactivity disorder; ASD, autism spectrum disorder; BD, bipolar disorder; AN, anorexia nervosa; MDD, major depressive disorder; OCD, obsessive-compulsive disorder; PTSD, post-traumatic stress disorder; IBD, inflammatory bowel disease; CD, Crohn’s disease; UC, ulcerative colitis.

**Figure 2** manifests the primary results of the causal relationship of gastrointestinal disorders with mental disorders. The MR Egger intercept test did not observe a significant pleiotropy, except for the causal relationship of CD on ADHD, and the causal relationship of UC on OCD. In the main results, we found a significant correlation between UC and odds of ADHD, ASD and OCD, with corresponding OR values of 1.050 (95% CI: 1.023-1.077; *P*=2.42×10^-4^), 1.041 (95% CI: 1.015-1.068; *P*=0.002), 1.245 (95% CI: 1.069-1.450; *P*=0.008), respectively. In addition, we found a suggestive association that genetically determined CD could increase the odds of ADHD, UC could increase the odds of anxiety disorder, and IBD could increase the odds of PTSD, with corresponding OR values of 1.065 (95% CI: 1.001-1.133; *P*=0.046), 1.056 (95% CI: 1.000-1.115; *P*=0.048) and 1.026 (95% CI: 1.001-1.052; *P*=0.043), respectively.

### 3.3 Causal atlas of mental disorders on gastrointestinal disorders

**Figure 3:**
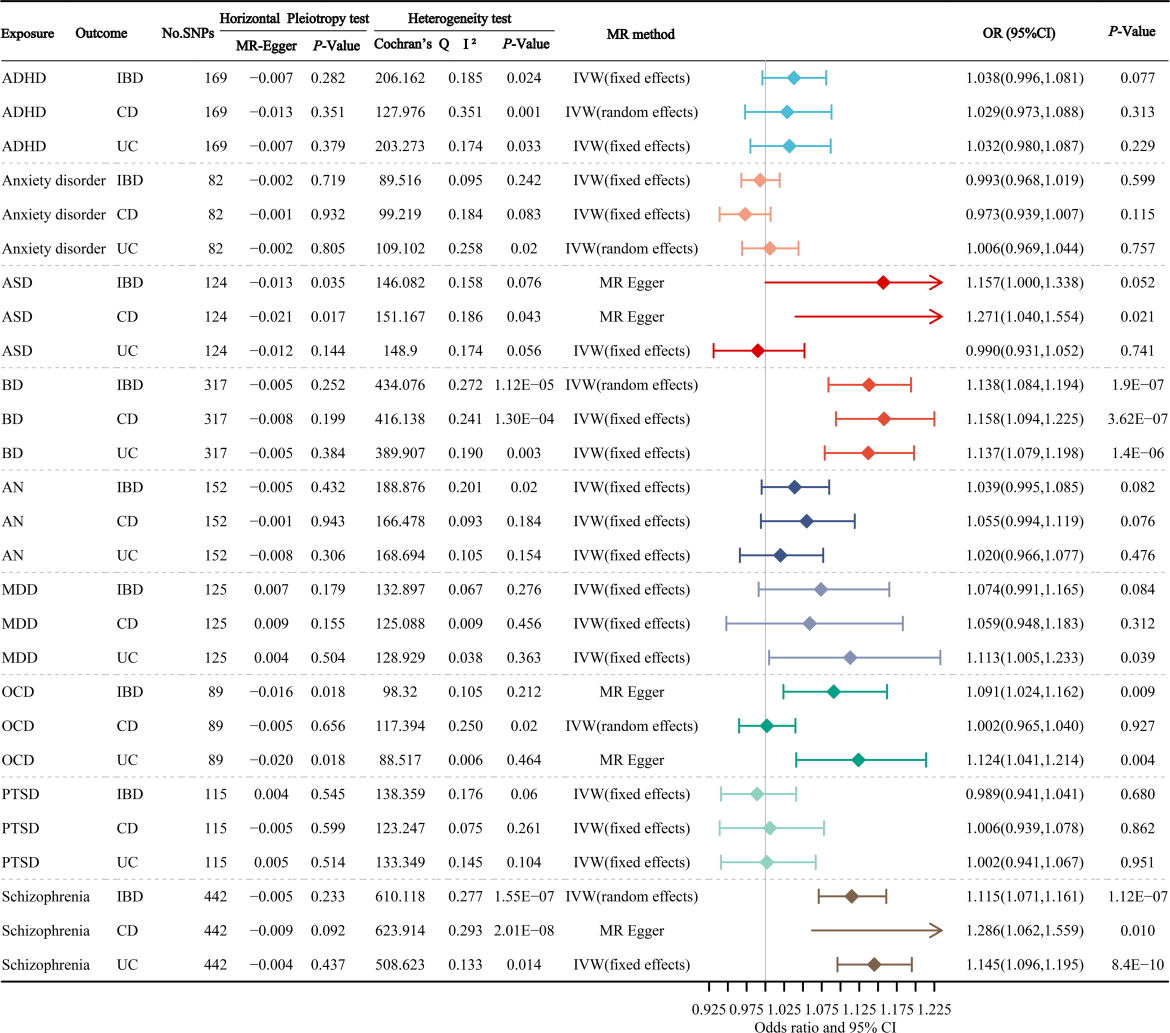
The primary results of the MR estimates for the causal atlas of mental disorders on gastrointestinal disorders. ADHD, attention deficit hyperactivity disorder; ASD, autism spectrum disorder; BD, bipolar disorder; AN, anorexia nervosa; MDD, major depressive disorder; OCD, obsessive-compulsive disorder; PTSD, post-traumatic stress disorder; IBD, inflammatory bowel disease; CD, Crohn’s disease; UC, ulcerative colitis.

**Figure 3** manifests the main results of the MR estimation for the causal atlas of mental disorders on gastrointestinal disorders. The MR Egger intercept test identified a notable horizontal pleiotropy between ASD and IBD, ASD and CD, OCD and IBD, OCD and UC, and Schizophrenia and CD. We found that BD was significantly correlated with IBD, CD and UC, with OR values of 1.138 (95% CI: 1.084-1.194; *P*=1.9×10^-7^), 1.158 (95% CI: 1.094-1.225; *P*=3.62×10^-7^) and 1.137 (95% CI: 1.079-1.198; *P*=1.4×10^-6^), respectively. OCD was significantly correlated with IBD and UC, with OR values of 1.091 (95% CI: 1.024-1.162; *P*=0.009), and 1.124 (95% CI: 1.041-1.214; *P*=0.004), respectively. There was also a significant relevance between schizophrenia and IBD, CD and UC, with OR values of 1.115 (95% CI: 1.071-1.161; *P*=1.12×10^-7^), 1.098 (95% CI: 1.039-1.161; *P*=0.010) and 1.145 (95% CI: 1.096-1.195; *P*=8.4×10^-10^), respectively. In addition, we found suggestive associations between ASD and CD, and MDD and UC, with OR values of 1.271 (95% CI: 1.040-1.554; *P*=0.021) and 1.113 (95% CI: 1.005-1.233; *P*=0.039), respectively.

### 3.4 Sensitivity analysis

We further developed a sensitivity analysis by selecting more stringent genetic instrumental variables for mental disorders (*P* < 5 × 10^−8^, LD: r2 = 0.001 and clump distance = 10,000 kb, **Supplementary Table S3**). **Supplementary Table S4** pools the summary results via a variety of complementary MR methods of the causal atlas of gastrointestinal disorders on mental disorders. Complementary MR analyses further confirmed the suggestive association between UC and ADHD, UC and ASD, and UC and OCD. **Supplementary Table S5** gathers the summary results via a variety of complementary MR methods of the causal atlas of mental disorders on gastrointestinal disorders. Complementary MR analysis also confirmed the suggestive association between BD and gastrointestinal disorders, schizophrenia and gastrointestinal disorders. Our results did not alter fundamentally after leave-one-out analyses.

## 4 Discussion

In the current study, we conducted a comprehensive 2SMR study for the first time to explore the potential causal atlas between IBD and mental disorders. Genetic variation is used as an instrumental variable for mental disorders and IBD. Our research results show that UC and ADHD, UC and ASD, UC and OCD, BD and IBD, OCD and UC, schizophrenia and IBD are significantly related, and there may be potential causal associations. In addition, we also discover some suggestive associations suggesting that CD can increase the odds of ADHD, UC is related to the increased anxiety risk, IBD can promote the occurrence of PTSD, ASD is associated with increased CD risk, and MDD is associated with increased UC risk. The robust causal atlas between IBD and mental disorders was supported by multi-aspect sensitivity analyses.

Mu-Hong Chen et al. used the database from the Taiwan National Health Insurance Research System finding that the probability of UC in ADHD patients is 2.31 times greater than that in non-patients (35). Tor-Arne Hegwik et al. used cross-sectional study data based on 2.5 million people to plumb the relationship between ADHD and immune system diseases and found that ADHD was related to UC and CD, with OR values of 1.28 and 1.44 for females, while in males, ADHD was negatively related to CD (36). However, these studies only provided evidence of the association between ADHD and IBD and did not analyze its causal relationship. In our study, we found a potential causal relationship between UC and the occurrence of ADHD, and the incidence risk of ADHD in UC patients was 1.050 times greater than in non-UC patients. In terms of ASD and OCD, we found that the risk of ASD and OCD in UC patients was 1.041 and 1.245 times higher than that in non-UC patients, respectively. Jong Yeob Kim et al. found that there was a correlation between ASD and the later development of IBD. The OR values between ASD and IBD, UC, CD of 1.66, 1.91, and 1.47, respectively, further support the results of Kim’s study (37). In another study, the prevalence of OCD in IBD patients was significantly higher than that in the general healthy population, which was 9.4% and 2% respectively (38). Previous studies have shown that parents with IBD, especially mothers with IBD, are associated with subsequent ASD in children (39). This suggests that the two diseases may share a conjoint genetic basis and common risk factors (40). In addition, the brain-gut axis is also an attractive potential factor, involving complex interactions between neuroendocrine regulatory pathways, the central, peripheral, and autonomic nervous systems, as well as the gastrointestinal tract system (41). Previous studies have pointed out that intestinal inflammation may affect the activity and behavior of the brain by affecting key brain neurotransmitters, such as glutamate, TNF-α, etc. (42). In addition, along with changes in the intestinal microbial environment, the imbalances in the vagus nerve, immune-inflammatory process and bacterial metabolites and products are related to the changes in blood-brain barrier function and neuroinflammation, which promotes the occurrence of mental disorders (43, 44).

Several previous researches have suggested that there is a close correlation between IBD and BD. Li-Ting Kao et al. used the database from the National Health Insurance Research system to report a significant cross-sectional association between IBD and BD with an OR of 2.10 (45). Charles N Bernstein et al. used population-based administrative health data from Manitoba, Canada data finding that the incidence rate of BD in IBD patients is 1.84 times higher. Females, with higher socio-economic status, 18-24 years old may be a risk factor (13). However, the authentic causal relationship between IBD and BD is still unknown. In this study, we revealed the potential causal relationship between BD and IBD, CD, and UC from the perspective of genetic linkage. The risk of IBD, CD, and UC in patients with BD was 1.138, 1.158, and 1.137 times higher than that in patients without BD, respectively. Zhe Wang et al. used the 2SMR method to inquire into the causal relationship of IBD with BD. The results stated that the predicted hereditary BD has a significant and positive correlation with the risk of IBD, CD, and UC, with ORs of 1.16, 1.20, and 1.17 respectively, which were consistent with our results (46). We also found a significant correlation between schizophrenia and IBD, CD, and UC, with OR values of 1.115, 1.098, and 1.145, respectively. Previous studies have reported that the incidence of schizophrenia in IBD patients is 1.64 times higher than that in the control population, which indicates the correlation between the two and supports this study to some extent (13). Mental disorders may promote the occurrence of IBD through the brain-gut axis, involving the vegetative nervous system, central nervous system responses, stress pathways response (hypothalamus-pituitary-adrenal axis), (gastrointestinal) corticotropin-releasing factor pathways, etc (47, 48). Emotional changes may also increase intestinal permeability so that intestinal bacteria can transfer to peripheral lymphatic organs through the intestinal tract, trigger an innate immune response, and promote the development of IBD (42). In addition, the abundance of gut microbiota is also a potential cause. A study on the gut microbiome of patients with depression has found that depression is associated with a decrease in gut microbiome richness and diversity, suggesting that this may be a potential influencing factor (49, 50).

Previous documents have implied that there is a two-way relationship between anxiety and depression and IBD (51). However, in this study, we only found a suggestive association that UC slightly increases the risk of anxiety, while MDD is significantly associated with an increased risk of UC. In addition, we found suggestive association that IBD is associated with an incremented incidence of PTSD, and ASD is associated with increased CD risk. Considering that the criteria we used when screening the tool variables of mental disorders are *P* < 5 × 10^−5^, LD: r^2 = ^0.001, and clump distance = 10,000 kb, this part of the results needs to be further verified with GWAS data of a larger sample.

Our study has some specific benefits. To begin with, our study applied the instrumental variable-based 2SMR design to reduce the impacts of uncontrolled confounding factors and small sample selection bias, and to replenish the genetically theoretical cornerstone of the bi-directional causal atlas between various mental disorders and IBD. Furthermore, our study comprehensively analyzed the causal relationship between nine mental disorders and IBD with its subtypes from two directions in detail, which is the most integrated study based on the 2SMR method at present. Finally, our research carried out multifaceted sensitivity analyses, and the outcomes gained by other approaches were accordant with the main IVW methods, which further demonstrated the robustness of our findings.

The main limitations of our study have been stated below. First, the statistical analysis of this study is dependent on the published summary data rather than personal data information. Consequently, we cannot examine the possible nonlinear causal relationship between mental disorders and IBD. In addition, we cannot evaluate the causal atlas between mental disorders and IBD by gender. Second, the congenital shortage of the MR analysis method is that the potential pleiotropy can affect the accuracy of causal inference. Fortunately, no SNP remarkably related to confounding was identified via the PhennoScanner database, and the MR Egger intercept test in our study presented that there was almost no notable pleiotropic effect, which proclaims that the pleiotropic effect was unlikely to occur for most causal estimations. For a small amount of significant horizontal pleiotropy, the MR-Egger regression was used to further adjust the causal association. Third, albeit we have only observed significant associations in UC and ADHD, UC and ASD, UC and OCD, BD and IBD, schizophrenia and IBD, we cannot utterly eliminate the possibility that the influence of mental system diseases and IBD is too small to be determined. These weak relationships may be discovered through the subsequent use of large-scale data. Fourth, because there are fewer reliable instrument variables available for the mental disorders of interest, in order to include more potentially available tool variables, we use *P* < 5 × 10^−5^ as one of the screening conditions for instrument variables, which makes the ability to detect the suggestive effect of mental disorders on IBD may be relatively low in this study. This may also lead to some relationships being difficult to discover in this study, therefore, more large-scale GWAS samples on exposure need to be used to verify our results in the future.

In this MR study, the results show that there is a significant causal relationship between UC and ADHD, UC and ASD, UC and OCD, BD and IBD, OCD and UC, and schizophrenia and IBD. In addition, we also found some suggestive associations that CD can increase the odds of ADHD, UC is related to the increased anxiety risk, IBD can promote the occurrence of PTSD, ASD is associated with increased CD risk, and MDD is associated with increased UC risk. The findings of our study further emphasize the interaction between the brain-gut axis from a genetic perspective, and improve the understanding of the causal atlas between IBD and various mental illnesses for clinicians and primary care doctors, which will help to make an accurate comprehensive diagnosis and interdisciplinary joint treatment care for potential patients in a more timely manner. In the future, more mechanism exploration and treatment interventions on mental disorders and IBD could be conducted to verify and extend our results and bring potential clinical benefits.

## Data availability statement

The original data sources used to support our findings of this study are provided within the supplementary material (**Supplementary Tables S6-S59**). All summary statistics approaches for the two-sample Mendelian randomization are retrieved online from every genome-wide association study consortia. Further inquiries can be directed to the corresponding authors.

## Author contributions

XY: Conceptualization, Data curation, Formal Analysis, Funding acquisition, Methodology, Visualization, Writing – original draft, Writing – review & editing. LY: Methodology, Supervision, Writing – review & editing. TZ: Data curation, Methodology, Validation, Visualization, Writing – review & editing. HZ: Software, Supervision, Visualization, Writing – review & editing. HC: Software, Supervision, Visualization, Writing – review & editing. XZ: Conceptualization, Funding acquisition, Methodology, Supervision, Writing – review & editing.
