# Supplementary material for: Causal atlas between inflammatory bowel disease and mental disorders: a bi-directional 2-sample Mendelian randomization study
Source: Front Immunol. 2023 Oct 13;14:1267834. doi: 10.3389/fimmu.2023.1267834 (PMC10611497; doi:10.3389/fimmu.2023.1267834)
Supplement: Supplementary file 1 [file DataSheet_1.pdf]

Table S1. The priori statistical power in the MR analysis of the causal effects.								
**Exposure**	**Outcome**	**Sample size**	**α**	**K**	**R2, %**	**Power at different odds ratios and a=0.05**		
						**1.1 or 0.9**	**1.2 or 0.8**	**1.4 or 0.7**
**IBD**	ADHD	53293	0.05	0.3583773	0.1366	0.98	1	1
**IBD**	Anxiety disorder	17310	0.05	0.33	0.1366	0.6	0.99	1
**IBD**	ASD	46351	0.05	0.3965826	0.1366	0.96	1	1
**IBD**	BD	413466	0.05	0.1013796	0.1366	1	1	1
**IBD**	AN	72517	0.05	0.2343175	0.1366	0.98	1	1
**IBD**	MDD	173005	0.05	0.3459495	0.1366	1	1	1
**IBD**	OCD	9725	0.05	0.276401	0.1366	0.36	0.87	1
**IBD**	PTSD	174659	0.05	0.132899	0.1366	1	1	1
**IBD**	Schizophrenia	127906	0.05	0.4066815	0.1366	1	1	1
**CD**	ADHD	53293	0.05	0.3583773	0.1788	0.99	1	1
**CD**	Anxiety disorder	17310	0.05	0.33	0.1788	0.72	1	1
**CD**	ASD	46351	0.05	0.3965826	0.1788	0.99	1	1
**CD**	BD	413466	0.05	0.1013796	0.1788	1	1	1
**CD**	AN	72517	0.05	0.2343175	0.1788	1	1	1
**CD**	MDD	173005	0.05	0.3459495	0.1788	1	1	1
**CD**	OCD	9725	0.05	0.276401	0.1788	0.44	0.94	1
**CD**	PTSD	174659	0.05	0.132899	0.1788	1	1	1
**CD**	Schizophrenia	127906	0.05	0.4066815	0.1788	1	1	1
**UC**	ADHD	53293	0.05	0.3583773	0.1136	0.95	1	1
**UC**	Anxiety disorder	17310	0.05	0.33	0.1136	0.52	0.98	1
**UC**	ASD	46351	0.05	0.3965826	0.1136	0.93	1	1
**UC**	BD	413466	0.05	0.1013796	0.1136	1	1	1
**UC**	AN	72517	0.05	0.2343175	0.1136	0.96	1	1

**UC**	MDD	173005	0.05	0.3459495	0.1136	1	1	1
**UC**	OCD	9725	0.05	0.276401	0.1136	0.3	0.81	1
**UC**	PTSD	174659	0.05	0.132899	0.1136	1	1	1
**UC**	Schizophrenia	127906	0.05	0.4066815	0.1136	1	1	1

Table S2. The priori statistical power in the MR analysis of the causal effects.								
**Exposure**	**Outcome**	**Sample size**	**α**	**K**	**R2, %**	**Power at different odds ratios and a=0.05**		
						**1.1 or 0.9**	**1.2 or 0.8**	**1.4 or 0.7**
**ADHD**	IBD	34652	0.05	0.3717534	0.0725	0.65	0.99	1
**ADHD**	CD	20883	0.05	0.2852081	0.0725	0.4	0.92	1
**ADHD**	UC	27432	0.05	0.2540099	0.0725	0.47	0.96	1
**Anxiety disorder**	IBD	34652	0.05	0.3717534	0.1674	0.94	1	1
**Anxiety disorder**	CD	20883	0.05	0.2852081	0.1674	0.74	1	1
**Anxiety disorder**	UC	27432	0.05	0.2540099	0.1674	0.82	1	1
**ASD**	IBD	34652	0.05	0.3717534	0.0602	0.57	0.98	1
**ASD**	CD	20883	0.05	0.2852081	0.0602	0.34	0.86	0.99
**ASD**	UC	27432	0.05	0.2540099	0.0602	0.41	0.92	1
**BD**	IBD	34652	0.05	0.3717534	0.0216	0.25	0.7	0.95
**BD**	CD	20883	0.05	0.2852081	0.0216	0.15	0.44	0.76
**BD**	UC	27432	0.05	0.2540099	0.0216	0.18	0.53	0.85
**AN**	IBD	34652	0.05	0.3717534	0.0516	0.51	0.97	1
**AN**	CD	20883	0.05	0.2852081	0.0516	0.3	0.8	0.98
**AN**	UC	27432	0.05	0.2540099	0.0516	0.36	0.88	1
**MDD**	IBD	34652	0.05	0.3717534	0.0171	0.21	0.59	0.89
**MDD**	CD	20883	0.05	0.2852081	0.0171	0.13	0.37	0.66
**MDD**	UC	27432	0.05	0.2540099	0.0171	0.15	0.44	0.75
**OCD**	IBD	34652	0.05	0.3717534	0.1975	0.97	1	1
**OCD**	CD	20883	0.05	0.2852081	0.1975	0.81	1	1
**OCD**	UC	27432	0.05	0.2540099	0.1975	0.88	1	1
**PTSD**	IBD	34652	0.05	0.3717534	0.0145	0.18	0.53	0.84
**PTSD**	CD	20883	0.05	0.2852081	0.0145	0.12	0.32	0.59
**PTSD**	UC	27432	0.05	0.2540099	0.0145	0.14	0.38	0.68
**Schizophrenia**	IBD	34652	0.05	0.3717534	0.1177	0.85	1	1

**Schizophrenia**	CD	20883	0.05	0.2852081	0.1177	0.59	0.99	1
**Schizophrenia**	UC	27432	0.05	0.2540099	0.1177	0.68	1	1

Table S3. The summary of instrumental variables for mental disorders (P<5*10-8).											
**Trait**	**Ancestry**	**Sample size**	**Cases**	**Controls**	**Number of SNPs**	**P value**	**No. SNPs in MR**	**LD**	**F-statistic range**	**R2, %**	**PMID**
**ADHD**	Only Europeans	53293	19099	34194	8094094	0.00000005	11	0.001	30.12-50.71	0.71	30478444
**Anxiety disorder**	Only Europeans	17310	5712.3	11597.7	6330995	0.00000005	1	0.001	31.94154778	0.18	26754954
**ASD**	Only Europeans	46351	18382	27969	9112386	0.00000005	2	0.001	32.89-35.77	0.15	30804558
**BD**	Only Europeans	413466	41917	371549	7608183	0.00000005	54	0.001	29.75-79.65	0.49	34002096
**AN**	Only Europeans	72517	16992	55525	8219102	0.00000005	8	0.001	30.08-60.70	0.4	31308545
**MDD**	Only Europeans	173005	59851	113154	13554550	0.00000005	5	0.001	31.64-44.50	0.11	29700475
**OCD**	>85% Europeans	9725	2688	7037	8409516	0.00000005	0	0.001	-	-	28761083
**PTSD**	>=90% Europeans	174659	23212	151447	9766174	0.00000005	2	0.001	30.06-35.03	0.04	31594949
**Schizophrenia**	Only Europeans	127906	52017	75889	7659767	0.00000005	155	0.001	29.46-175.28	5.36	35396580

SNPs: single nucleotide polymorphisms; MR: Mendelian randomization; LD: linkage disequilibrium; PMID: PubMed Unique Identifier; ADHD: attention deficit hyperactivity disorder; ASD: autism spectrum disorder; BD: bipolar disorder; AN: anorexia nervosa; MDD: major depressive disorder; OCD: obsessive-compulsive disorder; PTSD: post-traumatic stress disorder; IBD: inflammatory bowel disease; CD: Crohn's disease; UC: ulcerative colitis.

Table S4. The complementary MR analyses results of the causal effects of gastrointestinal disorders on mental disorders.										
**Exposure**	**Outcome**	**MR methods**	**No. SNPs**	**Egger**	**P-value**	**Cochran's**	**I2**	**P value**	**OR (95%CI)**	**P value**
**IBD**	ADHD	Inverse variance weighted	73	-0.002	0.796	85.677	0.16	0.129	1.016(0.992,1.041)	0.194
		MR Egger	73						1.025(0.954,1.102)	0.502
		Maximum likelihood	73						1.016(0.992,1.041)	0.189
		Weighted median	73						1.009(0.97,1.049)	0.659
		MR PRESSO	73						——	——
	Anxiety disorder	Inverse variance weighted	72	0.006	0.6	54.923	-0.29	0.921	1.052(1.000,1.106)	0.051
		MR Egger	72						1.012(0.869,1.178)	0.88
		Maximum likelihood	72						1.052(1,1.107)	0.051
		Weighted median	72						1.076(0.994,1.165)	0.072
		MR PRESSO	72						——	——
	ASD	Inverse variance weighted	69	-0.005	0.481	96.236	0.293	0.014	1.027(0.997,1.058)	0.076
		MR Egger	69						1.057(0.971,1.151)	0.203
		Maximum likelihood	69						1.028(1.002,1.054)	0.034
		Weighted median	69						1.029(0.989,1.071)	0.152
		MR PRESSO	69						——	——
	BD	Inverse variance weighted	62	0.005	0.417	116.234	0.475	2.62E-05	0.987(0.963,1.011)	0.289
		MR Egger	62						0.961(0.898,1.029)	0.257
		Maximum likelihood	62						0.987(0.969,1.005)	0.145
		Weighted median	62						0.981(0.953,1.01)	0.198
		MR PRESSO	62						0.994(0.969,1.02)	0.633
	AN	Inverse variance weighted	65	-0.001	0.891	91.102	0.297	0.015	1.012(0.982,1.043)	0.435
		MR Egger	65						1.018(0.935,1.107)	0.686
		Maximum likelihood	65						1.012(0.987,1.039)	0.348
		Weighted median	65						0.995(0.955,1.036)	0.809
		MR PRESSO	65						1.004(0.978,1.03)	0.786
	MDD	Inverse variance weighted	62	0.003	0.397	66.848	0.087	0.283	0.993(0.978,1.007)	0.332
		MR Egger	62						0.977(0.939,1.017)	0.256

		Maximum likelihood	62						0.993(0.978,1.008)	0.333
		Weighted median	62						0.986(0.963,1.01)	0.253
		MR PRESSO	62						——	——
	OCD	Inverse variance weighted	70	0.001	0.977	81.84	0.157	0.138	1.009(0.951,1.071)	0.767
		MR Egger	70						1.006(0.839,1.207)	0.945
		Maximum likelihood	70						1.009(0.95,1.071)	0.766
		Weighted median	70						1.028(0.931,1.136)	0.581
		MR PRESSO	70						——	——
	PTSD	Inverse variance weighted	78	0.001	0.928	83.341	0.076	0.291	1.026(1.001,1.052)	0.043
		MR Egger	78						1.023(0.957,1.093)	0.501
		Maximum likelihood	78						1.027(1.001,1.053)	0.04
		Weighted median	78						1.039(0.999,1.081)	0.055
		MR PRESSO	78						——	——
	Schizophrenia	Inverse variance weighted	62	-0.004	0.428	122.655	0.503	4.92E-06	1.016(0.990,1.043)	0.233
		MR Egger	62						1.025(0.954,1.102)	0.502
		Maximum likelihood	62						1.016(0.992,1.041)	0.189
		Weighted median	62						1.009(0.97,1.049)	0.659
		MR PRESSO	62						1.015(0.995,1.037)	0.144
**CD**	ADHD	Inverse variance weighted	57	-0.013	0.044	92.473	0.394	0.002	1.004(0.978,1.030)	0.79
		MR Egger	57						1.065(1.001,1.133)	0.051
		Maximum likelihood	57						1.004(0.983,1.024)	0.733
		Weighted median	57						1.005(0.971,1.04)	0.796
		MR PRESSO	57						——	——
	Anxiety disorder	Inverse variance weighted	57	-0.004	0.749	45.315	-0.24	0.846	1.009(0.967,1.053)	0.673
		MR Egger	57						1.026(0.918,1.147)	0.648
		Maximum likelihood	57						1.009(0.967,1.053)	0.673
		Weighted median	57						1.018(0.953,1.086)	0.599
		MR PRESSO	57						——	——
	ASD	Inverse variance weighted	54	-0.01	0.13	70.75	0.251	0.052	1.013(0.992,1.035)	0.214

	MR Egger	54						1.056(0.997,1.12)	0.07
	Maximum likelihood	54						1.013(0.992,1.035)	0.214
	Weighted median	54						1.02(0.988,1.054)	0.227
	MR PRESSO	54						——	——
BD	Inverse variance weighted	51	-0.002	0.796	104.107	0.52	9.13E-05	0.998(0.977,1.019)	0.845
	MR Egger	51						0.984(0.937,1.033)	0.515
	Maximum likelihood	51						0.998(0.983,1.013)	0.778
	Weighted median	51						0.988(0.964,1.012)	0.312
	MR PRESSO	51						1(0.982,1.019)	0.994
AN	Inverse variance weighted	53	-0.008	0.213	74.857	0.305	0.021	1.012(0.988,1.038)	0.326
	MR Egger	53						1.048(0.988,1.112)	0.126
	Maximum likelihood	53						1.012(0.992,1.034)	0.244
	Weighted median	53						0.997(0.965,1.03)	0.849
	MR PRESSO	53						1.004(0.984,1.024)	0.732
MDD	Inverse variance weighted	52	0.003	0.457	68.929	0.26	0.048	0.997(0.983,1.011)	0.668
	MR Egger	52						0.986(0.954,1.019)	0.394
	Maximum likelihood	52						0.997(0.985,1.009)	0.616
	Weighted median	52						0.986(0.968,1.005)	0.154
	MR PRESSO	52						——	——
OCD	Inverse variance weighted	55	0.006	0.654	60.768	0.111	0.245	1.021(0.972,1.073)	0.41
	MR Egger	55						0.995(0.878,1.127)	0.937
	Maximum likelihood	55						1.021(0.971,1.074)	0.407
	Weighted median	55						1.046(0.963,1.135)	0.286
	MR PRESSO	55						——	——
PTSD	Inverse variance weighted	62	-0.005	0.425	77.637	0.214	0.074	1.020(0.999,1.042)	0.062
	MR Egger	62						1.042(0.985,1.101)	0.159
	Maximum likelihood	62						1.02(0.999,1.042)	0.062
	Weighted median	62						1.028(0.994,1.064)	0.108
	MR PRESSO	62						——	——

	Schizophrenia	Inverse variance weighted	51	0.005	0.476	184.654	0.729	2.55E-17	1.021(0.995,1.047)	0.11
		MR Egger	51						1.001(0.943,1.063)	0.977
		Maximum likelihood	51						1.022(1.008,1.036)	0.002
		Weighted median	51						1.008(0.984,1.032)	0.514
		MR PRESSO	51						1.018(1,1.036)	0.045
**UC**	ADHD	Inverse variance weighted	42	0.005	0.492	43.994	0.068	0.346	1.050(1.023,1.077)	0.0002
		MR Egger	42						1.024(0.95,1.104)	0.543
		Maximum likelihood	42						1.05(1.023,1.078)	0.0002
		Weighted median	42						1.052(1.013,1.094)	0.009
		MR PRESSO	42						——	——
	Anxiety disorder	Inverse variance weighted	42	-0.012	0.461	30.738	-0.33	0.879	1.056(1.000,1.115)	0.048
		MR Egger	42						1.125(0.945,1.34)	0.194
		Maximum likelihood	42						1.057(1.001,1.117)	0.047
		Weighted median	42						1.06(0.982,1.144)	0.133
		MR PRESSO	42						——	——
	ASD	Inverse variance weighted	39	-0.001	0.95	45.203	0.159	0.196	1.041(1.015,1.068)	0.002
		MR Egger	39						1.043(0.97,1.123)	0.261
		Maximum likelihood	39						1.042(1.015,1.069)	0.002
		Weighted median	39						1.06(1.02,1.101)	0.003
		MR PRESSO	39						——	——
	BD	Inverse variance weighted	35	0.005	0.48	60.549	0.438	0.003	0.996(0.970,1.022)	0.735
		MR Egger	35						0.97(0.9,1.046)	0.438
		Maximum likelihood	35						0.995(0.976,1.015)	0.646
		Weighted median	35						1.004(0.973,1.036)	0.806
		MR PRESSO	35						1.01(0.986,1.035)	0.405
	AN	Inverse variance weighted	35	0.929	0.929	23.362	-0.46	0.915	1.011(0.982,1.041)	0.458
		MR Egger	35						1.015(0.933,1.104)	0.736
		Maximum likelihood	35						1.011(0.982,1.041)	0.457
		Weighted median	35						0.994(0.955,1.034)	0.752

	MR PRESSO	35						——	——
MDD	Inverse variance weighted	36	0.005	0.263	48.957	0.285	0.059	1.007(0.992,1.022)	0.385
	MR Egger	36						0.984(0.943,1.027)	0.473
	Maximum likelihood	36						1.007(0.992,1.022)	0.381
	Weighted median	36						0.991(0.969,1.015)	0.467
	MR PRESSO	36						——	——
OCD	Inverse variance weighted	41	-0.043	0.011	46.484	0.139	0.223	1.029(0.969,1.092)	0.354
	MR Egger	41						1.245(1.069,1.45)	0.008
	Maximum likelihood	41						1.029(0.969,1.093)	0.35
	Weighted median	41						1.053(0.959,1.156)	0.282
	MR PRESSO	41						——	——
PTSD	Inverse variance weighted	46	0.001	0.942	48.059	0.064	0.35	1.026(0.999,1.052)	0.055
	MR Egger	46						1.023(0.958,1.093)	0.496
	Maximum likelihood	46						1.026(1,1.053)	0.054
	Weighted median	46						1.022(0.983,1.063)	0.273
	MR PRESSO	46						——	——
Schizophrenia	Inverse variance weighted	35	-0.004	0.619	67.623	0.497	0.001	1.005(0.979,1.030)	0.723
	MR Egger	35						1.023(0.95,1.101)	0.557
	Maximum likelihood	35						1.005(0.987,1.023)	0.609
	Weighted median	35						0.991(0.964,1.019)	0.521
	MR PRESSO	35						1.014(0.99,1.039)	0.24

ADHD: attention deficit hyperactivity disorder; ASD: autism spectrum disorder; BD: bipolar disorder; AN: anorexia nervosa; MDD: major depressive disorder; OCD: obsessive-compulsive disorder; PTSD: post-traumatic stress disorder; IBD: inflammatory bowel disease; CD: Crohn's disease; UC: ulcerative colitis.

Table S5. The complementary MR analyses results of the causal effects of mental disorders on gastrointestinal disorders.										
**Exposure**	**Outcome**	**MR methods**	**No. SNPs**	**Egger**	**P-value**	**Cochran's**	**I2**	**P value**	**OR (95%CI)**	**P value**
**ADHD**	IBD	Inverse variance weighted	169	-0.007	0.282	206.162	0.185	0.024	1.038(0.996,1.081)	0.077
		MR Egger	169						1.128(0.963,1.322)	0.138
		Maximum likelihood	169						1.039(0.996,1.083)	0.077
		Weighted median	169						1.033(0.971,1.098)	0.302
		MR PRESSO	169						——	——
	CD	Inverse variance weighted	169	-0.013	0.351	127.976	0.351	0.001	1.029(0.973,1.088)	0.313
		MR Egger	169						1.17(0.946,1.447)	0.15
		Maximum likelihood	169						1.03(0.973,1.091)	0.311
		Weighted median	169						0.99(0.91,1.076)	0.807
		MR PRESSO	169						0.978(0.893,1.071)	0.632
	UC	Inverse variance weighted	169	-0.007	0.379	203.273	0.174	0.033	1.032(0.980,1.087)	0.229
		MR Egger	169						1.124(0.922,1.371)	0.249
		Maximum likelihood	169						1.033(0.98,1.089)	0.232
		Weighted median	169						1.03(0.955,1.11)	0.447
		MR PRESSO	169						——	——
**Anxiety disorder**	IBD	Inverse variance weighted	82	-0.002	0.719	89.516	0.095	0.242	0.993(0.968,1.019)	0.599
		MR Egger	82						1.003(0.944,1.066)	0.917
		Maximum likelihood	82						0.993(0.967,1.019)	0.59
		Weighted median	82						0.996(0.959,1.035)	0.848
		MR PRESSO	82						——	——
	CD	Inverse variance weighted	82	-0.001	0.932	99.219	0.184	0.083	0.973(0.939,1.007)	0.115
		MR Egger	82						0.976(0.895,1.064)	0.583

		Maximum likelihood	82						0.972(0.938,1.007)	0.117
		Weighted median	82						0.982(0.93,1.037)	0.51
		MR PRESSO	82						——	——
	UC	Inverse variance weighted	82	-0.002	0.805	109.102	0.258	0.02	1.006(0.969,1.044)	0.757
		MR Egger	82						1.016(0.933,1.105)	0.722
		Maximum likelihood	82						1.006(0.974,1.04)	0.716
		Weighted median	82						0.998(0.95,1.05)	0.95
		MR PRESSO	82						——	——
**ASD**	IBD	Inverse variance weighted	124	-0.013	0.035	146.082	0.158	0.076	0.998(0.951,1.048)	0.941
		MR Egger	124						1.157(1,1.338)	0.052
		Maximum likelihood	124						0.998(0.949,1.05)	0.943
		Weighted median	124						0.97(0.902,1.042)	0.406
		MR PRESSO	124						——	——
	CD	Inverse variance weighted	124	-0.021	0.017	151.167	0.186	0.043	1.009(0.944,1.078)	0.798
		MR Egger	124						1.271(1.04,1.554)	0.021
		Maximum likelihood	124						1.009(0.942,1.081)	0.798
		Weighted median	124						0.992(0.897,1.097)	0.878
		MR PRESSO	124						——	——
	UC	Inverse variance weighted	124	-0.012	0.144	148.9	0.174	0.056	0.990(0.931,1.052)	0.741
		MR Egger	124						1.128(0.936,1.36)	0.209
		Maximum likelihood	124						0.989(0.929,1.054)	0.742
		Weighted median	124						0.91(0.827,1.001)	0.053
		MR PRESSO	124						1.024(0.961,1.09)	0.466
**BD**	IBD	Inverse variance weighted	317	-0.005	0.252	434.076	0.272	0.0000112	1.138(1.084,1.194)	0.00000019
		MR Egger	317						1.25(1.057,1.479)	0.01

		Maximum likelihood	317						1.14(1.092,1.19)	1.96E-09
		Weighted median	317						1.131(1.063,1.203)	0.000105293
		MR PRESSO	317						1.119(1.072,1.167)	4.0137E-07
	CD	Inverse variance weighted	317	-0.008	0.199	416.138	0.241	0.0001299	1.158(1.094,1.225)	0.000000362
		MR Egger	317						1.333(1.066,1.666)	0.012
		Maximum likelihood	317						1.16(1.095,1.23)	0.000000545
		Weighted median	317						1.131(1.039,1.231)	0.004
		MR PRESSO	317						1.151(1.089,1.216)	9.21562E-07
	UC	Inverse variance weighted	317	-0.005	0.384	389.907	0.19	0.003	1.137(1.079,1.198)	1.40247E-06
		MR Egger	317						1.238(1.013,1.514)	0.038
		Maximum likelihood	317						1.139(1.08,1.202)	1.86419E-06
		Weighted median	317						1.117(1.031,1.21)	0.007
		MR PRESSO	317						1.13(1.075,1.188)	2.7361E-06
**AN**	IBD	Inverse variance weighted	152	-0.005	0.432	188.876	0.201	0.02	1.039(0.995,1.085)	0.082
		MR Egger	152						1.098(0.949,1.272)	0.211
		Maximum likelihood	152						1.04(0.995,1.088)	0.084
		Weighted median	152						1.021(0.957,1.089)	0.526
		MR PRESSO	152						1.029(0.984,1.077)	0.214
	CD	Inverse variance weighted	152	-0.001	0.943	166.478	0.093	0.184	1.055(0.994,1.119)	0.076
		MR Egger	152						1.062(0.88,1.281)	0.535
		Maximum likelihood	152						1.056(0.994,1.122)	0.078
		Weighted median	152						1.035(0.949,1.128)	0.439
		MR PRESSO	152						1.043(0.986,1.103)	0.145
	UC	Inverse variance weighted	152	-0.008	0.306	168.694	0.105	0.154	1.020(0.966,1.077)	0.476
		MR Egger	152						1.112(0.934,1.323)	0.235

		Maximum likelihood	152					1.021(0.965,1.079)	0.476
		Weighted median	152					1.032(0.955,1.115)	0.424
		MR PRESSO	152					1.016(0.962,1.072)	0.568
**MDD**	IBD	Inverse variance weighted	125	0.007	0.179	132.897	0.067	0.276	1.074(0.991,1.165)	0.084
		MR Egger	125						0.953(0.785,1.156)	0.625
		Maximum likelihood	125						1.076(0.99,1.17)	0.085
		Weighted median	125						1.082(0.958,1.222)	0.203
		MR PRESSO	125						——	——
	CD	Inverse variance weighted	125	0.009	0.155	125.088	0.009	0.456	1.059(0.948,1.183)	0.312
		MR Egger	125						0.896(0.694,1.155)	0.397
		Maximum likelihood	125						1.06(0.947,1.188)	0.311
		Weighted median	125						1.098(0.937,1.288)	0.248
		MR PRESSO	125						——	——
	UC	Inverse variance weighted	125	0.004	0.504	128.929	0.038	0.363	1.113(1.005,1.233)	0.039
		MR Egger	125						1.034(0.813,1.315)	0.788
		Maximum likelihood	125						1.115(1.004,1.238)	0.042
		Weighted median	125						1.035(0.893,1.2)	0.65
		MR PRESSO	125						——	——
**OCD**	IBD	Inverse variance weighted	89	-0.016	0.018	98.32	0.105	0.212	1.015(0.991,1.039)	0.215
		MR Egger	89						1.091(1.024,1.162)	0.009
		Maximum likelihood	89						1.015(0.991,1.04)	0.223
		Weighted median	89						1.019(0.986,1.054)	0.267
		MR PRESSO	89						——	——
	CD	Inverse variance weighted	89	-0.005	0.656	117.394	0.25	0.02	1.002(0.965,1.040)	0.927
		MR Egger	89						1.023(0.927,1.128)	0.655

		Maximum likelihood	89					1.002(0.969,1.036)	0.912
		Weighted median	89					1.024(0.975,1.074)	0.347
		MR PRESSO	89					——	——
	UC	Inverse variance weighted	89	-0.02	0.018	88.517	0.006	0.464	1.030(1.000,1.061)	0.049
		MR Egger	89						1.124(1.041,1.214)	0.004
		Maximum likelihood	89						1.03(1,1.062)	0.051
		Weighted median	89						1.046(1.001,1.092)	0.045
		MR PRESSO	89						——	——
**PTSD**	IBD	Inverse variance weighted	115	0.004	0.545	138.359	0.176	0.06	0.989(0.941,1.041)	0.68
		MR Egger	115						0.957(0.847,1.081)	0.478
		Maximum likelihood	115						0.989(0.939,1.042)	0.687
		Weighted median	115						0.955(0.884,1.032)	0.245
		MR PRESSO	115						——	——
	CD	Inverse variance weighted	115	-0.005	0.599	123.247	0.075	0.261	1.006(0.939,1.078)	0.862
		MR Egger	115						1.045(0.891,1.226)	0.586
		Maximum likelihood	115						1.006(0.937,1.081)	0.859
		Weighted median	115						1.009(0.908,1.121)	0.869
		MR PRESSO	115						——	——
	UC	Inverse variance weighted	115	0.005	0.514	133.349	0.145	0.104	1.002(0.941,1.067)	0.951
		MR Egger	115						0.959(0.826,1.113)	0.579
		Maximum likelihood	115						1.002(0.939,1.07)	0.952
		Weighted median	115						0.973(0.882,1.073)	0.58
		MR PRESSO	115						——	——
**Schizophrenia**	IBD	Inverse variance weighted	442	-0.005	0.233	610.118	0.277	1.548E-07	1.115(1.071,1.161)	1.11842E-07
		MR Egger	442						1.21(1.053,1.39)	0.007

	Maximum likelihood	442						1.116(1.078,1.156)	9.63478E-10
	Weighted median	442						1.134(1.076,1.196)	2.77308E-06
	MR PRESSO	442						1.115(1.078,1.153)	6.15E-10
CD	Inverse variance weighted	442	-0.009	0.092	623.914	0.293	2.01E-08	1.098(1.039,1.161)	0.001
	MR Egger	442						1.286(1.062,1.559)	0.01
	Maximum likelihood	442						1.099(1.048,1.153)	0.000115379
	Weighted median	442						1.101(1.024,1.184)	0.009
	MR PRESSO	442						1.067(1.018,1.118)	0.007
UC	Inverse variance weighted	442	-0.004	0.437	508.623	0.133	0.014	1.145(1.096,1.195)	8.4E-10
	MR Egger	442						1.216(1.037,1.426)	0.016
	Maximum likelihood	442						1.146(1.097,1.198)	1.4E-09
	Weighted median	442						1.149(1.077,1.224)	0.0000216
	MR PRESSO	442						1.149(1.102,1.198)	1.23343E-10

ADHD: attention deficit hyperactivity disorder; ASD: autism spectrum disorder; BD: bipolar disorder; AN: anorexia nervosa; MDD: major depressive disorder; OCD: obsessive-compulsive disorder; PTSD: post-traumatic stress disorder; IBD: inflammatory bowel disease; CD: Crohn's disease; UC: ulcerative colitis.

Table S6. Raw data for the MR analysis of the causal effect of IBD on ADHD

SNP	Effect allele	Other allele	Effect allele frequency	Beta.exposure	Se.exposure	Beta.outcome	Se.outcome
**rs1003342**	G	A	0.531	-0.095	0.017	-0.013	0.014
**rs10045431**	C	A	0.718	0.177	0.019	0.013	0.015
**rs10175585**	A	G	0.713	-0.133	0.02	-0.017	0.016
**rs10737481**	G	T	0.555	0.141	0.017	0.019	0.014
**rs10761659**	G	A	0.554	0.162	0.017	-0.015	0.014
**rs10800314**	A	C	0.649	-0.143	0.018	-0.015	0.014
**rs10826797**	T	G	0.306	-0.102	0.019	0.01	0.015
**rs10917547**	T	A	0.362	-0.1	0.018	-0.018	0.014
**rs11209026**	A	G	0.054	-0.726	0.042	0	0.029
**rs11236797**	A	C	0.47	0.156	0.017	0.026	0.014
**rs112401990**	A	G	0.375	0.142	0.017	-0.039	0.014
**rs112694524**	A	G	0.09	0.188	0.03	0.019	0.027
**rs112874012**	T	C	0.045	-0.25	0.045	0.009	0.033
**rs113825849**	G	C	0.014	0.584	0.08	0.017	0.053
**rs11548656**	G	A	0.034	-0.293	0.051	0.039	0.04
**rs11677953**	A	G	0.415	0.098	0.017	-0.001	0.014
**rs117292830**	A	G	0.027	0.43	0.058	0.009	0.044
**rs11949375**	C	T	0.098	0.189	0.028	-0.011	0.026
**rs12446550**	A	G	0.41	0.108	0.017	0.005	0.014
**rs1250573**	A	G	0.296	-0.114	0.019	0.022	0.015
**rs12764283**	A	G	0.337	0.127	0.018	0.002	0.014
**rs12936409**	T	C	0.476	0.146	0.017	-0.003	0.014
**rs131657**	A	T	0.202	0.136	0.021	-0.024	0.017
**rs140892874**	C	T	0.027	0.41	0.051	0.058	0.052
**rs142770866**	A	G	0.081	0.23	0.034	0.027	0.026
**rs144515162**	G	A	0.672	-0.15	0.022	-0.064	0.047
**rs145126485**	C	A	0.033	0.283	0.05	0.035	0.047
**rs1551399**	C	A	0.616	0.101	0.017	0.021	0.014
**rs1736161**	A	G	0.429	-0.123	0.017	0.031	0.014
**rs1873625**	A	C	0.323	0.177	0.018	-0.005	0.015
**rs1886731**	C	T	0.481	-0.097	0.018	-0.029	0.015
**rs1887428**	C	G	0.619	-0.172	0.018	-0.002	0.015
**rs2076756**	G	A	0.271	0.188	0.019	0.032	0.016
**rs2129944**	G	T	0.291	-0.119	0.02	-0.014	0.023
**rs2193041**	G	A	0.389	0.134	0.017	-0.002	0.014
**rs2230365**	T	C	0.163	0.154	0.023	-0.006	0.019
**rs2241878**	C	T	0.538	0.148	0.017	-0.01	0.014
**rs2542147**	T	G	0.837	-0.151	0.023	-0.019	0.017
**rs254559**	A	C	0.402	0.103	0.017	0.014	0.014
**rs2836882**	A	G	0.258	-0.196	0.02	0.007	0.015
**rs28383456**	T	C	0.345	-0.178	0.02	-0.027	0.039
**rs3024493**	A	C	0.17	0.213	0.022	-0.001	0.018

**rs3091316**	A	G	0.267	-0.112	0.019	0.014	0.015
**rs34190331**	A	G	0.085	0.177	0.03	-0.017	0.028
**rs34920465**	G	A	0.172	-0.139	0.023	-0.018	0.019
**rs35260072**	C	A	0.433	0.142	0.017	0.002	0.014
**rs35730213**	C	G	0.263	-0.151	0.019	-0.004	0.015
**rs3850378**	C	T	0.096	0.155	0.028	-0.008	0.023
**rs4077515**	T	C	0.424	0.179	0.017	-0.029	0.014
**rs4246905**	C	T	0.73	0.163	0.02	0.012	0.016
**rs444210**	G	A	0.546	0.11	0.017	-0.021	0.014
**rs45528737**	T	C	0.112	0.167	0.03	-0.038	0.025
**rs4712528**	C	G	0.788	0.123	0.021	-0.002	0.016
**rs4730272**	G	A	0.513	-0.134	0.018	-0.01	0.014
**rs56062135**	T	C	0.237	0.151	0.02	0.005	0.016
**rs56167332**	A	C	0.346	0.156	0.019	0.014	0.017
**rs6062496**	A	G	0.578	0.165	0.018	-0.012	0.015
**rs6584283**	C	T	0.511	-0.18	0.017	-0.018	0.014
**rs6826501**	T	C	0.536	-0.093	0.017	0.007	0.014
**rs6873866**	C	T	0.529	-0.107	0.018	0.017	0.014
**rs6880778**	G	A	0.621	0.188	0.017	-0.009	0.014
**rs6911490**	C	T	0.779	-0.143	0.021	-0.008	0.017
**rs6927172**	G	C	0.214	0.11	0.02	0.025	0.016
**rs72798422**	C	T	0.042	0.278	0.043	0.076	0.038
**rs7282490**	A	G	0.598	-0.145	0.017	-0.006	0.014
**rs7285952**	G	T	0.157	-0.176	0.024	0.031	0.019
**rs744166**	G	A	0.41	-0.121	0.017	-0.007	0.014
**rs7523335**	A	G	0.173	-0.141	0.023	-0.023	0.017
**rs75565243**	A	G	0.029	0.349	0.055	0.073	0.041
**rs7797798**	G	C	0.416	-0.096	0.017	-0.013	0.014
**rs9272514**	T	C	0.306	-0.235	0.021	-0.026	0.017
**rs9370774**	C	T	0.201	-0.131	0.022	0.008	0.018
**rs9934775**	T	C	0.161	-0.14	0.023	0.004	0.019

Table S7. Raw data for the MR analysis of the causal effect of CD on ADHD							
SNP	Effect allele	Other allele	Effect allele frequency	Beta.exposure	Se.exposure	Beta.outcome	Se.outcome
**rs10045431**	C	A	0.716	0.216	0.026	0.013	0.015
**rs10055349**	A	G	0.222	0.214	0.027	-0.012	0.016
**rs1056441**	C	T	0.698	0.167	0.026	-0.005	0.015
**rs10748781**	A	C	0.551	-0.219	0.024	-0.019	0.014
**rs10761659**	G	A	0.553	0.212	0.024	-0.015	0.014
**rs11209026**	A	G	0.056	-0.995	0.064	0	0.029
**rs11236797**	A	C	0.473	0.181	0.023	0.026	0.014
**rs112401990**	A	G	0.373	0.132	0.024	-0.039	0.014
**rs114607072**	T	G	0.04	0.442	0.063	0.073	0.037
**rs11564236**	T	A	0.034	0.519	0.06	0.057	0.052
**rs12194825**	A	T	0.186	-0.172	0.03	0.015	0.018
**rs1250573**	A	G	0.287	-0.171	0.026	0.022	0.015
**rs12692254**	T	A	0.543	0.301	0.023	-0.01	0.014
**rs12717899**	T	G	0.794	0.159	0.029	-0.018	0.017
**rs12936409**	T	C	0.471	0.138	0.023	-0.003	0.014
**rs1297271**	T	C	0.43	-0.155	0.024	0.031	0.014
**rs13135092**	G	A	0.095	0.221	0.039	0.015	0.029
**rs143345302**	T	C	0.134	0.212	0.036	0.012	0.021
**rs145126485**	C	A	0.036	0.627	0.062	0.035	0.047
**rs1456896**	T	C	0.698	0.139	0.025	0.007	0.015
**rs147018773**	T	C	0.097	0.322	0.038	-0.01	0.026
**rs147684209**	C	T	0.369	0.155	0.024	0.002	0.014
**rs151314883**	A	G	0.158	-0.224	0.033	0.028	0.019
**rs1873625**	A	C	0.32	0.181	0.024	-0.005	0.015
**rs1887428**	C	G	0.623	-0.168	0.024	-0.002	0.015
**rs1932990**	T	C	0.254	0.153	0.026	0.029	0.016
**rs2076756**	G	A	0.284	0.4	0.024	0.032	0.016
**rs2129944**	G	T	0.291	-0.156	0.027	-0.014	0.023
**rs2188962**	T	C	0.44	0.212	0.023	0.003	0.014
**rs2505640**	G	A	0.644	-0.146	0.024	-0.007	0.014
**rs281379**	A	G	0.489	0.14	0.024	-0.034	0.014
**rs2856997**	A	C	0.421	0.141	0.023	-0.007	0.014
**rs28701841**	A	G	0.117	0.224	0.037	0.041	0.023
**rs3024505**	A	G	0.162	0.178	0.03	0	0.018
**rs3091315**	G	A	0.266	-0.18	0.026	0.012	0.015
**rs3810936**	C	T	0.698	0.208	0.026	0.017	0.015
**rs4077515**	T	C	0.42	0.216	0.024	-0.029	0.014
**rs444210**	G	A	0.547	0.163	0.023	-0.021	0.014
**rs4486887**	T	C	0.694	-0.196	0.025	-0.005	0.015
**rs4820091**	G	T	0.198	0.172	0.028	-0.025	0.017
**rs4851586**	C	T	0.76	-0.169	0.026	-0.017	0.016
**rs4902642**	A	G	0.409	-0.129	0.024	-0.014	0.014

**rs56062135**	T	C	0.234	0.193	0.027	0.005	0.016
**rs56167332**	A	C	0.343	0.17	0.026	0.014	0.017
**rs6588243**	C	A	0.59	0.132	0.023	-0.036	0.014
**rs6704109**	T	C	0.256	0.202	0.026	-0.007	0.016
**rs6873866**	C	T	0.535	-0.168	0.024	0.017	0.014
**rs697693**	A	G	0.201	0.172	0.028	0.028	0.017
**rs7276302**	G	A	0.608	-0.172	0.023	-0.009	0.014
**rs72798422**	C	T	0.048	0.59	0.051	0.076	0.038
**rs74179925**	T	A	0.205	-0.173	0.03	0.024	0.017
**rs7423615**	T	C	0.194	0.164	0.029	-0.029	0.018
**rs744166**	G	A	0.408	-0.129	0.023	-0.007	0.014
**rs7543234**	T	C	0.239	0.155	0.027	0.022	0.015
**rs7713270**	T	C	0.624	0.297	0.024	-0.013	0.014
**rs78487399**	C	G	0.101	0.226	0.037	0.011	0.023
**rs80262450**	A	G	0.113	0.283	0.035	0.012	0.019
**rs921720**	G	A	0.619	0.163	0.024	0.023	0.014

Table S8. Raw data for the MR analysis of the causal effect of UC on ADHD

SNP	Effect allele	Other allele	Effect allele frequency	Beta.exposure	Se.exposure	Beta.outcome	Se.outcome
**rs10045431**	C	A	0.713	0.145	0.024	0.013	0.015
**rs10182512**	A	G	0.35	0.161	0.022	-0.037	0.014
**rs10272963**	T	C	0.426	-0.172	0.022	-0.008	0.014
**rs10737481**	G	T	0.556	0.25	0.022	0.019	0.014
**rs10917547**	T	A	0.362	-0.173	0.022	-0.018	0.014
**rs11209026**	A	G	0.059	-0.562	0.052	0	0.029
**rs114152040**	A	G	0.032	0.34	0.062	0.014	0.037
**rs117292830**	A	G	0.027	0.615	0.07	0.009	0.044
**rs12612675**	G	A	0.403	0.123	0.022	-0.003	0.014
**rs12817473**	G	A	0.382	0.191	0.022	0	0.014
**rs1317209**	A	G	0.188	0.146	0.027	0.031	0.018
**rs1359946**	A	G	0.196	0.158	0.027	0.04	0.018
**rs137845**	G	A	0.515	0.118	0.021	0.021	0.014
**rs143210366**	G	T	0.037	0.39	0.057	0.015	0.042
**rs144515162**	G	A	0.671	-0.232	0.027	-0.064	0.047
**rs1801274**	G	A	0.483	-0.183	0.022	-0.014	0.014
**rs1886731**	C	T	0.481	-0.141	0.022	-0.029	0.015
**rs1887428**	C	G	0.623	-0.177	0.022	-0.002	0.015
**rs2212434**	T	C	0.46	0.142	0.021	0.025	0.014
**rs2301989**	A	G	0.402	-0.141	0.022	-0.012	0.014
**rs254559**	A	C	0.404	0.124	0.022	0.014	0.014
**rs28383456**	T	C	0.335	-0.337	0.026	-0.027	0.039
**rs3024493**	A	C	0.168	0.236	0.028	-0.001	0.018
**rs34920465**	G	A	0.17	-0.193	0.029	-0.018	0.019
**rs35730213**	C	G	0.267	-0.167	0.025	-0.004	0.015
**rs3829111**	A	G	0.417	0.156	0.021	-0.028	0.014
**rs45627734**	A	G	0.03	0.406	0.063	0.075	0.041
**rs4574921**	T	C	0.741	0.151	0.026	0.001	0.016
**rs483905**	A	G	0.294	0.129	0.023	0.002	0.015
**rs484356**	G	C	0.328	-0.134	0.023	-0.003	0.014
**rs56167332**	A	C	0.342	0.152	0.023	0.014	0.017
**rs6017342**	C	A	0.538	0.191	0.024	-0.005	0.015
**rs6062496**	A	G	0.573	0.158	0.022	-0.012	0.015
**rs6933404**	C	T	0.216	0.167	0.025	0.024	0.017
**rs7282490**	A	G	0.604	-0.14	0.021	-0.006	0.014
**rs7523335**	A	G	0.177	-0.17	0.029	-0.023	0.017
**rs7752873**	T	C	0.137	0.182	0.03	0.032	0.021
**rs7911680**	C	A	0.49	-0.172	0.021	-0.017	0.014
**rs798502**	C	A	0.283	-0.136	0.024	-0.023	0.015
**rs9272514**	T	C	0.298	-0.402	0.027	-0.026	0.017
**rs9823546**	A	T	0.31	0.177	0.022	-0.001	0.015
**rs9891174**	A	T	0.47	0.145	0.021	-0.002	0.014

**rs9977672**	A	G	0.251	-0.245	0.026	0.007	0.016

Table S9. Raw data for the MR analysis of the causal effect of IBD on anxiety disorder							
**SNP**	**Effect allele**	**Other allele**	**Effect allele frequency**	**Beta.exposure**	**Se.exposure**	**Beta.outcome**	**Se.outcome**
**rs1003342**	G	A	0.531	-0.095	0.017	-0.019	0.026
**rs10045431**	C	A	0.718	0.177	0.019	-0.017	0.029
**rs10175585**	A	G	0.713	-0.133	0.02	0.011	0.033
**rs10408351**	A	G	0.238	0.138	0.022	-0.057	0.036
**rs10737481**	G	T	0.555	0.141	0.017	0.015	0.027
**rs10761659**	G	A	0.554	0.162	0.017	0.013	0.029
**rs10800314**	A	C	0.649	-0.143	0.018	-0.002	0.031
**rs10826797**	T	G	0.306	-0.102	0.019	-0.021	0.031
**rs10917547**	T	A	0.362	-0.1	0.018	-0.008	0.03
**rs11209026**	A	G	0.054	-0.726	0.042	-0.06	0.057
**rs11236797**	A	C	0.47	0.156	0.017	0.014	0.027
**rs112401990**	A	G	0.375	0.142	0.017	0.055	0.03
**rs112694524**	A	G	0.09	0.188	0.03	-0.027	0.058
**rs112874012**	T	C	0.045	-0.25	0.045	-0.175	0.111
**rs115312361**	C	A	0.331	-0.184	0.023	-0.019	0.07
**rs116465569**	C	T	0.61	-0.201	0.021	0.077	0.061
**rs11677953**	A	G	0.415	0.098	0.017	0.01	0.027
**rs11949375**	C	T	0.098	0.189	0.028	-0.018	0.053
**rs12446550**	A	G	0.41	0.108	0.017	0.031	0.031
**rs1250573**	A	G	0.296	-0.114	0.019	0.006	0.029
**rs12764283**	A	G	0.337	0.127	0.018	0.018	0.028
**rs12936409**	T	C	0.476	0.146	0.017	-0.031	0.027
**rs131657**	A	T	0.202	0.136	0.021	0.014	0.044
**rs142770866**	A	G	0.081	0.23	0.034	0.096	0.083
**rs144515162**	G	A	0.672	-0.15	0.022	0.018	0.067
**rs1551399**	C	A	0.616	0.101	0.017	-0.018	0.034
**rs1736161**	A	G	0.429	-0.123	0.017	-0.027	0.027
**rs1873625**	A	C	0.323	0.177	0.018	0.006	0.029
**rs1886731**	C	T	0.481	-0.097	0.018	-0.074	0.029
**rs1887428**	C	G	0.619	-0.172	0.018	0.032	0.029
**rs2076756**	G	A	0.271	0.188	0.019	-0.031	0.036
**rs2129944**	G	T	0.291	-0.119	0.02	-0.006	0.036
**rs2193041**	G	A	0.389	0.134	0.017	-0.003	0.03
**rs2230365**	T	C	0.163	0.154	0.023	0.019	0.043
**rs2241878**	C	T	0.538	0.148	0.017	-0.005	0.029
**rs2542147**	T	G	0.837	-0.151	0.023	-0.036	0.037
**rs254559**	A	C	0.402	0.103	0.017	0.005	0.027
**rs2836882**	A	G	0.258	-0.196	0.02	-0.043	0.031
**rs28383456**	T	C	0.345	-0.178	0.02	-0.029	0.059
**rs3024493**	A	C	0.17	0.213	0.022	0.015	0.036
**rs3091316**	A	G	0.267	-0.112	0.019	-0.033	0.029
**rs34190331**	A	G	0.085	0.177	0.03	0.008	0.053

**rs34920465**	G	A	0.172	-0.139	0.023	0.013	0.035
**rs35260072**	C	A	0.433	0.142	0.017	0.007	0.03
**rs35730213**	C	G	0.263	-0.151	0.019	-0.064	0.03
**rs3850378**	C	T	0.096	0.155	0.028	-0.041	0.045
**rs4077515**	T	C	0.424	0.179	0.017	0.013	0.028
**rs4246905**	C	T	0.73	0.163	0.02	0.031	0.03
**rs444210**	G	A	0.546	0.11	0.017	-0.008	0.027
**rs45528737**	T	C	0.112	0.167	0.03	0.052	0.058
**rs4676410**	A	G	0.195	0.155	0.023	0.051	0.045
**rs4712528**	C	G	0.788	0.123	0.021	0.017	0.033
**rs4730272**	G	A	0.513	-0.134	0.018	0.019	0.032
**rs4807569**	C	A	0.22	0.139	0.021	-0.043	0.042
**rs56062135**	T	C	0.237	0.151	0.02	-0.052	0.035
**rs56167332**	A	C	0.346	0.156	0.019	-0.025	0.03
**rs6062496**	A	G	0.578	0.165	0.018	0.026	0.03
**rs6584283**	C	T	0.511	-0.18	0.017	0.015	0.029
**rs6826501**	T	C	0.536	-0.093	0.017	-0.037	0.027
**rs6873866**	C	T	0.529	-0.107	0.018	0.017	0.028
**rs6880778**	G	A	0.621	0.188	0.017	-0.011	0.03
**rs6911490**	C	T	0.779	-0.143	0.021	-0.023	0.035
**rs6927172**	G	C	0.214	0.11	0.02	0.048	0.032
**rs72798422**	C	T	0.042	0.278	0.043	-0.26	0.276
**rs7282490**	A	G	0.598	-0.145	0.017	-0.039	0.027
**rs7285952**	G	T	0.157	-0.176	0.024	0.004	0.036
**rs744166**	G	A	0.41	-0.121	0.017	0.004	0.029
**rs7523335**	A	G	0.173	-0.141	0.023	0.037	0.037
**rs7797798**	G	C	0.416	-0.096	0.017	-0.006	0.027
**rs9272514**	T	C	0.306	-0.235	0.021	-0.052	0.061
**rs9370774**	C	T	0.201	-0.131	0.022	-0.038	0.036
**rs9934775**	T	C	0.161	-0.14	0.023	0.021	0.037

Table S10. Raw data for the MR analysis of the causal effect of CD on anxiety disorder							
SNP	Effect allele	Other allele	Effect allele frequency	Beta.exposure	Se.exposure	Beta.outcome	Se.outcome
**rs10045431**	C	A	0.716	0.216	0.026	-0.017	0.029
**rs10055349**	A	G	0.222	0.214	0.027	0.024	0.032
**rs1056441**	C	T	0.698	0.167	0.026	0.028	0.029
**rs10748781**	A	C	0.551	-0.219	0.024	0.003	0.029
**rs10761659**	G	A	0.553	0.212	0.024	0.013	0.029
**rs11209026**	A	G	0.056	-0.995	0.064	-0.06	0.057
**rs11236797**	A	C	0.473	0.181	0.023	0.014	0.027
**rs112401990**	A	G	0.373	0.132	0.024	0.055	0.03
**rs12194825**	A	T	0.186	-0.172	0.03	0.001	0.034
**rs1250573**	A	G	0.287	-0.171	0.026	0.006	0.029
**rs12692254**	T	A	0.543	0.301	0.023	-0.032	0.033
**rs12717899**	T	G	0.794	0.159	0.029	-0.004	0.032
**rs12936409**	T	C	0.471	0.138	0.023	-0.031	0.027
**rs1297271**	T	C	0.43	-0.155	0.024	-0.021	0.027
**rs13135092**	G	A	0.095	0.221	0.039	0.052	0.057
**rs143345302**	T	C	0.134	0.212	0.036	0.124	0.068
**rs1456896**	T	C	0.698	0.139	0.025	-0.045	0.028
**rs147018773**	T	C	0.097	0.322	0.038	0.012	0.06
**rs147684209**	C	T	0.369	0.155	0.024	0.019	0.034
**rs151314883**	A	G	0.158	-0.224	0.033	0.027	0.039
**rs1873625**	A	C	0.32	0.181	0.024	0.006	0.029
**rs1887428**	C	G	0.623	-0.168	0.024	0.032	0.029
**rs1932990**	T	C	0.254	0.153	0.026	0.063	0.031
**rs2076756**	G	A	0.284	0.4	0.024	-0.031	0.036
**rs2129944**	G	T	0.291	-0.156	0.027	-0.006	0.036
**rs2188962**	T	C	0.44	0.212	0.023	0.003	0.03
**rs2505640**	G	A	0.644	-0.146	0.024	-0.018	0.027
**rs281379**	A	G	0.489	0.14	0.024	0.036	0.033
**rs2856997**	A	C	0.421	0.141	0.023	0.003	0.03
**rs28701841**	A	G	0.117	0.224	0.037	0.025	0.048
**rs3024505**	A	G	0.162	0.178	0.03	0.057	0.039
**rs3091315**	G	A	0.266	-0.18	0.026	-0.009	0.033
**rs3810936**	C	T	0.698	0.208	0.026	0.024	0.029
**rs4077515**	T	C	0.42	0.216	0.024	0.013	0.028
**rs444210**	G	A	0.547	0.163	0.023	-0.008	0.027
**rs4486887**	T	C	0.694	-0.196	0.025	0.042	0.03
**rs4820091**	G	T	0.198	0.172	0.028	0.017	0.042
**rs4851586**	C	T	0.76	-0.169	0.026	0.00E+00	0.031
**rs4902642**	A	G	0.409	-0.129	0.024	0.027	0.027
**rs56062135**	T	C	0.234	0.193	0.027	-0.052	0.035
**rs56167332**	A	C	0.343	0.17	0.026	-0.025	0.03
**rs6588243**	C	A	0.59	0.132	0.023	-0.013	0.027

**rs6704109**	T	C	0.256	0.202	0.026	-0.038	0.035
**rs6873866**	C	T	0.535	-0.168	0.024	0.017	0.028
**rs697693**	A	G	0.201	0.172	0.028	-0.014	0.033
**rs7276302**	G	A	0.608	-0.172	0.023	-0.033	0.027
**rs72798422**	C	T	0.048	0.59	0.051	-0.26	0.276
**rs74179925**	T	A	0.205	-0.173	0.03	0.009	0.041
**rs7423615**	T	C	0.194	0.164	0.029	0.022	0.034
**rs744166**	G	A	0.408	-0.129	0.023	0.004	0.029
**rs7499231**	G	A	0.124	-0.239	0.042	0.004	0.06
**rs7543234**	T	C	0.239	0.155	0.027	0.04	0.035
**rs76532080**	T	C	0.068	0.358	0.052	0.158	0.097
**rs7713270**	T	C	0.624	0.297	0.024	-0.022	0.031
**rs78487399**	C	G	0.101	0.226	0.037	0.005	0.05
**rs80262450**	A	G	0.113	0.283	0.035	-0.007	0.047
**rs8178977**	C	G	0.239	0.193	0.027	-0.032	0.034
**rs921720**	G	A	0.619	0.163	0.024	-0.045	0.035

Table S11. Raw data for the MR analysis of the causal effect of UC on anxiety disorder							
**SNP**	**Effect allele**	**Other allele**	**Effect allele frequency**	**Beta.exposure**	**Se.exposure**	**Beta.outcome**	**Se.outcome**
**rs10045431**	C	A	0.713	0.145	0.024	-0.017	0.029
**rs10182512**	A	G	0.35	0.161	0.022	0.039	0.028
**rs10272963**	T	C	0.426	-0.172	0.022	0.02	0.027
**rs10737481**	G	T	0.556	0.25	0.022	0.015	0.027
**rs10917547**	T	A	0.362	-0.173	0.022	-0.008	0.03
**rs11209026**	A	G	0.059	-0.562	0.052	-0.06	0.057
**rs115312361**	C	A	0.329	-0.324	0.029	-0.019	0.07
**rs12612675**	G	A	0.403	0.123	0.022	-0.002	0.028
**rs12817473**	G	A	0.382	0.191	0.022	-0.004	0.03
**rs1317209**	A	G	0.188	0.146	0.027	0.002	0.038
**rs1359946**	A	G	0.196	0.158	0.027	-0.035	0.035
**rs137845**	G	A	0.515	0.118	0.021	0.013	0.027
**rs144515162**	G	A	0.671	-0.232	0.027	0.018	0.067
**rs144582178**	C	T	0.672	-0.276	0.028	0.026	0.066
**rs1801274**	G	A	0.483	-0.183	0.022	-0.005	0.027
**rs183231933**	T	C	0.24	-0.371	0.031	-0.025	0.077
**rs1886731**	C	T	0.481	-0.141	0.022	-0.074	0.029
**rs1887428**	C	G	0.623	-0.177	0.022	0.032	0.029
**rs2212434**	T	C	0.46	0.142	0.021	0.018	0.026
**rs2301989**	A	G	0.402	-0.141	0.022	-0.008	0.027
**rs254559**	A	C	0.404	0.124	0.022	0.005	0.027
**rs28383456**	T	C	0.335	-0.337	0.026	-0.029	0.059
**rs3024493**	A	C	0.168	0.236	0.028	0.015	0.036
**rs34920465**	G	A	0.17	-0.193	0.029	0.013	0.035
**rs35730213**	C	G	0.267	-0.167	0.025	-0.064	0.03
**rs3829111**	A	G	0.417	0.156	0.021	0.004	0.028
**rs4574921**	T	C	0.741	0.151	0.026	0.022	0.032
**rs4676410**	A	G	0.197	0.208	0.028	0.051	0.045
**rs483905**	A	G	0.294	0.129	0.023	-0.024	0.029
**rs484356**	G	C	0.328	-0.134	0.023	0.001	0.035
**rs56167332**	A	C	0.342	0.152	0.023	-0.025	0.03
**rs6017342**	C	A	0.538	0.191	0.024	-0.004	0.046
**rs6062496**	A	G	0.573	0.158	0.022	0.026	0.03
**rs6933404**	C	T	0.216	0.167	0.025	0.044	0.033
**rs7282490**	A	G	0.604	-0.14	0.021	-0.039	0.027
**rs7523335**	A	G	0.177	-0.17	0.029	0.037	0.037
**rs7752873**	T	C	0.137	0.182	0.03	0.018	0.04
**rs7911680**	C	A	0.49	-0.172	0.021	-0.009	0.029
**rs798502**	C	A	0.283	-0.136	0.024	0.053	0.029
**rs9272514**	T	C	0.298	-0.402	0.027	-0.052	0.061
**rs9823546**	A	T	0.31	0.177	0.022	0.004	0.029
**rs9891174**	A	T	0.47	0.145	0.021	-0.034	0.029

**rs9977672**	A	G	0.251	-0.245	0.026	-0.046	0.031

Table S12. Raw data for the MR analysis of the causal effect of IBD on ASD							
SNP	Effect allele	Other allele	Effect allele frequency	Beta.exposure	Se.exposure	Beta.outcome	Se.outcome
**rs1003342**	G	A	0.531	-0.095	0.017	-0.026	0.014
**rs10045431**	C	A	0.718	0.177	0.019	0.005	0.015
**rs10175585**	A	G	0.713	-0.133	0.02	-0.005	0.016
**rs10408351**	A	G	0.238	0.138	0.022	0.018	0.018
**rs10737481**	G	T	0.555	0.141	0.017	0.026	0.014
**rs10761659**	G	A	0.554	0.162	0.017	-0.007	0.014
**rs10800314**	A	C	0.649	-0.143	0.018	0	0.015
**rs10826797**	T	G	0.306	-0.102	0.019	-0.029	0.015
**rs10917547**	T	A	0.362	-0.1	0.018	0.006	0.014
**rs11209026**	A	G	0.054	-0.726	0.042	-0.045	0.03
**rs11236797**	A	C	0.47	0.156	0.017	0.013	0.014
**rs112401990**	A	G	0.375	0.142	0.017	-0.015	0.014
**rs112694524**	A	G	0.09	0.188	0.03	-0.005	0.029
**rs112874012**	T	C	0.045	-0.25	0.045	0.037	0.034
**rs113825849**	G	C	0.014	0.584	0.08	0.023	0.054
**rs11548656**	G	A	0.034	-0.293	0.051	0.06	0.042
**rs116465569**	C	T	0.61	-0.201	0.021	-0.03	0.016
**rs11677953**	A	G	0.415	0.098	0.017	-0.011	0.014
**rs117292830**	A	G	0.027	0.43	0.058	0.045	0.044
**rs11949375**	C	T	0.098	0.189	0.028	-0.033	0.027
**rs12446550**	A	G	0.41	0.108	0.017	0.01	0.014
**rs1250573**	A	G	0.296	-0.114	0.019	0.004	0.015
**rs12764283**	A	G	0.337	0.127	0.018	0.002	0.015
**rs12936409**	T	C	0.476	0.146	0.017	0.014	0.014
**rs131657**	A	T	0.202	0.136	0.021	-0.006	0.017
**rs140892874**	C	T	0.027	0.41	0.051	0.019	0.053
**rs142770866**	A	G	0.081	0.23	0.034	0.062	0.027
**rs144515162**	G	A	0.672	-0.15	0.022	-0.037	0.048
**rs145126485**	C	A	0.033	0.283	0.05	0.001	0.048
**rs1551399**	C	A	0.616	0.101	0.017	-0.008	0.014
**rs1736161**	A	G	0.429	-0.123	0.017	-0.001	0.014
**rs1873625**	A	C	0.323	0.177	0.018	0.032	0.015
**rs1886731**	C	T	0.481	-0.097	0.018	-0.032	0.014
**rs1887428**	C	G	0.619	-0.172	0.018	0.002	0.015
**rs2076756**	G	A	0.271	0.188	0.019	0.043	0.016
**rs2129944**	G	T	0.291	-0.119	0.02	0	0.023
**rs2193041**	G	A	0.389	0.134	0.017	-0.011	0.014
**rs2230365**	T	C	0.163	0.154	0.023	0.019	0.02
**rs2241878**	C	T	0.538	0.148	0.017	-0.004	0.014
**rs2542147**	T	G	0.837	-0.151	0.023	0.002	0.018
**rs254559**	A	C	0.402	0.103	0.017	0.004	0.014
**rs2836882**	A	G	0.258	-0.196	0.02	0	0.016

**rs28383456**	T	C	0.345	-0.178	0.02	0.019	0.04
**rs3024493**	A	C	0.17	0.213	0.022	0.034	0.018
**rs3091316**	A	G	0.267	-0.112	0.019	0.019	0.015
**rs34190331**	A	G	0.085	0.177	0.03	-0.069	0.029
**rs34920465**	G	A	0.172	-0.139	0.023	-0.004	0.019
**rs35260072**	C	A	0.433	0.142	0.017	0.005	0.014
**rs35730213**	C	G	0.263	-0.151	0.019	-0.018	0.015
**rs3850378**	C	T	0.096	0.155	0.028	0.019	0.024
**rs4077515**	T	C	0.424	0.179	0.017	-0.045	0.014
**rs4246905**	C	T	0.73	0.163	0.02	-0.006	0.016
**rs444210**	G	A	0.546	0.11	0.017	-0.026	0.014
**rs45528737**	T	C	0.112	0.167	0.03	0.015	0.024
**rs4676410**	A	G	0.195	0.155	0.023	-0.031	0.02
**rs4712528**	C	G	0.788	0.123	0.021	0.018	0.017
**rs4730272**	G	A	0.513	-0.134	0.018	-0.018	0.014
**rs4807569**	C	A	0.22	0.139	0.021	-0.008	0.018
**rs56062135**	T	C	0.237	0.151	0.02	-0.004	0.016
**rs56167332**	A	C	0.346	0.156	0.019	-0.001	0.016
**rs6062496**	A	G	0.578	0.165	0.018	0.024	0.015
**rs6584283**	C	T	0.511	-0.18	0.017	0.001	0.014
**rs6826501**	T	C	0.536	-0.093	0.017	0.019	0.014
**rs6873866**	C	T	0.529	-0.107	0.018	-0.017	0.014
**rs6880778**	G	A	0.621	0.188	0.017	0.006	0.014
**rs6911490**	C	T	0.779	-0.143	0.021	-0.011	0.017
**rs6927172**	G	C	0.214	0.11	0.02	0.005	0.017
**rs72798422**	C	T	0.042	0.278	0.043	0.062	0.039
**rs7282490**	A	G	0.598	-0.145	0.017	-0.001	0.014
**rs7285952**	G	T	0.157	-0.176	0.024	0.008	0.019
**rs744166**	G	A	0.41	-0.121	0.017	-0.016	0.014
**rs7523335**	A	G	0.173	-0.141	0.023	-0.016	0.018
**rs75565243**	A	G	0.029	0.349	0.055	0.007	0.042
**rs7797798**	G	C	0.416	-0.096	0.017	0.002	0.014
**rs9272514**	T	C	0.306	-0.235	0.021	0.005	0.015
**rs9370774**	C	T	0.201	-0.131	0.022	0.035	0.018
**rs9934775**	T	C	0.161	-0.14	0.023	0.03	0.019

Table S13. Raw data for the MR analysis of the causal effect of CD on ASD							
SNP	Effect allele	Other allele	Effect allele frequency	Beta.exposure	Se.exposure	Beta.outcome	Se.outcome
**rs10045431**	C	A	0.716	0.216	0.026	0.005	0.015
**rs10055349**	A	G	0.222	0.214	0.027	-0.012	0.017
**rs1056441**	C	T	0.698	0.167	0.026	0.01	0.015
**rs10748781**	A	C	0.551	-0.219	0.024	-0.001	0.014
**rs10761659**	G	A	0.553	0.212	0.024	-0.007	0.014
**rs11209026**	A	G	0.056	-0.995	0.064	-0.045	0.03
**rs11236797**	A	C	0.473	0.181	0.023	0.013	0.014
**rs112401990**	A	G	0.373	0.132	0.024	-0.015	0.014
**rs114607072**	T	G	0.04	0.442	0.063	0.026	0.038
**rs11564236**	T	A	0.034	0.519	0.06	0.019	0.053
**rs12194825**	A	T	0.186	-0.172	0.03	-0.008	0.018
**rs1250573**	A	G	0.287	-0.171	0.026	0.004	0.015
**rs12692254**	T	A	0.543	0.301	0.023	-0.007	0.014
**rs12717899**	T	G	0.794	0.159	0.029	0.02	0.017
**rs12936409**	T	C	0.471	0.138	0.023	0.014	0.014
**rs1297271**	T	C	0.43	-0.155	0.024	-0.001	0.014
**rs13135092**	G	A	0.095	0.221	0.039	0.061	0.029
**rs143345302**	T	C	0.134	0.212	0.036	0.01	0.022
**rs145126485**	C	A	0.036	0.627	0.062	0.001	0.048
**rs1456896**	T	C	0.698	0.139	0.025	-0.019	0.015
**rs147018773**	T	C	0.097	0.322	0.038	-0.034	0.027
**rs147684209**	C	T	0.369	0.155	0.024	0.003	0.014
**rs151314883**	A	G	0.158	-0.224	0.033	0.009	0.019
**rs1873625**	A	C	0.32	0.181	0.024	0.032	0.015
**rs1887428**	C	G	0.623	-0.168	0.024	0.002	0.015
**rs1932990**	T	C	0.254	0.153	0.026	0.036	0.017
**rs2076756**	G	A	0.284	0.4	0.024	0.043	0.016
**rs2129944**	G	T	0.291	-0.156	0.027	0	0.023
**rs2188962**	T	C	0.44	0.212	0.023	0.011	0.014
**rs2505640**	G	A	0.644	-0.146	0.024	-0.006	0.014
**rs281379**	A	G	0.489	0.14	0.024	0.016	0.014
**rs2856997**	A	C	0.421	0.141	0.023	0.002	0.014
**rs28701841**	A	G	0.117	0.224	0.037	0.01	0.023
**rs3024505**	A	G	0.162	0.178	0.03	0.032	0.018
**rs3091315**	G	A	0.266	-0.18	0.026	0.02	0.015
**rs3810936**	C	T	0.698	0.208	0.026	-0.01	0.015
**rs4077515**	T	C	0.42	0.216	0.024	-0.045	0.014
**rs444210**	G	A	0.547	0.163	0.023	-0.026	0.014
**rs4486887**	T	C	0.694	-0.196	0.025	0	0.016
**rs4820091**	G	T	0.198	0.172	0.028	-0.004	0.017
**rs4851586**	C	T	0.76	-0.169	0.026	-0.006	0.016
**rs4902642**	A	G	0.409	-0.129	0.024	0.015	0.014

**rs56062135**	T	C	0.234	0.193	0.027	-0.004	0.016
**rs56167332**	A	C	0.343	0.17	0.026	-0.001	0.016
**rs6588243**	C	A	0.59	0.132	0.023	-0.013	0.014
**rs6704109**	T	C	0.256	0.202	0.026	0.005	0.017
**rs6873866**	C	T	0.535	-0.168	0.024	-0.017	0.014
**rs697693**	A	G	0.201	0.172	0.028	0.011	0.018
**rs7276302**	G	A	0.608	-0.172	0.023	-0.003	0.014
**rs72798422**	C	T	0.048	0.59	0.051	0.062	0.039
**rs74179925**	T	A	0.205	-0.173	0.03	-0.011	0.017
**rs7423615**	T	C	0.194	0.164	0.029	-0.051	0.019
**rs744166**	G	A	0.408	-0.129	0.023	-0.016	0.014
**rs7499231**	G	A	0.124	-0.239	0.042	0.022	0.025
**rs7543234**	T	C	0.239	0.155	0.027	-0.014	0.016
**rs76532080**	T	C	0.068	0.358	0.052	-0.025	0.034
**rs7713270**	T	C	0.624	0.297	0.024	0.005	0.014
**rs78487399**	C	G	0.101	0.226	0.037	-0.003	0.024
**rs80262450**	A	G	0.113	0.283	0.035	-0.011	0.02
**rs8178977**	C	G	0.239	0.193	0.027	-0.014	0.017
**rs921720**	G	A	0.619	0.163	0.024	-0.01	0.014

Table S14. Raw data for the MR analysis of the causal effect of UC on ASD							
**SNP**	**Effect allele**	**Other allele**	**Effect allele frequency**	**Beta.exposure**	**Se.exposure**	**Beta.outcome**	**Se.outcome**
**rs10045431**	C	A	0.713	0.145	0.024	0.005	0.015
**rs10182512**	A	G	0.35	0.161	0.022	-0.015	0.014
**rs10272963**	T	C	0.426	-0.172	0.022	-0.017	0.014
**rs10737481**	G	T	0.556	0.25	0.022	0.026	0.014
**rs10917547**	T	A	0.362	-0.173	0.022	0.006	0.014
**rs11209026**	A	G	0.059	-0.562	0.052	-0.045	0.03
**rs114152040**	A	G	0.032	0.34	0.062	-0.02	0.038
**rs117292830**	A	G	0.027	0.615	0.07	0.045	0.044
**rs12612675**	G	A	0.403	0.123	0.022	-0.009	0.014
**rs12817473**	G	A	0.382	0.191	0.022	-0.009	0.014
**rs1317209**	A	G	0.188	0.146	0.027	0.036	0.018
**rs1359946**	A	G	0.196	0.158	0.027	0.023	0.018
**rs137845**	G	A	0.515	0.118	0.021	0.016	0.014
**rs143210366**	G	T	0.037	0.39	0.057	0.034	0.042
**rs144515162**	G	A	0.671	-0.232	0.027	-0.037	0.048
**rs1801274**	G	A	0.483	-0.183	0.022	-0.011	0.014
**rs183231933**	T	C	0.24	-0.371	0.031	-0.032	0.016
**rs1886731**	C	T	0.481	-0.141	0.022	-0.032	0.014
**rs1887428**	C	G	0.623	-0.177	0.022	0.002	0.015
**rs2212434**	T	C	0.46	0.142	0.021	0.012	0.014
**rs2301989**	A	G	0.402	-0.141	0.022	0.002	0.014
**rs254559**	A	C	0.404	0.124	0.022	0.004	0.014
**rs28383456**	T	C	0.335	-0.337	0.026	0.019	0.04
**rs3024493**	A	C	0.168	0.236	0.028	0.034	0.018
**rs34920465**	G	A	0.17	-0.193	0.029	-0.004	0.019
**rs35730213**	C	G	0.267	-0.167	0.025	-0.018	0.015
**rs3829111**	A	G	0.417	0.156	0.021	-0.044	0.014
**rs45627734**	A	G	0.03	0.406	0.063	0.008	0.042
**rs4574921**	T	C	0.741	0.151	0.026	-0.009	0.016
**rs4676410**	A	G	0.197	0.208	0.028	-0.031	0.02
**rs483905**	A	G	0.294	0.129	0.023	0.004	0.015
**rs484356**	G	C	0.328	-0.134	0.023	0.037	0.015
**rs56167332**	A	C	0.342	0.152	0.023	-0.001	0.016
**rs6017342**	C	A	0.538	0.191	0.024	0.012	0.014
**rs6062496**	A	G	0.573	0.158	0.022	0.024	0.015
**rs6933404**	C	T	0.216	0.167	0.025	0.007	0.017
**rs7282490**	A	G	0.604	-0.14	0.021	-0.001	0.014
**rs7523335**	A	G	0.177	-0.17	0.029	-0.016	0.018
**rs7752873**	T	C	0.137	0.182	0.03	0.022	0.021
**rs7911680**	C	A	0.49	-0.172	0.021	0.001	0.014
**rs798502**	C	A	0.283	-0.136	0.024	-0.029	0.015
**rs9272514**	T	C	0.298	-0.402	0.027	0.005	0.015

**rs9823546**	A	T	0.31	0.177	0.022	0.032	0.015
**rs9891174**	A	T	0.47	0.145	0.021	0.014	0.014
**rs9977672**	A	G	0.251	-0.245	0.026	-0.003	0.016

Table S15. Raw data for the MR analysis of the causal effect of IBD on BD							
SNP	Effect allele	Other allele	Effect allele frequency	Beta.exposure	Se.exposure	Beta.outcome	Se.outcome
**rs1003342**	G	A	0.531	-0.095	0.017	0.002	0.009
**rs10045431**	C	A	0.718	0.177	0.019	0.005	0.01
**rs10408351**	A	G	0.238	0.138	0.022	-0.003	0.012
**rs10737481**	G	T	0.555	0.141	0.017	0.01	0.009
**rs10761659**	G	A	0.554	0.162	0.017	-0.021	0.009
**rs10826797**	T	G	0.306	-0.102	0.019	0.011	0.011
**rs10917547**	T	A	0.362	-0.1	0.018	-0.018	0.01
**rs11209026**	A	G	0.054	-0.726	0.042	0.03	0.019
**rs11236797**	A	C	0.47	0.156	0.017	0.014	0.009
**rs112401990**	A	G	0.375	0.142	0.017	-0.026	0.01
**rs112874012**	T	C	0.045	-0.25	0.045	0.015	0.023
**rs113825849**	G	C	0.014	0.584	0.08	0.029	0.038
**rs11548656**	G	A	0.034	-0.293	0.051	0.004	0.027
**rs11677953**	A	G	0.415	0.098	0.017	0.008	0.01
**rs117292830**	A	G	0.027	0.43	0.058	-0.03	0.029
**rs11949375**	C	T	0.098	0.189	0.028	-0.015	0.021
**rs12446550**	A	G	0.41	0.108	0.017	0.016	0.01
**rs1250573**	A	G	0.296	-0.114	0.019	-0.009	0.01
**rs12764283**	A	G	0.337	0.127	0.018	0.016	0.01
**rs12936409**	T	C	0.476	0.146	0.017	-0.037	0.009
**rs140892874**	C	T	0.027	0.41	0.051	0.018	0.034
**rs142770866**	A	G	0.081	0.23	0.034	-0.019	0.018
**rs145126485**	C	A	0.033	0.283	0.05	-0.006	0.03
**rs148844907**	A	T	0.01	1.138	0.096	0.057	0.057
**rs1551399**	C	A	0.616	0.101	0.017	-0.005	0.01
**rs1736161**	A	G	0.429	-0.123	0.017	0.003	0.01
**rs1873625**	A	C	0.323	0.177	0.018	0.013	0.01
**rs1886731**	C	T	0.481	-0.097	0.018	0.028	0.01
**rs1887428**	C	G	0.619	-0.172	0.018	0.003	0.01
**rs2076756**	G	A	0.271	0.188	0.019	0.001	0.011
**rs2129944**	G	T	0.291	-0.119	0.02	-0.006	0.015
**rs2193041**	G	A	0.389	0.134	0.017	-0.014	0.01
**rs2230365**	T	C	0.163	0.154	0.023	0.005	0.013
**rs2241878**	C	T	0.538	0.148	0.017	0.002	0.009
**rs2542147**	T	G	0.837	-0.151	0.023	0.007	0.013
**rs254559**	A	C	0.402	0.103	0.017	-0.006	0.01
**rs2836882**	A	G	0.258	-0.196	0.02	0.002	0.011
**rs28383456**	T	C	0.345	-0.178	0.02	-0.016	0.014
**rs3024493**	A	C	0.17	0.213	0.022	-0.018	0.013
**rs3091316**	A	G	0.267	-0.112	0.019	-0.003	0.01
**rs34190331**	A	G	0.085	0.177	0.03	0.04	0.019
**rs34920465**	G	A	0.172	-0.139	0.023	-0.004	0.013

**rs35260072**	C	A	0.433	0.142	0.017	-0.006	0.01
**rs35730213**	C	G	0.263	-0.151	0.019	-0.034	0.011
**rs3850378**	C	T	0.096	0.155	0.028	0.002	0.016
**rs4077515**	T	C	0.424	0.179	0.017	-0.02	0.01
**rs4246905**	C	T	0.73	0.163	0.02	0.007	0.011
**rs444210**	G	A	0.546	0.11	0.017	0.002	0.009
**rs45528737**	T	C	0.112	0.167	0.03	0.029	0.016
**rs4676410**	A	G	0.195	0.155	0.023	0.013	0.012
**rs4712528**	C	G	0.788	0.123	0.021	-0.014	0.011
**rs4730272**	G	A	0.513	-0.134	0.018	-0.003	0.01
**rs4807569**	C	A	0.22	0.139	0.021	0.023	0.012
**rs56062135**	T	C	0.237	0.151	0.02	-0.012	0.011
**rs56167332**	A	C	0.346	0.156	0.019	-0.006	0.01
**rs6062496**	A	G	0.578	0.165	0.018	-0.024	0.01
**rs6584283**	C	T	0.511	-0.18	0.017	-0.004	0.009
**rs6826501**	T	C	0.536	-0.093	0.017	-0.023	0.009
**rs6880778**	G	A	0.621	0.188	0.017	-0.012	0.01
**rs6911490**	C	T	0.779	-0.143	0.021	-0.02	0.012
**rs6927172**	G	C	0.214	0.11	0.02	0.013	0.012
**rs72798422**	C	T	0.042	0.278	0.043	0.054	0.026
**rs7282490**	A	G	0.598	-0.145	0.017	-0.018	0.01
**rs7285952**	G	T	0.157	-0.176	0.024	0.004	0.013
**rs744166**	G	A	0.41	-0.121	0.017	-0.003	0.009
**rs7523335**	A	G	0.173	-0.141	0.023	0.002	0.012
**rs75565243**	A	G	0.029	0.349	0.055	0.038	0.055
**rs7797798**	G	C	0.416	-0.096	0.017	-0.01	0.009
**rs9370774**	C	T	0.201	-0.131	0.022	0.039	0.012
**rs9934775**	T	C	0.161	-0.14	0.023	0.003	0.013

Table S16. Raw data for the MR analysis of the causal effect of CD on BD							
**SNP**	**Effect allele**	**Other allele**	**Effect allele frequency**	**Beta.exposure**	**Se.exposure**	**Beta.outcome**	**Se.outcome**
**rs10045431**	C	A	0.716	0.216	0.026	0.005	0.01
**rs10055349**	A	G	0.222	0.214	0.027	-0.021	0.011
**rs1056441**	C	T	0.698	0.167	0.026	-0.021	0.01
**rs10748781**	A	C	0.551	-0.219	0.024	0.005	0.01
**rs10761659**	G	A	0.553	0.212	0.024	-0.021	0.009
**rs111564463**	G	A	0.021	0.563	0.093	-0.013	0.045
**rs11209026**	A	G	0.056	-0.995	0.064	0.03	0.019
**rs11236797**	A	C	0.473	0.181	0.023	0.014	0.009
**rs112401990**	A	G	0.373	0.132	0.024	-0.026	0.01
**rs114607072**	T	G	0.04	0.442	0.063	0.058	0.025
**rs11564236**	T	A	0.034	0.519	0.06	0.018	0.034
**rs12194825**	A	T	0.186	-0.172	0.03	0.011	0.012
**rs1250573**	A	G	0.287	-0.171	0.026	-0.009	0.01
**rs12692254**	T	A	0.543	0.301	0.023	0.002	0.009
**rs12717899**	T	G	0.794	0.159	0.029	0.016	0.011
**rs12936409**	T	C	0.471	0.138	0.023	-0.037	0.009
**rs1297271**	T	C	0.43	-0.155	0.024	0.005	0.01
**rs13135092**	G	A	0.095	0.221	0.039	0.044	0.018
**rs143345302**	T	C	0.134	0.212	0.036	0	0.014
**rs145126485**	C	A	0.036	0.627	0.062	-0.006	0.03
**rs1456896**	T	C	0.698	0.139	0.025	0.002	0.01
**rs148844907**	A	T	0.008	0.958	0.142	0.057	0.057
**rs151314883**	A	G	0.158	-0.224	0.033	0.003	0.013
**rs1873625**	A	C	0.32	0.181	0.024	0.013	0.01
**rs1887428**	C	G	0.623	-0.168	0.024	0.003	0.01
**rs1932990**	T	C	0.254	0.153	0.026	0.026	0.011
**rs2076756**	G	A	0.284	0.4	0.024	0.001	0.011
**rs2129944**	G	T	0.291	-0.156	0.027	-0.006	0.015
**rs2188962**	T	C	0.44	0.212	0.023	-0.005	0.01
**rs2505640**	G	A	0.644	-0.146	0.024	-0.018	0.01
**rs281379**	A	G	0.489	0.14	0.024	0.017	0.01
**rs2856997**	A	C	0.421	0.141	0.023	0.011	0.01
**rs28701841**	A	G	0.117	0.224	0.037	0.025	0.016
**rs3024505**	A	G	0.162	0.178	0.03	-0.018	0.013
**rs3091315**	G	A	0.266	-0.18	0.026	-0.004	0.01
**rs3810936**	C	T	0.698	0.208	0.026	0.008	0.01
**rs4077515**	T	C	0.42	0.216	0.024	-0.02	0.01
**rs444210**	G	A	0.547	0.163	0.023	0.002	0.009
**rs4486887**	T	C	0.694	-0.196	0.025	-0.011	0.011
**rs4820091**	G	T	0.198	0.172	0.028	-0.002	0.012
**rs4851586**	C	T	0.76	-0.169	0.026	-0.005	0.011
**rs4902642**	A	G	0.409	-0.129	0.024	-0.01	0.01

**rs56062135**	T	C	0.234	0.193	0.027	-0.012	0.011
**rs56167332**	A	C	0.343	0.17	0.026	-0.006	0.01
**rs6588243**	C	A	0.59	0.132	0.023	0.017	0.009
**rs6704109**	T	C	0.256	0.202	0.026	-0.002	0.011
**rs697693**	A	G	0.201	0.172	0.028	-0.009	0.012
**rs7276302**	G	A	0.608	-0.172	0.023	-0.016	0.01
**rs72798422**	C	T	0.048	0.59	0.051	0.054	0.026
**rs74179925**	T	A	0.205	-0.173	0.03	0.02	0.011
**rs7423615**	T	C	0.194	0.164	0.029	-0.007	0.012
**rs744166**	G	A	0.408	-0.129	0.023	-0.003	0.009
**rs7499231**	G	A	0.124	-0.239	0.042	-0.034	0.016
**rs76532080**	T	C	0.068	0.358	0.052	0.005	0.022
**rs7713270**	T	C	0.624	0.297	0.024	-0.013	0.01
**rs78487399**	C	G	0.101	0.226	0.037	-0.044	0.016
**rs80262450**	A	G	0.113	0.283	0.035	-0.024	0.014
**rs8178977**	C	G	0.239	0.193	0.027	0.023	0.011
**rs921720**	G	A	0.619	0.163	0.024	-0.006	0.01

Table S17. Raw data for the MR analysis of the causal effect of UC on BD							
**SNP**	**Effect allele**	**Other allele**	**Effect allele frequency**	**Beta.exposure**	**Se.exposure**	**Beta.outcome**	**Se.outcome**
**rs10045431**	C	A	0.713	0.145	0.024	0.005	0.01
**rs10182512**	A	G	0.35	0.161	0.022	-0.023	0.01
**rs10272963**	T	C	0.426	-0.172	0.022	-0.009	0.009
**rs10737481**	G	T	0.556	0.25	0.022	0.01	0.009
**rs10917547**	T	A	0.362	-0.173	0.022	-0.018	0.01
**rs11209026**	A	G	0.059	-0.562	0.052	0.03	0.019
**rs114152040**	A	G	0.032	0.34	0.062	0.013	0.026
**rs117292830**	A	G	0.027	0.615	0.07	-0.03	0.029
**rs12612675**	G	A	0.403	0.123	0.022	0.008	0.01
**rs12817473**	G	A	0.382	0.191	0.022	-0.016	0.01
**rs1317209**	A	G	0.188	0.146	0.027	0.009	0.013
**rs1359946**	A	G	0.196	0.158	0.027	-0.007	0.012
**rs137845**	G	A	0.515	0.118	0.021	0.024	0.01
**rs148844907**	A	T	0.01	1.341	0.109	0.057	0.057
**rs1801274**	G	A	0.483	-0.183	0.022	-0.005	0.009
**rs1886731**	C	T	0.481	-0.141	0.022	0.028	0.01
**rs1887428**	C	G	0.623	-0.177	0.022	0.003	0.01
**rs2212434**	T	C	0.46	0.142	0.021	0.013	0.009
**rs2301989**	A	G	0.402	-0.141	0.022	-0.011	0.009
**rs254559**	A	C	0.404	0.124	0.022	-0.006	0.01
**rs28383456**	T	C	0.335	-0.337	0.026	-0.016	0.014
**rs3024493**	A	C	0.168	0.236	0.028	-0.018	0.013
**rs34920465**	G	A	0.17	-0.193	0.029	-0.004	0.013
**rs35730213**	C	G	0.267	-0.167	0.025	-0.034	0.011
**rs3829111**	A	G	0.417	0.156	0.021	-0.02	0.01
**rs45627734**	A	G	0.03	0.406	0.063	0.033	0.029
**rs4574921**	T	C	0.741	0.151	0.026	0.01	0.011
**rs4676410**	A	G	0.197	0.208	0.028	0.013	0.012
**rs483905**	A	G	0.294	0.129	0.023	0.012	0.01
**rs56167332**	A	C	0.342	0.152	0.023	-0.006	0.01
**rs6017342**	C	A	0.538	0.191	0.024	-0.011	0.01
**rs6062496**	A	G	0.573	0.158	0.022	-0.024	0.01
**rs6933404**	C	T	0.216	0.167	0.025	0.014	0.012
**rs7282490**	A	G	0.604	-0.14	0.021	-0.018	0.01
**rs7523335**	A	G	0.177	-0.17	0.029	0.002	0.012
**rs7752873**	T	C	0.137	0.182	0.03	0.012	0.014
**rs7911680**	C	A	0.49	-0.172	0.021	0	0.009
**rs798502**	C	A	0.283	-0.136	0.024	0.011	0.01
**rs9823546**	A	T	0.31	0.177	0.022	0.012	0.01
**rs9891174**	A	T	0.47	0.145	0.021	-0.038	0.01
**rs9977672**	A	G	0.251	-0.245	0.026	0.003	0.011

Table S18. Raw data for the MR analysis of the causal effect of IBD on AN							
SNP	Effect allele	Other allele	Effect allele frequency	Beta.exposure	Se.exposure	Beta.outcome	Se.outcome
**rs1003342**	G	A	0.531	-0.095	0.017	-0.02	0.014
**rs10045431**	C	A	0.718	0.177	0.019	0.008	0.015
**rs10175585**	A	G	0.713	-0.133	0.02	0.011	0.016
**rs10408351**	A	G	0.238	0.138	0.022	0.024	0.019
**rs10737481**	G	T	0.555	0.141	0.017	0.003	0.013
**rs10761659**	G	A	0.554	0.162	0.017	-0.012	0.014
**rs10800314**	A	C	0.649	-0.143	0.018	-0.01	0.014
**rs10826797**	T	G	0.306	-0.102	0.019	0.003	0.016
**rs10917547**	T	A	0.362	-0.1	0.018	0.007	0.014
**rs11209026**	A	G	0.054	-0.726	0.042	0.004	0.028
**rs11236797**	A	C	0.47	0.156	0.017	-0.002	0.014
**rs112401990**	A	G	0.375	0.142	0.017	0.004	0.014
**rs112694524**	A	G	0.09	0.188	0.03	0.059	0.029
**rs112874012**	T	C	0.045	-0.25	0.045	0.014	0.035
**rs113825849**	G	C	0.014	0.584	0.08	-0.031	0.057
**rs11548656**	G	A	0.034	-0.293	0.051	0.053	0.039
**rs11677953**	A	G	0.415	0.098	0.017	-0.001	0.014
**rs117292830**	A	G	0.027	0.43	0.058	0.05	0.042
**rs11949375**	C	T	0.098	0.189	0.028	-0.021	0.025
**rs12446550**	A	G	0.41	0.108	0.017	-0.029	0.014
**rs1250573**	A	G	0.296	-0.114	0.019	-0.003	0.015
**rs12764283**	A	G	0.337	0.127	0.018	0.007	0.014
**rs12936409**	T	C	0.476	0.146	0.017	0.017	0.013
**rs131657**	A	T	0.202	0.136	0.021	-0.004	0.018
**rs140892874**	C	T	0.027	0.41	0.051	-0.091	0.05
**rs142770866**	A	G	0.081	0.23	0.034	-0.069	0.027
**rs145126485**	C	A	0.033	0.283	0.05	0.034	0.051
**rs1551399**	C	A	0.616	0.101	0.017	0.021	0.014
**rs1736161**	A	G	0.429	-0.123	0.017	0.011	0.014
**rs1873625**	A	C	0.323	0.177	0.018	0.069	0.015
**rs1886731**	C	T	0.481	-0.097	0.018	-0.019	0.016
**rs1887428**	C	G	0.619	-0.172	0.018	-0.003	0.014
**rs2076756**	G	A	0.271	0.188	0.019	0.03	0.016
**rs2129944**	G	T	0.291	-0.119	0.02	-0.001	0.024
**rs2193041**	G	A	0.389	0.134	0.017	-0.01	0.014
**rs2230365**	T	C	0.163	0.154	0.023	-0.005	0.019
**rs2241878**	C	T	0.538	0.148	0.017	0.004	0.013
**rs2542147**	T	G	0.837	-0.151	0.023	-0.007	0.018
**rs254559**	A	C	0.402	0.103	0.017	0.019	0.014
**rs2836882**	A	G	0.258	-0.196	0.02	0.003	0.015
**rs3024493**	A	C	0.17	0.213	0.022	-0.011	0.018
**rs3091316**	A	G	0.267	-0.112	0.019	0.021	0.015

**rs34190331**	A	G	0.085	0.177	0.03	-0.012	0.028
**rs34920465**	G	A	0.172	-0.139	0.023	0.008	0.018
**rs35260072**	C	A	0.433	0.142	0.017	0.002	0.014
**rs35730213**	C	G	0.263	-0.151	0.019	-0.039	0.015
**rs3850378**	C	T	0.096	0.155	0.028	0.002	0.023
**rs4077515**	T	C	0.424	0.179	0.017	-0.022	0.015
**rs4246905**	C	T	0.73	0.163	0.02	0.018	0.015
**rs444210**	G	A	0.546	0.11	0.017	-0.017	0.014
**rs45528737**	T	C	0.112	0.167	0.03	-0.038	0.028
**rs4676410**	A	G	0.195	0.155	0.023	0.019	0.021
**rs4712528**	C	G	0.788	0.123	0.021	-0.001	0.016
**rs4730272**	G	A	0.513	-0.134	0.018	0.006	0.014
**rs4807569**	C	A	0.22	0.139	0.021	0.017	0.018
**rs56062135**	T	C	0.237	0.151	0.02	-0.001	0.016
**rs56167332**	A	C	0.346	0.156	0.019	-0.003	0.016
**rs6062496**	A	G	0.578	0.165	0.018	0.002	0.015
**rs6584283**	C	T	0.511	-0.18	0.017	-0.015	0.014
**rs6826501**	T	C	0.536	-0.093	0.017	0.006	0.014
**rs6873866**	C	T	0.529	-0.107	0.018	0.012	0.014
**rs6880778**	G	A	0.621	0.188	0.017	0.011	0.014
**rs6911490**	C	T	0.779	-0.143	0.021	-0.024	0.016
**rs6927172**	G	C	0.214	0.11	0.02	-0.001	0.016
**rs72798422**	C	T	0.042	0.278	0.043	0.051	0.039
**rs7282490**	A	G	0.598	-0.145	0.017	0.008	0.014
**rs7285952**	G	T	0.157	-0.176	0.024	0.013	0.019
**rs744166**	G	A	0.41	-0.121	0.017	0.02	0.014
**rs7523335**	A	G	0.173	-0.141	0.023	0.008	0.018
**rs75565243**	A	G	0.029	0.349	0.055	0.072	0.042
**rs7797798**	G	C	0.416	-0.096	0.017	0.014	0.014
**rs9370774**	C	T	0.201	-0.131	0.022	0.022	0.018
**rs9934775**	T	C	0.161	-0.14	0.023	-0.016	0.019

Table S19. Raw data for the MR analysis of the causal effect of CD on AN							
SNP	Effect allele	Other allele	Effect allele frequency	Beta.exposure	Se.exposure	Beta.outcome	Se.outcome
**rs10045431**	C	A	0.716	0.216	0.026	0.008	0.015
**rs10055349**	A	G	0.222	0.214	0.027	-0.022	0.017
**rs1056441**	C	T	0.698	0.167	0.026	-0.006	0.015
**rs10748781**	A	C	0.551	-0.219	0.024	-0.008	0.014
**rs10761659**	G	A	0.553	0.212	0.024	-0.012	0.014
**rs11209026**	A	G	0.056	-0.995	0.064	0.004	0.028
**rs11236797**	A	C	0.473	0.181	0.023	-0.002	0.014
**rs112401990**	A	G	0.373	0.132	0.024	0.004	0.014
**rs114607072**	T	G	0.04	0.442	0.063	0.064	0.037
**rs11564236**	T	A	0.034	0.519	0.06	-0.09	0.05
**rs12194825**	A	T	0.186	-0.172	0.03	0.026	0.017
**rs1250573**	A	G	0.287	-0.171	0.026	-0.003	0.015
**rs12692254**	T	A	0.543	0.301	0.023	0.005	0.014
**rs12717899**	T	G	0.794	0.159	0.029	-0.004	0.016
**rs12936409**	T	C	0.471	0.138	0.023	0.017	0.013
**rs1297271**	T	C	0.43	-0.155	0.024	0.012	0.014
**rs13135092**	G	A	0.095	0.221	0.039	-0.003	0.028
**rs143345302**	T	C	0.134	0.212	0.036	0.003	0.021
**rs145126485**	C	A	0.036	0.627	0.062	0.034	0.051
**rs1456896**	T	C	0.698	0.139	0.025	-0.006	0.014
**rs147018773**	T	C	0.097	0.322	0.038	-0.016	0.025
**rs147684209**	C	T	0.369	0.155	0.024	-0.022	0.014
**rs151314883**	A	G	0.158	-0.224	0.033	0.013	0.019
**rs1873625**	A	C	0.32	0.181	0.024	0.069	0.015
**rs1887428**	C	G	0.623	-0.168	0.024	-0.003	0.014
**rs1932990**	T	C	0.254	0.153	0.026	0.031	0.016
**rs2076756**	G	A	0.284	0.4	0.024	0.03	0.016
**rs2129944**	G	T	0.291	-0.156	0.027	-0.001	0.024
**rs2188962**	T	C	0.44	0.212	0.023	0.008	0.014
**rs2505640**	G	A	0.644	-0.146	0.024	-0.006	0.014
**rs281379**	A	G	0.489	0.14	0.024	-0.01	0.014
**rs2856997**	A	C	0.421	0.141	0.023	-0.007	0.014
**rs28701841**	A	G	0.117	0.224	0.037	0.034	0.023
**rs3024505**	A	G	0.162	0.178	0.03	-0.011	0.018
**rs3091315**	G	A	0.266	-0.18	0.026	0.02	0.015
**rs3810936**	C	T	0.698	0.208	0.026	0.02	0.015
**rs4077515**	T	C	0.42	0.216	0.024	-0.022	0.015
**rs444210**	G	A	0.547	0.163	0.023	-0.017	0.014
**rs4486887**	T	C	0.694	-0.196	0.025	-0.01	0.015
**rs4820091**	G	T	0.198	0.172	0.028	0	0.017
**rs4851586**	C	T	0.76	-0.169	0.026	0.011	0.016
**rs4902642**	A	G	0.409	-0.129	0.024	-0.001	0.014

**rs56062135**	T	C	0.234	0.193	0.027	-0.001	0.016
**rs56167332**	A	C	0.343	0.17	0.026	-0.003	0.016
**rs6588243**	C	A	0.59	0.132	0.023	-0.006	0.014
**rs6704109**	T	C	0.256	0.202	0.026	0.005	0.016
**rs6873866**	C	T	0.535	-0.168	0.024	0.012	0.014
**rs697693**	A	G	0.201	0.172	0.028	-0.023	0.017
**rs7276302**	G	A	0.608	-0.172	0.023	0.009	0.014
**rs72798422**	C	T	0.048	0.59	0.051	0.051	0.039
**rs74179925**	T	A	0.205	-0.173	0.03	0.014	0.017
**rs7423615**	T	C	0.194	0.164	0.029	0.034	0.017
**rs744166**	G	A	0.408	-0.129	0.023	0.02	0.014
**rs7499231**	G	A	0.124	-0.239	0.042	-0.068	0.029
**rs7543234**	T	C	0.239	0.155	0.027	-0.017	0.015
**rs7713270**	T	C	0.624	0.297	0.024	0.011	0.014
**rs78487399**	C	G	0.101	0.226	0.037	0.022	0.023
**rs80262450**	A	G	0.113	0.283	0.035	0.007	0.02
**rs8178977**	C	G	0.239	0.193	0.027	0.006	0.018
**rs921720**	G	A	0.619	0.163	0.024	0.021	0.014

Table S20. Raw data for the MR analysis of the causal effect of UC on AN							
SNP	Effect allele	Other allele	Effect allele frequency	Beta.exposure	Se.exposure	Beta.outcome	Se.outcome
**rs10045431**	C	A	0.713	0.145	0.024	0.008	0.015
**rs10182512**	A	G	0.35	0.161	0.022	0.003	0.014
**rs10272963**	T	C	0.426	-0.172	0.022	0.016	0.014
**rs10737481**	G	T	0.556	0.25	0.022	0.003	0.013
**rs10917547**	T	A	0.362	-0.173	0.022	0.007	0.014
**rs11209026**	A	G	0.059	-0.562	0.052	0.004	0.028
**rs114152040**	A	G	0.032	0.34	0.062	-0.024	0.039
**rs117292830**	A	G	0.027	0.615	0.07	0.05	0.042
**rs12612675**	G	A	0.403	0.123	0.022	-0.002	0.014
**rs12817473**	G	A	0.382	0.191	0.022	-0.009	0.014
**rs1317209**	A	G	0.188	0.146	0.027	0.008	0.017
**rs1359946**	A	G	0.196	0.158	0.027	0.011	0.018
**rs137845**	G	A	0.515	0.118	0.021	-0.001	0.015
**rs143210366**	G	T	0.037	0.39	0.057	0.003	0.042
**rs1801274**	G	A	0.483	-0.183	0.022	0.004	0.014
**rs1886731**	C	T	0.481	-0.141	0.022	-0.019	0.016
**rs1887428**	C	G	0.623	-0.177	0.022	-0.003	0.014
**rs2212434**	T	C	0.46	0.142	0.021	0	0.014
**rs2301989**	A	G	0.402	-0.141	0.022	0.015	0.014
**rs254559**	A	C	0.404	0.124	0.022	0.019	0.014
**rs3024493**	A	C	0.168	0.236	0.028	-0.011	0.018
**rs34920465**	G	A	0.17	-0.193	0.029	0.008	0.018
**rs35730213**	C	G	0.267	-0.167	0.025	-0.039	0.015
**rs3829111**	A	G	0.417	0.156	0.021	-0.024	0.015
**rs45627734**	A	G	0.03	0.406	0.063	0.073	0.042
**rs4574921**	T	C	0.741	0.151	0.026	0.008	0.016
**rs4676410**	A	G	0.197	0.208	0.028	0.019	0.021
**rs483905**	A	G	0.294	0.129	0.023	0.009	0.015
**rs484356**	G	C	0.328	-0.134	0.023	0.006	0.015
**rs56167332**	A	C	0.342	0.152	0.023	-0.003	0.016
**rs6017342**	C	A	0.538	0.191	0.024	0.008	0.015
**rs6062496**	A	G	0.573	0.158	0.022	0.002	0.015
**rs6933404**	C	T	0.216	0.167	0.025	-0.002	0.017
**rs7282490**	A	G	0.604	-0.14	0.021	0.008	0.014
**rs7523335**	A	G	0.177	-0.17	0.029	0.008	0.018
**rs7752873**	T	C	0.137	0.182	0.03	0.034	0.02
**rs7911680**	C	A	0.49	-0.172	0.021	-0.011	0.014
**rs798502**	C	A	0.283	-0.136	0.024	-0.023	0.015
**rs9823546**	A	T	0.31	0.177	0.022	0.071	0.015
**rs9891174**	A	T	0.47	0.145	0.021	0.017	0.014
**rs9977672**	A	G	0.251	-0.245	0.026	0.004	0.015

Table S21. Raw data for the MR analysis of the causal effect of IBD on MDD							
**SNP**	**Effect allele**	**Other allele**	**Effect allele frequency**	**Beta.exposure**	**Se.exposure**	**Beta.outcome**	**Se.outcome**
**rs1003342**	G	A	0.531	-0.095	0.017	0.003	0.008
**rs10045431**	C	A	0.718	0.177	0.019	0.003	0.009
**rs10761659**	G	A	0.554	0.162	0.017	-0.01	0.008
**rs10800314**	A	C	0.649	-0.143	0.018	-0.002	0.009
**rs10826797**	T	G	0.306	-0.102	0.019	0.002	0.009
**rs11209026**	A	G	0.054	-0.726	0.042	0.014	0.016
**rs11236797**	A	C	0.47	0.156	0.017	0.008	0.008
**rs112694524**	A	G	0.09	0.188	0.03	-0.011	0.016
**rs112874012**	T	C	0.045	-0.25	0.045	0	0.019
**rs113825849**	G	C	0.014	0.584	0.08	-0.028	0.035
**rs115312361**	C	A	0.331	-0.184	0.023	-0.014	0.01
**rs11548656**	G	A	0.034	-0.293	0.051	0.049	0.022
**rs116465569**	C	T	0.61	-0.201	0.021	-0.006	0.01
**rs11677953**	A	G	0.415	0.098	0.017	-0.011	0.008
**rs117292830**	A	G	0.027	0.43	0.058	0.025	0.025
**rs11949375**	C	T	0.098	0.189	0.028	0.008	0.017
**rs12446550**	A	G	0.41	0.108	0.017	-0.006	0.008
**rs12764283**	A	G	0.337	0.127	0.018	0.003	0.008
**rs131657**	A	T	0.202	0.136	0.021	-0.024	0.01
**rs140892874**	C	T	0.027	0.41	0.051	-0.03	0.033
**rs142770866**	A	G	0.081	0.23	0.034	-0.017	0.016
**rs144515162**	G	A	0.672	-0.15	0.022	0.011	0.011
**rs145126485**	C	A	0.033	0.283	0.05	-0.019	0.026
**rs148844907**	A	T	0.01	1.138	0.096	0.054	0.073
**rs1736161**	A	G	0.429	-0.123	0.017	0.007	0.008
**rs1873625**	A	C	0.323	0.177	0.018	-0.022	0.009
**rs1886731**	C	T	0.481	-0.097	0.018	-0.017	0.008
**rs1887428**	C	G	0.619	-0.172	0.018	0.007	0.008
**rs2076756**	G	A	0.271	0.188	0.019	-0.001	0.009
**rs2129944**	G	T	0.291	-0.119	0.02	-0.005	0.011
**rs2230365**	T	C	0.163	0.154	0.023	-0.01	0.011
**rs2241878**	C	T	0.538	0.148	0.017	-0.001	0.008
**rs254559**	A	C	0.402	0.103	0.017	0.011	0.008
**rs2836882**	A	G	0.258	-0.196	0.02	0.008	0.009
**rs28383456**	T	C	0.345	-0.178	0.02	0.013	0.01
**rs3024493**	A	C	0.17	0.213	0.022	0.005	0.011
**rs34190331**	A	G	0.085	0.177	0.03	0	0.016
**rs35260072**	C	A	0.433	0.142	0.017	0.008	0.008
**rs35730213**	C	G	0.263	-0.151	0.019	-0.012	0.009
**rs3850378**	C	T	0.096	0.155	0.028	0.002	0.014
**rs4077515**	T	C	0.424	0.179	0.017	-0.013	0.008
**rs4246905**	C	T	0.73	0.163	0.02	0.006	0.009

**rs444210**	G	A	0.546	0.11	0.017	0.004	0.008
**rs4712528**	C	G	0.788	0.123	0.021	0.002	0.01
**rs4730272**	G	A	0.513	-0.134	0.018	-0.004	0.008
**rs4807569**	C	A	0.22	0.139	0.021	0	0.011
**rs56062135**	T	C	0.237	0.151	0.02	-0.006	0.009
**rs56167332**	A	C	0.346	0.156	0.019	-0.011	0.009
**rs6584283**	C	T	0.511	-0.18	0.017	-0.007	0.008
**rs6826501**	T	C	0.536	-0.093	0.017	0.009	0.008
**rs6880778**	G	A	0.621	0.188	0.017	0.003	0.008
**rs6911490**	C	T	0.779	-0.143	0.021	0	0.01
**rs6927172**	G	C	0.214	0.11	0.02	0.014	0.01
**rs72798422**	C	T	0.042	0.278	0.043	-0.004	0.022
**rs7282490**	A	G	0.598	-0.145	0.017	-0.001	0.009
**rs7285952**	G	T	0.157	-0.176	0.024	0.004	0.011
**rs744166**	G	A	0.41	-0.121	0.017	-0.007	0.008
**rs7523335**	A	G	0.173	-0.141	0.023	-0.021	0.01
**rs75565243**	A	G	0.029	0.349	0.055	0.067	0.029
**rs7797798**	G	C	0.416	-0.096	0.017	-0.006	0.008
**rs9272514**	T	C	0.306	-0.235	0.021	0.008	0.01
**rs9934775**	T	C	0.161	-0.14	0.023	0.003	0.011

Table S22. Raw data for the MR analysis of the causal effect of CD on MDD							
SNP	Effect allele	Other allele	Effect allele frequency	Beta.exposure	Se.exposure	Beta.outcome	Se.outcome
**rs10045431**	C	A	0.716	0.216	0.026	0.003	0.009
**rs10055349**	A	G	0.222	0.214	0.027	-0.01	0.01
**rs10748781**	A	C	0.551	-0.219	0.024	-0.007	0.008
**rs10761659**	G	A	0.553	0.212	0.024	-0.01	0.008
**rs111564463**	G	A	0.021	0.563	0.093	0.044	0.042
**rs11209026**	A	G	0.056	-0.995	0.064	0.014	0.016
**rs11236797**	A	C	0.473	0.181	0.023	0.008	0.008
**rs114607072**	T	G	0.04	0.442	0.063	0.052	0.021
**rs11564236**	T	A	0.034	0.519	0.06	-0.013	0.029
**rs12194825**	A	T	0.186	-0.172	0.03	0.005	0.01
**rs12692254**	T	A	0.543	0.301	0.023	-0.001	0.008
**rs12717899**	T	G	0.794	0.159	0.029	0.019	0.01
**rs1297271**	T	C	0.43	-0.155	0.024	0.005	0.008
**rs13135092**	G	A	0.095	0.221	0.039	-0.017	0.016
**rs143345302**	T	C	0.134	0.212	0.036	0.035	0.012
**rs145126485**	C	A	0.036	0.627	0.062	-0.019	0.026
**rs1456896**	T	C	0.698	0.139	0.025	0.009	0.009
**rs147018773**	T	C	0.097	0.322	0.038	0.015	0.017
**rs147684209**	C	T	0.369	0.155	0.024	-0.002	0.008
**rs148844907**	A	T	0.008	0.958	0.142	0.054	0.073
**rs151314883**	A	G	0.158	-0.224	0.033	0.003	0.011
**rs1873625**	A	C	0.32	0.181	0.024	-0.022	0.009
**rs1887428**	C	G	0.623	-0.168	0.024	0.007	0.008
**rs1932990**	T	C	0.254	0.153	0.026	0.014	0.01
**rs2076756**	G	A	0.284	0.4	0.024	-0.001	0.009
**rs2129944**	G	T	0.291	-0.156	0.027	-0.005	0.011
**rs2188962**	T	C	0.44	0.212	0.023	0.006	0.008
**rs2505640**	G	A	0.644	-0.146	0.024	-0.01	0.008
**rs2856997**	A	C	0.421	0.141	0.023	0.019	0.008
**rs28701841**	A	G	0.117	0.224	0.037	-0.004	0.014
**rs3024505**	A	G	0.162	0.178	0.03	0.005	0.011
**rs3810936**	C	T	0.698	0.208	0.026	0.003	0.009
**rs4077515**	T	C	0.42	0.216	0.024	-0.013	0.008
**rs444210**	G	A	0.547	0.163	0.023	0.004	0.008
**rs4486887**	T	C	0.694	-0.196	0.025	0.004	0.009
**rs4820091**	G	T	0.198	0.172	0.028	-0.017	0.01
**rs4902642**	A	G	0.409	-0.129	0.024	0.008	0.008
**rs56062135**	T	C	0.234	0.193	0.027	-0.006	0.009
**rs56167332**	A	C	0.343	0.17	0.026	-0.011	0.009
**rs6588243**	C	A	0.59	0.132	0.023	-0.002	0.008
**rs6704109**	T	C	0.256	0.202	0.026	-0.011	0.009
**rs697693**	A	G	0.201	0.172	0.028	-0.003	0.01

**rs7276302**	G	A	0.608	-0.172	0.023	-0.003	0.009
**rs72798422**	C	T	0.048	0.59	0.051	-0.004	0.022
**rs74179925**	T	A	0.205	-0.173	0.03	0.009	0.01
**rs7423615**	T	C	0.194	0.164	0.029	0.005	0.01
**rs744166**	G	A	0.408	-0.129	0.023	-0.007	0.008
**rs7499231**	G	A	0.124	-0.239	0.042	0.002	0.016
**rs7543234**	T	C	0.239	0.155	0.027	0.008	0.009
**rs76532080**	T	C	0.068	0.358	0.052	-0.044	0.019
**rs7713270**	T	C	0.624	0.297	0.024	0	0.008
**rs78487399**	C	G	0.101	0.226	0.037	-0.021	0.013
**rs8178977**	C	G	0.239	0.193	0.027	0.007	0.011

Table S23. Raw data for the MR analysis of the causal effect of UC on MDD							
SNP	Effect allele	Other allele	Effect allele frequency	Beta.exposure	Se.exposure	Beta.outcome	Se.outcome
rs10045431	C	A	0.713	0.145	0.024	0.003	0.009
rs10272963	T	C	0.426	-0.172	0.022	-0.005	0.008
rs11209026	A	G	0.059	-0.562	0.052	0.014	0.016
rs114152040	A	G	0.032	0.34	0.062	-0.007	0.023
rs115312361	C	A	0.329	-0.324	0.029	-0.014	0.01
rs117292830	A	G	0.027	0.615	0.07	0.025	0.025
rs12612675	G	A	0.403	0.123	0.022	-0.01	0.008
rs143210366	G	T	0.037	0.39	0.057	0.016	0.028
rs144515162	G	A	0.671	-0.232	0.027	0.011	0.011
rs144582178	C	T	0.672	-0.276	0.028	0.005	0.011
rs148844907	A	T	0.01	1.341	0.109	0.054	0.073
rs1801274	G	A	0.483	-0.183	0.022	-0.017	0.008
rs183231933	T	C	0.24	-0.371	0.031	0.004	0.012
rs1886731	C	T	0.481	-0.141	0.022	-0.017	0.008
rs1887428	C	G	0.623	-0.177	0.022	0.007	0.008
rs2212434	T	C	0.46	0.142	0.021	0.006	0.008
rs2301989	A	G	0.402	-0.141	0.022	-0.006	0.008
rs254559	A	C	0.404	0.124	0.022	0.011	0.008
rs28383456	T	C	0.335	-0.337	0.026	0.013	0.01
rs3024493	A	C	0.168	0.236	0.028	0.005	0.011
rs35730213	C	G	0.267	-0.167	0.025	-0.012	0.009
rs3829111	A	G	0.417	0.156	0.021	-0.013	0.008
rs45627734	A	G	0.03	0.406	0.063	0.057	0.027
rs4574921	T	C	0.741	0.151	0.026	0.012	0.009
rs483905	A	G	0.294	0.129	0.023	0	0.009
rs484356	G	C	0.328	-0.134	0.023	-0.006	0.009
rs56167332	A	C	0.342	0.152	0.023	-0.011	0.009
rs6933404	C	T	0.216	0.167	0.025	0.014	0.01
rs7282490	A	G	0.604	-0.14	0.021	-0.001	0.009
rs7523335	A	G	0.177	-0.17	0.029	-0.021	0.01
rs7752873	T	C	0.137	0.182	0.03	0.004	0.012
rs7911680	C	A	0.49	-0.172	0.021	-0.004	0.008
rs798502	C	A	0.283	-0.136	0.024	-0.006	0.009
rs9272514	T	C	0.298	-0.402	0.027	0.008	0.01
rs9823546	A	T	0.31	0.177	0.022	-0.022	0.009
rs9977672	A	G	0.251	-0.245	0.026	0.009	0.009

Table S24. Raw data for the MR analysis of the causal effect of IBD on OCD							
**SNP**	**Effect allele**	**Other allele**	**Effect allele frequency**	**Beta.exposure**	**Se.exposure**	**Beta.outcome**	**Se.outcome**
**rs1003342**	G	A	0.531	-0.095	0.017	-0.015	0.034
**rs10045431**	C	A	0.718	0.177	0.019	-0.071	0.036
**rs10175585**	A	G	0.713	-0.133	0.02	-0.005	0.051
**rs10408351**	A	G	0.238	0.138	0.022	0.042	0.042
**rs10737481**	G	T	0.555	0.141	0.017	-0.001	0.034
**rs10761659**	G	A	0.554	0.162	0.017	0.049	0.035
**rs10800314**	A	C	0.649	-0.143	0.018	0.012	0.036
**rs10826797**	T	G	0.306	-0.102	0.019	-0.075	0.037
**rs10917547**	T	A	0.362	-0.1	0.018	0.002	0.035
**rs11209026**	A	G	0.054	-0.726	0.042	-0.052	0.068
**rs11236797**	A	C	0.47	0.156	0.017	-0.045	0.034
**rs112401990**	A	G	0.375	0.142	0.017	-0.027	0.035
**rs112694524**	A	G	0.09	0.188	0.03	0.088	0.063
**rs112874012**	T	C	0.045	-0.25	0.045	0.002	0.087
**rs113825849**	G	C	0.014	0.584	0.08	-0.059	0.171
**rs115312361**	C	A	0.331	-0.184	0.023	-0.108	0.054
**rs11548656**	G	A	0.034	-0.293	0.051	-0.143	0.097
**rs116465569**	C	T	0.61	-0.201	0.021	0.037	0.048
**rs11677953**	A	G	0.415	0.098	0.017	0.009	0.035
**rs117292830**	A	G	0.027	0.43	0.058	-0.017	0.107
**rs11949375**	C	T	0.098	0.189	0.028	-0.01	0.057
**rs12446550**	A	G	0.41	0.108	0.017	-0.005	0.034
**rs1250573**	A	G	0.296	-0.114	0.019	-0.027	0.038
**rs12764283**	A	G	0.337	0.127	0.018	0.002	0.036
**rs12936409**	T	C	0.476	0.146	0.017	0.004	0.034
**rs131657**	A	T	0.202	0.136	0.021	0.012	0.044
**rs140892874**	C	T	0.027	0.41	0.051	-0.137	0.109
**rs142770866**	A	G	0.081	0.23	0.034	-0.109	0.066
**rs144515162**	G	A	0.672	-0.15	0.022	-0.017	0.056
**rs145126485**	C	A	0.033	0.283	0.05	-0.002	0.104
**rs148844907**	A	T	0.01	1.138	0.096	-0.212	0.413
**rs1551399**	C	A	0.616	0.101	0.017	0.016	0.034
**rs1736161**	A	G	0.429	-0.123	0.017	0.049	0.036
**rs1873625**	A	C	0.323	0.177	0.018	0.047	0.036
**rs1886731**	C	T	0.481	-0.097	0.018	0.046	0.035
**rs1887428**	C	G	0.619	-0.172	0.018	-0.017	0.035
**rs2076756**	G	A	0.271	0.188	0.019	-0.025	0.038
**rs2129944**	G	T	0.291	-0.119	0.02	-0.009	0.039
**rs2193041**	G	A	0.389	0.134	0.017	0.014	0.035
**rs2230365**	T	C	0.163	0.154	0.023	-0.069	0.046
**rs2241878**	C	T	0.538	0.148	0.017	-0.043	0.034
**rs2542147**	T	G	0.837	-0.151	0.023	-0.029	0.047

**rs254559**	A	C	0.402	0.103	0.017	0.032	0.034
**rs2836882**	A	G	0.258	-0.196	0.02	0.052	0.038
**rs28383456**	T	C	0.345	-0.178	0.02	-0.086	0.038
**rs3024493**	A	C	0.17	0.213	0.022	-0.058	0.047
**rs3091316**	A	G	0.267	-0.112	0.019	-0.012	0.038
**rs34190331**	A	G	0.085	0.177	0.03	-0.004	0.063
**rs34920465**	G	A	0.172	-0.139	0.023	0.045	0.044
**rs35260072**	C	A	0.433	0.142	0.017	0.006	0.034
**rs35730213**	C	G	0.263	-0.151	0.019	-0.073	0.039
**rs3850378**	C	T	0.096	0.155	0.028	-0.041	0.059
**rs4077515**	T	C	0.424	0.179	0.017	0.001	0.034
**rs4246905**	C	T	0.73	0.163	0.02	-0.017	0.038
**rs444210**	G	A	0.546	0.11	0.017	0.025	0.034
**rs45528737**	T	C	0.112	0.167	0.03	-0.004	0.063
**rs4676410**	A	G	0.195	0.155	0.023	-0.067	0.044
**rs4712528**	C	G	0.788	0.123	0.021	-0.03	0.041
**rs4730272**	G	A	0.513	-0.134	0.018	-0.014	0.034
**rs4807569**	C	A	0.22	0.139	0.021	-0.027	0.041
**rs56062135**	T	C	0.237	0.151	0.02	0.044	0.04
**rs56167332**	A	C	0.346	0.156	0.019	0.024	0.038
**rs6062496**	A	G	0.578	0.165	0.018	-0.072	0.035
**rs6584283**	C	T	0.511	-0.18	0.017	0.021	0.034
**rs6826501**	T	C	0.536	-0.093	0.017	-0.008	0.034
**rs6873866**	C	T	0.529	-0.107	0.018	0.016	0.036
**rs6880778**	G	A	0.621	0.188	0.017	0.042	0.034
**rs6911490**	C	T	0.779	-0.143	0.021	0.033	0.043
**rs6927172**	G	C	0.214	0.11	0.02	0.02	0.042
**rs72798422**	C	T	0.042	0.278	0.043	-0.1	0.087
**rs7282490**	A	G	0.598	-0.145	0.017	0.051	0.035
**rs7285952**	G	T	0.157	-0.176	0.024	-0.08	0.046
**rs744166**	G	A	0.41	-0.121	0.017	-0.037	0.034
**rs7523335**	A	G	0.173	-0.141	0.023	0.05	0.044
**rs75565243**	A	G	0.029	0.349	0.055	0.032	0.102
**rs7797798**	G	C	0.416	-0.096	0.017	-0.037	0.035
**rs9272514**	T	C	0.306	-0.235	0.021	-0.094	0.039
**rs9370774**	C	T	0.201	-0.131	0.022	-0.048	0.043
**rs9934775**	T	C	0.161	-0.14	0.023	-0.091	0.046

Table S25. Raw data for the MR analysis of the causal effect of CD on OCD							
SNP	Effect allele	Other allele	Effect allele frequency	Beta.exposure	Se.exposure	Beta.outcome	Se.outcome
**rs10045431**	C	A	0.716	0.216	0.026	-0.071	0.036
**rs10055349**	A	G	0.222	0.214	0.027	-0.058	0.042
**rs1056441**	C	T	0.698	0.167	0.026	-0.049	0.037
**rs10748781**	A	C	0.551	-0.219	0.024	0.015	0.035
**rs10761659**	G	A	0.553	0.212	0.024	0.049	0.035
**rs111564463**	G	A	0.021	0.563	0.093	-0.056	0.165
**rs11209026**	A	G	0.056	-0.995	0.064	-0.052	0.068
**rs11236797**	A	C	0.473	0.181	0.023	-0.045	0.034
**rs112401990**	A	G	0.373	0.132	0.024	-0.027	0.035
**rs114607072**	T	G	0.04	0.442	0.063	0.089	0.096
**rs11564236**	T	A	0.034	0.519	0.06	-0.119	0.103
**rs12194825**	A	T	0.186	-0.172	0.03	0.037	0.043
**rs1250573**	A	G	0.287	-0.171	0.026	-0.027	0.038
**rs12692254**	T	A	0.543	0.301	0.023	-0.038	0.034
**rs12717899**	T	G	0.794	0.159	0.029	-0.026	0.042
**rs12936409**	T	C	0.471	0.138	0.023	0.004	0.034
**rs1297271**	T	C	0.43	-0.155	0.024	0.05	0.035
**rs13135092**	G	A	0.095	0.221	0.039	0.043	0.06
**rs143345302**	T	C	0.134	0.212	0.036	-0.093	0.054
**rs145126485**	C	A	0.036	0.627	0.062	-0.002	0.104
**rs1456896**	T	C	0.698	0.139	0.025	0.03	0.036
**rs147018773**	T	C	0.097	0.322	0.038	-0.012	0.059
**rs147684209**	C	T	0.369	0.155	0.024	0.023	0.036
**rs148844907**	A	T	0.008	0.958	0.142	-0.212	0.413
**rs151314883**	A	G	0.158	-0.224	0.033	-0.079	0.046
**rs1873625**	A	C	0.32	0.181	0.024	0.047	0.036
**rs1887428**	C	G	0.623	-0.168	0.024	-0.017	0.035
**rs1932990**	T	C	0.254	0.153	0.026	0.057	0.039
**rs2076756**	G	A	0.284	0.4	0.024	-0.025	0.038
**rs2129944**	G	T	0.291	-0.156	0.027	-0.009	0.039
**rs2188962**	T	C	0.44	0.212	0.023	0.017	0.034
**rs2505640**	G	A	0.644	-0.146	0.024	-0.004	0.035
**rs281379**	A	G	0.489	0.14	0.024	0.106	0.034
**rs2856997**	A	C	0.421	0.141	0.023	-0.012	0.034
**rs28701841**	A	G	0.117	0.224	0.037	-0.037	0.057
**rs3024505**	A	G	0.162	0.178	0.03	-0.057	0.047
**rs3091315**	G	A	0.266	-0.18	0.026	-0.014	0.038
**rs3810936**	C	T	0.698	0.208	0.026	-0.019	0.037
**rs4077515**	T	C	0.42	0.216	0.024	0.001	0.034
**rs444210**	G	A	0.547	0.163	0.023	0.025	0.034
**rs4486887**	T	C	0.694	-0.196	0.025	-0.087	0.037
**rs4820091**	G	T	0.198	0.172	0.028	0.016	0.043

**rs4851586**	C	T	0.76	-0.169	0.026	-0.032	0.04
**rs4902642**	A	G	0.409	-0.129	0.024	0.036	0.034
**rs56062135**	T	C	0.234	0.193	0.027	0.044	0.04
**rs56167332**	A	C	0.343	0.17	0.026	0.024	0.038
**rs6588243**	C	A	0.59	0.132	0.023	0.023	0.034
**rs6704109**	T	C	0.256	0.202	0.026	0.023	0.039
**rs6873866**	C	T	0.535	-0.168	0.024	0.016	0.036
**rs697693**	A	G	0.201	0.172	0.028	0.03	0.042
**rs7276302**	G	A	0.608	-0.172	0.023	0.058	0.035
**rs72798422**	C	T	0.048	0.59	0.051	-0.1	0.087
**rs74179925**	T	A	0.205	-0.173	0.03	-0.027	0.043
**rs7423615**	T	C	0.194	0.164	0.029	-0.032	0.044
**rs744166**	G	A	0.408	-0.129	0.023	-0.037	0.034
**rs7499231**	G	A	0.124	-0.239	0.042	-0.027	0.055
**rs7543234**	T	C	0.239	0.155	0.027	-0.011	0.04
**rs76532080**	T	C	0.068	0.358	0.052	-0.083	0.083
**rs7713270**	T	C	0.624	0.297	0.024	0.043	0.034
**rs78487399**	C	G	0.101	0.226	0.037	0.144	0.057
**rs80262450**	A	G	0.113	0.283	0.035	0.04	0.055
**rs8178977**	C	G	0.239	0.193	0.027	-0.025	0.041
**rs921720**	G	A	0.619	0.163	0.024	0.015	0.034

Table S26 Raw data for the MR analysis of the causal effect of UC on OCD							
SNP	Effect allele	Other allele	Effect allele frequency	Beta.exposure	Se.exposure	Beta.outcome	Se.outcome
**rs10045431**	C	A	0.713	0.145	0.024	-0.071	0.036
**rs10182512**	A	G	0.35	0.161	0.022	-0.025	0.036
**rs10272963**	T	C	0.426	-0.172	0.022	-0.044	0.034
**rs10737481**	G	T	0.556	0.25	0.022	-0.001	0.034
**rs10917547**	T	A	0.362	-0.173	0.022	0.002	0.035
**rs11209026**	A	G	0.059	-0.562	0.052	-0.052	0.068
**rs114152040**	A	G	0.032	0.34	0.062	-0.008	0.104
**rs115312361**	C	A	0.329	-0.324	0.029	-0.108	0.054
**rs117292830**	A	G	0.027	0.615	0.07	-0.017	0.107
**rs12612675**	G	A	0.403	0.123	0.022	0.012	0.035
**rs12817473**	G	A	0.382	0.191	0.022	0.018	0.035
**rs1317209**	A	G	0.188	0.146	0.027	-0.007	0.043
**rs1359946**	A	G	0.196	0.158	0.027	-0.015	0.044
**rs137845**	G	A	0.515	0.118	0.021	0.045	0.034
**rs143210366**	G	T	0.037	0.39	0.057	0.013	0.089
**rs144515162**	G	A	0.671	-0.232	0.027	-0.017	0.056
**rs144582178**	C	T	0.672	-0.276	0.028	0.012	0.052
**rs148844907**	A	T	0.01	1.341	0.109	-0.212	0.413
**rs1801274**	G	A	0.483	-0.183	0.022	0.031	0.034
**rs183231933**	T	C	0.24	-0.371	0.031	-0.067	0.047
**rs1886731**	C	T	0.481	-0.141	0.022	0.046	0.035
**rs1887428**	C	G	0.623	-0.177	0.022	-0.017	0.035
**rs2212434**	T	C	0.46	0.142	0.021	-0.044	0.034
**rs2301989**	A	G	0.402	-0.141	0.022	-0.022	0.035
**rs254559**	A	C	0.404	0.124	0.022	0.032	0.034
**rs28383456**	T	C	0.335	-0.337	0.026	-0.086	0.038
**rs3024493**	A	C	0.168	0.236	0.028	-0.058	0.047
**rs34920465**	G	A	0.17	-0.193	0.029	0.045	0.044
**rs35730213**	C	G	0.267	-0.167	0.025	-0.073	0.039
**rs3829111**	A	G	0.417	0.156	0.021	-0.004	0.034
**rs45627734**	A	G	0.03	0.406	0.063	0.036	0.104
**rs4574921**	T	C	0.741	0.151	0.026	-0.001	0.039
**rs4676410**	A	G	0.197	0.208	0.028	-0.067	0.044
**rs483905**	A	G	0.294	0.129	0.023	0.041	0.037
**rs484356**	G	C	0.328	-0.134	0.023	-0.017	0.036
**rs56167332**	A	C	0.342	0.152	0.023	0.024	0.038
**rs6017342**	C	A	0.538	0.191	0.024	-0.008	0.04
**rs6062496**	A	G	0.573	0.158	0.022	-0.072	0.035
**rs6933404**	C	T	0.216	0.167	0.025	0.02	0.042
**rs7282490**	A	G	0.604	-0.14	0.021	0.051	0.035
**rs7523335**	A	G	0.177	-0.17	0.029	0.05	0.044
**rs7752873**	T	C	0.137	0.182	0.03	0	0.049

**rs7911680**	C	A	0.49	-0.172	0.021	-0.001	0.034
**rs798502**	C	A	0.283	-0.136	0.024	0.01	0.038
**rs9272514**	T	C	0.298	-0.402	0.027	-0.094	0.039
**rs9823546**	A	T	0.31	0.177	0.022	0.046	0.036
**rs9891174**	A	T	0.47	0.145	0.021	0.006	0.034
**rs9977672**	A	G	0.251	-0.245	0.026	0.036	0.039

Table S27. Raw data for the MR analysis of the causal effect of IBD on PTSD							
**SNP**	**Effect allele**	**Other allele**	**Effect allele frequency**	**Beta.exposure**	**Se.exposure**	**Beta.outcome**	**Se.outcome**
**rs1003342**	G	A	0.531	-0.095	0.017	0.012	0.015
**rs10045431**	C	A	0.718	0.177	0.019	0.007	0.016
**rs10175585**	A	G	0.713	-0.133	0.02	-0.014	0.019
**rs10408351**	A	G	0.238	0.138	0.022	0.02	0.018
**rs10737481**	G	T	0.555	0.141	0.017	-0.015	0.015
**rs10761659**	G	A	0.554	0.162	0.017	0.015	0.015
**rs10800314**	A	C	0.649	-0.143	0.018	-0.021	0.017
**rs10826797**	T	G	0.306	-0.102	0.019	-0.003	0.016
**rs10917547**	T	A	0.362	-0.1	0.018	-0.008	0.015
**rs11209026**	A	G	0.054	-0.726	0.042	-0.011	0.03
**rs11236797**	A	C	0.47	0.156	0.017	0.008	0.015
**rs112401990**	A	G	0.375	0.142	0.017	0.016	0.015
**rs112694524**	A	G	0.09	0.188	0.03	0.003	0.032
**rs112874012**	T	C	0.045	-0.25	0.045	0.068	0.036
**rs113825849**	G	C	0.014	0.584	0.08	0.001	0.059
**rs11548656**	G	A	0.034	-0.293	0.051	0.03	0.041
**rs116465569**	C	T	0.61	-0.201	0.021	-0.03	0.018
**rs11677953**	A	G	0.415	0.098	0.017	0.014	0.015
**rs117292830**	A	G	0.027	0.43	0.058	0.133	0.047
**rs11949375**	C	T	0.098	0.189	0.028	0.001	0.028
**rs12446550**	A	G	0.41	0.108	0.017	0.005	0.015
**rs1250573**	A	G	0.296	-0.114	0.019	-0.004	0.016
**rs12764283**	A	G	0.337	0.127	0.018	0.012	0.016
**rs12936409**	T	C	0.476	0.146	0.017	0.008	0.015
**rs131657**	A	T	0.202	0.136	0.021	-0.037	0.02
**rs140892874**	C	T	0.027	0.41	0.051	0.04	0.053
**rs142770866**	A	G	0.081	0.23	0.034	0.014	0.028
**rs144515162**	G	A	0.672	-0.15	0.022	-0.066	0.064
**rs145126485**	C	A	0.033	0.283	0.05	0.089	0.045
**rs148844907**	A	T	0.01	1.138	0.096	0.029	0.078
**rs1551399**	C	A	0.616	0.101	0.017	0.004	0.015
**rs1736161**	A	G	0.429	-0.123	0.017	0.022	0.015
**rs1873625**	A	C	0.323	0.177	0.018	0.014	0.016
**rs1886731**	C	T	0.481	-0.097	0.018	-0.002	0.015
**rs1887428**	C	G	0.619	-0.172	0.018	-0.014	0.016
**rs2076756**	G	A	0.271	0.188	0.019	0.028	0.017
**rs2129944**	G	T	0.291	-0.119	0.02	0.008	0.025
**rs2193041**	G	A	0.389	0.134	0.017	0.009	0.015
**rs2230365**	T	C	0.163	0.154	0.023	0.019	0.021
**rs2241878**	C	T	0.538	0.148	0.017	0.013	0.015
**rs2542147**	T	G	0.837	-0.151	0.023	-0.023	0.02
**rs254559**	A	C	0.402	0.103	0.017	0.037	0.015

**rs2836882**	A	G	0.258	-0.196	0.02	-0.011	0.017
**rs28383456**	T	C	0.345	-0.178	0.02	0.028	0.049
**rs3024493**	A	C	0.17	0.213	0.022	0.011	0.02
**rs3091316**	A	G	0.267	-0.112	0.019	-0.019	0.017
**rs34190331**	A	G	0.085	0.177	0.03	0.002	0.03
**rs34920465**	G	A	0.172	-0.139	0.023	0.001	0.02
**rs35260072**	C	A	0.433	0.142	0.017	0.003	0.015
**rs35730213**	C	G	0.263	-0.151	0.019	-0.017	0.016
**rs3850378**	C	T	0.096	0.155	0.028	0.019	0.025
**rs4077515**	T	C	0.424	0.179	0.017	-0.042	0.015
**rs4246905**	C	T	0.73	0.163	0.02	0.01	0.017
**rs444210**	G	A	0.546	0.11	0.017	0.008	0.015
**rs45528737**	T	C	0.112	0.167	0.03	0.006	0.026
**rs4676410**	A	G	0.195	0.155	0.023	-0.025	0.019
**rs4712528**	C	G	0.788	0.123	0.021	0	0.018
**rs4730272**	G	A	0.513	-0.134	0.018	0.005	0.015
**rs4807569**	C	A	0.22	0.139	0.021	-0.038	0.019
**rs56062135**	T	C	0.237	0.151	0.02	0.016	0.017
**rs56167332**	A	C	0.346	0.156	0.019	0.003	0.017
**rs6062496**	A	G	0.578	0.165	0.018	-0.009	0.015
**rs6584283**	C	T	0.511	-0.18	0.017	0.015	0.015
**rs6826501**	T	C	0.536	-0.093	0.017	0.004	0.015
**rs6873866**	C	T	0.529	-0.107	0.018	0.014	0.016
**rs6880778**	G	A	0.621	0.188	0.017	0.008	0.015
**rs6911490**	C	T	0.779	-0.143	0.021	0.008	0.018
**rs6927172**	G	C	0.214	0.11	0.02	0.018	0.018
**rs72798422**	C	T	0.042	0.278	0.043	0.041	0.041
**rs7282490**	A	G	0.598	-0.145	0.017	-0.006	0.015
**rs7285952**	G	T	0.157	-0.176	0.024	0.042	0.02
**rs744166**	G	A	0.41	-0.121	0.017	-0.007	0.015
**rs7523335**	A	G	0.173	-0.141	0.023	0.03	0.019
**rs75565243**	A	G	0.029	0.349	0.055	0.061	0.049
**rs7797798**	G	C	0.416	-0.096	0.017	-0.022	0.015
**rs9272514**	T	C	0.306	-0.235	0.021	0.026	0.018
**rs9370774**	C	T	0.201	-0.131	0.022	-0.009	0.019
**rs9934775**	T	C	0.161	-0.14	0.023	-0.005	0.02

Table S28. Raw data for the MR analysis of the causal effect of CD on PTSD							
SNP	Effect allele	Other allele	Effect allele frequency	Beta.exposure	Se.exposure	Beta.outcome	Se.outcome
**rs10045431**	C	A	0.716	0.216	0.026	0.007	0.016
**rs10055349**	A	G	0.222	0.214	0.027	-0.009	0.018
**rs1056441**	C	T	0.698	0.167	0.026	-0.015	0.016
**rs10748781**	A	C	0.551	-0.219	0.024	0.01	0.015
**rs10761659**	G	A	0.553	0.212	0.024	0.015	0.015
**rs111564463**	G	A	0.021	0.563	0.093	0.02	0.069
**rs11209026**	A	G	0.056	-0.995	0.064	-0.011	0.03
**rs11236797**	A	C	0.473	0.181	0.023	0.008	0.015
**rs112401990**	A	G	0.373	0.132	0.024	0.016	0.015
**rs114607072**	T	G	0.04	0.442	0.063	0.061	0.039
**rs11564236**	T	A	0.034	0.519	0.06	0.039	0.053
**rs12194825**	A	T	0.186	-0.172	0.03	-0.006	0.019
**rs1250573**	A	G	0.287	-0.171	0.026	-0.004	0.016
**rs12692254**	T	A	0.543	0.301	0.023	0.013	0.015
**rs12717899**	T	G	0.794	0.159	0.029	0.023	0.018
**rs12936409**	T	C	0.471	0.138	0.023	0.008	0.015
**rs1297271**	T	C	0.43	-0.155	0.024	0.024	0.015
**rs13135092**	G	A	0.095	0.221	0.039	0.088	0.027
**rs143345302**	T	C	0.134	0.212	0.036	0.014	0.022
**rs145126485**	C	A	0.036	0.627	0.062	0.089	0.045
**rs1456896**	T	C	0.698	0.139	0.025	0.001	0.016
**rs147018773**	T	C	0.097	0.322	0.038	0.009	0.029
**rs147684209**	C	T	0.369	0.155	0.024	0.004	0.016
**rs148844907**	A	T	0.008	0.958	0.142	0.029	0.078
**rs151314883**	A	G	0.158	-0.224	0.033	0.043	0.02
**rs1873625**	A	C	0.32	0.181	0.024	0.014	0.016
**rs1887428**	C	G	0.623	-0.168	0.024	-0.014	0.016
**rs1932990**	T	C	0.254	0.153	0.026	-0.011	0.018
**rs2076756**	G	A	0.284	0.4	0.024	0.028	0.017
**rs2129944**	G	T	0.291	-0.156	0.027	0.008	0.025
**rs2188962**	T	C	0.44	0.212	0.023	-0.005	0.015
**rs2505640**	G	A	0.644	-0.146	0.024	-0.001	0.015
**rs281379**	A	G	0.489	0.14	0.024	0.01	0.015
**rs2856997**	A	C	0.421	0.141	0.023	0.049	0.015
**rs28701841**	A	G	0.117	0.224	0.037	-0.008	0.025
**rs3024505**	A	G	0.162	0.178	0.03	0.009	0.02
**rs3091315**	G	A	0.266	-0.18	0.026	-0.017	0.017
**rs3810936**	C	T	0.698	0.208	0.026	0.003	0.016
**rs4077515**	T	C	0.42	0.216	0.024	-0.042	0.015
**rs444210**	G	A	0.547	0.163	0.023	0.008	0.015
**rs4486887**	T	C	0.694	-0.196	0.025	0.039	0.016
**rs4820091**	G	T	0.198	0.172	0.028	-0.018	0.019

**rs4851586**	C	T	0.76	-0.169	0.026	-0.017	0.017
**rs4902642**	A	G	0.409	-0.129	0.024	-0.006	0.015
**rs56062135**	T	C	0.234	0.193	0.027	0.016	0.017
**rs56167332**	A	C	0.343	0.17	0.026	0.003	0.017
**rs6588243**	C	A	0.59	0.132	0.023	-0.019	0.015
**rs6704109**	T	C	0.256	0.202	0.026	-0.008	0.017
**rs6873866**	C	T	0.535	-0.168	0.024	0.014	0.016
**rs697693**	A	G	0.201	0.172	0.028	0.02	0.019
**rs7276302**	G	A	0.608	-0.172	0.023	-0.007	0.015
**rs72798422**	C	T	0.048	0.59	0.051	0.041	0.041
**rs74179925**	T	A	0.205	-0.173	0.03	-0.014	0.018
**rs7423615**	T	C	0.194	0.164	0.029	-0.031	0.019
**rs744166**	G	A	0.408	-0.129	0.023	-0.007	0.015
**rs7499231**	G	A	0.124	-0.239	0.042	-0.031	0.025
**rs7543234**	T	C	0.239	0.155	0.027	0.006	0.018
**rs76532080**	T	C	0.068	0.358	0.052	0.011	0.034
**rs7713270**	T	C	0.624	0.297	0.024	0.006	0.015
**rs78487399**	C	G	0.101	0.226	0.037	0.014	0.025
**rs80262450**	A	G	0.113	0.283	0.035	0.006	0.023
**rs8178977**	C	G	0.239	0.193	0.027	-0.037	0.018
**rs921720**	G	A	0.619	0.163	0.024	0.006	0.015

Table S29. Raw data for the MR analysis of the causal effect of UC on PTSD							
SNP	Effect allele	Other allele	Effect allele frequency	Beta.exposure	Se.exposure	Beta.outcome	Se.outcome
**rs10045431**	C	A	0.713	0.145	0.024	0.007	0.016
**rs10182512**	A	G	0.35	0.161	0.022	0.018	0.015
**rs10272963**	T	C	0.426	-0.172	0.022	-0.002	0.015
**rs10737481**	G	T	0.556	0.25	0.022	-0.015	0.015
**rs10917547**	T	A	0.362	-0.173	0.022	-0.008	0.015
**rs11209026**	A	G	0.059	-0.562	0.052	-0.011	0.03
**rs114152040**	A	G	0.032	0.34	0.062	0.026	0.041
**rs117292830**	A	G	0.027	0.615	0.07	0.133	0.047
**rs12612675**	G	A	0.403	0.123	0.022	0.018	0.015
**rs12817473**	G	A	0.382	0.191	0.022	0.007	0.015
**rs1317209**	A	G	0.188	0.146	0.027	-0.004	0.019
**rs1359946**	A	G	0.196	0.158	0.027	0.003	0.019
**rs137845**	G	A	0.515	0.118	0.021	0.005	0.015
**rs143210366**	G	T	0.037	0.39	0.057	-0.008	0.049
**rs144515162**	G	A	0.671	-0.232	0.027	-0.066	0.064
**rs144582178**	C	T	0.672	-0.276	0.028	-0.114	0.066
**rs148844907**	A	T	0.01	1.341	0.109	0.029	0.078
**rs1801274**	G	A	0.483	-0.183	0.022	-0.016	0.015
**rs183231933**	T	C	0.24	-0.371	0.031	-0.035	0.019
**rs1886731**	C	T	0.481	-0.141	0.022	-0.002	0.015
**rs1887428**	C	G	0.623	-0.177	0.022	-0.014	0.016
**rs2212434**	T	C	0.46	0.142	0.021	0.007	0.015
**rs2301989**	A	G	0.402	-0.141	0.022	-0.022	0.015
**rs254559**	A	C	0.404	0.124	0.022	0.037	0.015
**rs28383456**	T	C	0.335	-0.337	0.026	0.028	0.049
**rs3024493**	A	C	0.168	0.236	0.028	0.011	0.02
**rs34920465**	G	A	0.17	-0.193	0.029	0.001	0.02
**rs35730213**	C	G	0.267	-0.167	0.025	-0.017	0.016
**rs3829111**	A	G	0.417	0.156	0.021	-0.037	0.015
**rs45627734**	A	G	0.03	0.406	0.063	0.065	0.046
**rs4574921**	T	C	0.741	0.151	0.026	0.008	0.017
**rs4676410**	A	G	0.197	0.208	0.028	-0.025	0.019
**rs483905**	A	G	0.294	0.129	0.023	0.004	0.016
**rs484356**	G	C	0.328	-0.134	0.023	0.001	0.017
**rs56167332**	A	C	0.342	0.152	0.023	0.003	0.017
**rs6017342**	C	A	0.538	0.191	0.024	0.003	0.015
**rs6062496**	A	G	0.573	0.158	0.022	-0.009	0.015
**rs6933404**	C	T	0.216	0.167	0.025	0.017	0.018
**rs7282490**	A	G	0.604	-0.14	0.021	-0.006	0.015
**rs7523335**	A	G	0.177	-0.17	0.029	0.03	0.019
**rs7752873**	T	C	0.137	0.182	0.03	0.004	0.022
**rs7911680**	C	A	0.49	-0.172	0.021	0.017	0.015

**rs798502**	C	A	0.283	-0.136	0.024	0.004	0.016
**rs9272514**	T	C	0.298	-0.402	0.027	0.026	0.018
**rs9823546**	A	T	0.31	0.177	0.022	0.014	0.016
**rs9891174**	A	T	0.47	0.145	0.021	0.008	0.015
**rs9977672**	A	G	0.251	-0.245	0.026	-0.009	0.017

Table S30. Raw data for the MR analysis of the causal effect of IBD on schizophrenia							
SNP	Effect allele	Other allele	Effect allele frequency	Beta.exposure	Se.exposure	Beta.outcome	Se.outcome
**rs1003342**	G	A	0.531	-0.095	0.017	-0.011	0.009
**rs10045431**	C	A	0.718	0.177	0.019	0	0.01
**rs10408351**	A	G	0.238	0.138	0.022	-0.008	0.011
**rs10737481**	G	T	0.555	0.141	0.017	-0.004	0.009
**rs10761659**	G	A	0.554	0.162	0.017	-0.025	0.009
**rs10826797**	T	G	0.306	-0.102	0.019	0.025	0.01
**rs10917547**	T	A	0.362	-0.1	0.018	0.003	0.009
**rs11209026**	A	G	0.054	-0.726	0.042	-0.008	0.018
**rs11236797**	A	C	0.47	0.156	0.017	0.016	0.009
**rs112401990**	A	G	0.375	0.142	0.017	-0.022	0.009
**rs112874012**	T	C	0.045	-0.25	0.045	0.006	0.021
**rs113825849**	G	C	0.014	0.584	0.08	-0.028	0.037
**rs11548656**	G	A	0.034	-0.293	0.051	0.004	0.024
**rs11677953**	A	G	0.415	0.098	0.017	-0.013	0.009
**rs117292830**	A	G	0.027	0.43	0.058	0.067	0.026
**rs11949375**	C	T	0.098	0.189	0.028	-0.019	0.019
**rs12446550**	A	G	0.41	0.108	0.017	0.018	0.009
**rs1250573**	A	G	0.296	-0.114	0.019	0.01	0.009
**rs12764283**	A	G	0.337	0.127	0.018	0.03	0.009
**rs12936409**	T	C	0.476	0.146	0.017	-0.018	0.009
**rs140892874**	C	T	0.027	0.41	0.051	0.028	0.03
**rs142770866**	A	G	0.081	0.23	0.034	0.015	0.017
**rs145126485**	C	A	0.033	0.283	0.05	-0.02	0.027
**rs148844907**	A	T	0.01	1.138	0.096	0.087	0.052
**rs1551399**	C	A	0.616	0.101	0.017	-0.003	0.009
**rs1736161**	A	G	0.429	-0.123	0.017	-0.005	0.009
**rs1873625**	A	C	0.323	0.177	0.018	0.027	0.009
**rs1886731**	C	T	0.481	-0.097	0.018	-0.003	0.009
**rs1887428**	C	G	0.619	-0.172	0.018	-0.004	0.009
**rs2076756**	G	A	0.271	0.188	0.019	0.018	0.01
**rs2129944**	G	T	0.291	-0.119	0.02	-0.003	0.015
**rs2193041**	G	A	0.389	0.134	0.017	-0.004	0.009
**rs2230365**	T	C	0.163	0.154	0.023	0.035	0.012
**rs2241878**	C	T	0.538	0.148	0.017	0.012	0.009
**rs2542147**	T	G	0.837	-0.151	0.023	0.004	0.012
**rs254559**	A	C	0.402	0.103	0.017	-0.005	0.009
**rs2836882**	A	G	0.258	-0.196	0.02	0.009	0.01
**rs28383456**	T	C	0.345	-0.178	0.02	0.029	0.015
**rs3024493**	A	C	0.17	0.213	0.022	-0.001	0.012
**rs3091316**	A	G	0.267	-0.112	0.019	-0.008	0.01
**rs34190331**	A	G	0.085	0.177	0.03	0.044	0.017
**rs34920465**	G	A	0.172	-0.139	0.023	-0.008	0.012

**rs35260072**	C	A	0.433	0.142	0.017	-0.014	0.009
**rs35730213**	C	G	0.263	-0.151	0.019	-0.037	0.01
**rs3850378**	C	T	0.096	0.155	0.028	-0.005	0.015
**rs4077515**	T	C	0.424	0.179	0.017	0.009	0.009
**rs4246905**	C	T	0.73	0.163	0.02	0.006	0.01
**rs444210**	G	A	0.546	0.11	0.017	-0.007	0.009
**rs45528737**	T	C	0.112	0.167	0.03	0.011	0.015
**rs4676410**	A	G	0.195	0.155	0.023	0.017	0.011
**rs4712528**	C	G	0.788	0.123	0.021	-0.006	0.01
**rs4730272**	G	A	0.513	-0.134	0.018	0.006	0.009
**rs4807569**	C	A	0.22	0.139	0.021	0.02	0.011
**rs56062135**	T	C	0.237	0.151	0.02	-0.01	0.01
**rs56167332**	A	C	0.346	0.156	0.019	0.003	0.009
**rs6062496**	A	G	0.578	0.165	0.018	-0.003	0.009
**rs6584283**	C	T	0.511	-0.18	0.017	0.006	0.009
**rs6826501**	T	C	0.536	-0.093	0.017	-0.001	0.009
**rs6880778**	G	A	0.621	0.188	0.017	-0.008	0.009
**rs6911490**	C	T	0.779	-0.143	0.021	-0.017	0.011
**rs6927172**	G	C	0.214	0.11	0.02	0.019	0.011
**rs72798422**	C	T	0.042	0.278	0.043	0.016	0.024
**rs7282490**	A	G	0.598	-0.145	0.017	-0.017	0.009
**rs7285952**	G	T	0.157	-0.176	0.024	0.002	0.012
**rs744166**	G	A	0.41	-0.121	0.017	-0.002	0.009
**rs7523335**	A	G	0.173	-0.141	0.023	0.005	0.011
**rs75565243**	A	G	0.029	0.349	0.055	0.142	0.05
**rs7797798**	G	C	0.416	-0.096	0.017	0.002	0.009
**rs9370774**	C	T	0.201	-0.131	0.022	0.002	0.011
**rs9934775**	T	C	0.161	-0.14	0.023	-0.005	0.012

Table S31. Raw data for the MR analysis of the causal effect of CD on schizophrenia							
**SNP**	**Effect allele**	**Other allele**	**Effect allele frequency**	**Beta.exposure**	**Se.exposure**	**Beta.outcome**	**Se.outcome**
**rs10045431**	C	A	0.716	0.216	0.026	0	0.01
**rs10055349**	A	G	0.222	0.214	0.027	0.01	0.011
**rs1056441**	C	T	0.698	0.167	0.026	-0.008	0.009
**rs10748781**	A	C	0.551	-0.219	0.024	0.01	0.009
**rs10761659**	G	A	0.553	0.212	0.024	-0.025	0.009
**rs111564463**	G	A	0.021	0.563	0.093	0.018	0.042
**rs11209026**	A	G	0.056	-0.995	0.064	-0.008	0.018
**rs11236797**	A	C	0.473	0.181	0.023	0.016	0.009
**rs112401990**	A	G	0.373	0.132	0.024	-0.022	0.009
**rs114607072**	T	G	0.04	0.442	0.063	0.029	0.024
**rs11564236**	T	A	0.034	0.519	0.06	0.023	0.03
**rs12194825**	A	T	0.186	-0.172	0.03	0.02	0.011
**rs1250573**	A	G	0.287	-0.171	0.026	0.01	0.009
**rs12692254**	T	A	0.543	0.301	0.023	0.011	0.009
**rs12717899**	T	G	0.794	0.159	0.029	0.003	0.011
**rs12936409**	T	C	0.471	0.138	0.023	-0.018	0.009
**rs1297271**	T	C	0.43	-0.155	0.024	-0.006	0.009
**rs13135092**	G	A	0.095	0.221	0.039	0.132	0.016
**rs143345302**	T	C	0.134	0.212	0.036	0.034	0.013
**rs145126485**	C	A	0.036	0.627	0.062	-0.02	0.027
**rs1456896**	T	C	0.698	0.139	0.025	0.001	0.009
**rs148844907**	A	T	0.008	0.958	0.142	0.087	0.052
**rs151314883**	A	G	0.158	-0.224	0.033	0.002	0.012
**rs1873625**	A	C	0.32	0.181	0.024	0.027	0.009
**rs1887428**	C	G	0.623	-0.168	0.024	-0.004	0.009
**rs1932990**	T	C	0.254	0.153	0.026	0.039	0.01
**rs2076756**	G	A	0.284	0.4	0.024	0.018	0.01
**rs2129944**	G	T	0.291	-0.156	0.027	-0.003	0.015
**rs2188962**	T	C	0.44	0.212	0.023	-0.014	0.009
**rs2505640**	G	A	0.644	-0.146	0.024	-0.031	0.009
**rs281379**	A	G	0.489	0.14	0.024	0.04	0.009
**rs2856997**	A	C	0.421	0.141	0.023	0.014	0.009
**rs28701841**	A	G	0.117	0.224	0.037	0.029	0.015
**rs3024505**	A	G	0.162	0.178	0.03	-0.001	0.012
**rs3091315**	G	A	0.266	-0.18	0.026	-0.008	0.01
**rs3810936**	C	T	0.698	0.208	0.026	0.007	0.009
**rs4077515**	T	C	0.42	0.216	0.024	0.009	0.009
**rs444210**	G	A	0.547	0.163	0.023	-0.007	0.009
**rs4486887**	T	C	0.694	-0.196	0.025	-0.016	0.01
**rs4820091**	G	T	0.198	0.172	0.028	0.021	0.011
**rs4851586**	C	T	0.76	-0.169	0.026	0	0.01
**rs4902642**	A	G	0.409	-0.129	0.024	-0.004	0.009

**rs56062135**	T	C	0.234	0.193	0.027	-0.01	0.01
**rs56167332**	A	C	0.343	0.17	0.026	0.003	0.009
**rs6588243**	C	A	0.59	0.132	0.023	0.005	0.009
**rs6704109**	T	C	0.256	0.202	0.026	0	0.01
**rs697693**	A	G	0.201	0.172	0.028	-0.016	0.011
**rs7276302**	G	A	0.608	-0.172	0.023	-0.017	0.009
**rs72798422**	C	T	0.048	0.59	0.051	0.016	0.024
**rs74179925**	T	A	0.205	-0.173	0.03	0.005	0.011
**rs7423615**	T	C	0.194	0.164	0.029	-0.009	0.011
**rs744166**	G	A	0.408	-0.129	0.023	-0.002	0.009
**rs7499231**	G	A	0.124	-0.239	0.042	0.001	0.015
**rs76532080**	T	C	0.068	0.358	0.052	-0.037	0.021
**rs7713270**	T	C	0.624	0.297	0.024	-0.007	0.009
**rs78487399**	C	G	0.101	0.226	0.037	-0.006	0.015
**rs80262450**	A	G	0.113	0.283	0.035	-0.003	0.013
**rs8178977**	C	G	0.239	0.193	0.027	0.024	0.01
**rs921720**	G	A	0.619	0.163	0.024	-0.004	0.009

Table S32. Raw data for the MR analysis of the causal effect of UC on schizophrenia							
**SNP**	**Effect allele**	**Other allele**	**Effect allele frequency**	**Beta.exposure**	**Se.exposure**	**Beta.outcome**	**Se.outcome**
**rs10045431**	C	A	0.713	0.145	0.024	0	0.01
**rs10182512**	A	G	0.35	0.161	0.022	-0.02	0.009
**rs10272963**	T	C	0.426	-0.172	0.022	0	0.009
**rs10737481**	G	T	0.556	0.25	0.022	-0.004	0.009
**rs10917547**	T	A	0.362	-0.173	0.022	0.003	0.009
**rs11209026**	A	G	0.059	-0.562	0.052	-0.008	0.018
**rs114152040**	A	G	0.032	0.34	0.062	0.011	0.024
**rs117292830**	A	G	0.027	0.615	0.07	0.067	0.026
**rs12612675**	G	A	0.403	0.123	0.022	-0.013	0.009
**rs12817473**	G	A	0.382	0.191	0.022	-0.005	0.009
**rs1317209**	A	G	0.188	0.146	0.027	-0.006	0.011
**rs1359946**	A	G	0.196	0.158	0.027	0.009	0.011
**rs137845**	G	A	0.515	0.118	0.021	0.022	0.009
**rs148844907**	A	T	0.01	1.341	0.109	0.087	0.052
**rs1801274**	G	A	0.483	-0.183	0.022	0.026	0.009
**rs1886731**	C	T	0.481	-0.141	0.022	-0.003	0.009
**rs1887428**	C	G	0.623	-0.177	0.022	-0.004	0.009
**rs2212434**	T	C	0.46	0.142	0.021	0.016	0.009
**rs2301989**	A	G	0.402	-0.141	0.022	0.002	0.009
**rs254559**	A	C	0.404	0.124	0.022	-0.005	0.009
**rs28383456**	T	C	0.335	-0.337	0.026	0.029	0.015
**rs3024493**	A	C	0.168	0.236	0.028	-0.001	0.012
**rs34920465**	G	A	0.17	-0.193	0.029	-0.008	0.012
**rs35730213**	C	G	0.267	-0.167	0.025	-0.037	0.01
**rs3829111**	A	G	0.417	0.156	0.021	0.008	0.009
**rs45627734**	A	G	0.03	0.406	0.063	0.086	0.026
**rs4574921**	T	C	0.741	0.151	0.026	-0.002	0.01
**rs4676410**	A	G	0.197	0.208	0.028	0.017	0.011
**rs483905**	A	G	0.294	0.129	0.023	0.01	0.01
**rs56167332**	A	C	0.342	0.152	0.023	0.003	0.009
**rs6017342**	C	A	0.538	0.191	0.024	-0.013	0.009
**rs6062496**	A	G	0.573	0.158	0.022	-0.003	0.009
**rs6933404**	C	T	0.216	0.167	0.025	0.018	0.011
**rs7282490**	A	G	0.604	-0.14	0.021	-0.017	0.009
**rs7523335**	A	G	0.177	-0.17	0.029	0.005	0.011
**rs7752873**	T	C	0.137	0.182	0.03	0.019	0.013
**rs7911680**	C	A	0.49	-0.172	0.021	0.007	0.009
**rs798502**	C	A	0.283	-0.136	0.024	0.008	0.009
**rs9823546**	A	T	0.31	0.177	0.022	0.026	0.009
**rs9891174**	A	T	0.47	0.145	0.021	-0.017	0.009
**rs9977672**	A	G	0.251	-0.245	0.026	0.011	0.01

Table S33. Raw data for the MR analysis of the causal effect of ADHD on IBD (P<5E-5)							
**SNP**	**Effect allele**	**Other allele**	**Effect allele frequency**	**Beta.exposure**	**Se.exposure**	**Beta.outcome**	**Se.outcome**
**rs10024525**	T	C	0.811	0.075	0.017	0.022	0.022
**rs10256728**	A	T	0.303	-0.064	0.015	-0.007	0.018
**rs10262192**	A	G	0.473	0.074	0.014	-0.024	0.017
**rs10400419**	T	C	0.476	-0.067	0.015	0.011	0.017
**rs10512379**	A	G	0.087	0.107	0.025	-0.024	0.028
**rs10759922**	C	G	0.564	-0.061	0.015	-0.014	0.018
**rs1077612**	T	C	0.217	-0.076	0.017	0.046	0.021
**rs10862707**	A	C	0.145	0.097	0.022	0.023	0.025
**rs10965173**	A	G	0.759	0.079	0.017	0.036	0.022
**rs111900779**	A	G	0.075	0.105	0.025	0.029	0.031
**rs112097663**	T	C	0.163	0.080	0.019	-0.022	0.024
**rs11245604**	A	G	0.109	0.133	0.026	0.020	0.029
**rs112736460**	T	G	0.935	-0.116	0.028	-0.031	0.038
**rs112788138**	T	G	0.102	-0.094	0.023	0.015	0.028
**rs115881829**	C	G	0.944	0.129	0.032	-0.023	0.044
**rs11591402**	A	T	0.224	-0.092	0.016	0.018	0.020
**rs11682489**	T	C	0.625	0.066	0.014	-0.003	0.021
**rs11698378**	T	C	0.808	-0.085	0.018	0.006	0.022
**rs117624174**	T	C	0.059	-0.142	0.031	-0.003	0.039
**rs11773992**	C	G	0.173	0.076	0.018	-0.035	0.023
**rs11782371**	A	G	0.387	-0.059	0.014	0.013	0.018
**rs11813307**	A	G	0.480	-0.057	0.014	-0.015	0.017
**rs11936939**	T	C	0.379	-0.061	0.014	0.013	0.017
**rs11954003**	T	C	0.903	-0.100	0.024	0.010	0.029
**rs12293450**	T	C	0.207	0.074	0.017	0.030	0.021
**rs12435486**	A	G	0.249	0.066	0.016	-0.045	0.020
**rs1246287**	A	G	0.569	0.062	0.014	-0.015	0.017
**rs12475235**	A	G	0.874	-0.082	0.020	0.008	0.026
**rs1255536**	A	G	0.258	0.071	0.015	0.016	0.019
**rs1272878**	T	C	0.767	0.091	0.018	0.019	0.021
**rs1286138**	T	G	0.318	0.063	0.014	-0.012	0.018
**rs13023832**	A	G	0.104	0.115	0.022	0.058	0.028
**rs1319859**	A	G	0.666	0.060	0.014	0.006	0.018
**rs13240946**	T	C	0.483	0.064	0.016	0.009	0.019
**rs1335762**	T	C	0.597	-0.058	0.014	-0.003	0.018
**rs13418985**	A	G	0.982	-0.216	0.050	-0.037	0.062
**rs139383262**	A	G	0.017	0.265	0.058	-0.073	0.078
**rs141547796**	A	G	0.085	-0.137	0.026	-0.018	0.032
**rs1427829**	A	G	0.436	0.082	0.014	-0.013	0.017
**rs143129347**	T	C	0.070	0.125	0.028	-0.041	0.033
**rs1431592**	T	C	0.306	0.068	0.015	-0.015	0.018
**rs143912172**	A	C	0.935	0.138	0.029	0.031	0.037

**rs144104266**	A	G	0.035	0.164	0.038	0.023	0.047
**rs146130170**	A	G	0.744	0.069	0.016	-0.009	0.020
**rs146523543**	A	G	0.020	0.207	0.050	-0.041	0.066
**rs147631443**	A	G	0.412	0.058	0.014	0.002	0.018
**rs147657937**	A	G	0.027	0.197	0.046	-0.018	0.050
**rs1484144**	T	C	0.449	0.064	0.014	0.021	0.017
**rs1513155**	A	G	0.846	-0.085	0.019	-0.028	0.023
**rs1592757**	C	G	0.353	0.075	0.014	-0.001	0.018
**rs17018790**	A	T	0.861	0.088	0.020	-0.006	0.024
**rs17084312**	T	C	0.041	0.179	0.034	0.051	0.046
**rs17531412**	A	G	0.692	0.105	0.015	0.015	0.018
**rs17636214**	A	G	0.034	-0.197	0.041	0.052	0.046
**rs17652815**	T	C	0.956	-0.146	0.032	-0.056	0.043
**rs17718640**	C	G	0.321	-0.063	0.015	-0.024	0.018
**rs1791794**	A	G	0.666	-0.069	0.015	-0.050	0.018
**rs180822580**	A	G	0.051	-0.157	0.033	-0.047	0.041
**rs1833322**	A	T	0.355	0.060	0.014	0.009	0.018
**rs1844070**	A	T	0.098	-0.103	0.024	0.011	0.030
**rs185160613**	T	C	0.036	-0.195	0.042	0.066	0.050
**rs1859057**	C	G	0.474	0.063	0.014	0.001	0.017
**rs1886753**	T	C	0.389	0.056	0.014	0.000	0.017
**rs1959848**	T	C	0.516	-0.070	0.014	0.010	0.017
**rs1978102**	T	C	0.529	0.059	0.014	-0.001	0.017
**rs2090317**	A	G	0.335	0.063	0.014	0.004	0.018
**rs2110042**	A	C	0.594	-0.060	0.014	0.002	0.017
**rs2113808**	A	G	0.647	0.062	0.015	0.032	0.018
**rs212178**	A	G	0.884	-0.117	0.021	0.009	0.027
**rs2144782**	T	C	0.378	0.071	0.015	-0.024	0.018
**rs2254448**	C	G	0.210	0.070	0.016	0.034	0.020
**rs227378**	A	C	0.666	0.079	0.015	-0.035	0.019
**rs2300861**	T	C	0.541	-0.062	0.014	0.000	0.017
**rs2302793**	A	G	0.551	0.061	0.014	-0.015	0.017
**rs2814561**	T	C	0.488	0.070	0.016	-0.008	0.018
**rs2839398**	C	G	0.908	-0.094	0.023	-0.049	0.030
**rs28400431**	A	C	0.500	0.064	0.015	-0.029	0.018
**rs28452470**	A	T	0.364	0.074	0.014	-0.006	0.018
**rs28527780**	A	G	0.107	0.096	0.021	0.082	0.027
**rs2859369**	T	C	0.086	-0.103	0.025	0.002	0.030
**rs28633403**	A	G	0.544	0.072	0.014	0.021	0.018
**rs288170**	A	T	0.824	0.083	0.018	0.030	0.022
**rs34825990**	A	T	0.672	-0.060	0.014	-0.062	0.018
**rs35531294**	T	C	0.285	0.074	0.018	0.010	0.021
**rs35880546**	T	C	0.640	0.061	0.014	0.035	0.019
**rs36092443**	A	G	0.044	0.139	0.033	0.009	0.046
**rs368761619**	C	G	0.017	0.254	0.053	0.111	0.069

**rs3782399**	A	G	0.939	0.131	0.030	0.013	0.035
**rs4144756**	A	G	0.457	0.077	0.015	0.002	0.018
**rs4275621**	A	G	0.628	0.073	0.014	-0.019	0.017
**rs4293822**	T	G	0.755	-0.071	0.016	-0.017	0.020
**rs4367080**	A	G	0.259	0.071	0.016	0.015	0.021
**rs4489042**	C	G	0.386	-0.059	0.014	-0.002	0.018
**rs4521021**	T	C	0.778	-0.080	0.019	0.013	0.022
**rs4586908**	C	G	0.526	-0.062	0.014	0.010	0.017
**rs4636402**	C	G	0.525	0.062	0.014	-0.006	0.017
**rs4739249**	A	C	0.822	0.082	0.018	-0.015	0.023
**rs4770610**	T	C	0.462	-0.058	0.014	0.006	0.017
**rs4839923**	A	G	0.435	0.065	0.014	0.002	0.017
**rs4858241**	T	G	0.618	0.082	0.014	0.005	0.018
**rs4894783**	A	T	0.375	-0.066	0.014	0.035	0.017
**rs4916723**	A	C	0.570	-0.078	0.014	0.005	0.017
**rs4947694**	A	C	0.110	-0.095	0.022	0.003	0.028
**rs55748262**	A	G	0.176	-0.085	0.018	-0.023	0.023
**rs55871549**	A	G	0.098	0.115	0.028	0.017	0.031
**rs56028527**	A	G	0.105	-0.097	0.023	-0.019	0.030
**rs57349798**	A	G	0.408	-0.066	0.015	0.015	0.018
**rs5771783**	T	C	0.416	0.064	0.014	0.000	0.017
**rs6032660**	A	G	0.759	-0.068	0.016	-0.071	0.019
**rs605921**	A	G	0.488	0.060	0.015	0.009	0.018
**rs6063848**	T	G	0.646	0.077	0.015	0.005	0.018
**rs60798171**	T	G	0.768	-0.078	0.016	-0.013	0.020
**rs61836972**	T	C	0.146	0.086	0.019	-0.023	0.024
**rs61842708**	A	C	0.934	-0.121	0.028	0.004	0.032
**rs62018635**	A	G	0.165	0.077	0.018	0.016	0.022
**rs62106688**	A	G	0.138	0.083	0.019	0.011	0.024
**rs62259516**	T	C	0.064	0.141	0.029	-0.042	0.036
**rs62260755**	C	G	0.771	-0.080	0.016	0.055	0.021
**rs62451450**	A	G	0.096	0.095	0.023	0.030	0.027
**rs6451675**	C	G	0.325	-0.073	0.015	-0.009	0.018
**rs6473035**	A	C	0.124	-0.087	0.021	-0.013	0.025
**rs652050**	A	G	0.291	0.061	0.015	-0.020	0.019
**rs6549873**	A	G	0.283	0.061	0.015	-0.035	0.018
**rs66931513**	A	G	0.689	-0.065	0.015	-0.027	0.019
**rs6770301**	A	G	0.226	0.070	0.016	0.031	0.020
**rs6809441**	T	C	0.683	0.061	0.015	0.002	0.018
**rs6812406**	A	T	0.535	-0.061	0.014	0.001	0.017
**rs6933023**	T	C	0.430	0.069	0.014	0.001	0.017
**rs696825**	T	C	0.263	-0.064	0.015	0.011	0.019
**rs7014426**	A	G	0.527	-0.062	0.015	0.028	0.017
**rs7087891**	A	T	0.375	0.070	0.014	-0.012	0.017
**rs713240**	T	C	0.524	0.063	0.014	-0.003	0.017

**rs71466470**	A	G	0.064	-0.128	0.030	-0.032	0.036
**rs7167767**	A	G	0.645	0.060	0.014	0.018	0.018
**rs72673548**	T	C	0.902	-0.116	0.024	-0.017	0.032
**rs72693213**	T	C	0.968	-0.171	0.042	-0.095	0.065
**rs72706977**	T	C	0.957	-0.155	0.034	-0.031	0.045
**rs7277470**	A	T	0.415	0.065	0.014	0.011	0.017
**rs72875959**	T	C	0.902	-0.095	0.023	-0.030	0.027
**rs72881889**	T	C	0.026	0.187	0.042	0.019	0.064
**rs72891531**	T	C	0.203	-0.078	0.019	0.016	0.023
**rs72971564**	T	C	0.231	0.065	0.016	0.014	0.020
**rs7330802**	A	G	0.362	0.070	0.014	0.003	0.018
**rs74129883**	A	C	0.073	-0.142	0.035	-0.096	0.041
**rs7459616**	C	G	0.424	0.065	0.014	-0.004	0.017
**rs74635476**	A	G	0.127	-0.090	0.022	-0.001	0.027
**rs74760947**	A	G	0.956	-0.180	0.032	-0.031	0.043
**rs75263467**	A	G	0.048	0.159	0.036	0.087	0.043
**rs756354**	C	G	0.599	-0.072	0.014	0.025	0.017
**rs7579744**	A	G	0.208	-0.075	0.017	-0.003	0.021
**rs7581057**	A	G	0.933	0.114	0.028	0.052	0.032
**rs761626**	A	G	0.115	-0.090	0.022	-0.028	0.025
**rs7634587**	A	G	0.601	0.065	0.014	-0.024	0.017
**rs77216804**	A	T	0.879	0.104	0.022	-0.031	0.025
**rs7733142**	A	C	0.789	0.074	0.017	0.002	0.020
**rs7824062**	A	G	0.428	0.059	0.014	0.011	0.017
**rs78305268**	A	G	0.022	0.212	0.046	0.067	0.063
**rs79221481**	T	C	0.029	0.197	0.045	0.004	0.058
**rs7930199**	T	C	0.961	-0.174	0.036	0.014	0.046
**rs80286484**	A	G	0.202	0.071	0.017	0.009	0.022
**rs8039398**	T	C	0.550	-0.080	0.014	0.006	0.017
**rs8058358**	T	C	0.490	-0.067	0.014	0.012	0.018
**rs8058677**	T	C	0.371	0.069	0.014	-0.029	0.017
**rs9502319**	C	G	0.644	-0.060	0.014	-0.028	0.018
**rs9547144**	T	C	0.223	-0.082	0.020	-0.007	0.022
**rs9553291**	T	C	0.080	0.119	0.025	0.019	0.031
**rs9559946**	A	C	0.151	-0.079	0.019	0.012	0.023
**rs9577464**	T	G	0.403	-0.068	0.016	-0.018	0.020
**rs9591003**	T	C	0.510	0.059	0.014	0.027	0.017
**rs9596789**	C	G	0.044	-0.157	0.034	0.064	0.040
**rs9661242**	A	G	0.525	-0.062	0.014	0.009	0.017
**rs9674999**	A	G	0.600	0.058	0.014	-0.006	0.018
**rs9678906**	T	C	0.230	-0.073	0.017	0.025	0.021
**rs9903088**	T	C	0.696	-0.059	0.015	-0.008	0.018
**rs992936**	T	C	0.483	0.072	0.014	-0.007	0.017
**rs9959583**	T	C	0.454	-0.060	0.014	-0.035	0.017

Table S34. Raw data for the MR analysis of the causal effect of ADHD on CD (P<5E-5)							
SNP	Effect allele	Other allele	Effect allele frequency	Beta.exposure	Se.exposure	Beta.outcome	Se.outcome
**rs10024525**	T	C	0.811	0.075	0.017	-0.020	0.030
**rs10256728**	A	T	0.303	-0.064	0.015	-0.001	0.025
**rs10262192**	A	G	0.473	0.074	0.014	-0.014	0.023
**rs10400419**	T	C	0.476	-0.067	0.015	0.017	0.023
**rs10512379**	A	G	0.087	0.107	0.025	0.002	0.037
**rs10759922**	C	G	0.564	-0.061	0.015	0.006	0.024
**rs1077612**	T	C	0.217	-0.076	0.017	0.054	0.029
**rs10862707**	A	C	0.145	0.097	0.022	0.016	0.035
**rs10965173**	A	G	0.759	0.079	0.017	0.069	0.030
**rs111900779**	A	G	0.075	0.105	0.025	0.012	0.042
**rs112097663**	T	C	0.163	0.080	0.019	-0.026	0.034
**rs11245604**	A	G	0.109	0.133	0.026	0.042	0.039
**rs112736460**	T	G	0.935	-0.116	0.028	-0.089	0.053
**rs112788138**	T	G	0.102	-0.094	0.023	0.039	0.037
**rs115881829**	C	G	0.944	0.129	0.032	0.034	0.064
**rs11591402**	A	T	0.224	-0.092	0.016	-0.043	0.028
**rs11682489**	T	C	0.625	0.066	0.014	0.027	0.029
**rs11698378**	T	C	0.808	-0.085	0.018	-0.016	0.030
**rs117624174**	T	C	0.059	-0.142	0.031	-0.006	0.054
**rs11773992**	C	G	0.173	0.076	0.018	-0.019	0.031
**rs11782371**	A	G	0.387	-0.059	0.014	0.040	0.024
**rs11813307**	A	G	0.480	-0.057	0.014	-0.032	0.024
**rs11936939**	T	C	0.379	-0.061	0.014	0.031	0.024
**rs11954003**	T	C	0.903	-0.100	0.024	-0.015	0.040
**rs12293450**	T	C	0.207	0.074	0.017	0.040	0.029
**rs12435486**	A	G	0.249	0.066	0.016	-0.059	0.027
**rs1246287**	A	G	0.569	0.062	0.014	0.004	0.024
**rs12475235**	A	G	0.874	-0.082	0.020	-0.029	0.035
**rs1255536**	A	G	0.258	0.071	0.015	-0.012	0.026
**rs1272878**	T	C	0.767	0.091	0.018	0.023	0.029
**rs1286138**	T	G	0.318	0.063	0.014	-0.022	0.024
**rs13023832**	A	G	0.104	0.115	0.022	0.041	0.038
**rs1319859**	A	G	0.666	0.060	0.014	0.000	0.025
**rs13240946**	T	C	0.483	0.064	0.016	-0.022	0.025
**rs1335762**	T	C	0.597	-0.058	0.014	0.033	0.024
**rs13418985**	A	G	0.982	-0.216	0.050	-0.020	0.083
**rs139383262**	A	G	0.017	0.265	0.058	-0.004	0.107
**rs141547796**	A	G	0.085	-0.137	0.026	0.019	0.043
**rs1427829**	A	G	0.436	0.082	0.014	-0.005	0.023
**rs143129347**	T	C	0.070	0.125	0.028	-0.071	0.044
**rs1431592**	T	C	0.306	0.068	0.015	-0.016	0.024
**rs143912172**	A	C	0.935	0.138	0.029	-0.032	0.051

**rs144104266**	A	G	0.035	0.164	0.038	-0.081	0.067
**rs146130170**	A	G	0.744	0.069	0.016	-0.030	0.027
**rs146523543**	A	G	0.020	0.207	0.050	-0.084	0.094
**rs147631443**	A	G	0.412	0.058	0.014	0.004	0.024
**rs147657937**	A	G	0.027	0.197	0.046	-0.009	0.067
**rs1484144**	T	C	0.449	0.064	0.014	0.013	0.023
**rs1513155**	A	G	0.846	-0.085	0.019	0.015	0.031
**rs1592757**	C	G	0.353	0.075	0.014	-0.040	0.025
**rs17018790**	A	T	0.861	0.088	0.020	-0.004	0.034
**rs17084312**	T	C	0.041	0.179	0.034	0.019	0.063
**rs17531412**	A	G	0.692	0.105	0.015	-0.008	0.025
**rs17636214**	A	G	0.034	-0.197	0.041	0.031	0.060
**rs17652815**	T	C	0.956	-0.146	0.032	-0.112	0.056
**rs17718640**	C	G	0.321	-0.063	0.015	-0.003	0.024
**rs1791794**	A	G	0.666	-0.069	0.015	-0.054	0.025
**rs180822580**	A	G	0.051	-0.157	0.033	-0.120	0.055
**rs1833322**	A	T	0.355	0.060	0.014	-0.001	0.024
**rs1844070**	A	T	0.098	-0.103	0.024	-0.032	0.042
**rs185160613**	T	C	0.036	-0.195	0.042	0.044	0.067
**rs1859057**	C	G	0.474	0.063	0.014	-0.005	0.023
**rs1886753**	T	C	0.389	0.056	0.014	0.019	0.024
**rs1959848**	T	C	0.516	-0.070	0.014	0.008	0.023
**rs1978102**	T	C	0.529	0.059	0.014	-0.013	0.023
**rs2090317**	A	G	0.335	0.063	0.014	0.002	0.024
**rs2110042**	A	C	0.594	-0.060	0.014	-0.004	0.024
**rs2113808**	A	G	0.647	0.062	0.015	0.021	0.025
**rs212178**	A	G	0.884	-0.117	0.021	0.040	0.037
**rs2144782**	T	C	0.378	0.071	0.015	-0.021	0.025
**rs2254448**	C	G	0.210	0.070	0.016	0.058	0.028
**rs227378**	A	C	0.666	0.079	0.015	0.007	0.026
**rs2300861**	T	C	0.541	-0.062	0.014	0.013	0.024
**rs2302793**	A	G	0.551	0.061	0.014	-0.018	0.023
**rs2814561**	T	C	0.488	0.070	0.016	-0.027	0.025
**rs2839398**	C	G	0.908	-0.094	0.023	0.002	0.041
**rs28400431**	A	C	0.500	0.064	0.015	-0.046	0.024
**rs28452470**	A	T	0.364	0.074	0.014	-0.001	0.024
**rs28527780**	A	G	0.107	0.096	0.021	0.079	0.036
**rs2859369**	T	C	0.086	-0.103	0.025	-0.044	0.042
**rs28633403**	A	G	0.544	0.072	0.014	0.002	0.024
**rs288170**	A	T	0.824	0.083	0.018	0.047	0.031
**rs34825990**	A	T	0.672	-0.060	0.014	-0.059	0.025
**rs35531294**	T	C	0.285	0.074	0.018	-0.005	0.031
**rs35880546**	T	C	0.640	0.061	0.014	0.062	0.026
**rs36092443**	A	G	0.044	0.139	0.033	0.125	0.064
**rs368761619**	C	G	0.017	0.254	0.053	0.139	0.091

**rs3782399**	A	G	0.939	0.131	0.030	0.011	0.049
**rs4144756**	A	G	0.457	0.077	0.015	-0.015	0.024
**rs4275621**	A	G	0.628	0.073	0.014	-0.008	0.023
**rs4293822**	T	G	0.755	-0.071	0.016	0.026	0.027
**rs4367080**	A	G	0.259	0.071	0.016	0.049	0.028
**rs4489042**	C	G	0.386	-0.059	0.014	-0.011	0.024
**rs4521021**	T	C	0.778	-0.080	0.019	0.023	0.031
**rs4586908**	C	G	0.526	-0.062	0.014	0.017	0.023
**rs4636402**	C	G	0.525	0.062	0.014	-0.004	0.023
**rs4739249**	A	C	0.822	0.082	0.018	-0.008	0.031
**rs4770610**	T	C	0.462	-0.058	0.014	-0.007	0.023
**rs4839923**	A	G	0.435	0.065	0.014	0.024	0.023
**rs4858241**	T	G	0.618	0.082	0.014	0.000	0.024
**rs4894783**	A	T	0.375	-0.066	0.014	0.070	0.024
**rs4916723**	A	C	0.570	-0.078	0.014	-0.025	0.024
**rs4947694**	A	C	0.110	-0.095	0.022	0.017	0.039
**rs55748262**	A	G	0.176	-0.085	0.018	-0.064	0.033
**rs55871549**	A	G	0.098	0.115	0.028	-0.017	0.043
**rs56028527**	A	G	0.105	-0.097	0.023	-0.005	0.040
**rs57349798**	A	G	0.408	-0.066	0.015	0.009	0.024
**rs5771783**	T	C	0.416	0.064	0.014	0.010	0.024
**rs6032660**	A	G	0.759	-0.068	0.016	-0.086	0.026
**rs605921**	A	G	0.488	0.060	0.015	-0.016	0.024
**rs6063848**	T	G	0.646	0.077	0.015	0.024	0.025
**rs60798171**	T	G	0.768	-0.078	0.016	0.004	0.027
**rs61836972**	T	C	0.146	0.086	0.019	0.018	0.033
**rs61842708**	A	C	0.934	-0.121	0.028	0.047	0.044
**rs62018635**	A	G	0.165	0.077	0.018	0.013	0.029
**rs62106688**	A	G	0.138	0.083	0.019	0.026	0.033
**rs62259516**	T	C	0.064	0.141	0.029	-0.035	0.049
**rs62260755**	C	G	0.771	-0.080	0.016	0.029	0.029
**rs62451450**	A	G	0.096	0.095	0.023	0.043	0.037
**rs6451675**	C	G	0.325	-0.073	0.015	0.005	0.025
**rs6473035**	A	C	0.124	-0.087	0.021	0.015	0.034
**rs652050**	A	G	0.291	0.061	0.015	-0.011	0.025
**rs6549873**	A	G	0.283	0.061	0.015	-0.013	0.025
**rs66931513**	A	G	0.689	-0.065	0.015	-0.007	0.025
**rs6770301**	A	G	0.226	0.070	0.016	0.029	0.027
**rs6809441**	T	C	0.683	0.061	0.015	0.000	0.025
**rs6812406**	A	T	0.535	-0.061	0.014	-0.018	0.023
**rs6933023**	T	C	0.430	0.069	0.014	-0.001	0.023
**rs696825**	T	C	0.263	-0.064	0.015	0.031	0.026
**rs7014426**	A	G	0.527	-0.062	0.015	0.026	0.024
**rs7087891**	A	T	0.375	0.070	0.014	-0.029	0.023
**rs713240**	T	C	0.524	0.063	0.014	0.024	0.023

**rs71466470**	A	G	0.064	-0.128	0.030	0.000	0.047
**rs7167767**	A	G	0.645	0.060	0.014	0.046	0.024
**rs72673548**	T	C	0.902	-0.116	0.024	0.037	0.045
**rs72693213**	T	C	0.968	-0.171	0.042	0.010	0.090
**rs72706977**	T	C	0.957	-0.155	0.034	-0.023	0.063
**rs7277470**	A	T	0.415	0.065	0.014	0.029	0.023
**rs72875959**	T	C	0.902	-0.095	0.023	-0.013	0.037
**rs72881889**	T	C	0.026	0.187	0.042	-0.028	0.091
**rs72891531**	T	C	0.203	-0.078	0.019	0.021	0.031
**rs72971564**	T	C	0.231	0.065	0.016	0.038	0.028
**rs7330802**	A	G	0.362	0.070	0.014	0.019	0.024
**rs74129883**	A	C	0.073	-0.142	0.035	-0.089	0.056
**rs7459616**	C	G	0.424	0.065	0.014	-0.014	0.023
**rs74635476**	A	G	0.127	-0.090	0.022	-0.031	0.038
**rs74760947**	A	G	0.956	-0.180	0.032	-0.061	0.061
**rs75263467**	A	G	0.048	0.159	0.036	0.098	0.057
**rs756354**	C	G	0.599	-0.072	0.014	0.055	0.024
**rs7579744**	A	G	0.208	-0.075	0.017	0.000	0.029
**rs7581057**	A	G	0.933	0.114	0.028	0.036	0.042
**rs761626**	A	G	0.115	-0.090	0.022	-0.011	0.034
**rs7634587**	A	G	0.601	0.065	0.014	-0.041	0.023
**rs77216804**	A	T	0.879	0.104	0.022	-0.005	0.035
**rs7733142**	A	C	0.789	0.074	0.017	-0.002	0.027
**rs7824062**	A	G	0.428	0.059	0.014	0.008	0.024
**rs78305268**	A	G	0.022	0.212	0.046	0.122	0.084
**rs79221481**	T	C	0.029	0.197	0.045	-0.001	0.081
**rs7930199**	T	C	0.961	-0.174	0.036	0.037	0.064
**rs80286484**	A	G	0.202	0.071	0.017	-0.001	0.029
**rs8039398**	T	C	0.550	-0.080	0.014	0.011	0.023
**rs8058358**	T	C	0.490	-0.067	0.014	0.024	0.024
**rs8058677**	T	C	0.371	0.069	0.014	-0.073	0.024
**rs9502319**	C	G	0.644	-0.060	0.014	-0.001	0.024
**rs9547144**	T	C	0.223	-0.082	0.020	0.006	0.029
**rs9553291**	T	C	0.080	0.119	0.025	0.028	0.042
**rs9559946**	A	C	0.151	-0.079	0.019	-0.004	0.031
**rs9577464**	T	G	0.403	-0.068	0.016	0.003	0.027
**rs9591003**	T	C	0.510	0.059	0.014	0.038	0.023
**rs9596789**	C	G	0.044	-0.157	0.034	0.058	0.054
**rs9661242**	A	G	0.525	-0.062	0.014	0.002	0.023
**rs9674999**	A	G	0.600	0.058	0.014	-0.012	0.025
**rs9678906**	T	C	0.230	-0.073	0.017	-0.009	0.029
**rs9903088**	T	C	0.696	-0.059	0.015	-0.008	0.025
**rs992936**	T	C	0.483	0.072	0.014	0.037	0.024
**rs9959583**	T	C	0.454	-0.060	0.014	-0.046	0.023

Table S35. Raw data for the MR analysis of the causal effect of ADHD on UC (P<5E-5)							
SNP	Effect allele	Other allele	Effect allele frequency	Beta.exposure	Se.exposure	Beta.outcome	Se.outcome
**rs10024525**	T	C	0.811	0.075	0.017	0.039	0.028
**rs10256728**	A	T	0.303	-0.064	0.015	-0.014	0.023
**rs10262192**	A	G	0.473	0.074	0.014	-0.033	0.022
**rs10400419**	T	C	0.476	-0.067	0.015	0.013	0.021
**rs10512379**	A	G	0.087	0.107	0.025	-0.023	0.036
**rs10759922**	C	G	0.564	-0.061	0.015	-0.034	0.022
**rs1077612**	T	C	0.217	-0.076	0.017	0.045	0.027
**rs10862707**	A	C	0.145	0.097	0.022	0.037	0.031
**rs10965173**	A	G	0.759	0.079	0.017	0.004	0.028
**rs111900779**	A	G	0.075	0.105	0.025	0.022	0.039
**rs112097663**	T	C	0.163	0.080	0.019	-0.046	0.030
**rs11245604**	A	G	0.109	0.133	0.026	0.015	0.037
**rs112736460**	T	G	0.935	-0.116	0.028	0.000	0.046
**rs112788138**	T	G	0.102	-0.094	0.023	-0.011	0.035
**rs115881829**	C	G	0.944	0.129	0.032	-0.042	0.053
**rs11591402**	A	T	0.224	-0.092	0.016	0.050	0.025
**rs11682489**	T	C	0.625	0.066	0.014	-0.038	0.026
**rs11698378**	T	C	0.808	-0.085	0.018	0.012	0.029
**rs117624174**	T	C	0.059	-0.142	0.031	-0.009	0.049
**rs11773992**	C	G	0.173	0.076	0.018	-0.041	0.028
**rs11782371**	A	G	0.387	-0.059	0.014	0.012	0.022
**rs11813307**	A	G	0.480	-0.057	0.014	-0.011	0.022
**rs11936939**	T	C	0.379	-0.061	0.014	-0.007	0.022
**rs11954003**	T	C	0.903	-0.100	0.024	0.010	0.037
**rs12293450**	T	C	0.207	0.074	0.017	0.028	0.027
**rs12435486**	A	G	0.249	0.066	0.016	-0.029	0.025
**rs1246287**	A	G	0.569	0.062	0.014	-0.020	0.022
**rs12475235**	A	G	0.874	-0.082	0.020	0.030	0.032
**rs1255536**	A	G	0.258	0.071	0.015	0.027	0.024
**rs1272878**	T	C	0.767	0.091	0.018	0.003	0.027
**rs1286138**	T	G	0.318	0.063	0.014	-0.004	0.023
**rs13023832**	A	G	0.104	0.115	0.022	0.055	0.035
**rs1319859**	A	G	0.666	0.060	0.014	0.017	0.023
**rs13240946**	T	C	0.483	0.064	0.016	0.025	0.024
**rs1335762**	T	C	0.597	-0.058	0.014	-0.026	0.022
**rs13418985**	A	G	0.982	-0.216	0.050	-0.077	0.078
**rs139383262**	A	G	0.017	0.265	0.058	-0.124	0.100
**rs141547796**	A	G	0.085	-0.137	0.026	-0.035	0.040
**rs1427829**	A	G	0.436	0.082	0.014	-0.024	0.021
**rs143129347**	T	C	0.070	0.125	0.028	-0.035	0.042
**rs1431592**	T	C	0.306	0.068	0.015	-0.018	0.023
**rs143912172**	A	C	0.935	0.138	0.029	0.067	0.047

**rs144104266**	A	G	0.035	0.164	0.038	0.070	0.058
**rs146130170**	A	G	0.744	0.069	0.016	0.017	0.025
**rs146523543**	A	G	0.020	0.207	0.050	-0.040	0.081
**rs147631443**	A	G	0.412	0.058	0.014	-0.001	0.022
**rs147657937**	A	G	0.027	0.197	0.046	0.003	0.064
**rs1484144**	T	C	0.449	0.064	0.014	0.031	0.021
**rs1513155**	A	G	0.846	-0.085	0.019	-0.057	0.029
**rs1592757**	C	G	0.353	0.075	0.014	0.027	0.022
**rs17018790**	A	T	0.861	0.088	0.020	-0.021	0.031
**rs17084312**	T	C	0.041	0.179	0.034	0.042	0.058
**rs17531412**	A	G	0.692	0.105	0.015	0.029	0.023
**rs17636214**	A	G	0.034	-0.197	0.041	0.056	0.058
**rs17652815**	T	C	0.956	-0.146	0.032	0.012	0.055
**rs17718640**	C	G	0.321	-0.063	0.015	-0.042	0.022
**rs1791794**	A	G	0.666	-0.069	0.015	-0.048	0.023
**rs180822580**	A	G	0.051	-0.157	0.033	0.022	0.051
**rs1833322**	A	T	0.355	0.060	0.014	0.011	0.022
**rs1844070**	A	T	0.098	-0.103	0.024	0.049	0.038
**rs185160613**	T	C	0.036	-0.195	0.042	0.055	0.063
**rs1859057**	C	G	0.474	0.063	0.014	-0.006	0.021
**rs1886753**	T	C	0.389	0.056	0.014	-0.013	0.022
**rs1959848**	T	C	0.516	-0.070	0.014	0.025	0.022
**rs1978102**	T	C	0.529	0.059	0.014	0.006	0.021
**rs2090317**	A	G	0.335	0.063	0.014	0.006	0.023
**rs2110042**	A	C	0.594	-0.060	0.014	0.001	0.022
**rs2113808**	A	G	0.647	0.062	0.015	0.035	0.023
**rs212178**	A	G	0.884	-0.117	0.021	-0.020	0.034
**rs2144782**	T	C	0.378	0.071	0.015	-0.027	0.023
**rs2254448**	C	G	0.210	0.070	0.016	0.018	0.026
**rs227378**	A	C	0.666	0.079	0.015	-0.068	0.024
**rs2300861**	T	C	0.541	-0.062	0.014	-0.005	0.022
**rs2302793**	A	G	0.551	0.061	0.014	-0.029	0.021
**rs2814561**	T	C	0.488	0.070	0.016	-0.003	0.023
**rs2839398**	C	G	0.908	-0.094	0.023	-0.096	0.037
**rs28400431**	A	C	0.500	0.064	0.015	-0.022	0.022
**rs28452470**	A	T	0.364	0.074	0.014	-0.007	0.022
**rs28527780**	A	G	0.107	0.096	0.021	0.081	0.034
**rs2859369**	T	C	0.086	-0.103	0.025	0.027	0.038
**rs28633403**	A	G	0.544	0.072	0.014	0.045	0.022
**rs288170**	A	T	0.824	0.083	0.018	-0.003	0.028
**rs34825990**	A	T	0.672	-0.060	0.014	-0.069	0.023
**rs35531294**	T	C	0.285	0.074	0.018	-0.010	0.025
**rs35880546**	T	C	0.640	0.061	0.014	0.021	0.023
**rs36092443**	A	G	0.044	0.139	0.033	-0.058	0.058
**rs368761619**	C	G	0.017	0.254	0.053	0.102	0.087

**rs3782399**	A	G	0.939	0.131	0.030	0.003	0.044
**rs4144756**	A	G	0.457	0.077	0.015	0.004	0.022
**rs4275621**	A	G	0.628	0.073	0.014	-0.019	0.022
**rs4293822**	T	G	0.755	-0.071	0.016	-0.043	0.025
**rs4367080**	A	G	0.259	0.071	0.016	-0.015	0.026
**rs4489042**	C	G	0.386	-0.059	0.014	-0.007	0.022
**rs4521021**	T	C	0.778	-0.080	0.019	0.005	0.027
**rs4586908**	C	G	0.526	-0.062	0.014	0.000	0.021
**rs4636402**	C	G	0.525	0.062	0.014	-0.002	0.021
**rs4739249**	A	C	0.822	0.082	0.018	-0.028	0.028
**rs4770610**	T	C	0.462	-0.058	0.014	0.013	0.021
**rs4839923**	A	G	0.435	0.065	0.014	-0.025	0.021
**rs4858241**	T	G	0.618	0.082	0.014	0.027	0.022
**rs4894783**	A	T	0.375	-0.066	0.014	0.016	0.022
**rs4916723**	A	C	0.570	-0.078	0.014	0.027	0.022
**rs4947694**	A	C	0.110	-0.095	0.022	0.017	0.035
**rs55748262**	A	G	0.176	-0.085	0.018	0.013	0.029
**rs55871549**	A	G	0.098	0.115	0.028	0.033	0.038
**rs56028527**	A	G	0.105	-0.097	0.023	-0.027	0.037
**rs57349798**	A	G	0.408	-0.066	0.015	0.021	0.022
**rs5771783**	T	C	0.416	0.064	0.014	-0.016	0.022
**rs6032660**	A	G	0.759	-0.068	0.016	-0.054	0.024
**rs605921**	A	G	0.488	0.060	0.015	0.022	0.023
**rs6063848**	T	G	0.646	0.077	0.015	-0.016	0.023
**rs60798171**	T	G	0.768	-0.078	0.016	-0.013	0.025
**rs61836972**	T	C	0.146	0.086	0.019	-0.032	0.031
**rs61842708**	A	C	0.934	-0.121	0.028	-0.026	0.041
**rs62018635**	A	G	0.165	0.077	0.018	0.013	0.028
**rs62106688**	A	G	0.138	0.083	0.019	0.002	0.031
**rs62259516**	T	C	0.064	0.141	0.029	-0.011	0.045
**rs62260755**	C	G	0.771	-0.080	0.016	0.071	0.027
**rs62451450**	A	G	0.096	0.095	0.023	0.026	0.034
**rs6451675**	C	G	0.325	-0.073	0.015	-0.023	0.023
**rs6473035**	A	C	0.124	-0.087	0.021	-0.035	0.032
**rs652050**	A	G	0.291	0.061	0.015	-0.036	0.024
**rs6549873**	A	G	0.283	0.061	0.015	-0.057	0.023
**rs66931513**	A	G	0.689	-0.065	0.015	-0.035	0.023
**rs6770301**	A	G	0.226	0.070	0.016	0.052	0.025
**rs6809441**	T	C	0.683	0.061	0.015	0.007	0.023
**rs6812406**	A	T	0.535	-0.061	0.014	0.015	0.021
**rs6933023**	T	C	0.430	0.069	0.014	0.009	0.021
**rs696825**	T	C	0.263	-0.064	0.015	-0.010	0.024
**rs7014426**	A	G	0.527	-0.062	0.015	0.031	0.022
**rs7087891**	A	T	0.375	0.070	0.014	-0.006	0.022
**rs713240**	T	C	0.524	0.063	0.014	-0.016	0.021

**rs71466470**	A	G	0.064	-0.128	0.030	-0.051	0.047
**rs7167767**	A	G	0.645	0.060	0.014	0.001	0.022
**rs72673548**	T	C	0.902	-0.116	0.024	-0.043	0.039
**rs72693213**	T	C	0.968	-0.171	0.042	-0.153	0.080
**rs72706977**	T	C	0.957	-0.155	0.034	-0.043	0.056
**rs7277470**	A	T	0.415	0.065	0.014	0.001	0.021
**rs72875959**	T	C	0.902	-0.095	0.023	-0.065	0.033
**rs72881889**	T	C	0.026	0.187	0.042	0.075	0.078
**rs72891531**	T	C	0.203	-0.078	0.019	0.010	0.029
**rs72971564**	T	C	0.231	0.065	0.016	0.003	0.025
**rs7330802**	A	G	0.362	0.070	0.014	-0.008	0.022
**rs74129883**	A	C	0.073	-0.142	0.035	-0.108	0.051
**rs7459616**	C	G	0.424	0.065	0.014	0.010	0.021
**rs74635476**	A	G	0.127	-0.090	0.022	0.033	0.034
**rs74760947**	A	G	0.956	-0.180	0.032	-0.024	0.054
**rs75263467**	A	G	0.048	0.159	0.036	0.066	0.056
**rs756354**	C	G	0.599	-0.072	0.014	0.008	0.022
**rs7579744**	A	G	0.208	-0.075	0.017	-0.005	0.027
**rs7581057**	A	G	0.933	0.114	0.028	0.052	0.040
**rs761626**	A	G	0.115	-0.090	0.022	-0.040	0.032
**rs7634587**	A	G	0.601	0.065	0.014	-0.018	0.022
**rs77216804**	A	T	0.879	0.104	0.022	-0.043	0.031
**rs7733142**	A	C	0.789	0.074	0.017	-0.001	0.026
**rs7824062**	A	G	0.428	0.059	0.014	0.014	0.022
**rs78305268**	A	G	0.022	0.212	0.046	0.030	0.079
**rs79221481**	T	C	0.029	0.197	0.045	-0.008	0.072
**rs7930199**	T	C	0.961	-0.174	0.036	0.001	0.057
**rs80286484**	A	G	0.202	0.071	0.017	0.002	0.027
**rs8039398**	T	C	0.550	-0.080	0.014	-0.003	0.021
**rs8058358**	T	C	0.490	-0.067	0.014	0.006	0.022
**rs8058677**	T	C	0.371	0.069	0.014	0.004	0.022
**rs9502319**	C	G	0.644	-0.060	0.014	-0.043	0.022
**rs9547144**	T	C	0.223	-0.082	0.020	-0.018	0.027
**rs9553291**	T	C	0.080	0.119	0.025	0.022	0.039
**rs9559946**	A	C	0.151	-0.079	0.019	0.013	0.029
**rs9577464**	T	G	0.403	-0.068	0.016	-0.030	0.026
**rs9591003**	T	C	0.510	0.059	0.014	0.016	0.021
**rs9596789**	C	G	0.044	-0.157	0.034	0.082	0.050
**rs9661242**	A	G	0.525	-0.062	0.014	0.020	0.022
**rs9674999**	A	G	0.600	0.058	0.014	-0.010	0.022
**rs9678906**	T	C	0.230	-0.073	0.017	0.041	0.027
**rs9903088**	T	C	0.696	-0.059	0.015	-0.005	0.023
**rs992936**	T	C	0.483	0.072	0.014	-0.027	0.022
**rs9959583**	T	C	0.454	-0.060	0.014	-0.027	0.021

Table S36. Raw data for the MR analysis of the causal effect of anxiety disorder on IBD (P<5E-5)							
**SNP**	**Effect allele**	**Other allele**	**Effect allele frequency**	**Beta.exposure**	**Se.exposure**	**Beta.outcome**	**Se.outcome**
**rs10104302**	T	G	0.736	-0.122	0.03	0	0.019
**rs10485029**	A	G	0.514	-0.146	0.032	0.005	0.017
**rs10766527**	A	G	0.872	0.2	0.045	0.002	0.025
**rs10814991**	T	C	0.392	-0.166	0.037	-0.009	0.019
**rs111711662**	T	C	0.93	-0.516	0.125	0.013	0.042
**rs112205280**	A	G	0.594	0.353	0.072	0.001	0.026
**rs11253200**	T	C	0.529	-0.112	0.028	-0.016	0.017
**rs117069908**	T	G	0.901	0.305	0.075	0.025	0.034
**rs11734058**	T	C	0.616	-0.167	0.039	-0.005	0.019
**rs117439037**	A	G	0.94	0.338	0.08	0.051	0.039
**rs11917582**	A	G	0.067	1.052	0.251	0.002	0.121
**rs11946789**	C	G	0.868	0.172	0.041	0.01	0.026
**rs12314784**	T	C	0.62	-0.117	0.027	-0.018	0.017
**rs12457088**	A	T	0.277	-0.14	0.033	-0.027	0.019
**rs12493813**	A	G	0.693	-0.126	0.031	0.012	0.018
**rs12508492**	T	C	0.932	0.309	0.076	-0.023	0.037
**rs12628414**	A	G	0.858	-0.206	0.05	0.004	0.026
**rs13045716**	C	G	0.532	-0.125	0.03	0.016	0.017
**rs13056278**	T	C	0.145	-0.174	0.042	-0.01	0.025
**rs13158658**	T	C	0.648	0.113	0.028	-0.011	0.018
**rs13334730**	A	C	0.885	-0.17	0.042	-0.049	0.028
**rs13335305**	A	G	0.441	0.154	0.035	-0.003	0.017
**rs1400184**	A	G	0.093	0.215	0.052	-0.003	0.032
**rs150551**	A	G	0.698	-0.119	0.029	-0.02	0.018
**rs1609480**	T	C	0.434	0.125	0.029	-0.005	0.017
**rs16963010**	T	C	0.164	0.176	0.039	0.003	0.023
**rs16983533**	A	G	0.279	0.148	0.035	-0.009	0.02
**rs1709393**	T	C	0.579	-0.151	0.027	0.002	0.017
**rs17164125**	T	C	0.911	0.213	0.052	-0.011	0.028
**rs17357773**	T	C	0.182	0.146	0.034	-0.01	0.022
**rs17368264**	C	G	0.738	0.135	0.033	-0.011	0.019
**rs1741594**	T	C	0.118	-0.283	0.067	-0.053	0.03
**rs17469573**	T	C	0.691	0.12	0.029	-0.013	0.018
**rs186491271**	A	G	0.2	-0.239	0.058	-0.044	0.027
**rs1869251**	A	G	0.539	-0.122	0.029	0.006	0.017
**rs1881540**	T	G	0.555	-0.112	0.027	-0.008	0.017
**rs2027114**	A	G	0.483	0.108	0.026	-0.009	0.017
**rs2124973**	T	C	0.94	-0.456	0.1	0.027	0.043
**rs2146346**	A	G	0.587	0.144	0.03	0.003	0.017
**rs2396595**	T	C	0.47	-0.111	0.027	-0.004	0.017
**rs2740360**	T	C	0.474	0.17	0.033	-0.003	0.018
**rs2753188**	A	G	0.727	0.16	0.033	0	0.019

**rs28373923**	A	G	0.068	0.419	0.092	-0.01	0.042
**rs34166097**	A	G	0.052	0.466	0.113	0.083	0.052
**rs35149381**	A	G	0.89	-0.173	0.043	-0.004	0.027
**rs353585**	A	G	0.815	0.18	0.044	-0.025	0.024
**rs35367883**	T	C	0.06	0.348	0.083	0.035	0.04
**rs356407**	A	C	0.305	0.139	0.031	0.004	0.018
**rs4254910**	A	C	0.787	-0.143	0.034	0.05	0.021
**rs55824216**	A	G	0.058	0.473	0.112	0.01	0.041
**rs56070849**	T	C	0.882	0.21	0.046	0.034	0.029
**rs58990403**	A	G	0.792	-0.185	0.039	-0.056	0.023
**rs60602867**	C	G	0.068	0.263	0.063	-0.02	0.035
**rs6122698**	T	C	0.585	0.123	0.028	0.024	0.018
**rs61605057**	T	G	0.938	0.317	0.076	-0.079	0.037
**rs62062388**	T	C	0.13	0.21	0.051	0.031	0.028
**rs62113566**	T	C	0.071	-0.399	0.097	0.009	0.037
**rs62574777**	T	C	0.284	0.132	0.032	-0.049	0.019
**rs6450387**	A	T	0.301	-0.152	0.036	-0.002	0.018
**rs6536734**	A	G	0.548	-0.115	0.027	-0.038	0.017
**rs6601080**	A	G	0.665	-0.128	0.028	0.04	0.018
**rs6742479**	T	G	0.828	-0.163	0.038	0.022	0.023
**rs6774302**	A	G	0.118	-0.255	0.057	0.067	0.03
**rs6887011**	A	C	0.372	0.123	0.028	0.01	0.017
**rs702227**	A	T	0.117	0.202	0.048	-0.003	0.026
**rs7022987**	T	C	0.301	0.123	0.029	0	0.019
**rs7195882**	T	C	0.428	-0.116	0.027	0.002	0.017
**rs72817576**	A	T	0.055	-0.485	0.108	0.031	0.039
**rs72839861**	A	C	0.949	0.727	0.165	-0.048	0.038
**rs739315**	A	G	0.569	-0.154	0.033	0.011	0.018
**rs74402545**	A	C	0.892	-0.199	0.046	-0.009	0.027
**rs7639522**	A	G	0.731	-0.149	0.035	0	0.019
**rs77506340**	T	C	0.097	0.233	0.057	0.013	0.031
**rs7813971**	T	C	0.404	0.119	0.027	0.001	0.017
**rs797494**	C	G	0.756	0.145	0.035	-0.023	0.02
**rs7979878**	A	G	0.587	-0.115	0.028	0.025	0.017
**rs800078**	A	T	0.537	0.126	0.029	-0.039	0.017
**rs80241045**	C	G	0.71	-0.363	0.084	0.012	0.029
**rs874707**	T	C	0.483	-0.141	0.032	0.026	0.018
**rs878321**	A	G	0.684	0.153	0.035	0.009	0.018
**rs9297727**	A	G	0.907	0.201	0.047	0.006	0.029
**rs9519381**	A	G	0.454	-0.133	0.033	0.054	0.017
**rs9749185**	A	G	0.113	0.475	0.112	0.015	0.029
**rs9975524**	A	G	0.216	0.214	0.049	0.012	0.024

Table S37. Raw data for the MR analysis of the causal effect of anxiety disorder on CD (P<5E-5)							
SNP	Effect allele	Other allele	Effect allele frequency	Beta.exposure	Se.exposure	Beta.outcome	Se.outcome
**rs10104302**	T	G	0.736	-0.122	0.03	-0.001	0.026
**rs10485029**	A	G	0.514	-0.146	0.032	-0.006	0.023
**rs10766527**	A	G	0.872	0.2	0.045	-0.03	0.033
**rs10814991**	T	C	0.392	-0.166	0.037	0	0.025
**rs111711662**	T	C	0.93	-0.516	0.125	0.009	0.059
**rs112205280**	A	G	0.594	0.353	0.072	-0.025	0.038
**rs11253200**	T	C	0.529	-0.112	0.028	0.021	0.023
**rs117069908**	T	G	0.901	0.305	0.075	0.031	0.048
**rs11734058**	T	C	0.616	-0.167	0.039	-0.015	0.026
**rs117439037**	A	G	0.94	0.338	0.08	0.06	0.054
**rs11917582**	A	G	0.067	1.052	0.251	0.01	0.153
**rs11946789**	C	G	0.868	0.172	0.041	-0.031	0.035
**rs12314784**	T	C	0.62	-0.117	0.027	0.028	0.023
**rs12457088**	A	T	0.277	-0.14	0.033	-0.002	0.025
**rs12493813**	A	G	0.693	-0.126	0.031	0.022	0.025
**rs12508492**	T	C	0.932	0.309	0.076	-0.115	0.049
**rs12628414**	A	G	0.858	-0.206	0.05	0.021	0.036
**rs13045716**	C	G	0.532	-0.125	0.03	0.052	0.023
**rs13056278**	T	C	0.145	-0.174	0.042	0.012	0.034
**rs13158658**	T	C	0.648	0.113	0.028	-0.001	0.024
**rs13334730**	A	C	0.885	-0.17	0.042	-0.034	0.038
**rs13335305**	A	G	0.441	0.154	0.035	0.003	0.024
**rs1400184**	A	G	0.093	0.215	0.052	-0.031	0.045
**rs150551**	A	G	0.698	-0.119	0.029	-0.023	0.025
**rs1609480**	T	C	0.434	0.125	0.029	0.019	0.023
**rs16963010**	T	C	0.164	0.176	0.039	0.015	0.031
**rs16983533**	A	G	0.279	0.148	0.035	-0.074	0.028
**rs1709393**	T	C	0.579	-0.151	0.027	-0.024	0.023
**rs17164125**	T	C	0.911	0.213	0.052	0.073	0.038
**rs17357773**	T	C	0.182	0.146	0.034	-0.011	0.03
**rs17368264**	C	G	0.738	0.135	0.033	-0.035	0.026
**rs1741594**	T	C	0.118	-0.283	0.067	-0.084	0.041
**rs17469573**	T	C	0.691	0.12	0.029	0.004	0.025
**rs186491271**	A	G	0.2	-0.239	0.058	-0.018	0.037
**rs1869251**	A	G	0.539	-0.122	0.029	0.018	0.023
**rs1881540**	T	G	0.555	-0.112	0.027	0.01	0.024
**rs2027114**	A	G	0.483	0.108	0.026	0.003	0.023
**rs2124973**	T	C	0.94	-0.456	0.1	0.036	0.061
**rs2146346**	A	G	0.587	0.144	0.03	0.01	0.023
**rs2396595**	T	C	0.47	-0.111	0.027	-0.019	0.023
**rs2740360**	T	C	0.474	0.17	0.033	-0.01	0.025
**rs2753188**	A	G	0.727	0.16	0.033	0.058	0.026

**rs28373923**	A	G	0.068	0.419	0.092	-0.032	0.058
**rs34166097**	A	G	0.052	0.466	0.113	0.046	0.072
**rs35149381**	A	G	0.89	-0.173	0.043	0.001	0.037
**rs353585**	A	G	0.815	0.18	0.044	0.012	0.032
**rs35367883**	T	C	0.06	0.348	0.083	0.101	0.053
**rs356407**	A	C	0.305	0.139	0.031	0.021	0.025
**rs4254910**	A	C	0.787	-0.143	0.034	0.048	0.028
**rs55824216**	A	G	0.058	0.473	0.112	0.02	0.058
**rs56070849**	T	C	0.882	0.21	0.046	-0.013	0.038
**rs58990403**	A	G	0.792	-0.185	0.039	-0.035	0.032
**rs60602867**	C	G	0.068	0.263	0.063	-0.075	0.049
**rs6122698**	T	C	0.585	0.123	0.028	0.024	0.024
**rs61605057**	T	G	0.938	0.317	0.076	-0.131	0.049
**rs62062388**	T	C	0.13	0.21	0.051	-0.017	0.038
**rs62113566**	T	C	0.071	-0.399	0.097	-0.01	0.049
**rs62574777**	T	C	0.284	0.132	0.032	-0.063	0.026
**rs6450387**	A	T	0.301	-0.152	0.036	0.018	0.025
**rs6536734**	A	G	0.548	-0.115	0.027	-0.011	0.024
**rs6601080**	A	G	0.665	-0.128	0.028	0.022	0.024
**rs6742479**	T	G	0.828	-0.163	0.038	0.045	0.031
**rs6774302**	A	G	0.118	-0.255	0.057	0.02	0.041
**rs6887011**	A	C	0.372	0.123	0.028	-0.015	0.024
**rs702227**	A	T	0.117	0.202	0.048	-0.008	0.035
**rs7022987**	T	C	0.301	0.123	0.029	0.031	0.025
**rs7195882**	T	C	0.428	-0.116	0.027	0.031	0.024
**rs72817576**	A	T	0.055	-0.485	0.108	0.017	0.053
**rs72839861**	A	C	0.949	0.727	0.165	-0.108	0.05
**rs739315**	A	G	0.569	-0.154	0.033	-0.003	0.026
**rs74402545**	A	C	0.892	-0.199	0.046	-0.064	0.036
**rs7639522**	A	G	0.731	-0.149	0.035	0.011	0.026
**rs77506340**	T	C	0.097	0.233	0.057	0.067	0.041
**rs7813971**	T	C	0.404	0.119	0.027	-0.002	0.023
**rs797494**	C	G	0.756	0.145	0.035	-0.019	0.027
**rs7979878**	A	G	0.587	-0.115	0.028	0.048	0.024
**rs800078**	A	T	0.537	0.126	0.029	-0.015	0.023
**rs80241045**	C	G	0.71	-0.363	0.084	0.075	0.041
**rs874707**	T	C	0.483	-0.141	0.032	-0.02	0.024
**rs878321**	A	G	0.684	0.153	0.035	-0.017	0.024
**rs9297727**	A	G	0.907	0.201	0.047	0.066	0.04
**rs9519381**	A	G	0.454	-0.133	0.033	0.04	0.024
**rs9749185**	A	G	0.113	0.475	0.112	0.014	0.039
**rs9975524**	A	G	0.216	0.214	0.049	-0.03	0.032

Table S38. Raw data for the MR analysis of the causal effect of anxiety disorder on UC (P<5E-5)							
SNP	Effect allele	Other allele	Effect allele frequency	Beta.exposure	Se.exposure	Beta.outcome	Se.outcome
**rs10104302**	T	G	0.736	-0.122	0.03	-0.006	0.024
**rs10485029**	A	G	0.514	-0.146	0.032	0.009	0.021
**rs10766527**	A	G	0.872	0.2	0.045	0.011	0.031
**rs10814991**	T	C	0.392	-0.166	0.037	-0.015	0.024
**rs111711662**	T	C	0.93	-0.516	0.125	0.005	0.052
**rs112205280**	A	G	0.594	0.353	0.072	0.015	0.03
**rs11253200**	T	C	0.529	-0.112	0.028	-0.04	0.021
**rs117069908**	T	G	0.901	0.305	0.075	0.016	0.043
**rs11734058**	T	C	0.616	-0.167	0.039	-0.006	0.024
**rs117439037**	A	G	0.94	0.338	0.08	0.035	0.048
**rs11917582**	A	G	0.067	1.052	0.251	0.069	0.161
**rs11946789**	C	G	0.868	0.172	0.041	0.04	0.033
**rs12314784**	T	C	0.62	-0.117	0.027	-0.044	0.022
**rs12457088**	A	T	0.277	-0.14	0.033	-0.043	0.024
**rs12493813**	A	G	0.693	-0.126	0.031	0.01	0.023
**rs12508492**	T	C	0.932	0.309	0.076	0.066	0.048
**rs12628414**	A	G	0.858	-0.206	0.05	-0.019	0.033
**rs13045716**	C	G	0.532	-0.125	0.03	-0.012	0.022
**rs13056278**	T	C	0.145	-0.174	0.042	-0.018	0.032
**rs13158658**	T	C	0.648	0.113	0.028	-0.021	0.022
**rs13334730**	A	C	0.885	-0.17	0.042	-0.062	0.035
**rs13335305**	A	G	0.441	0.154	0.035	-0.017	0.022
**rs1400184**	A	G	0.093	0.215	0.052	0.041	0.039
**rs150551**	A	G	0.698	-0.119	0.029	-0.03	0.023
**rs1609480**	T	C	0.434	0.125	0.029	-0.007	0.021
**rs16963010**	T	C	0.164	0.176	0.039	-0.014	0.029
**rs16983533**	A	G	0.279	0.148	0.035	0.023	0.024
**rs1709393**	T	C	0.579	-0.151	0.027	0.023	0.022
**rs17164125**	T	C	0.911	0.213	0.052	-0.03	0.035
**rs17357773**	T	C	0.182	0.146	0.034	-0.014	0.027
**rs17368264**	C	G	0.738	0.135	0.033	0.014	0.024
**rs1741594**	T	C	0.118	-0.283	0.067	-0.022	0.037
**rs17469573**	T	C	0.691	0.12	0.029	-0.018	0.023
**rs186491271**	A	G	0.2	-0.239	0.058	-0.069	0.034
**rs1869251**	A	G	0.539	-0.122	0.029	-0.015	0.021
**rs1881540**	T	G	0.555	-0.112	0.027	-0.024	0.022
**rs2027114**	A	G	0.483	0.108	0.026	-0.017	0.021
**rs2124973**	T	C	0.94	-0.456	0.1	0.046	0.054
**rs2146346**	A	G	0.587	0.144	0.03	-0.002	0.021
**rs2396595**	T	C	0.47	-0.111	0.027	0.022	0.021
**rs2740360**	T	C	0.474	0.17	0.033	0.009	0.023
**rs2753188**	A	G	0.727	0.16	0.033	-0.038	0.024

**rs28373923**	A	G	0.068	0.419	0.092	0.039	0.052
**rs34166097**	A	G	0.052	0.466	0.113	0.089	0.063
**rs35149381**	A	G	0.89	-0.173	0.043	0.013	0.034
**rs353585**	A	G	0.815	0.18	0.044	-0.048	0.03
**rs35367883**	T	C	0.06	0.348	0.083	-0.004	0.051
**rs356407**	A	C	0.305	0.139	0.031	-0.015	0.023
**rs4254910**	A	C	0.787	-0.143	0.034	0.027	0.026
**rs55824216**	A	G	0.058	0.473	0.112	0.001	0.051
**rs56070849**	T	C	0.882	0.21	0.046	0.057	0.037
**rs58990403**	A	G	0.792	-0.185	0.039	-0.085	0.029
**rs60602867**	C	G	0.068	0.263	0.063	-0.015	0.044
**rs6122698**	T	C	0.585	0.123	0.028	0.015	0.022
**rs61605057**	T	G	0.938	0.317	0.076	-0.043	0.047
**rs62062388**	T	C	0.13	0.21	0.051	0.047	0.035
**rs62113566**	T	C	0.071	-0.399	0.097	0.044	0.048
**rs62574777**	T	C	0.284	0.132	0.032	-0.035	0.024
**rs6450387**	A	T	0.301	-0.152	0.036	-0.01	0.023
**rs6536734**	A	G	0.548	-0.115	0.027	-0.051	0.022
**rs6601080**	A	G	0.665	-0.128	0.028	0.056	0.023
**rs6742479**	T	G	0.828	-0.163	0.038	0.011	0.029
**rs6774302**	A	G	0.118	-0.255	0.057	0.068	0.037
**rs6887011**	A	C	0.372	0.123	0.028	0.02	0.022
**rs702227**	A	T	0.117	0.202	0.048	0.004	0.032
**rs7022987**	T	C	0.301	0.123	0.029	-0.027	0.023
**rs7195882**	T	C	0.428	-0.116	0.027	-0.026	0.022
**rs72817576**	A	T	0.055	-0.485	0.108	0.052	0.049
**rs72839861**	A	C	0.949	0.727	0.165	-0.02	0.05
**rs739315**	A	G	0.569	-0.154	0.033	0.015	0.022
**rs74402545**	A	C	0.892	-0.199	0.046	0.032	0.034
**rs7639522**	A	G	0.731	-0.149	0.035	-0.001	0.024
**rs77506340**	T	C	0.097	0.233	0.057	-0.022	0.039
**rs7813971**	T	C	0.404	0.119	0.027	-0.003	0.022
**rs797494**	C	G	0.756	0.145	0.035	-0.02	0.025
**rs7979878**	A	G	0.587	-0.115	0.028	0.015	0.022
**rs800078**	A	T	0.537	0.126	0.029	-0.058	0.021
**rs80241045**	C	G	0.71	-0.363	0.084	-0.026	0.035
**rs874707**	T	C	0.483	-0.141	0.032	0.046	0.022
**rs878321**	A	G	0.684	0.153	0.035	0.029	0.023
**rs9297727**	A	G	0.907	0.201	0.047	-0.049	0.036
**rs9519381**	A	G	0.454	-0.133	0.033	0.071	0.022
**rs9749185**	A	G	0.113	0.475	0.112	0	0.036
**rs9975524**	A	G	0.216	0.214	0.049	0.051	0.031

Table S39. Raw data for the MR analysis of the causal effect of ASD on IBD (P<5E-5)							
**SNP**	**Effect allele**	**Other allele**	**Effect allele frequency**	**Beta.exposure**	**Se.exposure**	**Beta.outcome**	**Se.outcome**
**rs10099100**	C	G	0.346	0.084	0.015	-0.007	0.018
**rs10110094**	A	G	0.146	0.091	0.019	0.038	0.024
**rs10254161**	C	G	0.039	0.133	0.032	0.034	0.052
**rs10264235**	A	G	0.921	0.113	0.027	-0.005	0.031
**rs10481273**	T	C	0.523	-0.056	0.014	0.012	0.017
**rs1086528**	T	C	0.099	0.118	0.029	-0.045	0.031
**rs10897995**	A	G	0.803	0.07	0.017	0.02	0.021
**rs111363571**	A	G	0.053	-0.144	0.035	0.015	0.045
**rs11159344**	T	C	0.33	0.059	0.015	0.006	0.018
**rs11185408**	A	G	0.488	-0.069	0.014	-0.014	0.017
**rs111931861**	A	G	0.93	-0.217	0.041	0.022	0.038
**rs112635299**	T	G	0.017	0.221	0.043	0.169	0.067
**rs113003385**	A	G	0.056	0.133	0.029	0.072	0.041
**rs113764414**	A	G	0.704	0.073	0.016	-0.003	0.021
**rs115738686**	T	G	0.058	0.137	0.032	0.014	0.038
**rs115833252**	T	C	0.024	0.233	0.052	0.081	0.056
**rs116346488**	A	G	0.051	0.143	0.032	0.038	0.039
**rs116977567**	T	G	0.977	-0.196	0.043	0.077	0.067
**rs11707386**	A	G	0.291	-0.066	0.015	0	0.018
**rs11750781**	T	C	0.761	-0.069	0.016	0.02	0.02
**rs11787216**	T	C	0.358	-0.069	0.015	-0.022	0.018
**rs11872054**	T	C	0.108	-0.1	0.023	0.013	0.028
**rs12203328**	C	G	0.275	0.07	0.015	0.026	0.019
**rs12225635**	A	T	0.085	0.119	0.027	-0.012	0.033
**rs12518453**	A	C	0.931	-0.109	0.026	0.012	0.04
**rs12543498**	T	C	0.747	-0.065	0.016	-0.003	0.019
**rs12733548**	A	G	0.198	-0.082	0.02	-0.016	0.022
**rs12761401**	A	G	0.21	0.074	0.017	-0.027	0.022
**rs12772374**	A	G	0.827	-0.08	0.019	-0.058	0.022
**rs12917054**	T	C	0.219	-0.071	0.017	-0.003	0.021
**rs12974837**	A	C	0.17	-0.077	0.019	0.025	0.023
**rs13086752**	T	C	0.016	0.263	0.062	0.008	0.082
**rs1317345**	A	G	0.587	-0.062	0.015	-0.007	0.018
**rs13201465**	A	G	0.983	-0.282	0.062	0.021	0.072
**rs13397126**	A	C	0.7	0.066	0.015	0.008	0.018
**rs1357273**	T	C	0.539	0.06	0.014	-0.032	0.017
**rs141319505**	A	G	0.979	0.291	0.061	0.001	0.073
**rs141455452**	T	G	0.553	0.078	0.016	0.008	0.025
**rs141735644**	T	C	0.018	0.232	0.053	-0.068	0.069
**rs144911765**	T	C	0.974	-0.19	0.04	-0.155	0.061
**rs1452075**	T	C	0.721	0.081	0.016	-0.026	0.019
**rs146176468**	A	G	0.965	-0.179	0.041	-0.003	0.047

**rs146369513**	A	G	0.019	0.235	0.058	0.063	0.073
**rs147866472**	T	C	0.896	0.096	0.024	0.012	0.031
**rs148587110**	T	C	0.982	-0.33	0.074	0.063	0.072
**rs149923766**	T	G	0.978	-0.237	0.048	-0.014	0.059
**rs150055584**	T	G	0.958	-0.141	0.033	-0.032	0.042
**rs151249695**	A	T	0.948	-0.138	0.033	-0.108	0.039
**rs1522603**	T	C	0.535	0.064	0.014	-0.008	0.017
**rs1548635**	A	T	0.535	0.062	0.014	-0.026	0.017
**rs16879023**	A	G	0.144	-0.096	0.02	-0.001	0.024
**rs16933101**	A	G	0.043	-0.153	0.035	-0.029	0.043
**rs17496928**	T	C	0.079	-0.112	0.027	-0.015	0.034
**rs17517971**	A	G	0.063	0.141	0.032	0.027	0.038
**rs17755501**	T	C	0.861	-0.091	0.021	-0.033	0.025
**rs17831585**	C	G	0.666	0.061	0.015	0.021	0.018
**rs1986735**	T	C	0.11	-0.098	0.024	0.028	0.028
**rs2164224**	A	G	0.447	0.066	0.016	0.013	0.018
**rs2224274**	T	C	0.492	0.071	0.014	-0.008	0.017
**rs2259481**	A	G	0.796	-0.076	0.017	-0.037	0.021
**rs2270037**	T	C	0.311	0.064	0.015	0.029	0.018
**rs227375**	T	C	0.491	-0.058	0.014	0.017	0.017
**rs2304465**	A	G	0.413	-0.061	0.014	0.001	0.017
**rs2391769**	A	G	0.347	-0.077	0.015	0.011	0.018
**rs2419842**	C	G	0.586	-0.059	0.014	-0.01	0.017
**rs2613259**	A	C	0.261	-0.067	0.016	0.053	0.019
**rs2782502**	C	G	0.913	-0.11	0.027	-0.001	0.031
**rs28364433**	A	G	0.032	-0.158	0.038	0.027	0.049
**rs28495892**	A	G	0.804	0.076	0.018	-0.001	0.022
**rs285901**	T	C	0.151	-0.087	0.02	-0.022	0.024
**rs28729902**	A	G	0.81	-0.084	0.018	-0.009	0.022
**rs292441**	A	G	0.665	-0.072	0.015	0.044	0.018
**rs325485**	A	G	0.399	0.073	0.014	-0.005	0.017
**rs34509057**	A	G	0.227	0.073	0.017	-0.011	0.02
**rs35404050**	T	C	0.213	0.084	0.018	-0.016	0.021
**rs35737824**	T	C	0.026	-0.169	0.039	-0.029	0.062
**rs35923829**	A	G	0.062	0.119	0.028	-0.028	0.035
**rs36069412**	A	G	0.02	0.237	0.055	0.163	0.071
**rs36127550**	T	G	0.163	0.084	0.021	-0.005	0.027
**rs41363353**	C	G	0.836	-0.084	0.019	-0.028	0.023
**rs4236681**	T	C	0.219	0.07	0.017	-0.004	0.02
**rs4416511**	A	G	0.871	0.09	0.022	-0.029	0.028
**rs455468**	T	C	0.71	-0.063	0.015	0.028	0.019
**rs45595836**	T	C	0.086	0.139	0.027	-0.003	0.036
**rs4609618**	A	C	0.367	-0.064	0.014	-0.017	0.017
**rs463035**	A	G	0.323	-0.063	0.015	0.018	0.018
**rs4750990**	T	C	0.631	-0.068	0.014	0.015	0.018

**rs4760340**	A	T	0.522	0.057	0.014	-0.001	0.017
**rs4916723**	A	C	0.593	-0.067	0.014	0.005	0.017
**rs529507**	A	G	0.859	-0.091	0.02	0.004	0.025
**rs56305452**	A	C	0.78	-0.069	0.017	0.021	0.021
**rs57134244**	A	G	0.767	-0.067	0.016	-0.001	0.021
**rs58039481**	A	T	0.095	-0.101	0.023	-0.031	0.029
**rs59362641**	A	C	0.88	-0.094	0.023	-0.031	0.027
**rs597040**	C	G	0.019	0.258	0.058	0.048	0.069
**rs62003975**	C	G	0.051	0.131	0.032	0.011	0.043
**rs62170721**	T	G	0.138	-0.091	0.022	0.008	0.025
**rs62331287**	A	C	0.474	0.06	0.014	-0.012	0.017
**rs62444282**	T	G	0.801	-0.077	0.018	0.008	0.022
**rs62587113**	T	C	0.025	0.202	0.047	-0.003	0.058
**rs6422311**	A	G	0.4	-0.06	0.014	-0.021	0.018
**rs6430841**	A	G	0.808	0.08	0.018	-0.016	0.021
**rs644552**	A	G	0.056	0.159	0.035	-0.031	0.037
**rs6464584**	A	G	0.141	-0.087	0.021	-0.002	0.025
**rs6543224**	T	C	0.524	-0.059	0.014	0.011	0.017
**rs6674082**	A	G	0.423	0.059	0.014	-0.007	0.017
**rs6692705**	A	G	0.394	0.066	0.014	0.006	0.017
**rs6701243**	A	C	0.617	0.074	0.014	-0.026	0.018
**rs7186853**	T	C	0.338	0.062	0.015	-0.016	0.018
**rs7258751**	T	C	0.415	-0.062	0.015	0.02	0.018
**rs72831869**	A	G	0.943	-0.111	0.027	-0.03	0.038
**rs72934503**	A	G	0.485	-0.07	0.014	-0.005	0.017
**rs73073015**	A	G	0.107	0.104	0.025	0.126	0.028
**rs73082395**	C	G	0.104	0.09	0.022	0.012	0.028
**rs74439007**	A	G	0.015	0.232	0.057	0.011	0.081
**rs74647376**	T	C	0.975	-0.179	0.044	0.016	0.057
**rs75263467**	A	G	0.053	0.15	0.035	0.087	0.043
**rs75289223**	T	G	0.078	0.108	0.025	0.011	0.031
**rs7573532**	A	G	0.518	0.057	0.014	0.021	0.017
**rs7578456**	A	G	0.412	0.064	0.014	-0.02	0.017
**rs76397219**	A	G	0.924	-0.14	0.03	0.038	0.038
**rs76415301**	A	G	0.115	-0.097	0.023	0.024	0.027
**rs76504400**	A	G	0.166	0.079	0.019	0.003	0.024
**rs77339815**	T	C	0.05	0.147	0.035	-0.004	0.045
**rs77475618**	A	G	0.066	0.132	0.032	-0.027	0.039
**rs77691144**	T	C	0.971	-0.207	0.044	-0.041	0.063
**rs77768281**	A	G	0.057	-0.127	0.03	0.071	0.039
**rs7783557**	T	C	0.646	0.067	0.015	0.028	0.018
**rs78006462**	T	C	0.969	0.173	0.042	0.029	0.064
**rs78058104**	A	G	0.03	0.188	0.04	0.068	0.05
**rs78298487**	T	G	0.985	-0.255	0.058	0.103	0.079
**rs78653484**	T	C	0.035	-0.176	0.039	-0.034	0.049

**rs78769840**	A	T	0.948	-0.129	0.031	0	0.039
**rs78827416**	A	G	0.076	0.131	0.027	0.01	0.035
**rs79940520**	A	G	0.858	-0.095	0.021	-0.008	0.026
**rs910805**	A	G	0.78	-0.096	0.016	0.012	0.021
**rs9366877**	A	G	0.569	0.068	0.014	-0.026	0.017
**rs9386190**	A	G	0.066	-0.119	0.029	0.018	0.038
**rs9389208**	T	C	0.352	0.067	0.014	-0.019	0.018
**rs9530773**	T	G	0.691	-0.064	0.015	-0.003	0.018
**rs9688046**	C	G	0.419	-0.063	0.015	0.003	0.018
**rs976680**	A	T	0.756	-0.07	0.016	0.013	0.02

Table S40. Raw data for the MR analysis of the causal effect of ASD on CD (P<5E-5)							
SNP	Effect allele	Other allele	Effect allele frequency	Beta.exposure	Se.exposure	Beta.outcome	Se.outcome
rs10099100	C	G	0.347	0.084	0.015	-0.049	0.024
rs10110094	A	G	0.147	0.091	0.019	0.031	0.033
rs10254161	C	G	0.041	0.133	0.032	0.098	0.066
rs10264235	A	G	0.921	0.113	0.027	0.044	0.043
rs10481273	T	C	0.517	-0.056	0.014	0.017	0.023
rs1086528	T	C	0.099	0.118	0.029	-0.01	0.042
rs10897995	A	G	0.802	0.07	0.017	0.022	0.029
rs111363571	A	G	0.055	-0.144	0.035	0.026	0.059
rs11159344	T	C	0.328	0.059	0.015	-0.003	0.025
rs11185408	A	G	0.485	-0.069	0.014	-0.006	0.024
rs111931861	A	G	0.93	-0.217	0.041	0.005	0.051
rs112635299	T	G	0.016	0.221	0.043	0.139	0.091
rs113003385	A	G	0.053	0.133	0.029	0.092	0.057
rs113764414	A	G	0.706	0.073	0.016	0.024	0.029
rs115738686	T	G	0.058	0.137	0.032	-0.026	0.053
rs115833252	T	C	0.026	0.233	0.052	0.224	0.072
rs116346488	A	G	0.051	0.143	0.032	0.036	0.052
rs116977567	T	G	0.977	-0.196	0.043	0.064	0.094
rs11707386	A	G	0.289	-0.066	0.015	0.016	0.025
rs11750781	T	C	0.762	-0.069	0.016	0.05	0.027
rs11787216	T	C	0.358	-0.069	0.015	-0.008	0.024
rs11872054	T	C	0.11	-0.1	0.023	-0.015	0.038
rs12203328	C	G	0.273	0.07	0.015	0.035	0.026
rs12225635	A	T	0.085	0.119	0.027	-0.034	0.045
rs12518453	A	C	0.93	-0.109	0.026	-0.001	0.055
rs12543498	T	C	0.748	-0.065	0.016	0.013	0.026
rs12733548	A	G	0.198	-0.082	0.02	-0.018	0.03
rs12761401	A	G	0.206	0.074	0.017	-0.023	0.031
rs12772374	A	G	0.827	-0.08	0.019	-0.053	0.03
rs12917054	T	C	0.222	-0.071	0.017	0.024	0.028
rs12974837	A	C	0.17	-0.077	0.019	0.005	0.031
rs13086752	T	C	0.017	0.263	0.062	0.036	0.108
rs1317345	A	G	0.579	-0.062	0.015	-0.033	0.025
rs13201465	A	G	0.984	-0.282	0.062	0.033	0.105
rs13397126	A	C	0.697	0.066	0.015	0.011	0.025
rs1357273	T	C	0.539	0.06	0.014	-0.018	0.023
rs141319505	A	G	0.978	0.291	0.061	0.098	0.099
rs141455452	T	G	0.559	0.078	0.016	0.001	0.035
rs141735644	T	C	0.017	0.232	0.053	-0.001	0.097
rs144911765	T	C	0.975	-0.19	0.04	-0.175	0.084
rs1452075	T	C	0.721	0.081	0.016	-0.001	0.026
rs146176468	A	G	0.965	-0.179	0.041	-0.005	0.063

**rs146369513**	A	G	0.019	0.235	0.058	0.104	0.097
**rs147866472**	T	C	0.897	0.096	0.024	0.019	0.043
**rs148587110**	T	C	0.982	-0.33	0.074	-0.064	0.095
**rs149923766**	T	G	0.978	-0.237	0.048	-0.116	0.079
**rs150055584**	T	G	0.958	-0.141	0.033	-0.023	0.058
**rs151249695**	A	T	0.947	-0.138	0.033	-0.114	0.053
**rs1522603**	T	C	0.536	0.064	0.014	-0.005	0.023
**rs1548635**	A	T	0.534	0.062	0.014	-0.009	0.023
**rs16879023**	A	G	0.143	-0.096	0.02	-0.028	0.033
**rs16933101**	A	G	0.044	-0.153	0.035	0.007	0.059
**rs17496928**	T	C	0.077	-0.112	0.027	-0.063	0.046
**rs17517971**	A	G	0.062	0.141	0.032	0.104	0.052
**rs17755501**	T	C	0.859	-0.091	0.021	0.001	0.034
**rs17831585**	C	G	0.663	0.061	0.015	0.011	0.024
**rs1986735**	T	C	0.111	-0.098	0.024	0.051	0.038
**rs2164224**	A	G	0.45	0.066	0.016	-0.02	0.026
**rs2224274**	T	C	0.49	0.071	0.014	-0.054	0.023
**rs2259481**	A	G	0.798	-0.076	0.017	-0.048	0.029
**rs2270037**	T	C	0.315	0.064	0.015	0.023	0.025
**rs227375**	T	C	0.487	-0.058	0.014	-0.048	0.023
**rs2304465**	A	G	0.419	-0.061	0.014	0.041	0.023
**rs2391769**	A	G	0.343	-0.077	0.015	0.043	0.024
**rs2419842**	C	G	0.583	-0.059	0.014	-0.001	0.023
**rs2613259**	A	C	0.264	-0.067	0.016	0.073	0.026
**rs2782502**	C	G	0.911	-0.11	0.027	-0.024	0.042
**rs28364433**	A	G	0.032	-0.158	0.038	0.032	0.065
**rs28495892**	A	G	0.806	0.076	0.018	0.021	0.029
**rs285901**	T	C	0.15	-0.087	0.02	-0.01	0.032
**rs28729902**	A	G	0.811	-0.084	0.018	-0.03	0.029
**rs292441**	A	G	0.664	-0.072	0.015	0.058	0.025
**rs325485**	A	G	0.399	0.073	0.014	-0.031	0.024
**rs34509057**	A	G	0.225	0.073	0.017	-0.002	0.028
**rs35404050**	T	C	0.216	0.084	0.018	-0.042	0.028
**rs35737824**	T	C	0.025	-0.169	0.039	0.059	0.086
**rs35923829**	A	G	0.063	0.119	0.028	-0.03	0.047
**rs36069412**	A	G	0.019	0.237	0.055	0.114	0.101
**rs36127550**	T	G	0.168	0.084	0.021	-0.019	0.037
**rs41363353**	C	G	0.835	-0.084	0.019	0	0.031
**rs4236681**	T	C	0.217	0.07	0.017	-0.05	0.028
**rs4416511**	A	G	0.87	0.09	0.022	-0.027	0.037
**rs455468**	T	C	0.713	-0.063	0.015	0.016	0.026
**rs45595836**	T	C	0.088	0.139	0.027	-0.043	0.047
**rs4609618**	A	C	0.364	-0.064	0.014	-0.007	0.024
**rs463035**	A	G	0.323	-0.063	0.015	-0.005	0.024
**rs4750990**	T	C	0.633	-0.068	0.014	-0.011	0.025

**rs4760340**	A	T	0.531	0.057	0.014	0.011	0.023
**rs4916723**	A	C	0.599	-0.067	0.014	-0.025	0.024
**rs529507**	A	G	0.86	-0.091	0.02	-0.005	0.033
**rs56305452**	A	C	0.783	-0.069	0.017	-0.002	0.029
**rs57134244**	A	G	0.769	-0.067	0.016	-0.046	0.028
**rs58039481**	A	T	0.093	-0.101	0.023	0.011	0.04
**rs59362641**	A	C	0.881	-0.094	0.023	-0.001	0.037
**rs597040**	C	G	0.018	0.258	0.058	0.056	0.097
**rs62003975**	C	G	0.052	0.131	0.032	-0.043	0.058
**rs62170721**	T	G	0.138	-0.091	0.022	-0.021	0.034
**rs62331287**	A	C	0.474	0.06	0.014	0.017	0.023
**rs62444282**	T	G	0.788	-0.077	0.018	0.023	0.03
**rs62587113**	T	C	0.026	0.202	0.047	0.008	0.078
**rs6422311**	A	G	0.399	-0.06	0.014	-0.036	0.024
**rs6430841**	A	G	0.806	0.08	0.018	-0.046	0.029
**rs644552**	A	G	0.059	0.159	0.035	-0.025	0.049
**rs6464584**	A	G	0.142	-0.087	0.021	-0.046	0.034
**rs6543224**	T	C	0.522	-0.059	0.014	0.022	0.023
**rs6674082**	A	G	0.423	0.059	0.014	-0.001	0.023
**rs6692705**	A	G	0.389	0.066	0.014	-0.006	0.024
**rs6701243**	A	C	0.616	0.074	0.014	-0.04	0.024
**rs7186853**	T	C	0.341	0.062	0.015	-0.034	0.025
**rs7258751**	T	C	0.417	-0.062	0.015	0.014	0.024
**rs72831869**	A	G	0.945	-0.111	0.027	0.024	0.054
**rs72934503**	A	G	0.517	-0.07	0.014	-0.002	0.023
**rs73073015**	A	G	0.11	0.104	0.025	0.13	0.038
**rs73082395**	C	G	0.103	0.09	0.022	0.071	0.038
**rs74439007**	A	G	0.014	0.232	0.057	-0.007	0.114
**rs74647376**	T	C	0.977	-0.179	0.044	0.022	0.081
**rs75263467**	A	G	0.055	0.15	0.035	0.098	0.057
**rs75289223**	T	G	0.078	0.108	0.025	0.072	0.042
**rs7573532**	A	G	0.519	0.057	0.014	-0.006	0.023
**rs7578456**	A	G	0.414	0.064	0.014	-0.01	0.023
**rs76397219**	A	G	0.923	-0.14	0.03	0.064	0.052
**rs76415301**	A	G	0.115	-0.097	0.023	0.032	0.037
**rs76504400**	A	G	0.166	0.079	0.019	-0.003	0.033
**rs77339815**	T	C	0.049	0.147	0.035	-0.001	0.062
**rs77475618**	A	G	0.064	0.132	0.032	-0.052	0.056
**rs77691144**	T	C	0.972	-0.207	0.044	-0.019	0.088
**rs77768281**	A	G	0.056	-0.127	0.03	0.123	0.052
**rs7783557**	T	C	0.645	0.067	0.015	0.02	0.024
**rs78006462**	T	C	0.971	0.173	0.042	-0.06	0.092
**rs78058104**	A	G	0.03	0.188	0.04	0.02	0.069
**rs78298487**	T	G	0.986	-0.255	0.058	0.095	0.119
**rs78653484**	T	C	0.034	-0.176	0.039	0.041	0.066

**rs78769840**	A	T	0.949	-0.129	0.031	-0.046	0.053
**rs78827416**	A	G	0.075	0.131	0.027	0.008	0.048
**rs79940520**	A	G	0.857	-0.095	0.021	-0.037	0.035
**rs910805**	A	G	0.783	-0.096	0.016	-0.014	0.028
**rs9366877**	A	G	0.572	0.068	0.014	-0.039	0.023
**rs9386190**	A	G	0.066	-0.119	0.029	0.04	0.052
**rs9389208**	T	C	0.351	0.067	0.014	-0.016	0.024
**rs9530773**	T	G	0.69	-0.064	0.015	-0.024	0.025
**rs9688046**	C	G	0.422	-0.063	0.015	0.001	0.024
**rs976680**	A	T	0.762	-0.07	0.016	0.046	0.027

Table S41. Raw data for the MR analysis of the causal effect of ASD on UC (P<5E-5)							
**SNP**	**Effect allele**	**Other allele**	**Effect allele frequency**	**Beta.exposure**	**Se.exposure**	**Beta.outcome**	**Se.outcome**
**rs10099100**	C	G	0.346	0.084	0.015	0.024	0.022
**rs10110094**	A	G	0.145	0.091	0.019	0.014	0.031
**rs10254161**	C	G	0.037	0.133	0.032	-0.005	0.068
**rs10264235**	A	G	0.921	0.113	0.027	-0.048	0.039
**rs10481273**	T	C	0.525	-0.056	0.014	0.003	0.021
**rs1086528**	T	C	0.098	0.118	0.029	-0.041	0.039
**rs10897995**	A	G	0.803	0.07	0.017	0.021	0.027
**rs111363571**	A	G	0.053	-0.144	0.035	0.004	0.057
**rs11159344**	T	C	0.331	0.059	0.015	0.018	0.023
**rs11185408**	A	G	0.489	-0.069	0.014	-0.015	0.022
**rs111931861**	A	G	0.931	-0.217	0.041	0.038	0.048
**rs112635299**	T	G	0.016	0.221	0.043	0.211	0.083
**rs113003385**	A	G	0.057	0.133	0.029	0.039	0.051
**rs113764414**	A	G	0.702	0.073	0.016	-0.023	0.027
**rs115738686**	T	G	0.059	0.137	0.032	0.067	0.047
**rs115833252**	T	C	0.024	0.233	0.052	-0.031	0.074
**rs116346488**	A	G	0.051	0.143	0.032	0.048	0.048
**rs116977567**	T	G	0.976	-0.196	0.043	0.104	0.083
**rs11707386**	A	G	0.291	-0.066	0.015	-0.006	0.023
**rs11750781**	T	C	0.759	-0.069	0.016	0.006	0.025
**rs11787216**	T	C	0.359	-0.069	0.015	-0.032	0.023
**rs11872054**	T	C	0.108	-0.1	0.023	0.018	0.035
**rs12203328**	C	G	0.276	0.07	0.015	0.031	0.024
**rs12225635**	A	T	0.084	0.119	0.027	0.007	0.041
**rs12518453**	A	C	0.932	-0.109	0.026	-0.021	0.05
**rs12543498**	T	C	0.748	-0.065	0.016	-0.026	0.025
**rs12733548**	A	G	0.199	-0.082	0.02	-0.015	0.028
**rs12761401**	A	G	0.212	0.074	0.017	-0.024	0.028
**rs12772374**	A	G	0.827	-0.08	0.019	-0.059	0.028
**rs12917054**	T	C	0.218	-0.071	0.017	-0.026	0.026
**rs12974837**	A	C	0.171	-0.077	0.019	0.043	0.029
**rs13086752**	T	C	0.015	0.263	0.062	-0.058	0.109
**rs1317345**	A	G	0.591	-0.062	0.015	0.019	0.023
**rs13201465**	A	G	0.983	-0.282	0.062	-0.024	0.088
**rs13397126**	A	C	0.702	0.066	0.015	-0.002	0.023
**rs1357273**	T	C	0.54	0.06	0.014	-0.039	0.021
**rs141319505**	A	G	0.979	0.291	0.061	-0.106	0.092
**rs141455452**	T	G	0.549	0.078	0.016	0	0.032
**rs141735644**	T	C	0.018	0.232	0.053	-0.117	0.086
**rs144911765**	T	C	0.974	-0.19	0.04	-0.168	0.076
**rs1452075**	T	C	0.722	0.081	0.016	-0.047	0.024
**rs146176468**	A	G	0.966	-0.179	0.041	0.041	0.06

**rs146369513**	A	G	0.018	0.235	0.058	0.007	0.093
**rs147866472**	T	C	0.895	0.096	0.024	-0.009	0.039
**rs148587110**	T	C	0.982	-0.33	0.074	0.108	0.093
**rs149923766**	T	G	0.978	-0.237	0.048	0.075	0.076
**rs150055584**	T	G	0.958	-0.141	0.033	-0.042	0.053
**rs151249695**	A	T	0.949	-0.138	0.033	-0.126	0.05
**rs1522603**	T	C	0.534	0.064	0.014	-0.014	0.021
**rs1548635**	A	T	0.537	0.062	0.014	-0.028	0.021
**rs16879023**	A	G	0.145	-0.096	0.02	0.02	0.03
**rs16933101**	A	G	0.043	-0.153	0.035	-0.041	0.054
**rs17496928**	T	C	0.08	-0.112	0.027	0.025	0.042
**rs17517971**	A	G	0.063	0.141	0.032	-0.04	0.048
**rs17755501**	T	C	0.86	-0.091	0.021	-0.065	0.032
**rs17831585**	C	G	0.667	0.061	0.015	0.03	0.022
**rs1986735**	T	C	0.11	-0.098	0.024	0.027	0.035
**rs2164224**	A	G	0.445	0.066	0.016	0.033	0.022
**rs2224274**	T	C	0.494	0.071	0.014	0.029	0.021
**rs2259481**	A	G	0.798	-0.076	0.017	-0.032	0.027
**rs2270037**	T	C	0.308	0.064	0.015	0.024	0.023
**rs227375**	T	C	0.493	-0.058	0.014	0.067	0.021
**rs2304465**	A	G	0.41	-0.061	0.014	-0.026	0.022
**rs2391769**	A	G	0.347	-0.077	0.015	-0.016	0.022
**rs2419842**	C	G	0.586	-0.059	0.014	-0.014	0.021
**rs2613259**	A	C	0.258	-0.067	0.016	0.033	0.024
**rs2782502**	C	G	0.914	-0.11	0.027	0.025	0.038
**rs28364433**	A	G	0.031	-0.158	0.038	-0.011	0.062
**rs28495892**	A	G	0.803	0.076	0.018	-0.016	0.027
**rs285901**	T	C	0.15	-0.087	0.02	-0.024	0.03
**rs28729902**	A	G	0.81	-0.084	0.018	-0.026	0.027
**rs292441**	A	G	0.665	-0.072	0.015	0.03	0.023
**rs325485**	A	G	0.4	0.073	0.014	0.013	0.022
**rs34509057**	A	G	0.227	0.073	0.017	-0.013	0.025
**rs35404050**	T	C	0.213	0.084	0.018	0.011	0.026
**rs35737824**	T	C	0.026	-0.169	0.039	-0.094	0.078
**rs35923829**	A	G	0.062	0.119	0.028	-0.015	0.044
**rs36069412**	A	G	0.021	0.237	0.055	0.152	0.086
**rs36127550**	T	G	0.161	0.084	0.021	0.028	0.035
**rs41363353**	C	G	0.837	-0.084	0.019	-0.046	0.028
**rs4236681**	T	C	0.22	0.07	0.017	0.005	0.026
**rs4416511**	A	G	0.872	0.09	0.022	-0.03	0.035
**rs455468**	T	C	0.709	-0.063	0.015	0.029	0.024
**rs45595836**	T	C	0.084	0.139	0.027	0.025	0.047
**rs4609618**	A	C	0.369	-0.064	0.014	-0.023	0.022
**rs463035**	A	G	0.323	-0.063	0.015	0.031	0.023
**rs4750990**	T	C	0.63	-0.068	0.014	0.032	0.023

**rs4760340**	A	T	0.518	0.057	0.014	0.003	0.022
**rs4916723**	A	C	0.593	-0.067	0.014	0.027	0.022
**rs529507**	A	G	0.858	-0.091	0.02	0.011	0.031
**rs56305452**	A	C	0.78	-0.069	0.017	0.03	0.027
**rs57134244**	A	G	0.767	-0.067	0.016	0.031	0.026
**rs58039481**	A	T	0.095	-0.101	0.023	-0.065	0.037
**rs59362641**	A	C	0.88	-0.094	0.023	-0.048	0.034
**rs597040**	C	G	0.018	0.258	0.058	0.164	0.085
**rs62003975**	C	G	0.051	0.131	0.032	0.047	0.054
**rs62170721**	T	G	0.14	-0.091	0.022	0.036	0.03
**rs62331287**	A	C	0.473	0.06	0.014	-0.031	0.021
**rs62444282**	T	G	0.806	-0.077	0.018	0.017	0.028
**rs62587113**	T	C	0.025	0.202	0.047	0.041	0.072
**rs6422311**	A	G	0.402	-0.06	0.014	0.007	0.023
**rs6430841**	A	G	0.809	0.08	0.018	0.004	0.027
**rs644552**	A	G	0.054	0.159	0.035	-0.002	0.048
**rs6464584**	A	G	0.141	-0.087	0.021	0.041	0.031
**rs6543224**	T	C	0.522	-0.059	0.014	0.015	0.021
**rs6674082**	A	G	0.422	0.059	0.014	-0.013	0.022
**rs6692705**	A	G	0.396	0.066	0.014	0.034	0.022
**rs6701243**	A	C	0.618	0.074	0.014	-0.029	0.022
**rs7186853**	T	C	0.338	0.062	0.015	-0.006	0.023
**rs7258751**	T	C	0.415	-0.062	0.015	0.037	0.023
**rs72831869**	A	G	0.941	-0.111	0.027	-0.052	0.047
**rs72934503**	A	G	0.519	-0.07	0.014	0.013	0.021
**rs73073015**	A	G	0.104	0.104	0.025	0.129	0.035
**rs73082395**	C	G	0.103	0.09	0.022	-0.043	0.035
**rs74439007**	A	G	0.016	0.232	0.057	0.064	0.1
**rs74647376**	T	C	0.974	-0.179	0.044	0.013	0.07
**rs75263467**	A	G	0.053	0.15	0.035	0.066	0.056
**rs75289223**	T	G	0.078	0.108	0.025	-0.034	0.04
**rs7573532**	A	G	0.518	0.057	0.014	0.043	0.021
**rs7578456**	A	G	0.411	0.064	0.014	-0.017	0.021
**rs76397219**	A	G	0.925	-0.14	0.03	-0.007	0.048
**rs76415301**	A	G	0.115	-0.097	0.023	0.013	0.034
**rs76504400**	A	G	0.169	0.079	0.019	0.002	0.03
**rs77339815**	T	C	0.051	0.147	0.035	0.01	0.055
**rs77475618**	A	G	0.067	0.132	0.032	-0.028	0.047
**rs77691144**	T	C	0.971	-0.207	0.044	-0.053	0.078
**rs77768281**	A	G	0.056	-0.127	0.03	0.027	0.049
**rs7783557**	T	C	0.645	0.067	0.015	0.032	0.022
**rs78006462**	T	C	0.968	0.173	0.042	0.077	0.079
**rs78058104**	A	G	0.03	0.188	0.04	0.085	0.062
**rs78298487**	T	G	0.985	-0.255	0.058	0.026	0.096
**rs78653484**	T	C	0.035	-0.176	0.039	-0.066	0.062

**rs78769840**	A	T	0.947	-0.129	0.031	0.02	0.05
**rs78827416**	A	G	0.076	0.131	0.027	0.015	0.044
**rs79940520**	A	G	0.859	-0.095	0.021	0.009	0.033
**rs910805**	A	G	0.779	-0.096	0.016	0.02	0.026
**rs9366877**	A	G	0.569	0.068	0.014	-0.023	0.021
**rs9386190**	A	G	0.066	-0.119	0.029	0.042	0.048
**rs9389208**	T	C	0.352	0.067	0.014	-0.022	0.022
**rs9530773**	T	G	0.691	-0.064	0.015	0.01	0.023
**rs9688046**	C	G	0.417	-0.063	0.015	0.008	0.023
**rs976680**	A	T	0.752	-0.07	0.016	-0.007	0.025

Table S42 Raw data for the MR analysis of the causal effect of BD on IBD (P<5E-5)							
SNP	Effect allele	Other allele	Effect allele frequency	Beta.exposure	Se.exposure	Beta.outcome	Se.outcome
**rs10035722**	C	A	0.944	-0.099	0.023	-0.025	0.042
**rs10043984**	C	T	0.734	-0.059	0.011	-0.020	0.019
**rs10131905**	C	A	0.597	0.048	0.010	0.007	0.017
**rs10237578**	G	C	0.231	-0.050	0.012	-0.012	0.021
**rs10255167**	G	A	0.229	-0.066	0.012	0.013	0.020
**rs1027453**	T	C	0.498	-0.042	0.010	-0.030	0.017
**rs1032292**	A	C	0.829	0.057	0.013	0.054	0.023
**rs1033291**	A	T	0.436	-0.040	0.009	-0.019	0.017
**rs10412427**	T	C	0.814	0.059	0.013	-0.012	0.023
**rs10455979**	C	G	0.523	-0.056	0.010	-0.021	0.017
**rs10505139**	A	G	0.832	-0.051	0.012	-0.015	0.022
**rs10520294**	C	T	0.845	0.056	0.013	-0.009	0.023
**rs10737496**	C	T	0.463	0.054	0.009	-0.017	0.017
**rs10745843**	G	A	0.389	-0.042	0.010	0.015	0.017
**rs10791849**	T	A	0.802	0.069	0.012	0.016	0.021
**rs10858823**	C	T	0.653	-0.048	0.010	-0.006	0.018
**rs10863102**	C	T	0.680	-0.042	0.010	0.024	0.018
**rs10866641**	T	C	0.566	0.063	0.009	0.014	0.017
**rs10869262**	G	A	0.373	-0.052	0.010	-0.005	0.017
**rs10873527**	T	C	0.650	0.043	0.010	0.013	0.019
**rs10903451**	A	G	0.636	0.045	0.010	-0.002	0.018
**rs10903559**	G	A	0.431	0.045	0.010	-0.018	0.017
**rs10917509**	T	C	0.356	0.053	0.010	-0.001	0.018
**rs10930634**	C	T	0.796	-0.055	0.012	0.021	0.021
**rs10932936**	C	G	0.532	-0.039	0.010	-0.027	0.017
**rs10937241**	A	G	0.175	-0.059	0.013	0.049	0.023
**rs10937550**	T	G	0.179	-0.052	0.013	0.010	0.022
**rs10967578**	C	T	0.901	-0.065	0.016	-0.039	0.028
**rs10973223**	T	C	0.839	-0.064	0.012	-0.029	0.023
**rs10994415**	T	C	0.922	-0.118	0.017	0.034	0.032
**rs11062170**	G	C	0.678	-0.078	0.010	-0.026	0.018
**rs11085744**	C	T	0.436	-0.040	0.010	-0.007	0.018
**rs111480622**	C	T	0.985	-0.173	0.037	-0.014	0.069
**rs111586616**	G	A	0.985	-0.159	0.038	-0.107	0.081
**rs111620325**	T	C	0.981	-0.180	0.042	-0.075	0.069
**rs111837408**	T	C	0.848	-0.066	0.014	-0.033	0.024
**rs11210099**	T	C	0.536	0.046	0.009	-0.001	0.017
**rs112219496**	G	A	0.840	-0.070	0.013	-0.026	0.023
**rs112481526**	A	G	0.728	-0.063	0.011	-0.092	0.019
**rs113779084**	G	A	0.710	-0.075	0.010	-0.049	0.019
**rs1145207**	A	G	0.948	-0.090	0.021	-0.029	0.039
**rs1156690**	T	A	0.663	-0.042	0.010	0.000	0.018

**rs115694474**	T	A	0.810	0.066	0.012	0.001	0.021
**rs11644048**	A	G	0.575	-0.049	0.010	0.000	0.019
**rs116660876**	T	A	0.950	-0.085	0.021	0.035	0.040
**rs11679617**	C	T	0.610	0.048	0.010	0.054	0.017
**rs116981909**	G	A	0.974	-0.163	0.036	-0.169	0.056
**rs117003004**	C	T	0.988	-0.191	0.042	-0.078	0.080
**rs11737121**	G	A	0.850	0.057	0.013	0.004	0.024
**rs117394301**	C	G	0.982	0.191	0.045	-0.002	0.068
**rs11764361**	A	G	0.669	0.061	0.010	-0.044	0.019
**rs11766995**	C	T	0.977	-0.102	0.024	-0.074	0.064
**rs11785781**	A	G	0.119	-0.064	0.014	-0.026	0.026
**rs11788723**	T	C	0.850	-0.061	0.013	0.005	0.024
**rs118134876**	C	T	0.950	0.088	0.021	-0.023	0.041
**rs11858458**	A	G	0.098	-0.082	0.016	-0.053	0.028
**rs11858715**	C	T	0.762	0.053	0.012	-0.024	0.022
**rs11876196**	A	G	0.950	0.104	0.024	-0.010	0.040
**rs11877693**	G	C	0.900	0.069	0.016	-0.044	0.028
**rs11950745**	C	G	0.687	-0.043	0.010	-0.003	0.019
**rs11958187**	G	A	0.371	-0.050	0.010	-0.015	0.018
**rs11963899**	A	G	0.853	-0.065	0.013	0.013	0.025
**rs1204834**	T	A	0.452	0.045	0.009	-0.019	0.017
**rs12050642**	A	G	0.741	-0.050	0.011	-0.026	0.019
**rs12055890**	C	A	0.833	-0.067	0.013	-0.036	0.023
**rs12065220**	C	T	0.635	-0.048	0.010	0.015	0.018
**rs12133979**	C	T	0.970	-0.118	0.028	-0.064	0.057
**rs12134005**	C	T	0.881	0.077	0.016	0.027	0.029
**rs12233703**	A	T	0.335	-0.048	0.010	-0.009	0.018
**rs12323512**	G	A	0.584	-0.040	0.010	0.013	0.017
**rs12333212**	T	C	0.809	0.056	0.012	0.051	0.022
**rs12407573**	G	C	0.874	0.067	0.014	0.014	0.026
**rs12411130**	C	A	0.647	0.050	0.010	0.007	0.018
**rs12464279**	T	G	0.724	0.049	0.011	-0.007	0.019
**rs12491058**	G	A	0.744	0.044	0.011	0.046	0.020
**rs12544947**	A	T	0.660	0.044	0.010	-0.012	0.018
**rs12575685**	G	A	0.694	-0.065	0.010	0.003	0.019
**rs12600720**	C	G	0.683	-0.044	0.011	-0.036	0.020
**rs12603807**	T	C	0.664	0.046	0.010	0.029	0.018
**rs12644938**	T	C	0.565	-0.042	0.010	-0.017	0.017
**rs12668848**	G	A	0.579	0.057	0.010	0.029	0.017
**rs12675289**	A	C	0.735	0.048	0.011	0.023	0.019
**rs12729719**	A	G	0.790	0.054	0.011	0.029	0.021
**rs12764732**	C	T	0.955	-0.106	0.022	0.004	0.041
**rs12783919**	G	A	0.656	0.047	0.010	0.011	0.018
**rs12865195**	A	G	0.570	0.039	0.010	0.008	0.017
**rs12892189**	C	A	0.676	-0.049	0.010	0.002	0.018

**rs12919664**	T	C	0.729	-0.053	0.011	-0.015	0.019
**rs12932628**	G	T	0.501	-0.057	0.010	0.000	0.019
**rs1298062**	A	G	0.332	-0.041	0.010	0.004	0.018
**rs1299858**	T	C	0.242	0.048	0.011	-0.005	0.020
**rs13044225**	A	G	0.542	-0.055	0.010	0.016	0.017
**rs13049106**	G	A	0.964	0.114	0.027	0.060	0.045
**rs13085687**	C	A	0.543	-0.039	0.010	-0.045	0.017
**rs13133001**	T	C	0.961	-0.103	0.024	0.068	0.051
**rs13195402**	G	T	0.936	0.136	0.018	0.055	0.039
**rs13333582**	T	C	0.953	-0.097	0.023	0.009	0.040
**rs13353610**	T	C	0.622	-0.042	0.010	-0.003	0.018
**rs13404366**	A	G	0.323	0.051	0.010	0.064	0.018
**rs13405781**	G	A	0.835	0.054	0.013	0.012	0.025
**rs13417268**	C	G	0.766	0.062	0.011	-0.026	0.020
**rs139005549**	C	T	0.974	-0.116	0.028	0.023	0.061
**rs139129362**	G	A	0.986	-0.276	0.057	-0.001	0.095
**rs1391439**	G	A	0.385	0.039	0.010	-0.036	0.017
**rs140565076**	G	A	0.976	0.168	0.032	0.067	0.058
**rs143278184**	A	C	0.986	-0.140	0.033	-0.086	0.086
**rs144225206**	G	A	0.923	-0.077	0.017	-0.053	0.031
**rs145279901**	G	A	0.974	-0.125	0.031	0.013	0.055
**rs145316860**	T	C	0.991	-0.164	0.039	0.053	0.117
**rs145767750**	T	C	0.970	0.127	0.028	0.094	0.055
**rs146655022**	C	T	0.992	-0.258	0.053	-0.305	0.109
**rs146950761**	C	G	0.841	-0.065	0.013	-0.017	0.023
**rs1470308**	A	G	0.646	-0.045	0.010	-0.004	0.018
**rs1477105**	A	G	0.663	0.042	0.010	0.000	0.018
**rs1487445**	C	T	0.527	-0.074	0.009	0.016	0.017
**rs149002246**	C	G	0.962	0.132	0.025	-0.002	0.045
**rs149174768**	A	G	0.987	-0.148	0.035	0.146	0.099
**rs149305303**	A	T	0.983	-0.166	0.038	-0.076	0.077
**rs1533309**	A	G	0.469	-0.040	0.010	0.005	0.018
**rs1646022**	C	G	0.546	0.046	0.009	0.002	0.017
**rs1689956**	T	G	0.481	0.039	0.010	-0.013	0.017
**rs1695769**	G	A	0.889	-0.064	0.015	-0.019	0.027
**rs1711506**	T	C	0.260	0.045	0.011	0.039	0.020
**rs17155863**	A	G	0.896	0.062	0.015	0.008	0.028
**rs17183814**	G	A	0.925	0.103	0.019	0.015	0.033
**rs1737333**	T	C	0.262	-0.044	0.011	0.014	0.019
**rs174592**	A	G	0.635	-0.072	0.010	-0.065	0.018
**rs174927**	C	T	0.290	-0.048	0.010	0.010	0.019
**rs17680262**	C	T	0.923	-0.091	0.018	-0.003	0.034
**rs17680579**	A	G	0.872	0.066	0.014	0.024	0.025
**rs17705372**	A	G	0.889	-0.066	0.015	-0.006	0.027
**rs1846162**	G	A	0.567	0.041	0.010	0.001	0.017

**rs1875665**	A	G	0.978	-0.164	0.035	-0.070	0.059
**rs1890787**	T	C	0.441	0.039	0.010	-0.015	0.017
**rs1895538**	T	C	0.480	0.039	0.009	-0.023	0.017
**rs191139555**	T	A	0.986	0.145	0.036	0.139	0.088
**rs1925122**	T	C	0.675	-0.043	0.010	-0.002	0.018
**rs1955454**	T	G	0.431	-0.042	0.010	-0.003	0.017
**rs1959440**	G	T	0.573	-0.048	0.010	-0.009	0.017
**rs1969208**	A	G	0.725	-0.046	0.011	-0.018	0.019
**rs197120**	G	C	0.151	0.070	0.013	-0.019	0.024
**rs1998820**	T	A	0.896	0.084	0.015	0.020	0.028
**rs2006760**	C	G	0.787	-0.052	0.012	0.008	0.021
**rs2009336**	C	T	0.546	-0.045	0.011	-0.029	0.023
**rs2011302**	T	A	0.632	-0.053	0.010	-0.007	0.017
**rs2038061**	A	G	0.434	-0.051	0.010	-0.037	0.017
**rs2126180**	G	A	0.544	-0.057	0.009	0.003	0.017
**rs2127288**	A	G	0.005	0.207	0.049	0.054	0.268
**rs217619**	A	G	0.288	0.044	0.010	-0.026	0.019
**rs2254900**	G	A	0.811	-0.051	0.012	-0.006	0.021
**rs2271047**	A	G	0.588	-0.044	0.010	0.005	0.018
**rs2273738**	C	T	0.849	-0.092	0.014	-0.011	0.024
**rs2282551**	T	C	0.670	-0.041	0.010	0.000	0.018
**rs228768**	G	T	0.314	0.064	0.010	-0.025	0.019
**rs2299098**	G	C	0.815	-0.060	0.012	0.005	0.022
**rs2305929**	A	G	0.804	-0.064	0.012	-0.037	0.021
**rs2336147**	T	C	0.493	0.068	0.009	0.032	0.017
**rs2339519**	G	A	0.554	-0.049	0.009	0.001	0.017
**rs237460**	C	T	0.559	-0.055	0.009	0.019	0.017
**rs2484034**	G	A	0.727	-0.050	0.011	0.028	0.019
**rs2496038**	T	G	0.255	0.050	0.011	0.002	0.019
**rs2496543**	C	T	0.607	0.041	0.010	0.028	0.017
**rs251381**	A	G	0.348	-0.049	0.010	0.003	0.018
**rs2551976**	T	C	0.533	-0.040	0.009	-0.011	0.018
**rs255373**	T	A	0.536	-0.050	0.009	-0.032	0.017
**rs2622540**	A	G	0.341	-0.041	0.010	-0.029	0.018
**rs2635209**	T	C	0.516	-0.051	0.009	-0.027	0.017
**rs2650087**	G	C	0.501	0.044	0.010	0.018	0.017
**rs2651566**	G	A	0.600	0.044	0.010	-0.015	0.017
**rs2693698**	A	G	0.450	-0.053	0.009	-0.005	0.017
**rs2697136**	G	T	0.652	-0.042	0.010	-0.009	0.019
**rs27957**	A	G	0.739	-0.046	0.011	-0.031	0.019
**rs2807742**	G	A	0.217	0.051	0.011	-0.004	0.021
**rs2809444**	G	C	0.543	0.039	0.009	0.000	0.017
**rs2814736**	T	C	0.397	0.039	0.010	0.011	0.017
**rs2817332**	T	A	0.144	0.067	0.015	0.017	0.026
**rs28360326**	A	T	0.967	-0.210	0.039	0.104	0.061

**rs28377676**	G	A	0.781	0.061	0.012	0.000	0.022
**rs28455634**	G	A	0.607	0.063	0.010	-0.026	0.018
**rs28565152**	G	A	0.757	-0.067	0.011	0.007	0.020
**rs28568531**	T	A	0.683	-0.044	0.010	0.048	0.018
**rs28865701**	A	G	0.637	-0.044	0.010	-0.006	0.018
**rs28890251**	T	C	0.646	0.046	0.010	-0.014	0.018
**rs2953928**	G	A	0.951	-0.116	0.020	-0.064	0.038
**rs2960323**	C	G	0.834	0.055	0.013	0.030	0.023
**rs2962370**	G	C	0.356	0.051	0.010	0.011	0.018
**rs308800**	T	C	0.800	0.056	0.012	0.018	0.021
**rs3145**	G	A	0.232	-0.053	0.011	0.035	0.020
**rs318517**	C	T	0.838	0.067	0.013	0.009	0.023
**rs3208937**	A	G	0.268	0.045	0.010	-0.006	0.019
**rs324017**	A	C	0.290	-0.053	0.011	0.030	0.019
**rs34044430**	T	C	0.693	0.046	0.010	0.022	0.021
**rs34223888**	C	T	0.968	-0.151	0.037	-0.108	0.056
**rs34324305**	T	C	0.822	0.056	0.012	0.037	0.022
**rs345257**	G	A	0.174	-0.059	0.012	0.018	0.022
**rs34550586**	A	G	0.530	0.045	0.009	0.013	0.017
**rs34568676**	G	A	0.763	-0.049	0.011	-0.018	0.020
**rs34626151**	A	G	0.814	-0.056	0.012	-0.002	0.023
**rs348798**	G	A	0.351	-0.050	0.010	-0.036	0.018
**rs35058554**	G	T	0.956	-0.089	0.020	-0.032	0.044
**rs35082563**	A	C	0.720	-0.048	0.010	-0.001	0.019
**rs35090414**	G	T	0.920	0.083	0.017	-0.032	0.031
**rs35306827**	G	A	0.771	0.066	0.011	-0.011	0.020
**rs35343411**	G	A	0.934	-0.089	0.018	0.053	0.034
**rs35512633**	C	T	0.886	0.072	0.015	0.028	0.026
**rs358699**	T	C	0.845	0.061	0.013	-0.005	0.024
**rs35944620**	A	G	0.910	-0.069	0.016	0.009	0.030
**rs35958438**	G	A	0.784	0.064	0.012	0.038	0.021
**rs35985675**	T	A	0.806	0.055	0.012	0.033	0.022
**rs3775065**	G	A	0.910	-0.078	0.017	-0.005	0.031
**rs3776433**	A	C	0.329	-0.044	0.011	-0.012	0.018
**rs3780001**	T	G	0.678	-0.043	0.010	-0.012	0.018
**rs3795443**	A	G	0.864	-0.061	0.013	-0.026	0.024
**rs384182**	A	G	0.430	0.041	0.009	-0.029	0.017
**rs40948**	G	A	0.658	0.045	0.010	0.030	0.018
**rs4239191**	G	A	0.442	0.041	0.009	-0.002	0.018
**rs4331993**	T	A	0.669	-0.055	0.010	-0.002	0.018
**rs4357172**	G	A	0.754	-0.048	0.011	-0.025	0.019
**rs4376446**	A	T	0.725	-0.056	0.010	-0.017	0.019
**rs4447398**	A	C	0.135	0.082	0.014	0.033	0.024
**rs4489684**	C	G	0.243	0.050	0.011	-0.044	0.020
**rs4619651**	G	A	0.690	0.066	0.010	0.001	0.018

**rs4666995**	A	G	0.240	0.057	0.011	0.000	0.020
**rs4676412**	G	A	0.232	-0.061	0.011	-0.057	0.021
**rs4684787**	C	T	0.676	-0.044	0.010	-0.021	0.019
**rs4738519**	T	C	0.862	-0.065	0.014	-0.044	0.025
**rs4783319**	G	A	0.728	-0.049	0.011	-0.005	0.019
**rs4786364**	C	G	0.588	0.043	0.010	0.024	0.018
**rs4788865**	G	T	0.703	0.055	0.011	-0.007	0.019
**rs4790841**	C	T	0.845	-0.073	0.013	-0.024	0.026
**rs4794563**	G	A	0.621	-0.047	0.010	-0.015	0.017
**rs4845399**	C	T	0.637	-0.055	0.010	0.038	0.018
**rs4884464**	T	C	0.373	0.049	0.010	0.003	0.017
**rs4899437**	C	G	0.586	-0.042	0.010	0.001	0.017
**rs4905906**	T	G	0.308	0.051	0.010	0.011	0.018
**rs4916266**	C	A	0.594	-0.043	0.010	-0.033	0.017
**rs4941932**	A	G	0.213	0.050	0.011	0.010	0.021
**rs4949980**	A	G	0.936	0.085	0.019	0.016	0.036
**rs4964660**	A	T	0.291	0.049	0.011	-0.015	0.018
**rs512285**	T	C	0.434	0.043	0.009	0.024	0.017
**rs535066**	G	T	0.418	-0.045	0.009	-0.009	0.017
**rs55647329**	C	T	0.865	0.059	0.014	0.010	0.025
**rs55661361**	G	A	0.665	0.041	0.010	-0.002	0.018
**rs56014219**	A	T	0.820	-0.064	0.014	-0.008	0.025
**rs56108920**	A	G	0.435	0.043	0.010	-0.001	0.017
**rs56331084**	C	A	0.815	-0.058	0.012	0.017	0.022
**rs56351137**	C	T	0.941	-0.092	0.020	-0.028	0.040
**rs57116584**	C	T	0.637	-0.047	0.010	-0.008	0.017
**rs5758064**	T	C	0.515	0.052	0.009	0.024	0.017
**rs577027**	C	G	0.739	-0.049	0.011	-0.024	0.020
**rs581614**	A	C	0.527	0.045	0.010	-0.012	0.017
**rs58627659**	C	T	0.920	0.075	0.017	0.018	0.031
**rs588882**	A	G	0.444	0.041	0.010	-0.016	0.017
**rs59212827**	G	A	0.459	0.050	0.010	0.031	0.017
**rs59234174**	C	T	0.834	-0.055	0.013	-0.027	0.023
**rs59881258**	G	A	0.438	-0.042	0.010	-0.022	0.017
**rs6007473**	C	T	0.515	0.041	0.010	-0.006	0.017
**rs6010045**	T	C	0.302	-0.047	0.010	0.009	0.019
**rs60271**	A	C	0.320	0.052	0.010	0.008	0.018
**rs6074741**	T	C	0.720	-0.052	0.010	0.030	0.019
**rs614230**	C	T	0.363	-0.040	0.010	0.036	0.018
**rs61554907**	G	T	0.898	-0.087	0.015	0.052	0.030
**rs61774748**	T	G	0.736	-0.052	0.011	-0.012	0.020
**rs617760**	A	G	0.309	0.046	0.010	-0.010	0.018
**rs61933110**	G	A	0.892	-0.063	0.015	-0.036	0.031
**rs62029325**	C	T	0.894	-0.070	0.016	-0.033	0.028
**rs62087023**	T	C	0.721	0.049	0.011	0.017	0.019

**rs62151026**	C	G	0.598	-0.043	0.010	0.000	0.017
**rs62218838**	A	C	0.986	-0.196	0.047	0.107	0.110
**rs62234939**	T	C	0.505	-0.046	0.009	-0.041	0.017
**rs62489493**	C	G	0.872	-0.090	0.014	-0.026	0.025
**rs62581014**	C	T	0.624	-0.065	0.012	0.023	0.022
**rs645565**	C	T	0.898	-0.085	0.016	0.011	0.030
**rs6502721**	C	T	0.343	0.044	0.010	-0.024	0.019
**rs6572737**	C	A	0.824	-0.057	0.012	-0.004	0.022
**rs6580698**	T	C	0.571	-0.046	0.010	-0.009	0.017
**rs66550110**	G	A	0.942	0.098	0.020	-0.082	0.037
**rs66704380**	T	G	0.828	0.056	0.013	0.015	0.026
**rs6715448**	T	C	0.298	0.047	0.010	-0.006	0.018
**rs6721570**	C	T	0.824	-0.050	0.012	-0.018	0.022
**rs6746786**	C	T	0.907	0.072	0.017	-0.014	0.030
**rs6747175**	T	C	0.311	0.045	0.011	-0.010	0.019
**rs6748160**	C	T	0.357	-0.041	0.010	-0.018	0.018
**rs67506493**	A	G	0.881	-0.065	0.015	0.029	0.029
**rs67712855**	T	G	0.686	0.068	0.010	-0.035	0.018
**rs6783438**	G	A	0.644	0.043	0.010	-0.010	0.019
**rs683808**	A	G	0.463	-0.039	0.010	0.002	0.017
**rs6838474**	C	T	0.040	-0.115	0.024	0.032	0.043
**rs6883429**	C	T	0.181	0.058	0.013	0.024	0.023
**rs6887473**	G	A	0.715	0.060	0.011	0.017	0.019
**rs6946056**	A	C	0.392	-0.053	0.010	0.005	0.017
**rs6954854**	G	A	0.420	0.058	0.009	0.003	0.017
**rs6960056**	G	A	0.556	-0.040	0.009	0.000	0.017
**rs696366**	C	A	0.548	0.052	0.009	-0.008	0.017
**rs6992333**	A	G	0.565	-0.060	0.010	-0.008	0.017
**rs7001397**	C	T	0.533	-0.044	0.010	-0.012	0.017
**rs7007192**	C	A	0.784	0.052	0.012	0.035	0.021
**rs7108878**	T	G	0.888	-0.081	0.015	0.044	0.027
**rs7131187**	A	C	0.946	-0.117	0.028	0.057	0.049
**rs7143692**	A	G	0.780	-0.051	0.011	-0.017	0.021
**rs7160151**	A	G	0.697	0.044	0.010	-0.028	0.019
**rs7201930**	T	C	0.682	-0.059	0.010	0.035	0.018
**rs7235698**	A	G	0.495	-0.039	0.009	-0.024	0.017
**rs72725665**	G	A	0.920	0.071	0.017	-0.006	0.032
**rs72745470**	C	G	0.631	-0.050	0.010	-0.023	0.018
**rs72800727**	T	A	0.953	-0.101	0.022	-0.048	0.045
**rs72815749**	A	G	0.961	0.127	0.025	0.029	0.044
**rs72827147**	T	G	0.942	0.101	0.024	-0.162	0.046
**rs72827820**	C	T	0.754	0.048	0.011	-0.026	0.021
**rs72920394**	G	A	0.910	-0.084	0.016	-0.052	0.031
**rs73103717**	G	A	0.990	-0.191	0.042	-0.260	0.113
**rs73136039**	C	T	0.875	0.059	0.014	0.046	0.026

**rs73155124**	C	G	0.986	-0.183	0.041	0.043	0.090
**rs73206066**	C	T	0.985	-0.184	0.042	-0.099	0.074
**rs733760**	G	T	0.560	0.051	0.010	-0.004	0.017
**rs735931**	A	G	0.493	0.050	0.010	-0.018	0.018
**rs73923135**	C	A	0.835	-0.063	0.013	-0.031	0.023
**rs74109996**	C	T	0.935	-0.080	0.019	-0.064	0.034
**rs74840318**	C	T	0.986	-0.197	0.043	0.038	0.077
**rs748455**	T	C	0.696	0.067	0.010	0.000	0.018
**rs7520007**	C	A	0.487	-0.039	0.009	0.003	0.017
**rs7534376**	A	G	0.970	-0.164	0.037	0.005	0.053
**rs75438122**	T	C	0.963	0.112	0.024	-0.035	0.052
**rs7544805**	C	T	0.665	-0.043	0.010	-0.014	0.018
**rs75623709**	T	G	0.917	-0.074	0.016	-0.057	0.030
**rs7573275**	G	A	0.716	-0.050	0.010	-0.014	0.019
**rs75888683**	G	T	0.959	-0.117	0.022	0.031	0.044
**rs759073**	T	C	0.568	-0.051	0.011	0.030	0.020
**rs76076778**	A	T	0.975	0.146	0.035	0.030	0.070
**rs7609336**	G	A	0.617	0.051	0.010	0.009	0.017
**rs76187039**	G	T	0.874	0.063	0.013	0.026	0.026
**rs764240**	G	A	0.604	0.043	0.010	0.031	0.017
**rs76769832**	C	T	0.923	-0.088	0.018	-0.025	0.033
**rs76795191**	A	G	0.352	0.044	0.010	-0.028	0.018
**rs7684**	T	G	0.592	-0.040	0.010	-0.033	0.018
**rs7707252**	A	G	0.736	-0.057	0.010	-0.002	0.019
**rs7709645**	G	C	0.516	-0.038	0.009	0.015	0.017
**rs77426572**	C	G	0.961	-0.140	0.028	-0.018	0.057
**rs77926607**	A	G	0.985	0.154	0.037	0.061	0.071
**rs78104110**	C	T	0.963	0.132	0.026	-0.020	0.051
**rs7842666**	G	A	0.488	-0.041	0.009	-0.013	0.017
**rs7870016**	T	A	0.528	0.039	0.009	0.034	0.017
**rs78885330**	T	A	0.936	0.103	0.024	0.031	0.044
**rs7895364**	G	C	0.593	-0.049	0.009	0.026	0.017
**rs789865**	A	C	0.551	0.041	0.009	0.014	0.017
**rs79269869**	G	A	0.992	-0.195	0.047	0.205	0.120
**rs7937640**	A	T	0.426	-0.048	0.010	-0.026	0.017
**rs7940866**	T	A	0.510	0.047	0.010	0.008	0.017
**rs79434268**	G	A	0.926	-0.076	0.017	-0.006	0.032
**rs7947951**	A	G	0.325	-0.047	0.010	0.004	0.018
**rs79576701**	T	C	0.916	-0.079	0.017	-0.057	0.031
**rs7969100**	C	T	0.728	-0.046	0.011	-0.015	0.020
**rs7982263**	T	C	0.415	-0.052	0.010	0.008	0.017
**rs79917448**	A	G	0.833	0.055	0.013	-0.022	0.023
**rs8040193**	T	C	0.965	-0.116	0.027	-0.113	0.053
**rs8043792**	T	C	0.502	-0.045	0.009	-0.010	0.017
**rs8090457**	A	G	0.502	-0.050	0.009	-0.034	0.017

**rs8131986**	A	C	0.507	-0.048	0.009	0.035	0.017
**rs817492**	T	C	0.121	-0.064	0.014	0.001	0.027
**rs823104**	T	C	0.126	-0.058	0.014	-0.029	0.026
**rs82390**	T	C	0.488	-0.042	0.009	-0.011	0.017
**rs880447**	G	C	0.592	-0.053	0.011	-0.010	0.019
**rs880983**	G	A	0.979	0.169	0.037	0.048	0.074
**rs909775**	G	T	0.682	-0.042	0.010	0.016	0.018
**rs9389556**	C	G	0.739	-0.044	0.011	-0.001	0.019
**rs9389625**	T	G	0.583	-0.040	0.010	0.001	0.017
**rs9395495**	G	A	0.396	-0.048	0.010	-0.015	0.017
**rs9397125**	T	C	0.703	0.045	0.010	0.004	0.018
**rs942636**	C	T	0.827	-0.055	0.012	-0.047	0.023
**rs9529103**	G	A	0.359	0.044	0.010	0.018	0.018
**rs9562480**	T	C	0.966	-0.124	0.028	-0.044	0.049
**rs9638618**	G	A	0.290	-0.046	0.010	-0.004	0.018
**rs9644288**	C	G	0.562	-0.041	0.009	0.020	0.017
**rs9671125**	T	G	0.373	0.053	0.010	0.021	0.017
**rs9812434**	C	T	0.541	-0.047	0.009	-0.014	0.017
**rs9834970**	T	C	0.509	-0.083	0.009	-0.014	0.017
**rs9883919**	T	A	0.552	0.046	0.010	-0.017	0.017
**rs994280**	A	G	0.662	-0.050	0.010	-0.033	0.018
**rs9944**	A	G	0.226	-0.056	0.011	-0.008	0.022

Table S43. Raw data for the MR analysis of the causal effect of BD on CD (P<5E-5)							
SNP	Effect allele	Other allele	Effect allele frequency	Beta.exposure	Se.exposure	Beta.outcome	Se.outcome
**rs10035722**	C	A	0.943	-0.099	0.023	-0.001	0.056
**rs10043984**	C	T	0.733	-0.059	0.011	0.015	0.026
**rs10131905**	C	A	0.593	0.048	0.010	0.020	0.024
**rs10237578**	G	C	0.233	-0.050	0.012	-0.012	0.028
**rs10255167**	G	A	0.231	-0.066	0.012	0.036	0.027
**rs1027453**	T	C	0.502	-0.042	0.010	-0.022	0.024
**rs1032292**	A	C	0.829	0.057	0.013	0.057	0.031
**rs1033291**	A	T	0.433	-0.040	0.009	-0.043	0.023
**rs10412427**	T	C	0.816	0.059	0.013	-0.002	0.031
**rs10455979**	C	G	0.524	-0.056	0.010	-0.018	0.023
**rs10505139**	A	G	0.833	-0.051	0.012	-0.006	0.030
**rs10520294**	C	T	0.845	0.056	0.013	0.010	0.032
**rs10737496**	C	T	0.467	0.054	0.009	-0.013	0.023
**rs10745843**	G	A	0.390	-0.042	0.010	-0.003	0.023
**rs10791849**	T	A	0.798	0.069	0.012	-0.005	0.028
**rs10858823**	C	T	0.655	-0.048	0.010	-0.009	0.024
**rs10863102**	C	T	0.676	-0.042	0.010	0.024	0.024
**rs10866641**	T	C	0.565	0.063	0.009	0.008	0.023
**rs10869262**	G	A	0.374	-0.052	0.010	0.011	0.024
**rs10873527**	T	C	0.652	0.043	0.010	0.023	0.026
**rs10903451**	A	G	0.641	0.045	0.010	-0.006	0.024
**rs10903559**	G	A	0.428	0.045	0.010	-0.043	0.023
**rs10917509**	T	C	0.359	0.053	0.010	0.018	0.024
**rs10930634**	C	T	0.797	-0.055	0.012	0.054	0.029
**rs10932936**	C	G	0.537	-0.039	0.010	-0.016	0.023
**rs10937241**	A	G	0.177	-0.059	0.013	0.026	0.031
**rs10937550**	T	G	0.183	-0.052	0.013	-0.009	0.030
**rs10967578**	C	T	0.898	-0.065	0.016	-0.014	0.038
**rs10973223**	T	C	0.839	-0.064	0.012	-0.067	0.031
**rs10994415**	T	C	0.923	-0.118	0.017	0.014	0.043
**rs11062170**	G	C	0.682	-0.078	0.010	-0.020	0.024
**rs11085744**	C	T	0.438	-0.040	0.010	-0.001	0.024
**rs111480622**	C	T	0.985	-0.173	0.037	0.069	0.096
**rs111586616**	G	A	0.985	-0.159	0.038	-0.096	0.115
**rs111620325**	T	C	0.980	-0.180	0.042	-0.090	0.091
**rs111837408**	T	C	0.848	-0.066	0.014	-0.052	0.034
**rs11210099**	T	C	0.536	0.046	0.009	0.022	0.023
**rs112219496**	G	A	0.837	-0.070	0.013	-0.031	0.031
**rs112481526**	A	G	0.733	-0.063	0.011	-0.090	0.026
**rs113779084**	G	A	0.716	-0.075	0.010	-0.048	0.026
**rs1145207**	A	G	0.948	-0.090	0.021	0.075	0.054
**rs1156690**	T	A	0.662	-0.042	0.010	0.007	0.024

**rs115694474**	T	A	0.813	0.066	0.012	-0.009	0.029
**rs11644048**	A	G	0.561	-0.049	0.010	-0.002	0.025
**rs116660876**	T	A	0.951	-0.085	0.021	-0.020	0.055
**rs11679617**	C	T	0.606	0.048	0.010	0.013	0.024
**rs116981909**	G	A	0.973	-0.163	0.036	-0.162	0.075
**rs117003004**	C	T	0.988	-0.191	0.042	-0.180	0.109
**rs11737121**	G	A	0.851	0.057	0.013	0.003	0.032
**rs117394301**	C	G	0.981	0.191	0.045	0.085	0.092
**rs11764361**	A	G	0.669	0.061	0.010	-0.025	0.026
**rs11766995**	C	T	0.978	-0.102	0.024	-0.135	0.089
**rs11785781**	A	G	0.117	-0.064	0.014	-0.007	0.036
**rs11788723**	T	C	0.849	-0.061	0.013	0.016	0.032
**rs118134876**	C	T	0.948	0.088	0.021	-0.062	0.053
**rs11858458**	A	G	0.097	-0.082	0.016	-0.052	0.039
**rs11858715**	C	T	0.767	0.053	0.012	0.001	0.031
**rs11876196**	A	G	0.951	0.104	0.024	0.048	0.054
**rs11877693**	G	C	0.900	0.069	0.016	-0.035	0.038
**rs11950745**	C	G	0.686	-0.043	0.010	-0.024	0.025
**rs11958187**	G	A	0.367	-0.050	0.010	-0.022	0.025
**rs11963899**	A	G	0.854	-0.065	0.013	-0.002	0.034
**rs1204834**	T	A	0.448	0.045	0.009	-0.007	0.023
**rs12050642**	A	G	0.742	-0.050	0.011	-0.055	0.026
**rs12055890**	C	A	0.837	-0.067	0.013	-0.011	0.032
**rs12065220**	C	T	0.633	-0.048	0.010	0.002	0.025
**rs12133979**	C	T	0.970	-0.118	0.028	-0.030	0.077
**rs12134005**	C	T	0.880	0.077	0.016	0.093	0.040
**rs12233703**	A	T	0.334	-0.048	0.010	-0.012	0.024
**rs12323512**	G	A	0.583	-0.040	0.010	-0.002	0.023
**rs12333212**	T	C	0.810	0.056	0.012	0.070	0.031
**rs12407573**	G	C	0.874	0.067	0.014	-0.031	0.035
**rs12411130**	C	A	0.655	0.050	0.010	0.028	0.025
**rs12464279**	T	G	0.722	0.049	0.011	-0.023	0.026
**rs12491058**	G	A	0.740	0.044	0.011	0.059	0.028
**rs12544947**	A	T	0.663	0.044	0.010	-0.036	0.024
**rs12575685**	G	A	0.699	-0.065	0.010	0.020	0.026
**rs12600720**	C	G	0.680	-0.044	0.011	-0.057	0.027
**rs12603807**	T	C	0.664	0.046	0.010	0.048	0.024
**rs12644938**	T	C	0.566	-0.042	0.010	-0.017	0.023
**rs12668848**	G	A	0.577	0.057	0.010	0.054	0.023
**rs12675289**	A	C	0.737	0.048	0.011	0.023	0.026
**rs12729719**	A	G	0.790	0.054	0.011	-0.013	0.028
**rs12764732**	C	T	0.955	-0.106	0.022	-0.010	0.055
**rs12783919**	G	A	0.656	0.047	0.010	-0.005	0.024
**rs12865195**	A	G	0.568	0.039	0.010	0.027	0.023
**rs12892189**	C	A	0.680	-0.049	0.010	-0.012	0.025

**rs12919664**	T	C	0.731	-0.053	0.011	0.014	0.026
**rs12932628**	G	T	0.497	-0.057	0.010	-0.017	0.026
**rs1298062**	A	G	0.332	-0.041	0.010	0.016	0.024
**rs1299858**	T	C	0.240	0.048	0.011	-0.044	0.028
**rs13044225**	A	G	0.546	-0.055	0.010	0.013	0.023
**rs13049106**	G	A	0.963	0.114	0.027	-0.007	0.060
**rs13085687**	C	A	0.543	-0.039	0.010	-0.004	0.024
**rs13133001**	T	C	0.960	-0.103	0.024	0.006	0.069
**rs13195402**	G	T	0.942	0.136	0.018	0.114	0.057
**rs13333582**	T	C	0.952	-0.097	0.023	0.023	0.054
**rs13353610**	T	C	0.622	-0.042	0.010	-0.019	0.025
**rs13404366**	A	G	0.319	0.051	0.010	0.082	0.025
**rs13405781**	G	A	0.832	0.054	0.013	0.037	0.034
**rs13417268**	C	G	0.768	0.062	0.011	-0.020	0.027
**rs139005549**	C	T	0.974	-0.116	0.028	-0.005	0.084
**rs139129362**	G	A	0.986	-0.276	0.057	0.082	0.126
**rs1391439**	G	A	0.390	0.039	0.010	-0.007	0.023
**rs140565076**	G	A	0.975	0.168	0.032	-0.025	0.078
**rs143278184**	A	C	0.987	-0.140	0.033	-0.136	0.124
**rs144225206**	G	A	0.925	-0.077	0.017	-0.026	0.044
**rs145279901**	G	A	0.973	-0.125	0.031	0.007	0.075
**rs145316860**	T	C	0.991	-0.164	0.039	-0.091	0.145
**rs145767750**	T	C	0.971	0.127	0.028	0.104	0.076
**rs146655022**	C	T	0.992	-0.258	0.053	-0.242	0.140
**rs146950761**	C	G	0.841	-0.065	0.013	-0.007	0.032
**rs1470308**	A	G	0.648	-0.045	0.010	0.011	0.024
**rs1477105**	A	G	0.664	0.042	0.010	0.019	0.024
**rs1487445**	C	T	0.525	-0.074	0.009	0.003	0.023
**rs149002246**	C	G	0.961	0.132	0.025	-0.016	0.061
**rs149174768**	A	G	0.988	-0.148	0.035	0.072	0.141
**rs149305303**	A	T	0.982	-0.166	0.038	-0.116	0.102
**rs1533309**	A	G	0.453	-0.040	0.010	0.001	0.026
**rs1646022**	C	G	0.551	0.046	0.009	0.034	0.023
**rs1689956**	T	G	0.479	0.039	0.010	-0.009	0.024
**rs1695769**	G	A	0.886	-0.064	0.015	-0.001	0.037
**rs1711506**	T	C	0.257	0.045	0.011	0.051	0.028
**rs17155863**	A	G	0.897	0.062	0.015	0.003	0.038
**rs17183814**	G	A	0.926	0.103	0.019	0.031	0.045
**rs1737333**	T	C	0.262	-0.044	0.011	0.005	0.026
**rs174592**	A	G	0.635	-0.072	0.010	-0.117	0.024
**rs174927**	C	T	0.289	-0.048	0.010	0.022	0.025
**rs17680262**	C	T	0.924	-0.091	0.018	0.002	0.046
**rs17680579**	A	G	0.869	0.066	0.014	0.068	0.034
**rs17705372**	A	G	0.891	-0.066	0.015	-0.033	0.037
**rs1846162**	G	A	0.564	0.041	0.010	0.029	0.023

**rs1875665**	A	G	0.978	-0.164	0.035	-0.188	0.077
**rs1890787**	T	C	0.444	0.039	0.010	-0.015	0.023
**rs1895538**	T	C	0.479	0.039	0.009	-0.023	0.023
**rs191139555**	T	A	0.987	0.145	0.036	0.097	0.127
**rs1925122**	T	C	0.670	-0.043	0.010	-0.020	0.024
**rs1955454**	T	G	0.431	-0.042	0.010	-0.022	0.023
**rs1959440**	G	T	0.577	-0.048	0.010	-0.013	0.023
**rs1969208**	A	G	0.726	-0.046	0.011	-0.020	0.026
**rs197120**	G	C	0.150	0.070	0.013	-0.063	0.034
**rs1998820**	T	A	0.896	0.084	0.015	0.003	0.038
**rs2006760**	C	G	0.785	-0.052	0.012	0.038	0.030
**rs2009336**	C	T	0.542	-0.045	0.011	0.014	0.032
**rs2011302**	T	A	0.631	-0.053	0.010	-0.021	0.024
**rs2038061**	A	G	0.431	-0.051	0.010	-0.035	0.023
**rs2126180**	G	A	0.545	-0.057	0.009	0.015	0.023
**rs2127288**	A	G	0.005	0.207	0.049	0.225	0.254
**rs217619**	A	G	0.294	0.044	0.010	-0.022	0.025
**rs2254900**	G	A	0.806	-0.051	0.012	-0.006	0.029
**rs2271047**	A	G	0.584	-0.044	0.010	0.007	0.024
**rs2273738**	C	T	0.846	-0.092	0.014	0.012	0.032
**rs2282551**	T	C	0.670	-0.041	0.010	-0.011	0.024
**rs228768**	G	T	0.318	0.064	0.010	-0.035	0.025
**rs2299098**	G	C	0.817	-0.060	0.012	0.024	0.030
**rs2305929**	A	G	0.799	-0.064	0.012	0.003	0.029
**rs2336147**	T	C	0.494	0.068	0.009	0.011	0.023
**rs2339519**	G	A	0.557	-0.049	0.009	0.013	0.023
**rs237460**	C	T	0.556	-0.055	0.009	0.056	0.023
**rs2484034**	G	A	0.725	-0.050	0.011	0.045	0.026
**rs2496038**	T	G	0.252	0.050	0.011	-0.023	0.027
**rs2496543**	C	T	0.604	0.041	0.010	0.029	0.023
**rs251381**	A	G	0.350	-0.049	0.010	0.007	0.024
**rs2551976**	T	C	0.544	-0.040	0.009	0.004	0.024
**rs255373**	T	A	0.534	-0.050	0.009	-0.038	0.023
**rs2622540**	A	G	0.337	-0.041	0.010	-0.057	0.024
**rs2635209**	T	C	0.515	-0.051	0.009	-0.023	0.023
**rs2650087**	G	C	0.502	0.044	0.010	0.014	0.023
**rs2651566**	G	A	0.599	0.044	0.010	-0.034	0.023
**rs2693698**	A	G	0.452	-0.053	0.009	-0.012	0.023
**rs2697136**	G	T	0.648	-0.042	0.010	-0.036	0.026
**rs27957**	A	G	0.740	-0.046	0.011	-0.036	0.026
**rs2807742**	G	A	0.217	0.051	0.011	0.008	0.028
**rs2809444**	G	C	0.543	0.039	0.009	-0.013	0.023
**rs2814736**	T	C	0.398	0.039	0.010	-0.004	0.024
**rs2817332**	T	A	0.142	0.067	0.015	0.007	0.035
**rs28360326**	A	T	0.967	-0.210	0.039	0.114	0.086

**rs28377676**	G	A	0.777	0.061	0.012	-0.021	0.030
**rs28455634**	G	A	0.609	0.063	0.010	-0.047	0.024
**rs28565152**	G	A	0.757	-0.067	0.011	0.015	0.028
**rs28568531**	T	A	0.681	-0.044	0.010	-0.034	0.024
**rs28865701**	A	G	0.641	-0.044	0.010	0.023	0.024
**rs28890251**	T	C	0.655	0.046	0.010	-0.025	0.025
**rs2953928**	G	A	0.953	-0.116	0.020	-0.071	0.054
**rs2960323**	C	G	0.831	0.055	0.013	-0.003	0.031
**rs2962370**	G	C	0.362	0.051	0.010	-0.001	0.025
**rs308800**	T	C	0.797	0.056	0.012	0.017	0.029
**rs3145**	G	A	0.228	-0.053	0.011	0.018	0.027
**rs318517**	C	T	0.837	0.067	0.013	-0.009	0.031
**rs3208937**	A	G	0.264	0.045	0.010	-0.008	0.026
**rs324017**	A	C	0.289	-0.053	0.011	0.091	0.026
**rs34044430**	T	C	0.691	0.046	0.010	0.020	0.027
**rs34223888**	C	T	0.969	-0.151	0.037	-0.197	0.077
**rs34324305**	T	C	0.824	0.056	0.012	0.028	0.030
**rs345257**	G	A	0.171	-0.059	0.012	0.037	0.030
**rs34550586**	A	G	0.522	0.045	0.009	0.008	0.023
**rs34568676**	G	A	0.764	-0.049	0.011	-0.002	0.028
**rs34626151**	A	G	0.814	-0.056	0.012	0.014	0.032
**rs348798**	G	A	0.353	-0.050	0.010	-0.047	0.024
**rs35058554**	G	T	0.957	-0.089	0.020	-0.061	0.060
**rs35082563**	A	C	0.721	-0.048	0.010	-0.003	0.025
**rs35090414**	G	T	0.921	0.083	0.017	-0.006	0.043
**rs35306827**	G	A	0.773	0.066	0.011	-0.020	0.027
**rs35343411**	G	A	0.935	-0.089	0.018	0.041	0.048
**rs35512633**	C	T	0.884	0.072	0.015	0.031	0.036
**rs358699**	T	C	0.849	0.061	0.013	0.012	0.033
**rs35944620**	A	G	0.910	-0.069	0.016	0.006	0.040
**rs35958438**	G	A	0.787	0.064	0.012	0.042	0.029
**rs35985675**	T	A	0.803	0.055	0.012	0.034	0.030
**rs3775065**	G	A	0.909	-0.078	0.017	-0.029	0.042
**rs3776433**	A	C	0.324	-0.044	0.011	-0.025	0.024
**rs3780001**	T	G	0.683	-0.043	0.010	0.006	0.024
**rs3795443**	A	G	0.867	-0.061	0.013	-0.034	0.033
**rs384182**	A	G	0.433	0.041	0.009	-0.010	0.024
**rs40948**	G	A	0.659	0.045	0.010	0.034	0.024
**rs4239191**	G	A	0.444	0.041	0.009	-0.040	0.024
**rs4331993**	T	A	0.668	-0.055	0.010	-0.019	0.024
**rs4357172**	G	A	0.756	-0.048	0.011	-0.033	0.027
**rs4376446**	A	T	0.730	-0.056	0.010	-0.019	0.026
**rs4447398**	A	C	0.136	0.082	0.014	0.033	0.033
**rs4489684**	C	G	0.235	0.050	0.011	-0.033	0.027
**rs4619651**	G	A	0.697	0.066	0.010	0.023	0.025

**rs4666995**	A	G	0.240	0.057	0.011	0.026	0.027
**rs4676412**	G	A	0.235	-0.061	0.011	-0.039	0.028
**rs4684787**	C	T	0.677	-0.044	0.010	-0.017	0.025
**rs4738519**	T	C	0.861	-0.065	0.014	-0.037	0.034
**rs4783319**	G	A	0.731	-0.049	0.011	0.008	0.026
**rs4786364**	C	G	0.590	0.043	0.010	-0.016	0.024
**rs4788865**	G	T	0.698	0.055	0.011	-0.001	0.026
**rs4790841**	C	T	0.848	-0.073	0.013	-0.019	0.035
**rs4794563**	G	A	0.622	-0.047	0.010	-0.026	0.023
**rs4845399**	C	T	0.635	-0.055	0.010	0.047	0.024
**rs4884464**	T	C	0.373	0.049	0.010	0.010	0.023
**rs4899437**	C	G	0.582	-0.042	0.010	0.023	0.024
**rs4905906**	T	G	0.307	0.051	0.010	0.019	0.025
**rs4916266**	C	A	0.595	-0.043	0.010	-0.041	0.023
**rs4941932**	A	G	0.210	0.050	0.011	0.053	0.028
**rs4949980**	A	G	0.937	0.085	0.019	0.065	0.051
**rs4964660**	A	T	0.291	0.049	0.011	-0.010	0.025
**rs512285**	T	C	0.432	0.043	0.009	0.034	0.023
**rs535066**	G	T	0.416	-0.045	0.009	0.007	0.023
**rs55647329**	C	T	0.865	0.059	0.014	0.057	0.034
**rs55661361**	G	A	0.664	0.041	0.010	0.011	0.025
**rs56014219**	A	T	0.822	-0.064	0.014	0.032	0.034
**rs56108920**	A	G	0.433	0.043	0.010	0.023	0.024
**rs56331084**	C	A	0.820	-0.058	0.012	0.085	0.030
**rs56351137**	C	T	0.939	-0.092	0.020	-0.113	0.053
**rs57116584**	C	T	0.637	-0.047	0.010	-0.025	0.024
**rs5758064**	T	C	0.516	0.052	0.009	0.045	0.023
**rs577027**	C	G	0.740	-0.049	0.011	-0.013	0.027
**rs581614**	A	C	0.530	0.045	0.010	-0.002	0.023
**rs58627659**	C	T	0.919	0.075	0.017	0.001	0.042
**rs588882**	A	G	0.445	0.041	0.010	0.002	0.023
**rs59212827**	G	A	0.454	0.050	0.010	0.013	0.023
**rs59234174**	C	T	0.834	-0.055	0.013	-0.037	0.031
**rs59881258**	G	A	0.437	-0.042	0.010	-0.033	0.024
**rs6007473**	C	T	0.510	0.041	0.010	0.008	0.024
**rs6010045**	T	C	0.302	-0.047	0.010	0.000	0.026
**rs60271**	A	C	0.317	0.052	0.010	-0.025	0.025
**rs6074741**	T	C	0.720	-0.052	0.010	0.075	0.026
**rs614230**	C	T	0.359	-0.040	0.010	0.021	0.024
**rs61554907**	G	T	0.896	-0.087	0.015	0.041	0.040
**rs61774748**	T	G	0.732	-0.052	0.011	-0.024	0.027
**rs617760**	A	G	0.308	0.046	0.010	0.019	0.025
**rs61933110**	G	A	0.893	-0.063	0.015	-0.066	0.041
**rs62029325**	C	T	0.893	-0.070	0.016	-0.065	0.037
**rs62087023**	T	C	0.723	0.049	0.011	0.018	0.026

**rs62151026**	C	G	0.600	-0.043	0.010	-0.001	0.024
**rs62218838**	A	C	0.987	-0.196	0.047	0.262	0.164
**rs62234939**	T	C	0.502	-0.046	0.009	-0.049	0.023
**rs62489493**	C	G	0.872	-0.090	0.014	-0.088	0.034
**rs62581014**	C	T	0.625	-0.065	0.012	0.058	0.032
**rs645565**	C	T	0.895	-0.085	0.016	-0.026	0.040
**rs6502721**	C	T	0.341	0.044	0.010	-0.055	0.025
**rs6572737**	C	A	0.826	-0.057	0.012	-0.029	0.030
**rs6580698**	T	C	0.575	-0.046	0.010	-0.004	0.023
**rs66550110**	G	A	0.945	0.098	0.020	-0.064	0.051
**rs66704380**	T	G	0.826	0.056	0.013	0.042	0.036
**rs6715448**	T	C	0.299	0.047	0.010	-0.027	0.025
**rs6721570**	C	T	0.827	-0.050	0.012	0.004	0.030
**rs6746786**	C	T	0.904	0.072	0.017	-0.015	0.040
**rs6747175**	T	C	0.308	0.045	0.011	-0.061	0.027
**rs6748160**	C	T	0.363	-0.041	0.010	-0.020	0.024
**rs67506493**	A	G	0.880	-0.065	0.015	0.037	0.041
**rs67712855**	T	G	0.681	0.068	0.010	-0.007	0.025
**rs6783438**	G	A	0.651	0.043	0.010	-0.008	0.025
**rs683808**	A	G	0.466	-0.039	0.010	-0.011	0.023
**rs6838474**	C	T	0.040	-0.115	0.024	0.033	0.058
**rs6883429**	C	T	0.182	0.058	0.013	0.007	0.031
**rs6887473**	G	A	0.715	0.060	0.011	0.027	0.025
**rs6946056**	A	C	0.387	-0.053	0.010	0.007	0.024
**rs6954854**	G	A	0.420	0.058	0.009	-0.004	0.023
**rs6960056**	G	A	0.558	-0.040	0.009	-0.001	0.023
**rs696366**	C	A	0.551	0.052	0.009	-0.013	0.023
**rs6992333**	A	G	0.562	-0.060	0.010	0.003	0.023
**rs7001397**	C	T	0.532	-0.044	0.010	-0.022	0.023
**rs7007192**	C	A	0.782	0.052	0.012	-0.001	0.028
**rs7108878**	T	G	0.888	-0.081	0.015	0.041	0.036
**rs7131187**	A	C	0.945	-0.117	0.028	0.069	0.065
**rs7143692**	A	G	0.780	-0.051	0.011	0.010	0.029
**rs7160151**	A	G	0.694	0.044	0.010	-0.050	0.025
**rs7201930**	T	C	0.677	-0.059	0.010	0.038	0.025
**rs7235698**	A	G	0.493	-0.039	0.009	-0.042	0.023
**rs72725665**	G	A	0.921	0.071	0.017	-0.028	0.043
**rs72745470**	C	G	0.630	-0.050	0.010	-0.042	0.025
**rs72800727**	T	A	0.954	-0.101	0.022	-0.037	0.061
**rs72815749**	A	G	0.962	0.127	0.025	0.004	0.060
**rs72827147**	T	G	0.945	0.101	0.024	-0.138	0.065
**rs72827820**	C	T	0.756	0.048	0.011	-0.015	0.029
**rs72920394**	G	A	0.913	-0.084	0.016	-0.082	0.043
**rs73103717**	G	A	0.990	-0.191	0.042	-0.214	0.153
**rs73136039**	C	T	0.876	0.059	0.014	0.036	0.036

**rs73155124**	C	G	0.984	-0.183	0.041	-0.028	0.115
**rs73206066**	C	T	0.984	-0.184	0.042	-0.034	0.100
**rs733760**	G	T	0.559	0.051	0.010	0.013	0.023
**rs735931**	A	G	0.488	0.050	0.010	-0.003	0.025
**rs73923135**	C	A	0.835	-0.063	0.013	-0.046	0.031
**rs74109996**	C	T	0.936	-0.080	0.019	-0.058	0.047
**rs74840318**	C	T	0.985	-0.197	0.043	-0.034	0.100
**rs748455**	T	C	0.694	0.067	0.010	0.009	0.025
**rs7520007**	C	A	0.480	-0.039	0.009	0.015	0.023
**rs7534376**	A	G	0.967	-0.164	0.037	-0.045	0.068
**rs75438122**	T	C	0.964	0.112	0.024	0.013	0.074
**rs7544805**	C	T	0.665	-0.043	0.010	-0.037	0.024
**rs75623709**	T	G	0.917	-0.074	0.016	-0.069	0.041
**rs7573275**	G	A	0.716	-0.050	0.010	-0.029	0.025
**rs75888683**	G	T	0.960	-0.117	0.022	-0.025	0.060
**rs759073**	T	C	0.573	-0.051	0.011	0.013	0.027
**rs76076778**	A	T	0.974	0.146	0.035	0.101	0.093
**rs7609336**	G	A	0.618	0.051	0.010	0.024	0.024
**rs76187039**	G	T	0.878	0.063	0.013	0.059	0.036
**rs764240**	G	A	0.602	0.043	0.010	0.020	0.023
**rs76769832**	C	T	0.924	-0.088	0.018	-0.040	0.045
**rs76795191**	A	G	0.351	0.044	0.010	-0.054	0.024
**rs7684**	T	G	0.587	-0.040	0.010	-0.030	0.024
**rs7707252**	A	G	0.739	-0.057	0.010	0.026	0.027
**rs7709645**	G	C	0.515	-0.038	0.009	0.002	0.023
**rs77426572**	C	G	0.961	-0.140	0.028	-0.096	0.077
**rs77926607**	A	G	0.987	0.154	0.037	0.090	0.103
**rs78104110**	C	T	0.961	0.132	0.026	-0.014	0.068
**rs7842666**	G	A	0.491	-0.041	0.009	-0.031	0.023
**rs7870016**	T	A	0.528	0.039	0.009	0.052	0.023
**rs78885330**	T	A	0.933	0.103	0.024	-0.020	0.058
**rs7895364**	G	C	0.600	-0.049	0.009	0.054	0.023
**rs789865**	A	C	0.552	0.041	0.009	0.022	0.023
**rs79269869**	G	A	0.992	-0.195	0.047	0.013	0.151
**rs7937640**	A	T	0.429	-0.048	0.010	-0.022	0.023
**rs7940866**	T	A	0.517	0.047	0.010	-0.023	0.023
**rs79434268**	G	A	0.927	-0.076	0.017	0.003	0.044
**rs7947951**	A	G	0.324	-0.047	0.010	0.032	0.024
**rs79576701**	T	C	0.918	-0.079	0.017	-0.031	0.043
**rs7969100**	C	T	0.727	-0.046	0.011	-0.017	0.027
**rs7982263**	T	C	0.419	-0.052	0.010	0.034	0.023
**rs79917448**	A	G	0.834	0.055	0.013	-0.014	0.031
**rs8040193**	T	C	0.964	-0.116	0.027	-0.138	0.070
**rs8043792**	T	C	0.501	-0.045	0.009	0.011	0.023
**rs8090457**	A	G	0.500	-0.050	0.009	-0.040	0.023

**rs8131986**	A	C	0.506	-0.048	0.009	0.073	0.023
**rs817492**	T	C	0.118	-0.064	0.014	-0.009	0.037
**rs823104**	T	C	0.127	-0.058	0.014	-0.012	0.035
**rs82390**	T	C	0.488	-0.042	0.009	-0.015	0.023
**rs880447**	G	C	0.588	-0.053	0.011	-0.045	0.027
**rs880983**	G	A	0.978	0.169	0.037	0.057	0.103
**rs909775**	G	T	0.674	-0.042	0.010	-0.005	0.025
**rs9389556**	C	G	0.735	-0.044	0.011	-0.003	0.026
**rs9389625**	T	G	0.580	-0.040	0.010	-0.001	0.023
**rs9395495**	G	A	0.401	-0.048	0.010	-0.021	0.023
**rs9397125**	T	C	0.703	0.045	0.010	0.019	0.025
**rs942636**	C	T	0.832	-0.055	0.012	-0.016	0.031
**rs9529103**	G	A	0.361	0.044	0.010	0.014	0.025
**rs9562480**	T	C	0.966	-0.124	0.028	0.046	0.071
**rs9638618**	G	A	0.286	-0.046	0.010	-0.019	0.025
**rs9644288**	C	G	0.565	-0.041	0.009	0.008	0.023
**rs9671125**	T	G	0.377	0.053	0.010	0.020	0.023
**rs9812434**	C	T	0.541	-0.047	0.009	-0.021	0.023
**rs9834970**	T	C	0.509	-0.083	0.009	-0.008	0.023
**rs9883919**	T	A	0.556	0.046	0.010	0.000	0.023
**rs994280**	A	G	0.659	-0.050	0.010	-0.065	0.024
**rs9944**	A	G	0.226	-0.056	0.011	-0.040	0.030

Table S44. Raw data for the MR analysis of the causal effect of BD on UC (P<5E-5)							
SNP	Effect allele	Other allele	Effect allele frequency	Beta.exposure	Se.exposure	Beta.outcome	Se.outcome
**rs10035722**	C	A	0.944	-0.099	0.023	-0.025	0.042
**rs10043984**	C	T	0.734	-0.059	0.011	-0.020	0.019
**rs10131905**	C	A	0.597	0.048	0.010	0.007	0.017
**rs10237578**	G	C	0.231	-0.050	0.012	-0.012	0.021
**rs10255167**	G	A	0.229	-0.066	0.012	0.013	0.020
**rs1027453**	T	C	0.498	-0.042	0.010	-0.030	0.017
**rs1032292**	A	C	0.829	0.057	0.013	0.054	0.023
**rs1033291**	A	T	0.436	-0.040	0.009	-0.019	0.017
**rs10412427**	T	C	0.814	0.059	0.013	-0.012	0.023
**rs10455979**	C	G	0.523	-0.056	0.010	-0.021	0.017
**rs10505139**	A	G	0.832	-0.051	0.012	-0.015	0.022
**rs10520294**	C	T	0.845	0.056	0.013	-0.009	0.023
**rs10737496**	C	T	0.463	0.054	0.009	-0.017	0.017
**rs10745843**	G	A	0.389	-0.042	0.010	0.015	0.017
**rs10791849**	T	A	0.802	0.069	0.012	0.016	0.021
**rs10858823**	C	T	0.653	-0.048	0.010	-0.006	0.018
**rs10863102**	C	T	0.680	-0.042	0.010	0.024	0.018
**rs10866641**	T	C	0.566	0.063	0.009	0.014	0.017
**rs10869262**	G	A	0.373	-0.052	0.010	-0.005	0.017
**rs10873527**	T	C	0.650	0.043	0.010	0.013	0.019
**rs10903451**	A	G	0.636	0.045	0.010	-0.002	0.018
**rs10903559**	G	A	0.431	0.045	0.010	-0.018	0.017
**rs10917509**	T	C	0.356	0.053	0.010	-0.001	0.018
**rs10930634**	C	T	0.796	-0.055	0.012	0.021	0.021
**rs10932936**	C	G	0.532	-0.039	0.010	-0.027	0.017
**rs10937241**	A	G	0.175	-0.059	0.013	0.049	0.023
**rs10937550**	T	G	0.179	-0.052	0.013	0.010	0.022
**rs10967578**	C	T	0.901	-0.065	0.016	-0.039	0.028
**rs10973223**	T	C	0.839	-0.064	0.012	-0.029	0.023
**rs10994415**	T	C	0.922	-0.118	0.017	0.034	0.032
**rs11062170**	G	C	0.678	-0.078	0.010	-0.026	0.018
**rs11085744**	C	T	0.436	-0.040	0.010	-0.007	0.018
**rs111480622**	C	T	0.985	-0.173	0.037	-0.014	0.069
**rs111586616**	G	A	0.985	-0.159	0.038	-0.107	0.081
**rs111620325**	T	C	0.981	-0.180	0.042	-0.075	0.069
**rs111837408**	T	C	0.848	-0.066	0.014	-0.033	0.024
**rs11210099**	T	C	0.536	0.046	0.009	-0.001	0.017
**rs112219496**	G	A	0.840	-0.070	0.013	-0.026	0.023
**rs112481526**	A	G	0.728	-0.063	0.011	-0.092	0.019
**rs113779084**	G	A	0.710	-0.075	0.010	-0.049	0.019
**rs1145207**	A	G	0.948	-0.090	0.021	-0.029	0.039
**rs1156690**	T	A	0.663	-0.042	0.010	0.000	0.018

**rs115694474**	T	A	0.810	0.066	0.012	0.001	0.021
**rs11644048**	A	G	0.575	-0.049	0.010	0.000	0.019
**rs116660876**	T	A	0.950	-0.085	0.021	0.035	0.040
**rs11679617**	C	T	0.610	0.048	0.010	0.054	0.017
**rs116981909**	G	A	0.974	-0.163	0.036	-0.169	0.056
**rs117003004**	C	T	0.988	-0.191	0.042	-0.078	0.080
**rs11737121**	G	A	0.850	0.057	0.013	0.004	0.024
**rs117394301**	C	G	0.982	0.191	0.045	-0.002	0.068
**rs11764361**	A	G	0.669	0.061	0.010	-0.044	0.019
**rs11766995**	C	T	0.977	-0.102	0.024	-0.074	0.064
**rs11785781**	A	G	0.119	-0.064	0.014	-0.026	0.026
**rs11788723**	T	C	0.850	-0.061	0.013	0.005	0.024
**rs118134876**	C	T	0.950	0.088	0.021	-0.023	0.041
**rs11858458**	A	G	0.098	-0.082	0.016	-0.053	0.028
**rs11858715**	C	T	0.762	0.053	0.012	-0.024	0.022
**rs11876196**	A	G	0.950	0.104	0.024	-0.010	0.040
**rs11877693**	G	C	0.900	0.069	0.016	-0.044	0.028
**rs11950745**	C	G	0.687	-0.043	0.010	-0.003	0.019
**rs11958187**	G	A	0.371	-0.050	0.010	-0.015	0.018
**rs11963899**	A	G	0.853	-0.065	0.013	0.013	0.025
**rs1204834**	T	A	0.452	0.045	0.009	-0.019	0.017
**rs12050642**	A	G	0.741	-0.050	0.011	-0.026	0.019
**rs12055890**	C	A	0.833	-0.067	0.013	-0.036	0.023
**rs12065220**	C	T	0.635	-0.048	0.010	0.015	0.018
**rs12133979**	C	T	0.970	-0.118	0.028	-0.064	0.057
**rs12134005**	C	T	0.881	0.077	0.016	0.027	0.029
**rs12233703**	A	T	0.335	-0.048	0.010	-0.009	0.018
**rs12323512**	G	A	0.584	-0.040	0.010	0.013	0.017
**rs12333212**	T	C	0.809	0.056	0.012	0.051	0.022
**rs12407573**	G	C	0.874	0.067	0.014	0.014	0.026
**rs12411130**	C	A	0.647	0.050	0.010	0.007	0.018
**rs12464279**	T	G	0.724	0.049	0.011	-0.007	0.019
**rs12491058**	G	A	0.744	0.044	0.011	0.046	0.020
**rs12544947**	A	T	0.660	0.044	0.010	-0.012	0.018
**rs12575685**	G	A	0.694	-0.065	0.010	0.003	0.019
**rs12600720**	C	G	0.683	-0.044	0.011	-0.036	0.020
**rs12603807**	T	C	0.664	0.046	0.010	0.029	0.018
**rs12644938**	T	C	0.565	-0.042	0.010	-0.017	0.017
**rs12668848**	G	A	0.579	0.057	0.010	0.029	0.017
**rs12675289**	A	C	0.735	0.048	0.011	0.023	0.019
**rs12729719**	A	G	0.790	0.054	0.011	0.029	0.021
**rs12764732**	C	T	0.955	-0.106	0.022	0.004	0.041
**rs12783919**	G	A	0.656	0.047	0.010	0.011	0.018
**rs12865195**	A	G	0.570	0.039	0.010	0.008	0.017
**rs12892189**	C	A	0.676	-0.049	0.010	0.002	0.018

**rs12919664**	T	C	0.729	-0.053	0.011	-0.015	0.019
**rs12932628**	G	T	0.501	-0.057	0.010	0.000	0.019
**rs1298062**	A	G	0.332	-0.041	0.010	0.004	0.018
**rs1299858**	T	C	0.242	0.048	0.011	-0.005	0.020
**rs13044225**	A	G	0.542	-0.055	0.010	0.016	0.017
**rs13049106**	G	A	0.964	0.114	0.027	0.060	0.045
**rs13085687**	C	A	0.543	-0.039	0.010	-0.045	0.017
**rs13133001**	T	C	0.961	-0.103	0.024	0.068	0.051
**rs13195402**	G	T	0.936	0.136	0.018	0.055	0.039
**rs13333582**	T	C	0.953	-0.097	0.023	0.009	0.040
**rs13353610**	T	C	0.622	-0.042	0.010	-0.003	0.018
**rs13404366**	A	G	0.323	0.051	0.010	0.064	0.018
**rs13405781**	G	A	0.835	0.054	0.013	0.012	0.025
**rs13417268**	C	G	0.766	0.062	0.011	-0.026	0.020
**rs139005549**	C	T	0.974	-0.116	0.028	0.023	0.061
**rs139129362**	G	A	0.986	-0.276	0.057	-0.001	0.095
**rs1391439**	G	A	0.385	0.039	0.010	-0.036	0.017
**rs140565076**	G	A	0.976	0.168	0.032	0.067	0.058
**rs143278184**	A	C	0.986	-0.140	0.033	-0.086	0.086
**rs144225206**	G	A	0.923	-0.077	0.017	-0.053	0.031
**rs145279901**	G	A	0.974	-0.125	0.031	0.013	0.055
**rs145316860**	T	C	0.991	-0.164	0.039	0.053	0.117
**rs145767750**	T	C	0.970	0.127	0.028	0.094	0.055
**rs146655022**	C	T	0.992	-0.258	0.053	-0.305	0.109
**rs146950761**	C	G	0.841	-0.065	0.013	-0.017	0.023
**rs1470308**	A	G	0.646	-0.045	0.010	-0.004	0.018
**rs1477105**	A	G	0.663	0.042	0.010	0.000	0.018
**rs1487445**	C	T	0.527	-0.074	0.009	0.016	0.017
**rs149002246**	C	G	0.962	0.132	0.025	-0.002	0.045
**rs149174768**	A	G	0.987	-0.148	0.035	0.146	0.099
**rs149305303**	A	T	0.983	-0.166	0.038	-0.076	0.077
**rs1533309**	A	G	0.469	-0.040	0.010	0.005	0.018
**rs1646022**	C	G	0.546	0.046	0.009	0.002	0.017
**rs1689956**	T	G	0.481	0.039	0.010	-0.013	0.017
**rs1695769**	G	A	0.889	-0.064	0.015	-0.019	0.027
**rs1711506**	T	C	0.260	0.045	0.011	0.039	0.020
**rs17155863**	A	G	0.896	0.062	0.015	0.008	0.028
**rs17183814**	G	A	0.925	0.103	0.019	0.015	0.033
**rs1737333**	T	C	0.262	-0.044	0.011	0.014	0.019
**rs174592**	A	G	0.635	-0.072	0.010	-0.065	0.018
**rs174927**	C	T	0.290	-0.048	0.010	0.010	0.019
**rs17680262**	C	T	0.923	-0.091	0.018	-0.003	0.034
**rs17680579**	A	G	0.872	0.066	0.014	0.024	0.025
**rs17705372**	A	G	0.889	-0.066	0.015	-0.006	0.027
**rs1846162**	G	A	0.567	0.041	0.010	0.001	0.017

**rs1875665**	A	G	0.978	-0.164	0.035	-0.070	0.059
**rs1890787**	T	C	0.441	0.039	0.010	-0.015	0.017
**rs1895538**	T	C	0.480	0.039	0.009	-0.023	0.017
**rs191139555**	T	A	0.986	0.145	0.036	0.139	0.088
**rs1925122**	T	C	0.675	-0.043	0.010	-0.002	0.018
**rs1955454**	T	G	0.431	-0.042	0.010	-0.003	0.017
**rs1959440**	G	T	0.573	-0.048	0.010	-0.009	0.017
**rs1969208**	A	G	0.725	-0.046	0.011	-0.018	0.019
**rs197120**	G	C	0.151	0.070	0.013	-0.019	0.024
**rs1998820**	T	A	0.896	0.084	0.015	0.020	0.028
**rs2006760**	C	G	0.787	-0.052	0.012	0.008	0.021
**rs2009336**	C	T	0.546	-0.045	0.011	-0.029	0.023
**rs2011302**	T	A	0.632	-0.053	0.010	-0.007	0.017
**rs2038061**	A	G	0.434	-0.051	0.010	-0.037	0.017
**rs2126180**	G	A	0.544	-0.057	0.009	0.003	0.017
**rs2127288**	A	G	0.005	0.207	0.049	0.054	0.268
**rs217619**	A	G	0.288	0.044	0.010	-0.026	0.019
**rs2254900**	G	A	0.811	-0.051	0.012	-0.006	0.021
**rs2271047**	A	G	0.588	-0.044	0.010	0.005	0.018
**rs2273738**	C	T	0.849	-0.092	0.014	-0.011	0.024
**rs2282551**	T	C	0.670	-0.041	0.010	0.000	0.018
**rs228768**	G	T	0.314	0.064	0.010	-0.025	0.019
**rs2299098**	G	C	0.815	-0.060	0.012	0.005	0.022
**rs2305929**	A	G	0.804	-0.064	0.012	-0.037	0.021
**rs2336147**	T	C	0.493	0.068	0.009	0.032	0.017
**rs2339519**	G	A	0.554	-0.049	0.009	0.001	0.017
**rs237460**	C	T	0.559	-0.055	0.009	0.019	0.017
**rs2484034**	G	A	0.727	-0.050	0.011	0.028	0.019
**rs2496038**	T	G	0.255	0.050	0.011	0.002	0.019
**rs2496543**	C	T	0.607	0.041	0.010	0.028	0.017
**rs251381**	A	G	0.348	-0.049	0.010	0.003	0.018
**rs2551976**	T	C	0.533	-0.040	0.009	-0.011	0.018
**rs255373**	T	A	0.536	-0.050	0.009	-0.032	0.017
**rs2622540**	A	G	0.341	-0.041	0.010	-0.029	0.018
**rs2635209**	T	C	0.516	-0.051	0.009	-0.027	0.017
**rs2650087**	G	C	0.501	0.044	0.010	0.018	0.017
**rs2651566**	G	A	0.600	0.044	0.010	-0.015	0.017
**rs2693698**	A	G	0.450	-0.053	0.009	-0.005	0.017
**rs2697136**	G	T	0.652	-0.042	0.010	-0.009	0.019
**rs27957**	A	G	0.739	-0.046	0.011	-0.031	0.019
**rs2807742**	G	A	0.217	0.051	0.011	-0.004	0.021
**rs2809444**	G	C	0.543	0.039	0.009	0.000	0.017
**rs2814736**	T	C	0.397	0.039	0.010	0.011	0.017
**rs2817332**	T	A	0.144	0.067	0.015	0.017	0.026
**rs28360326**	A	T	0.967	-0.210	0.039	0.104	0.061

**rs28377676**	G	A	0.781	0.061	0.012	0.000	0.022
**rs28455634**	G	A	0.607	0.063	0.010	-0.026	0.018
**rs28565152**	G	A	0.757	-0.067	0.011	0.007	0.020
**rs28568531**	T	A	0.683	-0.044	0.010	0.048	0.018
**rs28865701**	A	G	0.637	-0.044	0.010	-0.006	0.018
**rs28890251**	T	C	0.646	0.046	0.010	-0.014	0.018
**rs2953928**	G	A	0.951	-0.116	0.020	-0.064	0.038
**rs2960323**	C	G	0.834	0.055	0.013	0.030	0.023
**rs2962370**	G	C	0.356	0.051	0.010	0.011	0.018
**rs308800**	T	C	0.800	0.056	0.012	0.018	0.021
**rs3145**	G	A	0.232	-0.053	0.011	0.035	0.020
**rs318517**	C	T	0.838	0.067	0.013	0.009	0.023
**rs3208937**	A	G	0.268	0.045	0.010	-0.006	0.019
**rs324017**	A	C	0.290	-0.053	0.011	0.030	0.019
**rs34044430**	T	C	0.693	0.046	0.010	0.022	0.021
**rs34223888**	C	T	0.968	-0.151	0.037	-0.108	0.056
**rs34324305**	T	C	0.822	0.056	0.012	0.036	0.022
**rs345257**	G	A	0.174	-0.059	0.012	0.018	0.022
**rs34550586**	A	G	0.530	0.045	0.009	0.013	0.017
**rs34568676**	G	A	0.763	-0.049	0.011	-0.018	0.020
**rs34626151**	A	G	0.814	-0.056	0.012	-0.002	0.023
**rs348798**	G	A	0.351	-0.050	0.010	-0.036	0.018
**rs35058554**	G	T	0.956	-0.089	0.020	-0.032	0.044
**rs35082563**	A	C	0.720	-0.048	0.010	-0.001	0.019
**rs35090414**	G	T	0.920	0.083	0.017	-0.032	0.031
**rs35306827**	G	A	0.771	0.066	0.011	-0.011	0.020
**rs35343411**	G	A	0.934	-0.089	0.018	0.053	0.034
**rs35512633**	C	T	0.886	0.072	0.015	0.028	0.026
**rs358699**	T	C	0.845	0.061	0.013	-0.005	0.024
**rs35944620**	A	G	0.910	-0.069	0.016	0.009	0.030
**rs35958438**	G	A	0.784	0.064	0.012	0.038	0.021
**rs35985675**	T	A	0.806	0.055	0.012	0.033	0.022
**rs3775065**	G	A	0.910	-0.078	0.017	-0.005	0.031
**rs3776433**	A	C	0.329	-0.044	0.011	-0.012	0.018
**rs3780001**	T	G	0.678	-0.043	0.010	-0.012	0.018
**rs3795443**	A	G	0.864	-0.061	0.013	-0.026	0.024
**rs384182**	A	G	0.430	0.041	0.009	-0.029	0.017
**rs40948**	G	A	0.658	0.045	0.010	0.030	0.018
**rs4239191**	G	A	0.442	0.041	0.009	-0.002	0.018
**rs4331993**	T	A	0.669	-0.055	0.010	-0.002	0.018
**rs4357172**	G	A	0.754	-0.048	0.011	-0.025	0.019
**rs4376446**	A	T	0.725	-0.056	0.010	-0.017	0.019
**rs4447398**	A	C	0.135	0.082	0.014	0.033	0.024
**rs4489684**	C	G	0.243	0.050	0.011	-0.044	0.020
**rs4619651**	G	A	0.690	0.066	0.010	0.001	0.018

**rs4666995**	A	G	0.240	0.057	0.011	0.000	0.020
**rs4676412**	G	A	0.232	-0.061	0.011	-0.057	0.021
**rs4684787**	C	T	0.676	-0.044	0.010	-0.021	0.019
**rs4738519**	T	C	0.862	-0.065	0.014	-0.044	0.025
**rs4783319**	G	A	0.728	-0.049	0.011	-0.005	0.019
**rs4786364**	C	G	0.588	0.043	0.010	0.024	0.018
**rs4788865**	G	T	0.703	0.055	0.011	-0.007	0.019
**rs4790841**	C	T	0.845	-0.073	0.013	-0.024	0.026
**rs4794563**	G	A	0.621	-0.047	0.010	-0.015	0.017
**rs4845399**	C	T	0.637	-0.055	0.010	0.038	0.018
**rs4884464**	T	C	0.373	0.049	0.010	0.003	0.017
**rs4899437**	C	G	0.586	-0.042	0.010	0.001	0.017
**rs4905906**	T	G	0.308	0.051	0.010	0.011	0.018
**rs4916266**	C	A	0.594	-0.043	0.010	-0.033	0.017
**rs4941932**	A	G	0.213	0.050	0.011	0.010	0.021
**rs4949980**	A	G	0.936	0.085	0.019	0.016	0.036
**rs4964660**	A	T	0.291	0.049	0.011	-0.015	0.018
**rs512285**	T	C	0.434	0.043	0.009	0.024	0.017
**rs535066**	G	T	0.418	-0.045	0.009	-0.009	0.017
**rs55647329**	C	T	0.865	0.059	0.014	0.010	0.025
**rs55661361**	G	A	0.665	0.041	0.010	-0.002	0.018
**rs56014219**	A	T	0.820	-0.064	0.014	-0.008	0.025
**rs56108920**	A	G	0.435	0.043	0.010	-0.001	0.017
**rs56331084**	C	A	0.815	-0.058	0.012	0.017	0.022
**rs56351137**	C	T	0.941	-0.092	0.020	-0.028	0.040
**rs57116584**	C	T	0.637	-0.047	0.010	-0.008	0.017
**rs5758064**	T	C	0.515	0.052	0.009	0.024	0.017
**rs577027**	C	G	0.739	-0.049	0.011	-0.024	0.020
**rs581614**	A	C	0.527	0.045	0.010	-0.012	0.017
**rs58627659**	C	T	0.920	0.075	0.017	0.018	0.031
**rs588882**	A	G	0.444	0.041	0.010	-0.016	0.017
**rs59212827**	G	A	0.459	0.050	0.010	0.031	0.017
**rs59234174**	C	T	0.834	-0.055	0.013	-0.027	0.023
**rs59881258**	G	A	0.438	-0.042	0.010	-0.022	0.017
**rs6007473**	C	T	0.515	0.041	0.010	-0.006	0.017
**rs6010045**	T	C	0.302	-0.047	0.010	0.009	0.019
**rs60271**	A	C	0.320	0.052	0.010	0.008	0.018
**rs6074741**	T	C	0.720	-0.052	0.010	0.030	0.019
**rs614230**	C	T	0.363	-0.040	0.010	0.036	0.018
**rs61554907**	G	T	0.898	-0.087	0.015	0.052	0.030
**rs61774748**	T	G	0.736	-0.052	0.011	-0.012	0.020
**rs617760**	A	G	0.309	0.046	0.010	-0.010	0.018
**rs61933110**	G	A	0.892	-0.063	0.015	-0.036	0.031
**rs62029325**	C	T	0.894	-0.070	0.016	-0.033	0.028
**rs62087023**	T	C	0.721	0.049	0.011	0.017	0.019

**rs62151026**	C	G	0.598	-0.043	0.010	0.000	0.017
**rs62218838**	A	C	0.986	-0.196	0.047	0.107	0.110
**rs62234939**	T	C	0.505	-0.046	0.009	-0.041	0.017
**rs62489493**	C	G	0.872	-0.090	0.014	-0.026	0.025
**rs62581014**	C	T	0.624	-0.065	0.012	0.023	0.022
**rs645565**	C	T	0.898	-0.085	0.016	0.011	0.030
**rs6502721**	C	T	0.343	0.044	0.010	-0.024	0.019
**rs6572737**	C	A	0.824	-0.057	0.012	-0.004	0.022
**rs6580698**	T	C	0.571	-0.046	0.010	-0.009	0.017
**rs66550110**	G	A	0.942	0.098	0.020	-0.082	0.037
**rs66704380**	T	G	0.828	0.056	0.013	0.015	0.026
**rs6715448**	T	C	0.298	0.047	0.010	-0.006	0.018
**rs6721570**	C	T	0.824	-0.050	0.012	-0.018	0.022
**rs6746786**	C	T	0.907	0.072	0.017	-0.014	0.030
**rs6747175**	T	C	0.311	0.045	0.011	-0.010	0.019
**rs6748160**	C	T	0.357	-0.041	0.010	-0.018	0.018
**rs67506493**	A	G	0.881	-0.065	0.015	0.029	0.029
**rs67712855**	T	G	0.686	0.068	0.010	-0.035	0.018
**rs6783438**	G	A	0.644	0.043	0.010	-0.010	0.019
**rs683808**	A	G	0.463	-0.039	0.010	0.002	0.017
**rs6838474**	C	T	0.040	-0.115	0.024	0.032	0.043
**rs6883429**	C	T	0.181	0.058	0.013	0.024	0.023
**rs6887473**	G	A	0.715	0.060	0.011	0.017	0.019
**rs6946056**	A	C	0.392	-0.053	0.010	0.005	0.017
**rs6954854**	G	A	0.420	0.058	0.009	0.003	0.017
**rs6960056**	G	A	0.556	-0.040	0.009	0.000	0.017
**rs696366**	C	A	0.548	0.052	0.009	-0.008	0.017
**rs6992333**	A	G	0.565	-0.060	0.010	-0.008	0.017
**rs7001397**	C	T	0.533	-0.044	0.010	-0.012	0.017
**rs7007192**	C	A	0.784	0.052	0.012	0.035	0.021
**rs7108878**	T	G	0.888	-0.081	0.015	0.044	0.027
**rs7131187**	A	C	0.946	-0.117	0.028	0.057	0.049
**rs7143692**	A	G	0.780	-0.051	0.011	-0.017	0.021
**rs7160151**	A	G	0.697	0.044	0.010	-0.028	0.019
**rs7201930**	T	C	0.682	-0.059	0.010	0.035	0.018
**rs7235698**	A	G	0.495	-0.039	0.009	-0.024	0.017
**rs72725665**	G	A	0.920	0.071	0.017	-0.006	0.032
**rs72745470**	C	G	0.631	-0.050	0.010	-0.023	0.018
**rs72800727**	T	A	0.953	-0.101	0.022	-0.048	0.045
**rs72815749**	A	G	0.961	0.127	0.025	0.029	0.044
**rs72827147**	T	G	0.942	0.101	0.024	-0.162	0.046
**rs72827820**	C	T	0.754	0.048	0.011	-0.026	0.021
**rs72920394**	G	A	0.910	-0.084	0.016	-0.052	0.031
**rs73103717**	G	A	0.990	-0.191	0.042	-0.260	0.113
**rs73136039**	C	T	0.875	0.059	0.014	0.046	0.026

**rs73155124**	C	G	0.986	-0.183	0.041	0.043	0.090
**rs73206066**	C	T	0.985	-0.184	0.042	-0.099	0.074
**rs733760**	G	T	0.560	0.051	0.010	-0.004	0.017
**rs735931**	A	G	0.493	0.050	0.010	-0.018	0.018
**rs73923135**	C	A	0.835	-0.063	0.013	-0.031	0.023
**rs74109996**	C	T	0.935	-0.080	0.019	-0.064	0.034
**rs74840318**	C	T	0.986	-0.197	0.043	0.038	0.077
**rs748455**	T	C	0.696	0.067	0.010	0.000	0.018
**rs7520007**	C	A	0.487	-0.039	0.009	0.003	0.017
**rs7534376**	A	G	0.970	-0.164	0.037	0.005	0.053
**rs75438122**	T	C	0.963	0.112	0.024	-0.035	0.052
**rs7544805**	C	T	0.665	-0.043	0.010	-0.014	0.018
**rs75623709**	T	G	0.917	-0.074	0.016	-0.057	0.030
**rs7573275**	G	A	0.716	-0.050	0.010	-0.014	0.019
**rs75888683**	G	T	0.959	-0.117	0.022	0.031	0.044
**rs759073**	T	C	0.568	-0.051	0.011	0.030	0.020
**rs76076778**	A	T	0.975	0.146	0.035	0.030	0.070
**rs7609336**	G	A	0.617	0.051	0.010	0.009	0.017
**rs76187039**	G	T	0.874	0.063	0.013	0.026	0.026
**rs764240**	G	A	0.604	0.043	0.010	0.031	0.017
**rs76769832**	C	T	0.923	-0.088	0.018	-0.025	0.033
**rs76795191**	A	G	0.352	0.044	0.010	-0.028	0.018
**rs7684**	T	G	0.592	-0.040	0.010	-0.033	0.018
**rs7707252**	A	G	0.736	-0.057	0.010	-0.002	0.019
**rs7709645**	G	C	0.516	-0.038	0.009	0.015	0.017
**rs77426572**	C	G	0.961	-0.140	0.028	-0.018	0.057
**rs77926607**	A	G	0.985	0.154	0.037	0.061	0.071
**rs78104110**	C	T	0.963	0.132	0.026	-0.020	0.051
**rs7842666**	G	A	0.488	-0.041	0.009	-0.013	0.017
**rs7870016**	T	A	0.528	0.039	0.009	0.034	0.017
**rs78885330**	T	A	0.936	0.103	0.024	0.031	0.044
**rs7895364**	G	C	0.593	-0.049	0.009	0.026	0.017
**rs789865**	A	C	0.551	0.041	0.009	0.014	0.017
**rs79269869**	G	A	0.992	-0.195	0.047	0.205	0.120
**rs7937640**	A	T	0.426	-0.048	0.010	-0.026	0.017
**rs7940866**	T	A	0.510	0.047	0.010	0.008	0.017
**rs79434268**	G	A	0.926	-0.076	0.017	-0.006	0.032
**rs7947951**	A	G	0.325	-0.047	0.010	0.004	0.018
**rs79576701**	T	C	0.916	-0.079	0.017	-0.057	0.031
**rs7969100**	C	T	0.728	-0.046	0.011	-0.015	0.020
**rs7982263**	T	C	0.415	-0.052	0.010	0.008	0.017
**rs79917448**	A	G	0.833	0.055	0.013	-0.022	0.023
**rs8040193**	T	C	0.965	-0.116	0.027	-0.113	0.053
**rs8043792**	T	C	0.502	-0.045	0.009	-0.010	0.017
**rs8090457**	A	G	0.502	-0.050	0.009	-0.034	0.017

**rs8131986**	A	C	0.507	-0.048	0.009	0.035	0.017
**rs817492**	T	C	0.121	-0.064	0.014	0.001	0.027
**rs823104**	T	C	0.126	-0.058	0.014	-0.029	0.026
**rs82390**	T	C	0.488	-0.042	0.009	-0.011	0.017
**rs880447**	G	C	0.592	-0.053	0.011	-0.010	0.019
**rs880983**	G	A	0.979	0.169	0.037	0.048	0.074
**rs909775**	G	T	0.682	-0.042	0.010	0.016	0.018
**rs9389556**	C	G	0.739	-0.044	0.011	-0.001	0.019
**rs9389625**	T	G	0.583	-0.040	0.010	0.001	0.017
**rs9395495**	G	A	0.396	-0.048	0.010	-0.015	0.017
**rs9397125**	T	C	0.703	0.045	0.010	0.004	0.018
**rs942636**	C	T	0.827	-0.055	0.012	-0.047	0.023
**rs9529103**	G	A	0.359	0.044	0.010	0.018	0.018
**rs9562480**	T	C	0.966	-0.124	0.028	-0.044	0.049
**rs9638618**	G	A	0.290	-0.046	0.010	-0.004	0.018
**rs9644288**	C	G	0.562	-0.041	0.009	0.020	0.017
**rs9671125**	T	G	0.373	0.053	0.010	0.021	0.017
**rs9812434**	C	T	0.541	-0.047	0.009	-0.014	0.017
**rs9834970**	T	C	0.509	-0.083	0.009	-0.014	0.017
**rs9883919**	T	A	0.552	0.046	0.010	-0.017	0.017
**rs994280**	A	G	0.662	-0.050	0.010	-0.033	0.018
**rs9944**	A	G	0.226	-0.056	0.011	-0.008	0.022

Table S45. Raw data for the MR analysis of the causal effect of AN on IBD (P<5E-5)							
**SNP**	**Effect allele**	**Other allele**	**Effect allele frequency**	**Beta.exposure**	**Se.exposure**	**Beta.outcome**	**Se.outcome**
**rs10055754**	C	T	0.454	0.064	0.014	0.015	0.017
**rs10193235**	T	C	0.692	-0.061	0.015	0.029	0.019
**rs10422003**	A	T	0.808	-0.080	0.019	-0.033	0.023
**rs10747478**	T	G	0.423	0.076	0.014	0.018	0.017
**rs10788797**	G	A	0.131	-0.090	0.020	0.014	0.025
**rs10792434**	G	A	0.587	-0.057	0.014	-0.032	0.018
**rs1079314**	A	G	0.649	-0.058	0.014	0.002	0.018
**rs10882687**	G	A	0.218	0.076	0.017	-0.004	0.021
**rs10939796**	A	C	0.651	0.066	0.015	0.020	0.018
**rs11083128**	G	T	0.603	-0.062	0.014	0.026	0.017
**rs11105079**	T	C	0.970	0.216	0.052	-0.038	0.051
**rs11109807**	T	C	0.763	-0.066	0.016	0.001	0.020
**rs11126614**	G	A	0.745	-0.063	0.015	0.003	0.019
**rs11209815**	T	C	0.522	0.056	0.014	0.013	0.017
**rs11236814**	A	T	0.896	-0.120	0.024	0.056	0.031
**rs11245446**	A	G	0.666	0.079	0.017	0.019	0.018
**rs112502176**	G	T	0.779	-0.081	0.017	0.006	0.021
**rs113630856**	C	T	0.870	-0.090	0.022	0.004	0.025
**rs113645989**	C	T	0.987	-0.239	0.057	0.084	0.077
**rs113807441**	A	C	0.893	-0.092	0.022	-0.006	0.028
**rs114635345**	A	G	0.980	-0.224	0.051	-0.051	0.064
**rs11598668**	C	T	0.736	0.069	0.017	0.028	0.020
**rs11615526**	G	A	0.784	0.090	0.017	-0.009	0.021
**rs11627883**	T	C	0.554	0.075	0.017	-0.011	0.020
**rs116663873**	A	G	0.964	-0.173	0.040	0.046	0.050
**rs11672130**	G	A	0.943	-0.171	0.039	-0.071	0.042
**rs11673365**	G	T	0.834	-0.089	0.021	-0.014	0.025
**rs11680878**	T	C	0.664	-0.060	0.015	-0.014	0.018
**rs117230926**	C	A	0.978	-0.225	0.051	-0.052	0.061
**rs117289604**	G	T	0.957	-0.145	0.036	0.044	0.043
**rs11742575**	C	T	0.467	-0.055	0.013	0.026	0.017
**rs11784148**	C	A	0.201	0.075	0.018	0.045	0.023
**rs12315996**	C	T	0.805	-0.079	0.017	0.000	0.021
**rs12345267**	T	C	0.450	0.063	0.014	-0.004	0.017
**rs12349823**	C	T	0.518	-0.061	0.014	-0.006	0.017
**rs12401347**	A	G	0.891	-0.104	0.023	0.038	0.029
**rs12458700**	T	A	0.461	-0.071	0.017	0.021	0.019
**rs12477879**	C	T	0.847	0.076	0.019	-0.025	0.023
**rs12737676**	G	C	0.868	-0.090	0.022	-0.012	0.027
**rs12822914**	A	G	0.684	-0.067	0.015	0.000	0.019
**rs12826213**	C	T	0.484	0.069	0.014	0.015	0.017

**rs12939807**	A	G	0.541	-0.059	0.014	-0.015	0.017
**rs13100344**	T	A	0.540	0.075	0.014	-0.018	0.017
**rs13122802**	C	T	0.561	0.058	0.014	0.021	0.017
**rs13125932**	C	T	0.489	-0.072	0.013	0.001	0.017
**rs13251633**	T	A	0.389	-0.064	0.015	-0.014	0.018
**rs13397642**	G	A	0.972	0.212	0.048	0.009	0.053
**rs13422947**	G	A	0.818	0.088	0.019	-0.013	0.022
**rs1354060**	A	G	0.460	-0.059	0.014	0.002	0.017
**rs1373600**	C	T	0.760	-0.067	0.016	-0.013	0.020
**rs139681560**	G	A	0.981	-0.187	0.046	-0.116	0.062
**rs1405050**	C	T	0.489	-0.059	0.013	-0.002	0.017
**rs1417954**	T	C	0.405	-0.071	0.017	0.038	0.018
**rs142357293**	C	T	0.942	-0.198	0.049	0.015	0.041
**rs143272943**	C	T	0.954	0.136	0.032	0.019	0.040
**rs1442241**	A	G	0.072	0.107	0.025	-0.038	0.032
**rs146440771**	C	T	0.971	-0.201	0.048	-0.058	0.061
**rs146567439**	G	A	0.953	-0.132	0.031	0.048	0.045
**rs148094205**	C	T	0.969	-0.190	0.046	0.059	0.053
**rs150954178**	C	G	0.975	-0.167	0.041	-0.030	0.054
**rs1539725**	T	C	0.583	-0.073	0.014	-0.021	0.017
**rs1660242**	A	T	0.572	-0.057	0.014	-0.026	0.017
**rs16852686**	G	A	0.848	0.082	0.020	-0.031	0.024
**rs16963319**	T	G	0.919	0.105	0.025	0.011	0.031
**rs16965238**	A	G	0.857	-0.083	0.019	0.039	0.024
**rs17049722**	C	T	0.848	0.093	0.019	0.009	0.023
**rs17218035**	A	G	0.583	0.062	0.015	-0.016	0.017
**rs17447029**	G	A	0.716	-0.080	0.019	-0.016	0.020
**rs17452122**	T	C	0.823	0.086	0.019	0.017	0.023
**rs17580614**	A	G	0.757	-0.086	0.019	0.006	0.021
**rs17760820**	T	C	0.968	-0.170	0.039	0.001	0.049
**rs17842856**	T	C	0.753	0.077	0.017	-0.023	0.020
**rs1833556**	C	A	0.968	-0.184	0.041	-0.058	0.049
**rs188568019**	T	A	0.976	-0.203	0.046	-0.117	0.059
**rs189391781**	A	G	0.978	-0.239	0.051	-0.021	0.065
**rs189413073**	C	T	0.970	-0.241	0.055	-0.062	0.055
**rs1952966**	G	A	0.520	-0.060	0.014	-0.025	0.017
**rs2008387**	G	A	0.661	-0.082	0.015	-0.021	0.018
**rs2068447**	G	C	0.574	-0.055	0.014	-0.006	0.017
**rs2131959**	G	C	0.249	0.077	0.015	-0.051	0.020
**rs2173141**	T	C	0.388	0.062	0.014	0.024	0.017
**rs2178688**	G	C	0.399	0.063	0.014	-0.008	0.017
**rs2188244**	C	A	0.507	-0.068	0.014	0.017	0.017
**rs2287348**	C	T	0.838	-0.104	0.018	-0.033	0.023
**rs2531690**	A	C	0.304	-0.065	0.015	-0.013	0.019
**rs2565088**	C	T	0.357	0.058	0.014	0.004	0.018

**rs2750424**	T	A	0.665	0.064	0.014	-0.044	0.018
**rs2774074**	A	G	0.534	0.059	0.014	-0.007	0.018
**rs2821359**	T	C	0.777	-0.082	0.016	0.004	0.021
**rs28380**	G	C	0.424	-0.075	0.014	0.032	0.017
**rs2884594**	A	G	0.770	-0.087	0.018	-0.008	0.020
**rs3097882**	A	T	0.665	-0.063	0.015	0.030	0.019
**rs323509**	A	C	0.319	-0.060	0.015	0.007	0.018
**rs34029884**	A	C	0.726	-0.071	0.015	0.024	0.019
**rs34306607**	A	G	0.542	-0.065	0.014	-0.031	0.017
**rs35643176**	A	T	0.920	-0.125	0.026	0.012	0.033
**rs36075569**	A	G	0.622	0.060	0.014	0.013	0.017
**rs370838138**	G	C	0.577	0.075	0.014	-0.003	0.017
**rs3848726**	G	T	0.634	0.066	0.014	-0.027	0.018
**rs41284784**	A	G	0.990	-0.226	0.055	-0.095	0.093
**rs4129585**	A	C	0.441	0.059	0.013	-0.030	0.017
**rs41374645**	G	A	0.949	-0.133	0.031	0.031	0.038
**rs4145641**	A	C	0.747	0.075	0.016	-0.033	0.019
**rs4438286**	A	T	0.891	-0.099	0.024	0.005	0.028
**rs4556923**	C	T	0.867	-0.091	0.020	-0.015	0.026
**rs4651247**	G	A	0.629	-0.067	0.015	-0.026	0.018
**rs4751**	G	T	0.574	-0.082	0.017	-0.075	0.018
**rs4787855**	C	T	0.734	-0.065	0.015	-0.013	0.020
**rs4909316**	C	T	0.464	0.061	0.014	0.039	0.017
**rs55696831**	T	C	0.972	0.171	0.042	-0.033	0.051
**rs55780663**	T	C	0.956	-0.155	0.036	-0.067	0.041
**rs55906335**	C	A	0.961	0.161	0.038	0.071	0.043
**rs56248108**	G	C	0.986	-0.263	0.059	-0.076	0.078
**rs56345831**	G	A	0.815	0.084	0.019	0.012	0.024
**rs570333**	C	T	0.460	-0.065	0.014	-0.011	0.017
**rs5759292**	T	C	0.823	-0.075	0.018	-0.030	0.022
**rs58733813**	G	C	0.803	0.074	0.018	0.021	0.022
**rs58875646**	G	A	0.974	-0.215	0.050	0.015	0.056
**rs59388854**	C	T	0.862	-0.084	0.020	-0.003	0.025
**rs59416278**	G	A	0.919	-0.140	0.031	0.012	0.032
**rs60304095**	A	C	0.872	-0.088	0.021	-0.040	0.026
**rs6092932**	G	A	0.856	0.095	0.019	0.015	0.025
**rs61754806**	G	A	0.954	-0.156	0.033	-0.033	0.046
**rs62049002**	A	T	0.965	-0.241	0.054	0.058	0.049
**rs62083940**	G	T	0.988	-0.250	0.061	0.150	0.091
**rs62176532**	T	C	0.777	0.086	0.017	-0.033	0.020
**rs62425955**	G	A	0.867	-0.104	0.024	-0.052	0.026
**rs641325**	T	G	0.329	0.060	0.015	-0.039	0.018
**rs6452772**	T	C	0.792	-0.074	0.018	0.008	0.021
**rs6520157**	G	A	0.970	-0.180	0.038	-0.103	0.050
**rs6537828**	C	G	0.673	-0.059	0.014	0.004	0.018

**rs6589488**	A	T	0.143	0.127	0.020	0.015	0.024
**rs6687296**	G	A	0.876	0.092	0.022	0.017	0.027
**rs6727288**	T	C	0.561	-0.057	0.014	0.035	0.017
**rs6752750**	C	G	0.917	-0.125	0.028	0.000	0.034
**rs6765679**	C	T	0.772	-0.068	0.016	0.008	0.020
**rs6773424**	G	A	0.864	-0.091	0.020	-0.036	0.024
**rs67854953**	C	T	0.843	-0.094	0.021	-0.054	0.024
**rs6789500**	T	C	0.731	0.091	0.018	0.018	0.020
**rs6792954**	G	A	0.485	0.076	0.016	0.010	0.019
**rs6977110**	G	A	0.807	0.077	0.018	-0.017	0.021
**rs705696**	G	A	0.664	-0.064	0.014	-0.001	0.018
**rs7147039**	G	A	0.951	-0.165	0.039	-0.080	0.044
**rs725861**	A	G	0.816	0.088	0.018	-0.026	0.022
**rs72635674**	T	C	0.784	-0.080	0.017	0.018	0.021
**rs72709705**	C	A	0.983	-0.286	0.070	0.118	0.078
**rs72734967**	G	A	0.877	-0.113	0.024	0.008	0.026
**rs7300592**	G	A	0.864	-0.094	0.020	-0.039	0.025
**rs73090540**	A	G	0.750	0.078	0.018	-0.002	0.021
**rs7325923**	A	T	0.878	-0.092	0.022	0.039	0.027
**rs7329613**	A	G	0.523	0.061	0.014	0.001	0.017
**rs73320465**	T	C	0.948	-0.160	0.039	-0.002	0.040
**rs7461587**	C	G	0.323	0.061	0.014	0.015	0.018
**rs750350**	G	T	0.877	-0.111	0.021	0.033	0.026
**rs75335411**	A	G	0.924	0.120	0.028	-0.007	0.036
**rs75484398**	A	G	0.899	-0.098	0.024	-0.049	0.029
**rs75561792**	T	G	0.862	-0.085	0.020	0.001	0.025
**rs7559141**	T	A	0.985	-0.294	0.061	0.008	0.077
**rs75963814**	C	A	0.989	-0.250	0.059	-0.084	0.098
**rs7630377**	T	C	0.497	-0.061	0.013	0.031	0.017
**rs76382855**	T	C	0.927	-0.136	0.032	0.058	0.037
**rs76453976**	A	G	0.963	-0.164	0.036	-0.058	0.044
**rs77947356**	G	T	0.829	-0.087	0.020	-0.016	0.023
**rs7797895**	A	C	0.774	0.078	0.017	-0.022	0.020
**rs7801611**	A	C	0.679	-0.059	0.014	-0.011	0.018
**rs78440618**	A	T	0.903	-0.098	0.024	-0.063	0.029
**rs79333207**	A	G	0.979	-0.190	0.046	-0.021	0.064
**rs79630707**	T	G	0.988	-0.244	0.058	-0.116	0.085
**rs80259280**	G	A	0.943	-0.114	0.028	0.032	0.037
**rs8070063**	G	T	0.474	-0.075	0.014	0.004	0.017
**rs8101357**	G	T	0.935	-0.123	0.029	0.035	0.036
**rs900144**	C	T	0.446	0.060	0.014	0.007	0.017
**rs906313**	A	C	0.674	-0.064	0.014	-0.023	0.018
**rs9302300**	T	C	0.220	0.068	0.017	-0.013	0.020
**rs9309871**	C	A	0.771	-0.078	0.016	0.026	0.020
**rs9439752**	T	G	0.767	0.070	0.017	0.004	0.020

**rs9450288**	A	T	0.473	-0.056	0.014	-0.026	0.017
**rs9530551**	T	G	0.980	-0.228	0.055	-0.003	0.062
**rs9612027**	G	C	0.579	-0.060	0.014	-0.020	0.017
**rs9784437**	A	G	0.790	-0.083	0.017	0.013	0.021
**rs9821797**	T	A	0.875	-0.157	0.020	-0.158	0.025
**rs9874207**	T	C	0.492	-0.081	0.015	0.009	0.017
**rs9929084**	C	G	0.667	0.069	0.015	0.018	0.018
**rs9963279**	G	A	0.987	-0.257	0.057	-0.032	0.094

Table S46. Raw data for the MR analysis of the causal effect of AN on CD (P<5E-5)							
**SNP**	**Effect allele**	**Other allele**	**Effect allele frequency**	**Beta.exposure**	**Se.exposure**	**Beta.outcome**	**Se.outcome**
**rs10055754**	C	T	0.452	0.064	0.014	0.011	0.023
**rs10193235**	T	C	0.692	-0.061	0.015	-0.005	0.025
**rs10422003**	A	T	0.807	-0.080	0.019	-0.061	0.031
**rs10747478**	T	G	0.424	0.076	0.014	0.051	0.023
**rs10788797**	G	A	0.130	-0.090	0.020	0.046	0.034
**rs10792434**	G	A	0.587	-0.057	0.014	-0.055	0.024
**rs1079314**	A	G	0.652	-0.058	0.014	-0.002	0.025
**rs10882687**	G	A	0.213	0.076	0.017	0.017	0.028
**rs10939796**	A	C	0.647	0.066	0.015	0.031	0.025
**rs11083128**	G	T	0.598	-0.062	0.014	0.021	0.024
**rs11105079**	T	C	0.971	0.216	0.052	0.009	0.071
**rs11109807**	T	C	0.763	-0.066	0.016	0.016	0.027
**rs11126614**	G	A	0.745	-0.063	0.015	0.025	0.026
**rs11209815**	T	C	0.523	0.056	0.014	0.023	0.023
**rs11236814**	A	T	0.891	-0.120	0.024	0.038	0.042
**rs11245446**	A	G	0.659	0.079	0.017	0.028	0.025
**rs112502176**	G	T	0.779	-0.081	0.017	-0.016	0.029
**rs113630856**	C	T	0.869	-0.090	0.022	0.040	0.034
**rs113645989**	C	T	0.988	-0.239	0.057	-0.084	0.104
**rs113807441**	A	C	0.891	-0.092	0.022	-0.056	0.037
**rs114635345**	A	G	0.982	-0.224	0.051	-0.078	0.092
**rs11598668**	C	T	0.728	0.069	0.017	0.062	0.027
**rs11615526**	G	A	0.786	0.090	0.017	-0.010	0.028
**rs11627883**	T	C	0.555	0.075	0.017	0.035	0.027
**rs116663873**	A	G	0.963	-0.173	0.040	0.047	0.067
**rs11672130**	G	A	0.942	-0.171	0.039	0.007	0.057
**rs11673365**	G	T	0.838	-0.089	0.021	-0.002	0.034
**rs11680878**	T	C	0.664	-0.060	0.015	-0.001	0.024
**rs117230926**	C	A	0.978	-0.225	0.051	-0.086	0.084
**rs117289604**	G	T	0.956	-0.145	0.036	0.022	0.058
**rs11742575**	C	T	0.464	-0.055	0.013	0.029	0.023
**rs11784148**	C	A	0.197	0.075	0.018	0.072	0.032
**rs12315996**	C	T	0.806	-0.079	0.017	0.032	0.029
**rs12345267**	T	C	0.461	0.063	0.014	0.012	0.023
**rs12349823**	C	T	0.517	-0.061	0.014	-0.040	0.023
**rs12401347**	A	G	0.892	-0.104	0.023	0.022	0.041
**rs12458700**	T	A	0.457	-0.071	0.017	0.019	0.026
**rs12477879**	C	T	0.850	0.076	0.019	-0.031	0.032
**rs12737676**	G	C	0.867	-0.090	0.022	-0.014	0.036
**rs12822914**	A	G	0.684	-0.067	0.015	-0.020	0.026
**rs12826213**	C	T	0.482	0.069	0.014	0.025	0.024
**rs12939807**	A	G	0.543	-0.059	0.014	-0.044	0.023

**rs13100344**	T	A	0.543	0.075	0.014	0.006	0.023
**rs13122802**	C	T	0.561	0.058	0.014	0.002	0.023
**rs13125932**	C	T	0.486	-0.072	0.013	-0.023	0.023
**rs13251633**	T	A	0.390	-0.064	0.015	0.007	0.025
**rs13397642**	G	A	0.971	0.212	0.048	-0.034	0.072
**rs13422947**	G	A	0.819	0.088	0.019	-0.010	0.031
**rs1354060**	A	G	0.458	-0.059	0.014	0.008	0.023
**rs1373600**	C	T	0.760	-0.067	0.016	-0.006	0.028
**rs139681560**	G	A	0.983	-0.187	0.046	-0.069	0.087
**rs1405050**	C	T	0.477	-0.059	0.013	0.021	0.023
**rs1417954**	T	C	0.406	-0.071	0.017	0.055	0.025
**rs142357293**	C	T	0.941	-0.198	0.049	0.001	0.056
**rs143272943**	C	T	0.953	0.136	0.032	-0.041	0.054
**rs1442241**	A	G	0.073	0.107	0.025	-0.025	0.044
**rs146440771**	C	T	0.971	-0.201	0.048	0.006	0.084
**rs146567439**	G	A	0.954	-0.132	0.031	0.085	0.063
**rs148094205**	C	T	0.969	-0.190	0.046	0.026	0.072
**rs150954178**	C	G	0.976	-0.167	0.041	-0.081	0.074
**rs1539725**	T	C	0.584	-0.073	0.014	0.005	0.024
**rs1660242**	A	T	0.565	-0.057	0.014	-0.010	0.023
**rs16852686**	G	A	0.847	0.082	0.020	-0.043	0.032
**rs16963319**	T	G	0.919	0.105	0.025	-0.041	0.041
**rs16965238**	A	G	0.857	-0.083	0.019	0.028	0.033
**rs17049722**	C	T	0.846	0.093	0.019	0.019	0.032
**rs17218035**	A	G	0.588	0.062	0.015	-0.030	0.023
**rs17447029**	G	A	0.720	-0.080	0.019	-0.002	0.027
**rs17452122**	T	C	0.822	0.086	0.019	-0.008	0.031
**rs17580614**	A	G	0.753	-0.086	0.019	-0.010	0.029
**rs17760820**	T	C	0.969	-0.170	0.039	0.023	0.068
**rs17842856**	T	C	0.758	0.077	0.017	-0.006	0.027
**rs1833556**	C	A	0.969	-0.184	0.041	-0.036	0.067
**rs188568019**	T	A	0.976	-0.203	0.046	-0.011	0.082
**rs189391781**	A	G	0.978	-0.239	0.051	-0.056	0.090
**rs189413073**	C	T	0.969	-0.241	0.055	-0.098	0.072
**rs1952966**	G	A	0.516	-0.060	0.014	-0.067	0.023
**rs2008387**	G	A	0.660	-0.082	0.015	-0.046	0.024
**rs2068447**	G	C	0.572	-0.055	0.014	0.021	0.023
**rs2131959**	G	C	0.240	0.077	0.015	-0.044	0.027
**rs2173141**	T	C	0.389	0.062	0.014	0.016	0.023
**rs2178688**	G	C	0.395	0.063	0.014	0.000	0.024
**rs2188244**	C	A	0.500	-0.068	0.014	0.017	0.023
**rs2287348**	C	T	0.838	-0.104	0.018	-0.051	0.030
**rs2531690**	A	C	0.303	-0.065	0.015	-0.008	0.025
**rs2565088**	C	T	0.358	0.058	0.014	0.004	0.024
**rs2750424**	T	A	0.667	0.064	0.014	-0.058	0.024

**rs2774074**	A	G	0.529	0.059	0.014	0.011	0.025
**rs2821359**	T	C	0.777	-0.082	0.016	0.047	0.028
**rs28380**	G	C	0.431	-0.075	0.014	0.026	0.024
**rs2884594**	A	G	0.771	-0.087	0.018	0.008	0.028
**rs3097882**	A	T	0.667	-0.063	0.015	0.016	0.027
**rs323509**	A	C	0.316	-0.060	0.015	-0.026	0.025
**rs34029884**	A	C	0.723	-0.071	0.015	-0.015	0.026
**rs34306607**	A	G	0.548	-0.065	0.014	-0.035	0.023
**rs35643176**	A	T	0.920	-0.125	0.026	-0.029	0.044
**rs36075569**	A	G	0.622	0.060	0.014	0.004	0.024
**rs370838138**	G	C	0.581	0.075	0.014	-0.020	0.023
**rs3848726**	G	T	0.637	0.066	0.014	-0.037	0.024
**rs41284784**	A	G	0.990	-0.226	0.055	-0.096	0.128
**rs4129585**	A	C	0.442	0.059	0.013	-0.036	0.023
**rs41374645**	G	A	0.949	-0.133	0.031	0.014	0.052
**rs4145641**	A	C	0.744	0.075	0.016	-0.007	0.026
**rs4438286**	A	T	0.886	-0.099	0.024	-0.008	0.037
**rs4556923**	C	T	0.868	-0.091	0.020	0.017	0.035
**rs4651247**	G	A	0.628	-0.067	0.015	-0.014	0.024
**rs4751**	G	T	0.575	-0.082	0.017	-0.075	0.025
**rs4787855**	C	T	0.739	-0.065	0.015	-0.047	0.028
**rs4909316**	C	T	0.465	0.061	0.014	0.015	0.023
**rs55696831**	T	C	0.972	0.171	0.042	-0.060	0.070
**rs55780663**	T	C	0.955	-0.155	0.036	-0.085	0.055
**rs55906335**	C	A	0.960	0.161	0.038	0.023	0.058
**rs56248108**	G	C	0.987	-0.263	0.059	0.042	0.115
**rs56345831**	G	A	0.819	0.084	0.019	0.024	0.034
**rs570333**	C	T	0.463	-0.065	0.014	-0.001	0.023
**rs5759292**	T	C	0.826	-0.075	0.018	-0.017	0.031
**rs58733813**	G	C	0.807	0.074	0.018	0.027	0.030
**rs58875646**	G	A	0.972	-0.215	0.050	0.032	0.076
**rs59388854**	C	T	0.865	-0.084	0.020	0.019	0.035
**rs59416278**	G	A	0.918	-0.140	0.031	0.058	0.043
**rs60304095**	A	C	0.870	-0.088	0.021	-0.007	0.035
**rs6092932**	G	A	0.858	0.095	0.019	-0.011	0.034
**rs61754806**	G	A	0.955	-0.156	0.033	-0.011	0.065
**rs62049002**	A	T	0.963	-0.241	0.054	0.073	0.066
**rs62083940**	G	T	0.987	-0.250	0.061	0.010	0.122
**rs62176532**	T	C	0.777	0.086	0.017	-0.043	0.028
**rs62425955**	G	A	0.864	-0.104	0.024	-0.046	0.034
**rs641325**	T	G	0.323	0.060	0.015	-0.044	0.025
**rs6452772**	T	C	0.783	-0.074	0.018	0.035	0.028
**rs6520157**	G	A	0.970	-0.180	0.038	-0.100	0.068
**rs6537828**	C	G	0.668	-0.059	0.014	-0.023	0.024
**rs6589488**	A	T	0.142	0.127	0.020	0.032	0.033

**rs6687296**	G	A	0.877	0.092	0.022	-0.048	0.037
**rs6727288**	T	C	0.560	-0.057	0.014	0.061	0.024
**rs6752750**	C	G	0.918	-0.125	0.028	0.015	0.047
**rs6765679**	C	T	0.770	-0.068	0.016	-0.018	0.027
**rs6773424**	G	A	0.864	-0.091	0.020	-0.048	0.033
**rs67854953**	C	T	0.845	-0.094	0.021	-0.030	0.032
**rs6789500**	T	C	0.731	0.091	0.018	0.010	0.027
**rs6792954**	G	A	0.483	0.076	0.016	-0.003	0.025
**rs6977110**	G	A	0.806	0.077	0.018	-0.020	0.029
**rs705696**	G	A	0.661	-0.064	0.014	-0.008	0.025
**rs7147039**	G	A	0.953	-0.165	0.039	-0.086	0.060
**rs725861**	A	G	0.820	0.088	0.018	-0.050	0.029
**rs72635674**	T	C	0.782	-0.080	0.017	0.012	0.028
**rs72709705**	C	A	0.984	-0.286	0.070	-0.011	0.110
**rs72734967**	G	A	0.876	-0.113	0.024	0.028	0.036
**rs7300592**	G	A	0.863	-0.094	0.020	-0.024	0.033
**rs73090540**	A	G	0.751	0.078	0.018	0.006	0.029
**rs7325923**	A	T	0.875	-0.092	0.022	0.032	0.036
**rs7329613**	A	G	0.520	0.061	0.014	-0.012	0.023
**rs73320465**	T	C	0.948	-0.160	0.039	-0.030	0.053
**rs7461587**	C	G	0.324	0.061	0.014	-0.007	0.024
**rs750350**	G	T	0.881	-0.111	0.021	0.075	0.036
**rs75335411**	A	G	0.923	0.120	0.028	-0.001	0.048
**rs75484398**	A	G	0.897	-0.098	0.024	0.024	0.040
**rs75561792**	T	G	0.863	-0.085	0.020	0.006	0.034
**rs7559141**	T	A	0.986	-0.294	0.061	0.148	0.111
**rs75963814**	C	A	0.989	-0.250	0.059	-0.183	0.137
**rs7630377**	T	C	0.491	-0.061	0.013	0.015	0.023
**rs76382855**	T	C	0.927	-0.136	0.032	0.022	0.049
**rs76453976**	A	G	0.961	-0.164	0.036	-0.066	0.059
**rs77947356**	G	T	0.829	-0.087	0.020	-0.003	0.031
**rs7797895**	A	C	0.774	0.078	0.017	-0.010	0.027
**rs7801611**	A	C	0.679	-0.059	0.014	-0.016	0.024
**rs78440618**	A	T	0.906	-0.098	0.024	-0.073	0.039
**rs79333207**	A	G	0.978	-0.190	0.046	-0.102	0.087
**rs79630707**	T	G	0.987	-0.244	0.058	-0.091	0.112
**rs80259280**	G	A	0.944	-0.114	0.028	0.042	0.051
**rs8070063**	G	T	0.483	-0.075	0.014	0.009	0.023
**rs8101357**	G	T	0.929	-0.123	0.029	0.024	0.048
**rs900144**	C	T	0.450	0.060	0.014	0.047	0.023
**rs906313**	A	C	0.675	-0.064	0.014	-0.012	0.025
**rs9302300**	T	C	0.219	0.068	0.017	0.030	0.028
**rs9309871**	C	A	0.775	-0.078	0.016	0.003	0.027
**rs9439752**	T	G	0.764	0.070	0.017	0.022	0.027
**rs9450288**	A	T	0.475	-0.056	0.014	-0.025	0.023

**rs9530551**	T	G	0.979	-0.228	0.055	-0.072	0.082
**rs9612027**	G	C	0.574	-0.060	0.014	-0.033	0.024
**rs9784437**	A	G	0.791	-0.083	0.017	0.030	0.028
**rs9821797**	T	A	0.874	-0.157	0.020	-0.149	0.034
**rs9874207**	T	C	0.490	-0.081	0.015	-0.019	0.024
**rs9929084**	C	G	0.670	0.069	0.015	0.032	0.025
**rs9963279**	G	A	0.987	-0.257	0.057	-0.087	0.127

Table S47. Raw data for the MR analysis of the causal effect of AN on UC (P<5E-5)							
SNP	Effect allele	Other allele	Effect allele frequency	Beta.exposure	Se.exposure	Beta.outcome	Se.outcome
**rs10055754**	C	T	0.453	0.064	0.014	0.020	0.021
**rs10193235**	T	C	0.692	-0.061	0.015	0.039	0.023
**rs10422003**	A	T	0.811	-0.080	0.019	-0.019	0.028
**rs10747478**	T	G	0.421	0.076	0.014	-0.006	0.021
**rs10788797**	G	A	0.132	-0.090	0.020	-0.007	0.032
**rs10792434**	G	A	0.589	-0.057	0.014	-0.011	0.023
**rs1079314**	A	G	0.649	-0.058	0.014	0.007	0.023
**rs10882687**	G	A	0.219	0.076	0.017	-0.018	0.026
**rs10939796**	A	C	0.652	0.066	0.015	0.007	0.023
**rs11083128**	G	T	0.603	-0.062	0.014	0.022	0.022
**rs11105079**	T	C	0.969	0.216	0.052	-0.095	0.062
**rs11109807**	T	C	0.762	-0.066	0.016	0.002	0.025
**rs11126614**	G	A	0.744	-0.063	0.015	-0.017	0.024
**rs11209815**	T	C	0.520	0.056	0.014	0.013	0.021
**rs11236814**	A	T	0.897	-0.120	0.024	0.075	0.039
**rs11245446**	A	G	0.668	0.079	0.017	0.004	0.023
**rs112502176**	G	T	0.780	-0.081	0.017	0.029	0.027
**rs113630856**	C	T	0.869	-0.090	0.022	-0.035	0.031
**rs113645989**	C	T	0.987	-0.239	0.057	0.116	0.098
**rs113807441**	A	C	0.895	-0.092	0.022	0.013	0.035
**rs114635345**	A	G	0.979	-0.224	0.051	-0.069	0.078
**rs11598668**	C	T	0.738	0.069	0.017	0.004	0.025
**rs11615526**	G	A	0.785	0.090	0.017	-0.016	0.026
**rs11627883**	T	C	0.551	0.075	0.017	-0.033	0.026
**rs116663873**	A	G	0.964	-0.173	0.040	0.035	0.064
**rs11672130**	G	A	0.942	-0.171	0.039	-0.134	0.053
**rs11673365**	G	T	0.832	-0.089	0.021	-0.008	0.031
**rs11680878**	T	C	0.663	-0.060	0.015	-0.030	0.022
**rs117230926**	C	A	0.978	-0.225	0.051	-0.046	0.077
**rs117289604**	G	T	0.957	-0.145	0.036	0.063	0.055
**rs11742575**	C	T	0.467	-0.055	0.013	0.019	0.021
**rs11784148**	C	A	0.200	0.075	0.018	0.044	0.029
**rs12315996**	C	T	0.805	-0.079	0.017	-0.020	0.027
**rs12345267**	T	C	0.447	0.063	0.014	-0.021	0.021
**rs12349823**	C	T	0.519	-0.061	0.014	0.011	0.021
**rs12401347**	A	G	0.890	-0.104	0.023	0.039	0.035
**rs12458700**	T	A	0.463	-0.071	0.017	0.039	0.024
**rs12477879**	C	T	0.847	0.076	0.019	-0.010	0.029
**rs12737676**	G	C	0.869	-0.090	0.022	0.001	0.034
**rs12822914**	A	G	0.684	-0.067	0.015	0.008	0.024
**rs12826213**	C	T	0.487	0.069	0.014	0.011	0.021
**rs12939807**	A	G	0.542	-0.059	0.014	0.010	0.022

**rs13100344**	T	A	0.539	0.075	0.014	-0.035	0.021
**rs13122802**	C	T	0.560	0.058	0.014	0.029	0.021
**rs13125932**	C	T	0.491	-0.072	0.013	0.022	0.021
**rs13251633**	T	A	0.387	-0.064	0.015	-0.029	0.023
**rs13397642**	G	A	0.972	0.212	0.048	0.025	0.067
**rs13422947**	G	A	0.818	0.088	0.019	-0.017	0.028
**rs1354060**	A	G	0.460	-0.059	0.014	0.009	0.021
**rs1373600**	C	T	0.760	-0.067	0.016	-0.029	0.026
**rs139681560**	G	A	0.981	-0.187	0.046	-0.122	0.076
**rs1405050**	C	T	0.493	-0.059	0.013	-0.015	0.021
**rs1417954**	T	C	0.402	-0.071	0.017	0.026	0.023
**rs142357293**	C	T	0.942	-0.198	0.049	-0.015	0.052
**rs143272943**	C	T	0.954	0.136	0.032	0.067	0.051
**rs1442241**	A	G	0.072	0.107	0.025	-0.037	0.041
**rs146440771**	C	T	0.971	-0.201	0.048	-0.037	0.076
**rs146567439**	G	A	0.952	-0.132	0.031	0.050	0.055
**rs148094205**	C	T	0.969	-0.190	0.046	0.079	0.068
**rs150954178**	C	G	0.975	-0.167	0.041	0.017	0.068
**rs1539725**	T	C	0.583	-0.073	0.014	-0.033	0.022
**rs1660242**	A	T	0.574	-0.057	0.014	-0.034	0.022
**rs16852686**	G	A	0.849	0.082	0.020	-0.024	0.030
**rs16963319**	T	G	0.920	0.105	0.025	0.044	0.039
**rs16965238**	A	G	0.857	-0.083	0.019	0.044	0.030
**rs17049722**	C	T	0.849	0.093	0.019	-0.011	0.030
**rs17218035**	A	G	0.582	0.062	0.015	-0.005	0.022
**rs17447029**	G	A	0.715	-0.080	0.019	-0.019	0.025
**rs17452122**	T	C	0.824	0.086	0.019	0.016	0.028
**rs17580614**	A	G	0.759	-0.086	0.019	-0.003	0.026
**rs17760820**	T	C	0.967	-0.170	0.039	-0.007	0.061
**rs17842856**	T	C	0.750	0.077	0.017	-0.020	0.024
**rs1833556**	C	A	0.968	-0.184	0.041	-0.020	0.061
**rs188568019**	T	A	0.976	-0.203	0.046	-0.201	0.073
**rs189391781**	A	G	0.978	-0.239	0.051	0.045	0.082
**rs189413073**	C	T	0.970	-0.241	0.055	-0.046	0.070
**rs1952966**	G	A	0.523	-0.060	0.014	0.017	0.021
**rs2008387**	G	A	0.664	-0.082	0.015	-0.009	0.022
**rs2068447**	G	C	0.574	-0.055	0.014	-0.013	0.021
**rs2131959**	G	C	0.255	0.077	0.015	-0.049	0.025
**rs2173141**	T	C	0.387	0.062	0.014	0.033	0.022
**rs2178688**	G	C	0.401	0.063	0.014	0.001	0.022
**rs2188244**	C	A	0.511	-0.068	0.014	0.013	0.021
**rs2287348**	C	T	0.839	-0.104	0.018	-0.017	0.029
**rs2531690**	A	C	0.303	-0.065	0.015	-0.002	0.023
**rs2565088**	C	T	0.356	0.058	0.014	0.002	0.022
**rs2750424**	T	A	0.665	0.064	0.014	-0.039	0.022

**rs2774074**	A	G	0.536	0.059	0.014	-0.026	0.023
**rs2821359**	T	C	0.777	-0.082	0.016	-0.015	0.026
**rs28380**	G	C	0.420	-0.075	0.014	0.039	0.022
**rs2884594**	A	G	0.769	-0.087	0.018	-0.012	0.026
**rs3097882**	A	T	0.665	-0.063	0.015	0.025	0.024
**rs323509**	A	C	0.322	-0.060	0.015	0.026	0.023
**rs34029884**	A	C	0.727	-0.071	0.015	0.045	0.024
**rs34306607**	A	G	0.540	-0.065	0.014	-0.015	0.021
**rs35643176**	A	T	0.921	-0.125	0.026	0.031	0.041
**rs36075569**	A	G	0.621	0.060	0.014	0.022	0.022
**rs370838138**	G	C	0.576	0.075	0.014	0.007	0.021
**rs3848726**	G	T	0.634	0.066	0.014	-0.024	0.022
**rs41284784**	A	G	0.989	-0.226	0.055	-0.094	0.116
**rs4129585**	A	C	0.443	0.059	0.013	-0.012	0.021
**rs41374645**	G	A	0.949	-0.133	0.031	0.080	0.049
**rs4145641**	A	C	0.748	0.075	0.016	-0.050	0.024
**rs4438286**	A	T	0.894	-0.099	0.024	0.033	0.036
**rs4556923**	C	T	0.867	-0.091	0.020	-0.037	0.032
**rs4651247**	G	A	0.629	-0.067	0.015	-0.024	0.022
**rs4751**	G	T	0.574	-0.082	0.017	-0.061	0.023
**rs4787855**	C	T	0.734	-0.065	0.015	0.011	0.025
**rs4909316**	C	T	0.464	0.061	0.014	0.053	0.021
**rs55696831**	T	C	0.972	0.171	0.042	-0.012	0.065
**rs55780663**	T	C	0.957	-0.155	0.036	-0.103	0.052
**rs55906335**	C	A	0.961	0.161	0.038	0.086	0.055
**rs56248108**	G	C	0.986	-0.263	0.059	-0.139	0.094
**rs56345831**	G	A	0.813	0.084	0.019	-0.002	0.030
**rs570333**	C	T	0.457	-0.065	0.014	-0.015	0.022
**rs5759292**	T	C	0.823	-0.075	0.018	-0.043	0.028
**rs58733813**	G	C	0.801	0.074	0.018	0.009	0.027
**rs58875646**	G	A	0.974	-0.215	0.050	0.030	0.071
**rs59388854**	C	T	0.859	-0.084	0.020	-0.010	0.031
**rs59416278**	G	A	0.918	-0.140	0.031	-0.011	0.040
**rs60304095**	A	C	0.873	-0.088	0.021	-0.055	0.032
**rs6092932**	G	A	0.855	0.095	0.019	0.030	0.031
**rs61754806**	G	A	0.953	-0.156	0.033	-0.039	0.055
**rs62049002**	A	T	0.966	-0.241	0.054	-0.012	0.063
**rs62083940**	G	T	0.988	-0.250	0.061	0.250	0.116
**rs62176532**	T	C	0.778	0.086	0.017	-0.021	0.026
**rs62425955**	G	A	0.868	-0.104	0.024	-0.037	0.033
**rs641325**	T	G	0.331	0.060	0.015	-0.031	0.023
**rs6452772**	T	C	0.795	-0.074	0.018	-0.005	0.027
**rs6520157**	G	A	0.970	-0.180	0.038	-0.101	0.062
**rs6537828**	C	G	0.676	-0.059	0.014	0.027	0.023
**rs6589488**	A	T	0.143	0.127	0.020	0.017	0.030

**rs6687296**	G	A	0.875	0.092	0.022	0.072	0.034
**rs6727288**	T	C	0.560	-0.057	0.014	0.003	0.022
**rs6752750**	C	G	0.917	-0.125	0.028	-0.017	0.042
**rs6765679**	C	T	0.773	-0.068	0.016	0.026	0.026
**rs6773424**	G	A	0.865	-0.091	0.020	-0.038	0.031
**rs67854953**	C	T	0.843	-0.094	0.021	-0.081	0.030
**rs6789500**	T	C	0.733	0.091	0.018	0.009	0.025
**rs6792954**	G	A	0.483	0.076	0.016	-0.003	0.023
**rs6977110**	G	A	0.809	0.077	0.018	-0.026	0.027
**rs705696**	G	A	0.664	-0.064	0.014	0.002	0.023
**rs7147039**	G	A	0.952	-0.165	0.039	-0.064	0.054
**rs725861**	A	G	0.814	0.088	0.018	-0.010	0.027
**rs72635674**	T	C	0.786	-0.080	0.017	0.027	0.026
**rs72709705**	C	A	0.983	-0.286	0.070	0.185	0.097
**rs72734967**	G	A	0.876	-0.113	0.024	-0.017	0.033
**rs7300592**	G	A	0.865	-0.094	0.020	-0.040	0.031
**rs73090540**	A	G	0.751	0.078	0.018	-0.009	0.027
**rs7325923**	A	T	0.879	-0.092	0.022	0.029	0.034
**rs7329613**	A	G	0.525	0.061	0.014	0.001	0.021
**rs73320465**	T	C	0.949	-0.160	0.039	-0.011	0.049
**rs7461587**	C	G	0.322	0.061	0.014	0.029	0.022
**rs750350**	G	T	0.875	-0.111	0.021	0.000	0.032
**rs75335411**	A	G	0.925	0.120	0.028	-0.004	0.045
**rs75484398**	A	G	0.899	-0.098	0.024	-0.093	0.037
**rs75561792**	T	G	0.862	-0.085	0.020	0.003	0.031
**rs7559141**	T	A	0.985	-0.294	0.061	-0.079	0.093
**rs75963814**	C	A	0.989	-0.250	0.059	-0.037	0.119
**rs7630377**	T	C	0.497	-0.061	0.013	0.036	0.021
**rs76382855**	T	C	0.928	-0.136	0.032	0.087	0.047
**rs76453976**	A	G	0.963	-0.164	0.036	-0.044	0.055
**rs77947356**	G	T	0.829	-0.087	0.020	-0.026	0.028
**rs7797895**	A	C	0.773	0.078	0.017	-0.031	0.025
**rs7801611**	A	C	0.679	-0.059	0.014	-0.010	0.023
**rs78440618**	A	T	0.902	-0.098	0.024	-0.067	0.036
**rs79333207**	A	G	0.979	-0.190	0.046	0.028	0.079
**rs79630707**	T	G	0.987	-0.244	0.058	-0.061	0.109
**rs80259280**	G	A	0.942	-0.114	0.028	0.036	0.045
**rs8070063**	G	T	0.470	-0.075	0.014	-0.005	0.022
**rs8101357**	G	T	0.938	-0.123	0.029	0.054	0.046
**rs900144**	C	T	0.445	0.060	0.014	-0.022	0.021
**rs906313**	A	C	0.676	-0.064	0.014	-0.031	0.023
**rs9302300**	T	C	0.218	0.068	0.017	-0.030	0.026
**rs9309871**	C	A	0.769	-0.078	0.016	0.038	0.025
**rs9439752**	T	G	0.770	0.070	0.017	-0.020	0.025
**rs9450288**	A	T	0.474	-0.056	0.014	-0.037	0.022

**rs9530551**	T	G	0.980	-0.228	0.055	0.013	0.080
**rs9612027**	G	C	0.578	-0.060	0.014	0.006	0.022
**rs9784437**	A	G	0.790	-0.083	0.017	0.006	0.026
**rs9821797**	T	A	0.878	-0.157	0.020	-0.160	0.032
**rs9874207**	T	C	0.495	-0.081	0.015	0.011	0.022
**rs9929084**	C	G	0.667	0.069	0.015	0.013	0.023
**rs9963279**	G	A	0.987	-0.257	0.057	0.005	0.120

Table S48. Raw data for the MR analysis of the causal effect of MDD on IBD (P<5E-5)							
SNP	Effect allele	Other allele	Effect allele frequency	Beta.exposure	Se.exposure	Beta.outcome	Se.outcome
**rs10235395**	A	G	0.69	-0.037	0.009	0	0.018
**rs10411666**	C	G	0.214	0.053	0.013	-0.022	0.029
**rs10759934**	A	T	0.497	0.036	0.008	-0.02	0.017
**rs10782599**	C	G	0.844	0.045	0.011	0.032	0.024
**rs10813253**	T	C	0.908	-0.061	0.014	0.042	0.033
**rs10825942**	T	G	0.455	-0.044	0.008	-0.008	0.017
**rs10861729**	T	C	0.316	-0.039	0.008	0.003	0.018
**rs1086490**	A	G	0.887	0.058	0.014	-0.017	0.027
**rs1095163**	A	G	0.454	0.033	0.008	-0.015	0.017
**rs10987923**	A	G	0.323	0.035	0.009	-0.009	0.019
**rs1106235**	A	G	0.198	0.05	0.012	0.009	0.022
**rs11077049**	A	G	0.022	0.139	0.03	0.042	0.06
**rs11121207**	A	C	0.197	0.043	0.01	0.021	0.021
**rs11192230**	A	T	0.738	0.041	0.009	0.001	0.019
**rs11220476**	T	C	0.163	-0.048	0.012	0.019	0.023
**rs112379491**	A	G	0.823	-0.044	0.011	-0.014	0.022
**rs113021645**	A	C	0.021	0.123	0.03	-0.099	0.068
**rs114923224**	T	C	0.062	0.072	0.015	0.024	0.035
**rs1152589**	A	T	0.479	-0.041	0.009	0.012	0.017
**rs115776708**	T	C	0.033	0.121	0.029	-0.084	0.061
**rs11590766**	T	C	0.567	0.036	0.008	-0.011	0.017
**rs116070829**	T	C	0.018	0.149	0.035	-0.025	0.072
**rs117141160**	A	G	0.014	0.249	0.056	-0.097	0.094
**rs11725076**	A	T	0.762	-0.053	0.012	0.021	0.022
**rs11789013**	T	C	0.752	-0.042	0.009	-0.045	0.02
**rs117901488**	T	C	0.043	0.095	0.021	0.025	0.048
**rs11852819**	T	C	0.017	0.14	0.033	0.05	0.072
**rs11945613**	A	G	0.03	0.115	0.027	-0.053	0.051
**rs11980163**	A	G	0.975	-0.111	0.027	-0.023	0.059
**rs12129573**	A	C	0.376	0.048	0.008	-0.021	0.017
**rs1214608**	A	T	0.562	0.035	0.008	-0.007	0.017
**rs12200766**	A	G	0.758	0.047	0.009	0.031	0.02
**rs12552**	A	G	0.434	0.04	0.008	0.012	0.017
**rs12666117**	A	G	0.473	0.034	0.008	0.02	0.017
**rs12700978**	T	C	0.545	0.034	0.008	-0.024	0.017
**rs13171899**	A	G	0.754	-0.041	0.01	-0.012	0.021
**rs13287217**	A	G	0.978	0.119	0.029	0.053	0.058
**rs1361500**	T	C	0.637	-0.038	0.008	0.003	0.018
**rs139161896**	A	G	0.032	0.099	0.022	-0.04	0.049
**rs139627276**	T	C	0.009	0.309	0.067	-0.031	0.114
**rs141053258**	A	G	0.02	0.151	0.033	-0.029	0.068
**rs141487324**	A	C	0.05	0.096	0.022	0.131	0.053

**rs141811146**	A	G	0.988	0.202	0.049	0.078	0.089
**rs141858113**	A	T	0.01	0.234	0.052	0.05	0.146
**rs142205197**	A	G	0.014	0.206	0.05	-0.016	0.086
**rs144895331**	T	C	0.906	-0.08	0.016	0.012	0.034
**rs144926805**	T	C	0.991	0.254	0.06	0.096	0.108
**rs145403365**	A	G	0.014	0.146	0.035	0.139	0.078
**rs145549507**	A	G	0.972	-0.121	0.03	-0.086	0.056
**rs146299656**	A	G	0.993	-0.281	0.069	0.005	0.14
**rs1463728**	A	T	0.84	-0.045	0.011	0.028	0.023
**rs1504132**	T	C	0.735	0.039	0.009	0.001	0.021
**rs150658015**	T	C	0.974	0.101	0.024	0.012	0.056
**rs150888111**	A	C	0.011	-0.225	0.05	0.074	0.099
**rs1525970**	A	G	0.236	-0.039	0.01	0.007	0.02
**rs164470**	T	C	0.674	0.038	0.009	0.039	0.018
**rs167728**	A	G	0.629	-0.034	0.008	0.014	0.017
**rs17232271**	A	G	0.05	0.089	0.02	-0.01	0.044
**rs17499892**	A	C	0.623	-0.038	0.008	-0.012	0.017
**rs17737641**	A	G	0.968	-0.102	0.022	-0.038	0.048
**rs184986187**	A	C	0.013	0.174	0.042	0.02	0.08
**rs1859199**	C	G	0.771	-0.04	0.01	0.007	0.021
**rs1899048**	T	C	0.192	0.046	0.01	0.034	0.022
**rs1950829**	A	G	0.496	0.045	0.008	-0.001	0.017
**rs1968295**	T	C	0.371	0.043	0.01	0.002	0.02
**rs2001315**	A	G	0.391	-0.036	0.008	-0.032	0.017
**rs2056476**	T	C	0.483	-0.04	0.009	-0.005	0.019
**rs2084080**	A	G	0.164	-0.048	0.011	0.016	0.023
**rs2085224**	A	G	0.888	-0.053	0.012	0.011	0.03
**rs2193534**	A	G	0.442	0.033	0.008	0.002	0.017
**rs2378804**	T	C	0.171	-0.048	0.011	-0.008	0.023
**rs2451828**	T	C	0.026	0.144	0.027	-0.024	0.059
**rs2509805**	T	C	0.343	0.043	0.009	-0.046	0.019
**rs2589912**	A	G	0.334	0.037	0.008	0.01	0.018
**rs2678273**	T	C	0.84	0.049	0.011	0.026	0.024
**rs2756119**	A	G	0.382	-0.043	0.009	0.03	0.018
**rs2804863**	T	C	0.19	-0.043	0.01	-0.004	0.023
**rs2824418**	C	G	0.774	0.041	0.01	-0.025	0.02
**rs28803900**	A	C	0.52	0.036	0.008	0.015	0.017
**rs3008841**	T	G	0.557	0.036	0.008	0.022	0.017
**rs302274**	T	C	0.923	-0.07	0.017	0.027	0.032
**rs34034295**	C	G	0.981	-0.127	0.03	-0.021	0.079
**rs34675181**	A	G	0.533	0.036	0.008	0.002	0.017
**rs372519**	A	G	0.524	0.038	0.008	0.015	0.017
**rs4133214**	A	G	0.712	-0.036	0.009	-0.024	0.019
**rs4261305**	A	G	0.183	0.045	0.01	0.015	0.022
**rs4667368**	A	G	0.024	0.144	0.035	0.051	0.068

**rs4735241**	A	G	0.509	0.039	0.008	0.027	0.018
**rs4745763**	A	C	0.73	-0.037	0.009	0.004	0.019
**rs4761839**	A	G	0.208	0.043	0.01	0.004	0.021
**rs4776634**	A	G	0.05	0.086	0.021	0.002	0.048
**rs4776768**	T	C	0.278	-0.042	0.009	-0.029	0.019
**rs4902962**	A	G	0.092	0.059	0.014	-0.005	0.029
**rs4903267**	T	G	0.531	-0.034	0.008	-0.011	0.017
**rs5011520**	A	G	0.087	-0.063	0.014	-0.031	0.03
**rs55643602**	A	T	0.812	0.044	0.01	-0.001	0.022
**rs55730016**	T	C	0.354	0.039	0.009	-0.006	0.019
**rs55796107**	T	C	0.479	-0.034	0.008	0.031	0.017
**rs56016904**	A	G	0.794	-0.051	0.01	-0.01	0.023
**rs568941172**	T	A	0.023	0.272	0.065	0.018	0.056
**rs569606209**	A	G	0.018	0.28	0.068	-0.086	0.071
**rs60747160**	T	G	0.374	0.04	0.008	-0.011	0.018
**rs60809787**	T	C	0.98	-0.121	0.029	0.062	0.065
**rs61356283**	T	C	0.017	0.154	0.033	0.119	0.07
**rs61945218**	T	C	0.864	-0.051	0.012	0.015	0.025
**rs62074125**	A	C	0.751	0.041	0.01	-0.021	0.023
**rs6511757**	A	G	0.982	-0.205	0.047	0.007	0.071
**rs6532547**	T	C	0.693	-0.039	0.009	-0.005	0.018
**rs6717413**	A	G	0.637	-0.041	0.008	-0.016	0.018
**rs6806340**	T	C	0.204	-0.043	0.01	0.034	0.021
**rs6832890**	C	G	0.202	-0.061	0.011	0.014	0.021
**rs6905391**	A	G	0.142	-0.074	0.011	-0.01	0.024
**rs6939697**	A	G	0.779	-0.046	0.011	-0.008	0.02
**rs6964065**	T	G	0.918	-0.067	0.016	-0.061	0.033
**rs7035041**	T	C	0.33	-0.038	0.009	-0.001	0.019
**rs7117176**	T	C	0.108	0.057	0.014	-0.023	0.029
**rs7127852**	A	T	0.818	-0.044	0.011	-0.001	0.024
**rs7136545**	T	C	0.022	0.112	0.028	0.07	0.057
**rs7144406**	A	G	0.8	-0.04	0.01	0.049	0.021
**rs71637418**	T	C	0.158	-0.047	0.011	-0.019	0.023
**rs726857**	A	T	0.369	-0.037	0.008	0.014	0.017
**rs73091592**	A	T	0.111	-0.053	0.013	0.065	0.027
**rs73129119**	A	G	0.041	-0.086	0.02	0.011	0.042
**rs73173739**	T	C	0.218	0.039	0.01	0.016	0.02
**rs74488857**	T	C	0.033	-0.109	0.026	0.025	0.058
**rs75244260**	T	G	0.938	-0.074	0.018	-0.048	0.038
**rs7531118**	T	C	0.476	-0.045	0.008	0.028	0.018
**rs76025409**	C	G	0.36	0.057	0.009	0.006	0.018
**rs7603111**	A	G	0.685	-0.041	0.009	0.01	0.018
**rs76414965**	A	T	0.031	0.103	0.023	-0.026	0.049
**rs77098061**	T	C	0.879	-0.058	0.012	0	0.026
**rs77532933**	T	C	0.088	-0.068	0.014	-0.009	0.034

**rs77731636**	A	G	0.038	0.093	0.023	0.062	0.047
**rs7784031**	A	G	0.354	-0.039	0.008	-0.009	0.017
**rs77903372**	T	C	0.01	0.226	0.053	-0.105	0.089
**rs78338135**	A	C	0.978	-0.132	0.032	0.119	0.083
**rs7856424**	T	C	0.287	-0.045	0.009	-0.01	0.019
**rs78955704**	A	T	0.031	0.112	0.021	0.003	0.057
**rs79010246**	A	G	0.966	-0.101	0.025	-0.039	0.055
**rs8012923**	A	G	0.407	-0.04	0.008	-0.03	0.018
**rs8029553**	A	G	0.307	0.044	0.011	0.017	0.021
**rs80325622**	A	G	0.027	-0.119	0.025	0.063	0.052
**rs8106047**	A	T	0.151	-0.061	0.014	0.081	0.027
**rs8138223**	T	C	0.405	-0.039	0.009	-0.055	0.018
**rs9292849**	A	G	0.473	0.035	0.008	0.009	0.017
**rs9367487**	A	C	0.285	-0.036	0.009	-0.031	0.019
**rs9926691**	A	G	0.316	-0.036	0.009	-0.02	0.018

Table S49. Raw data for the MR analysis of the causal effect of MDD on CD (P<5E-5)							
SNP	Effect allele	Other allele	Effect allele frequency	Beta.exposure	Se.exposure	Beta.outcome	Se.outcome
rs10235395	A	G	0.69	-0.037	0.009	0.029	0.025
rs10411666	C	G	0.207	0.053	0.013	-0.08	0.04
rs10759934	A	T	0.497	0.036	0.008	-0.001	0.023
rs10782599	C	G	0.845	0.045	0.011	0.04	0.032
rs10813253	T	C	0.908	-0.061	0.014	0.023	0.045
rs10825942	T	G	0.455	-0.044	0.008	-0.045	0.024
rs10861729	T	C	0.315	-0.039	0.008	-0.025	0.025
rs1086490	A	G	0.891	0.058	0.014	0.031	0.037
rs1095163	A	G	0.452	0.033	0.008	0.006	0.023
rs10987923	A	G	0.327	0.035	0.009	0.004	0.025
rs1106235	A	G	0.202	0.05	0.012	0.012	0.03
rs11077049	A	G	0.022	0.139	0.03	-0.016	0.082
rs11121207	A	C	0.199	0.043	0.01	-0.005	0.029
rs11192230	A	T	0.737	0.041	0.009	0.042	0.026
rs11220476	T	C	0.167	-0.048	0.012	0.012	0.031
rs112379491	A	G	0.822	-0.044	0.011	-0.003	0.03
rs113021645	A	C	0.021	0.123	0.03	-0.03	0.093
rs114923224	T	C	0.061	0.072	0.015	0.013	0.048
rs1152589	A	T	0.474	-0.041	0.009	-0.006	0.023
rs115776708	T	C	0.033	0.121	0.029	-0.085	0.084
rs11590766	T	C	0.567	0.036	0.008	-0.015	0.023
rs116070829	T	C	0.018	0.149	0.035	-0.24	0.101
rs117141160	A	G	0.014	0.249	0.056	-0.031	0.12
rs11725076	A	T	0.759	-0.053	0.012	0.035	0.03
rs11789013	T	C	0.751	-0.042	0.009	-0.066	0.026
rs117901488	T	C	0.043	0.095	0.021	-0.026	0.066
rs11852819	T	C	0.016	0.14	0.033	0.045	0.099
rs11945613	A	G	0.03	0.115	0.027	-0.099	0.071
rs11980163	A	G	0.975	-0.111	0.027	0.033	0.081
rs12129573	A	C	0.382	0.048	0.008	-0.012	0.024
rs1214608	A	T	0.565	0.035	0.008	0.007	0.023
rs12200766	A	G	0.763	0.047	0.009	0.046	0.027
rs12552	A	G	0.436	0.04	0.008	0.005	0.023
rs12666117	A	G	0.477	0.034	0.008	0.035	0.023
rs12700978	T	C	0.542	0.034	0.008	-0.035	0.023
rs13171899	A	G	0.76	-0.041	0.01	0.027	0.029
rs13287217	A	G	0.978	0.119	0.029	0.002	0.079
rs1361500	T	C	0.637	-0.038	0.008	-0.011	0.025
rs139161896	A	G	0.032	0.099	0.022	-0.063	0.067
rs139627276	T	C	0.009	0.309	0.067	-0.033	0.148
rs141053258	A	G	0.02	0.151	0.033	-0.078	0.091
rs141487324	A	C	0.051	0.096	0.022	0.11	0.071

**rs141811146**	A	G	0.988	0.202	0.049	0.031	0.119
**rs141858113**	A	T	0.011	0.234	0.052	0.012	0.201
**rs142205197**	A	G	0.014	0.206	0.05	0.032	0.116
**rs144895331**	T	C	0.905	-0.08	0.016	0.008	0.045
**rs144926805**	T	C	0.992	0.254	0.06	0.197	0.149
**rs145403365**	A	G	0.013	0.146	0.035	0.076	0.115
**rs145549507**	A	G	0.972	-0.121	0.03	-0.061	0.076
**rs146299656**	A	G	0.995	-0.281	0.069	-0.078	0.205
**rs1463728**	A	T	0.84	-0.045	0.011	-0.008	0.031
**rs1504132**	T	C	0.738	0.039	0.009	0	0.028
**rs150658015**	T	C	0.976	0.101	0.024	0	0.085
**rs150888111**	A	C	0.01	-0.225	0.05	0.228	0.144
**rs1525970**	A	G	0.239	-0.039	0.01	-0.002	0.028
**rs164470**	T	C	0.671	0.038	0.009	0.041	0.026
**rs167728**	A	G	0.63	-0.034	0.008	-0.001	0.024
**rs17232271**	A	G	0.049	0.089	0.02	0.035	0.062
**rs17499892**	A	C	0.624	-0.038	0.008	-0.013	0.024
**rs17737641**	A	G	0.969	-0.102	0.022	-0.033	0.066
**rs184986187**	A	C	0.013	0.174	0.042	0.075	0.107
**rs1859199**	C	G	0.772	-0.04	0.01	0.041	0.029
**rs1899048**	T	C	0.192	0.046	0.01	0.043	0.03
**rs1950829**	A	G	0.497	0.045	0.008	-0.006	0.023
**rs1968295**	T	C	0.367	0.043	0.01	0.029	0.027
**rs2001315**	A	G	0.389	-0.036	0.008	-0.024	0.023
**rs2056476**	T	C	0.488	-0.04	0.009	-0.032	0.025
**rs2084080**	A	G	0.165	-0.048	0.011	-0.026	0.031
**rs2085224**	A	G	0.893	-0.053	0.012	0.034	0.042
**rs2193534**	A	G	0.437	0.033	0.008	-0.026	0.023
**rs2378804**	T	C	0.172	-0.048	0.011	0.001	0.031
**rs2451828**	T	C	0.026	0.144	0.027	-0.008	0.081
**rs2509805**	T	C	0.34	0.043	0.009	-0.051	0.025
**rs2589912**	A	G	0.336	0.037	0.008	0.01	0.024
**rs2678273**	T	C	0.837	0.049	0.011	0.039	0.033
**rs2756119**	A	G	0.381	-0.043	0.009	0.056	0.024
**rs2804863**	T	C	0.189	-0.043	0.01	0.026	0.03
**rs2824418**	C	G	0.77	0.041	0.01	-0.007	0.027
**rs28803900**	A	C	0.515	0.036	0.008	0.02	0.023
**rs3008841**	T	G	0.559	0.036	0.008	0.011	0.023
**rs302274**	T	C	0.926	-0.07	0.017	-0.015	0.044
**rs34034295**	C	G	0.981	-0.127	0.03	0.057	0.109
**rs34675181**	A	G	0.538	0.036	0.008	0.023	0.024
**rs372519**	A	G	0.525	0.038	0.008	0.021	0.023
**rs4133214**	A	G	0.718	-0.036	0.009	-0.047	0.026
**rs4261305**	A	G	0.178	0.045	0.01	0.012	0.03
**rs4667368**	A	G	0.025	0.144	0.035	-0.019	0.09

**rs4735241**	A	G	0.506	0.039	0.008	0.028	0.024
**rs4745763**	A	C	0.729	-0.037	0.009	0.008	0.025
**rs4761839**	A	G	0.209	0.043	0.01	-0.007	0.028
**rs4776634**	A	G	0.052	0.086	0.021	0.013	0.066
**rs4776768**	T	C	0.277	-0.042	0.009	-0.028	0.026
**rs4902962**	A	G	0.093	0.059	0.014	-0.043	0.04
**rs4903267**	T	G	0.529	-0.034	0.008	-0.011	0.023
**rs5011520**	A	G	0.087	-0.063	0.014	-0.005	0.041
**rs55643602**	A	T	0.813	0.044	0.01	-0.015	0.029
**rs55730016**	T	C	0.353	0.039	0.009	-0.013	0.026
**rs55796107**	T	C	0.477	-0.034	0.008	0.041	0.023
**rs56016904**	A	G	0.797	-0.051	0.01	-0.014	0.031
**rs568941172**	T	A	0.023	0.272	0.065	0	0.079
**rs569606209**	A	G	0.019	0.28	0.068	-0.072	0.096
**rs60747160**	T	G	0.382	0.04	0.008	0.036	0.024
**rs60809787**	T	C	0.98	-0.121	0.029	-0.017	0.088
**rs61356283**	T	C	0.016	0.154	0.033	0.08	0.097
**rs61945218**	T	C	0.862	-0.051	0.012	-0.011	0.033
**rs62074125**	A	C	0.751	0.041	0.01	-0.011	0.032
**rs6511757**	A	G	0.979	-0.205	0.047	0.022	0.089
**rs6532547**	T	C	0.698	-0.039	0.009	0.007	0.025
**rs6717413**	A	G	0.638	-0.041	0.008	-0.022	0.025
**rs6806340**	T	C	0.202	-0.043	0.01	0.051	0.028
**rs6832890**	C	G	0.201	-0.061	0.011	0.035	0.029
**rs6905391**	A	G	0.141	-0.074	0.011	-0.036	0.033
**rs6939697**	A	G	0.779	-0.046	0.011	0.008	0.028
**rs6964065**	T	G	0.918	-0.067	0.016	-0.097	0.046
**rs7035041**	T	C	0.326	-0.038	0.009	-0.022	0.026
**rs7117176**	T	C	0.108	0.057	0.014	-0.033	0.039
**rs7127852**	A	T	0.813	-0.044	0.011	0.012	0.032
**rs7136545**	T	C	0.023	0.112	0.028	0.016	0.076
**rs7144406**	A	G	0.801	-0.04	0.01	0.066	0.029
**rs71637418**	T	C	0.162	-0.047	0.011	-0.004	0.031
**rs726857**	A	T	0.362	-0.037	0.008	-0.025	0.024
**rs73091592**	A	T	0.107	-0.053	0.013	0.074	0.037
**rs73129119**	A	G	0.042	-0.086	0.02	-0.013	0.058
**rs73173739**	T	C	0.219	0.039	0.01	0.041	0.028
**rs74488857**	T	C	0.033	-0.109	0.026	0.104	0.078
**rs75244260**	T	G	0.936	-0.074	0.018	-0.034	0.052
**rs7531118**	T	C	0.472	-0.045	0.008	0.058	0.024
**rs76025409**	C	G	0.358	0.057	0.009	-0.031	0.024
**rs7603111**	A	G	0.686	-0.041	0.009	-0.016	0.025
**rs76414965**	A	T	0.032	0.103	0.023	0.069	0.064
**rs77098061**	T	C	0.881	-0.058	0.012	0.027	0.036
**rs77532933**	T	C	0.084	-0.068	0.014	-0.072	0.049

**rs77731636**	A	G	0.039	0.093	0.023	0.054	0.062
**rs7784031**	A	G	0.352	-0.039	0.008	-0.004	0.024
**rs77903372**	T	C	0.008	0.226	0.053	-0.111	0.133
**rs78338135**	A	C	0.977	-0.132	0.032	0.066	0.111
**rs7856424**	T	C	0.288	-0.045	0.009	-0.014	0.025
**rs78955704**	A	T	0.029	0.112	0.021	0.06	0.081
**rs79010246**	A	G	0.966	-0.101	0.025	0.007	0.075
**rs8012923**	A	G	0.415	-0.04	0.008	-0.008	0.024
**rs8029553**	A	G	0.304	0.044	0.011	-0.014	0.029
**rs80325622**	A	G	0.027	-0.119	0.025	0.039	0.071
**rs8106047**	A	T	0.15	-0.061	0.014	0.097	0.037
**rs8138223**	T	C	0.405	-0.039	0.009	-0.054	0.025
**rs9292849**	A	G	0.468	0.035	0.008	-0.022	0.024
**rs9367487**	A	C	0.285	-0.036	0.009	-0.025	0.026
**rs9926691**	A	G	0.318	-0.036	0.009	0.038	0.025

Table S50. Raw data for the MR analysis of the causal effect of MDD on UC (P<5E-5)							
**SNP**	**Effect allele**	**Other allele**	**Effect allele frequency**	**Beta.exposure**	**Se.exposure**	**Beta.outcome**	**Se.outcome**
**rs10235395**	A	G	0.689	-0.037	0.009	-0.035	0.023
**rs10411666**	C	G	0.216	0.053	0.013	0.005	0.036
**rs10759934**	A	T	0.496	0.036	0.008	-0.028	0.021
**rs10782599**	C	G	0.844	0.045	0.011	0.029	0.03
**rs10813253**	T	C	0.909	-0.061	0.014	0.053	0.041
**rs10825942**	T	G	0.457	-0.044	0.008	0.02	0.022
**rs10861729**	T	C	0.318	-0.039	0.008	0.019	0.023
**rs1086490**	A	G	0.886	0.058	0.014	-0.048	0.034
**rs1095163**	A	G	0.454	0.033	0.008	-0.03	0.022
**rs10987923**	A	G	0.322	0.035	0.009	-0.02	0.023
**rs1106235**	A	G	0.196	0.05	0.012	0.003	0.027
**rs11077049**	A	G	0.021	0.139	0.03	0.086	0.074
**rs11121207**	A	C	0.195	0.043	0.01	0.043	0.027
**rs11192230**	A	T	0.739	0.041	0.009	-0.02	0.024
**rs11220476**	T	C	0.161	-0.048	0.012	0.025	0.03
**rs112379491**	A	G	0.822	-0.044	0.011	-0.016	0.028
**rs113021645**	A	C	0.021	0.123	0.03	-0.044	0.087
**rs114923224**	T	C	0.063	0.072	0.015	0.036	0.044
**rs1152589**	A	T	0.481	-0.041	0.009	0.02	0.021
**rs115776708**	T	C	0.033	0.121	0.029	-0.122	0.077
**rs11590766**	T	C	0.568	0.036	0.008	-0.005	0.022
**rs116070829**	T	C	0.018	0.149	0.035	0.086	0.09
**rs117141160**	A	G	0.014	0.249	0.056	-0.125	0.122
**rs11725076**	A	T	0.763	-0.053	0.012	0.004	0.028
**rs11789013**	T	C	0.757	-0.042	0.009	-0.026	0.025
**rs117901488**	T	C	0.043	0.095	0.021	0.051	0.06
**rs11852819**	T	C	0.016	0.14	0.033	0.088	0.09
**rs11945613**	A	G	0.03	0.115	0.027	-0.013	0.063
**rs11980163**	A	G	0.975	-0.111	0.027	-0.076	0.073
**rs12129573**	A	C	0.374	0.048	0.008	-0.019	0.022
**rs1214608**	A	T	0.56	0.035	0.008	-0.019	0.022
**rs12200766**	A	G	0.755	0.047	0.009	0.012	0.025
**rs12552**	A	G	0.434	0.04	0.008	0.021	0.021
**rs12666117**	A	G	0.472	0.034	0.008	0.01	0.021
**rs12700978**	T	C	0.548	0.034	0.008	-0.016	0.021
**rs13171899**	A	G	0.75	-0.041	0.01	-0.038	0.025
**rs13287217**	A	G	0.978	0.119	0.029	0.074	0.074
**rs1361500**	T	C	0.637	-0.038	0.008	0.012	0.022
**rs139161896**	A	G	0.033	0.099	0.022	-0.05	0.063
**rs139627276**	T	C	0.009	0.309	0.067	0.066	0.143
**rs141053258**	A	G	0.021	0.151	0.033	0.044	0.086
**rs141487324**	A	C	0.049	0.096	0.022	0.179	0.066

**rs141811146**	A	G	0.988	0.202	0.049	0.081	0.114
**rs141858113**	A	T	0.01	0.234	0.052	0.201	0.182
**rs142205197**	A	G	0.014	0.206	0.05	0.033	0.11
**rs144895331**	T	C	0.907	-0.08	0.016	0.005	0.042
**rs144926805**	T	C	0.991	0.254	0.06	0.08	0.134
**rs145403365**	A	G	0.014	0.146	0.035	0.161	0.094
**rs145549507**	A	G	0.972	-0.121	0.03	-0.1	0.07
**rs146299656**	A	G	0.993	-0.281	0.069	0.1	0.174
**rs1463728**	A	T	0.841	-0.045	0.011	0.063	0.029
**rs1504132**	T	C	0.733	0.039	0.009	-0.017	0.026
**rs150658015**	T	C	0.973	0.101	0.024	-0.01	0.067
**rs150888111**	A	C	0.011	-0.225	0.05	0.002	0.12
**rs1525970**	A	G	0.234	-0.039	0.01	0.014	0.025
**rs164470**	T	C	0.674	0.038	0.009	0.038	0.023
**rs167728**	A	G	0.628	-0.034	0.008	0.029	0.022
**rs17232271**	A	G	0.051	0.089	0.02	-0.051	0.054
**rs17499892**	A	C	0.622	-0.038	0.008	-0.019	0.022
**rs17737641**	A	G	0.967	-0.102	0.022	-0.029	0.06
**rs184986187**	A	C	0.012	0.174	0.042	0.005	0.102
**rs1859199**	C	G	0.77	-0.04	0.01	-0.015	0.026
**rs1899048**	T	C	0.19	0.046	0.01	0.046	0.028
**rs1950829**	A	G	0.496	0.045	0.008	-0.008	0.021
**rs1968295**	T	C	0.372	0.043	0.01	-0.003	0.025
**rs2001315**	A	G	0.393	-0.036	0.008	-0.042	0.022
**rs2056476**	T	C	0.482	-0.04	0.009	0.012	0.023
**rs2084080**	A	G	0.164	-0.048	0.011	0.032	0.029
**rs2085224**	A	G	0.884	-0.053	0.012	0.003	0.036
**rs2193534**	A	G	0.445	0.033	0.008	0.024	0.022
**rs2378804**	T	C	0.172	-0.048	0.011	-0.012	0.028
**rs2451828**	T	C	0.026	0.144	0.027	-0.055	0.074
**rs2509805**	T	C	0.345	0.043	0.009	-0.037	0.023
**rs2589912**	A	G	0.334	0.037	0.008	0.005	0.022
**rs2678273**	T	C	0.838	0.049	0.011	0.011	0.03
**rs2756119**	A	G	0.381	-0.043	0.009	0.02	0.022
**rs2804863**	T	C	0.189	-0.043	0.01	-0.038	0.029
**rs2824418**	C	G	0.775	0.041	0.01	-0.045	0.026
**rs28803900**	A	C	0.521	0.036	0.008	0.013	0.021
**rs3008841**	T	G	0.557	0.036	0.008	0.009	0.021
**rs302274**	T	C	0.924	-0.07	0.017	0.038	0.04
**rs34034295**	C	G	0.981	-0.127	0.03	-0.047	0.098
**rs34675181**	A	G	0.53	0.036	0.008	-0.01	0.022
**rs372519**	A	G	0.522	0.038	0.008	-0.001	0.022
**rs4133214**	A	G	0.711	-0.036	0.009	-0.013	0.024
**rs4261305**	A	G	0.184	0.045	0.01	0.022	0.027
**rs4667368**	A	G	0.024	0.144	0.035	0.003	0.086

**rs4735241**	A	G	0.508	0.039	0.008	0.034	0.022
**rs4745763**	A	C	0.73	-0.037	0.009	0	0.024
**rs4761839**	A	G	0.209	0.043	0.01	0.018	0.027
**rs4776634**	A	G	0.05	0.086	0.021	0	0.062
**rs4776768**	T	C	0.279	-0.042	0.009	-0.029	0.024
**rs4902962**	A	G	0.091	0.059	0.014	0.016	0.037
**rs4903267**	T	G	0.531	-0.034	0.008	-0.024	0.021
**rs5011520**	A	G	0.088	-0.063	0.014	-0.043	0.038
**rs55643602**	A	T	0.814	0.044	0.01	-0.013	0.027
**rs55730016**	T	C	0.355	0.039	0.009	0.003	0.024
**rs55796107**	T	C	0.478	-0.034	0.008	0.024	0.021
**rs56016904**	A	G	0.793	-0.051	0.01	0.007	0.029
**rs568941172**	T	A	0.024	0.272	0.065	0.058	0.07
**rs569606209**	A	G	0.018	0.28	0.068	-0.05	0.091
**rs60747160**	T	G	0.37	0.04	0.008	-0.05	0.022
**rs60809787**	T	C	0.98	-0.121	0.029	0.116	0.081
**rs61356283**	T	C	0.017	0.154	0.033	0.132	0.087
**rs61945218**	T	C	0.865	-0.051	0.012	0.037	0.031
**rs62074125**	A	C	0.749	0.041	0.01	-0.027	0.03
**rs6511757**	A	G	0.983	-0.205	0.047	-0.006	0.093
**rs6532547**	T	C	0.691	-0.039	0.009	-0.016	0.023
**rs6717413**	A	G	0.638	-0.041	0.008	-0.026	0.023
**rs6806340**	T	C	0.203	-0.043	0.01	0.031	0.026
**rs6832890**	C	G	0.201	-0.061	0.011	0.01	0.027
**rs6905391**	A	G	0.143	-0.074	0.011	0.004	0.03
**rs6939697**	A	G	0.778	-0.046	0.011	-0.021	0.026
**rs6964065**	T	G	0.918	-0.067	0.016	-0.052	0.042
**rs7035041**	T	C	0.33	-0.038	0.009	0.002	0.023
**rs7117176**	T	C	0.107	0.057	0.014	-0.003	0.036
**rs7127852**	A	T	0.818	-0.044	0.011	-0.019	0.031
**rs7136545**	T	C	0.021	0.112	0.028	0.053	0.073
**rs7144406**	A	G	0.799	-0.04	0.01	0.041	0.027
**rs71637418**	T	C	0.156	-0.047	0.011	-0.031	0.029
**rs726857**	A	T	0.373	-0.037	0.008	0.043	0.022
**rs73091592**	A	T	0.111	-0.053	0.013	0.061	0.033
**rs73129119**	A	G	0.042	-0.086	0.02	0.007	0.053
**rs73173739**	T	C	0.216	0.039	0.01	0.007	0.026
**rs74488857**	T	C	0.033	-0.109	0.026	-0.059	0.074
**rs75244260**	T	G	0.939	-0.074	0.018	-0.035	0.048
**rs7531118**	T	C	0.473	-0.045	0.008	0	0.022
**rs76025409**	C	G	0.363	0.057	0.009	0.029	0.022
**rs7603111**	A	G	0.685	-0.041	0.009	0.024	0.023
**rs76414965**	A	T	0.031	0.103	0.023	-0.111	0.062
**rs77098061**	T	C	0.878	-0.058	0.012	-0.026	0.032
**rs77532933**	T	C	0.089	-0.068	0.014	0.042	0.042

**rs77731636**	A	G	0.038	0.093	0.023	0.032	0.059
**rs7784031**	A	G	0.354	-0.039	0.008	-0.008	0.022
**rs77903372**	T	C	0.01	0.226	0.053	-0.103	0.107
**rs78338135**	A	C	0.979	-0.132	0.032	0.107	0.109
**rs7856424**	T	C	0.287	-0.045	0.009	-0.01	0.023
**rs78955704**	A	T	0.033	0.112	0.021	-0.061	0.07
**rs79010246**	A	G	0.966	-0.101	0.025	-0.059	0.068
**rs8012923**	A	G	0.404	-0.04	0.008	-0.052	0.022
**rs8029553**	A	G	0.307	0.044	0.011	0.038	0.027
**rs80325622**	A	G	0.027	-0.119	0.025	0.049	0.065
**rs8106047**	A	T	0.151	-0.061	0.014	0.046	0.034
**rs8138223**	T	C	0.407	-0.039	0.009	-0.062	0.023
**rs9292849**	A	G	0.475	0.035	0.008	0.021	0.022
**rs9367487**	A	C	0.285	-0.036	0.009	-0.044	0.024
**rs9926691**	A	G	0.315	-0.036	0.009	-0.054	0.023

Table S51. Raw data for the MR analysis of the causal effect of OCD on IBD (P<5E-5)							
SNP	Effect allele	Other allele	Effect allele frequency	Beta.exposure	Se.exposure	Beta.outcome	Se.outcome
**rs10185006**	A	G	0.438	0.141	0.034	-0.006	0.017
**rs1030757**	A	C	0.515	-0.165	0.034	-0.023	0.017
**rs10474856**	A	T	0.514	0.148	0.034	0.026	0.017
**rs10479583**	C	G	0.581	-0.144	0.034	0.007	0.017
**rs10773765**	T	C	0.255	0.184	0.04	-0.022	0.02
**rs111926263**	A	G	0.203	0.187	0.043	0.01	0.023
**rs112883781**	T	C	0.105	-0.242	0.059	-0.011	0.028
**rs116045211**	T	C	0.019	0.804	0.186	-0.012	0.078
**rs116347760**	A	T	0.02	0.63	0.134	0.089	0.068
**rs117310268**	T	C	0.035	0.449	0.097	0.084	0.051
**rs117502683**	T	C	0.014	0.719	0.167	0.123	0.082
**rs118060392**	A	G	0.974	-0.448	0.106	-0.024	0.054
**rs12033048**	T	C	0.845	0.218	0.05	0.015	0.024
**rs12055962**	A	G	0.68	0.152	0.036	0.015	0.018
**rs12095546**	A	G	0.247	0.175	0.043	-0.024	0.022
**rs12195828**	A	G	0.359	0.145	0.035	0.012	0.018
**rs12504244**	C	G	0.604	-0.169	0.035	0.014	0.018
**rs12568997**	A	G	0.301	-0.293	0.058	-0.024	0.02
**rs12596951**	T	C	0.417	0.156	0.036	0.012	0.018
**rs12638795**	A	G	0.086	0.237	0.058	-0.018	0.031
**rs12804088**	T	C	0.985	-0.602	0.148	0.006	0.08
**rs13107289**	A	C	0.616	-0.145	0.035	-0.017	0.018
**rs13127241**	A	G	0.154	0.187	0.046	0.021	0.023
**rs13141765**	T	C	0.6	-0.266	0.056	0.002	0.02
**rs13256859**	T	C	0.145	0.21	0.05	-0.048	0.026
**rs138445568**	A	T	0.988	-0.928	0.207	0.032	0.104
**rs139286049**	A	G	0.982	-0.601	0.135	-0.143	0.075
**rs139617**	A	C	0.381	0.155	0.036	0.018	0.018
**rs141664172**	A	G	0.022	0.488	0.113	-0.001	0.059
**rs1652783**	A	G	0.781	-0.269	0.059	-0.021	0.024
**rs17748070**	T	C	0.02	0.513	0.126	-0.095	0.064
**rs17756387**	T	C	0.965	-0.396	0.095	-0.105	0.048
**rs17833131**	A	G	0.115	0.214	0.053	0.034	0.026
**rs1918508**	A	G	0.547	0.151	0.036	0.009	0.018
**rs203665**	T	C	0.116	0.252	0.06	-0.018	0.03
**rs2040325**	T	C	0.922	-0.268	0.062	-0.004	0.031
**rs2200119**	A	T	0.733	0.262	0.062	-0.01	0.023
**rs2253477**	A	T	0.248	-0.173	0.04	0.036	0.019
**rs2469785**	C	G	0.721	-0.161	0.04	-0.003	0.021
**rs2545802**	A	G	0.08	-0.433	0.102	-0.064	0.04
**rs28599745**	A	G	0.156	-0.363	0.08	0.019	0.03
**rs28827783**	T	C	0.13	0.23	0.054	-0.025	0.028

**rs2927708**	T	C	0.67	-0.148	0.036	-0.032	0.019
**rs2940229**	C	G	0.723	-0.202	0.046	0.008	0.02
**rs3097331**	T	C	0.629	0.157	0.035	-0.013	0.018
**rs35306**	A	G	0.539	-0.143	0.034	-0.007	0.017
**rs35727592**	T	C	0.029	0.469	0.109	-0.023	0.06
**rs35894340**	A	G	0.75	-0.175	0.039	-0.011	0.02
**rs369128**	A	T	0.715	-0.155	0.037	-0.01	0.019
**rs3785423**	T	C	0.034	-0.471	0.107	-0.103	0.051
**rs3787457**	T	C	0.244	0.164	0.039	0	0.02
**rs3935665**	A	G	0.676	0.152	0.036	-0.023	0.018
**rs4074650**	A	G	0.357	0.151	0.036	0.011	0.018
**rs4449480**	T	C	0.648	0.158	0.036	-0.018	0.018
**rs4733767**	A	G	0.274	0.194	0.039	0.013	0.02
**rs4734125**	A	G	0.123	-0.221	0.054	-0.039	0.026
**rs4859478**	A	G	0.226	0.163	0.04	-0.03	0.02
**rs487974**	A	G	0.345	0.175	0.041	0.008	0.021
**rs4903664**	T	C	0.47	0.144	0.033	0.019	0.017
**rs4969099**	A	G	0.67	0.152	0.037	-0.026	0.018
**rs4978387**	T	C	0.81	0.304	0.074	-0.033	0.026
**rs55687617**	A	G	0.117	-0.27	0.058	0.039	0.028
**rs56025909**	T	C	0.03	0.412	0.092	-0.038	0.051
**rs56343802**	A	T	0.716	-0.17	0.037	-0.009	0.018
**rs57514340**	T	C	0.77	0.176	0.042	-0.024	0.022
**rs5757717**	T	C	0.467	-0.146	0.035	0.031	0.017
**rs58776066**	A	C	0.131	0.204	0.05	0.024	0.028
**rs6111584**	T	C	0.973	-0.482	0.11	-0.084	0.056
**rs61146272**	T	C	0.921	0.294	0.067	0.02	0.032
**rs61742168**	A	G	0.987	-0.662	0.161	-0.198	0.085
**rs61933097**	A	G	0.949	-0.314	0.074	0.008	0.042
**rs62249119**	T	C	0.031	-0.484	0.117	-0.047	0.052
**rs639560**	T	C	0.047	-0.416	0.088	0.009	0.04
**rs6580184**	A	G	0.536	0.147	0.034	-0.022	0.017
**rs6871469**	A	C	0.243	-0.169	0.04	0.023	0.02
**rs6984782**	T	C	0.858	-0.208	0.048	-0.002	0.025
**rs6997554**	T	C	0.64	0.151	0.035	-0.013	0.018
**rs7124427**	A	G	0.234	0.171	0.039	0.015	0.02
**rs7206058**	T	G	0.821	0.214	0.052	-0.046	0.025
**rs72781967**	T	C	0.657	-0.167	0.035	0.024	0.018
**rs72783425**	A	C	0.055	0.34	0.073	0.021	0.04
**rs73120742**	A	G	0.022	0.486	0.119	0.083	0.066
**rs73404203**	T	C	0.035	0.393	0.097	-0.047	0.048
**rs74156199**	T	C	0.014	0.534	0.125	0.037	0.073
**rs74750933**	A	G	0.218	-0.168	0.041	0.011	0.021
**rs74993536**	C	G	0.016	0.821	0.201	-0.107	0.082
**rs75740353**	A	G	0.096	-0.433	0.098	-0.069	0.043

**rs7658261**	C	G	0.421	0.151	0.036	0.004	0.018
**rs77885126**	T	C	0.985	-0.603	0.131	0.026	0.072
**rs7790821**	A	G	0.245	0.155	0.038	0.014	0.02
**rs78784799**	A	G	0.013	0.684	0.158	-0.081	0.089
**rs78807573**	T	G	0.124	-0.41	0.1	-0.023	0.034
**rs79306469**	A	G	0.073	0.292	0.069	0.005	0.036
**rs7937505**	A	C	0.258	0.163	0.039	-0.014	0.019
**rs8030746**	A	C	0.722	-0.151	0.037	0.002	0.02
**rs80344830**	A	G	0.936	-0.3	0.068	-0.067	0.035
**rs909701**	C	G	0.506	-0.155	0.034	-0.01	0.017
**rs9399861**	A	G	0.445	0.144	0.035	0.014	0.017
**rs9544927**	A	G	0.793	0.193	0.043	-0.025	0.021
**rs9733630**	A	G	0.269	0.165	0.039	-0.023	0.02
**rs9836705**	A	G	0.469	0.187	0.043	0.001	0.018
**rs9904844**	T	C	0.124	0.205	0.049	-0.006	0.026
**rs9952159**	T	C	0.22	0.182	0.04	0.006	0.02

Table S52 Raw data for the MR analysis of the causal effect of OCD on CD (P<5E-5)							
**SNP**	**Effect allele**	**Other allele**	**Effect allele frequency**	**Beta.exposure**	**Se.exposure**	**Beta.outcome**	**Se.outcome**
**rs10185006**	A	G	0.439	0.141	0.034	0.004	0.023
**rs1030757**	A	C	0.522	-0.165	0.034	-0.03	0.023
**rs10474856**	A	T	0.513	0.148	0.034	0.016	0.023
**rs10479583**	C	G	0.579	-0.144	0.034	0.019	0.023
**rs10773765**	T	C	0.255	0.184	0.04	0.004	0.027
**rs111926263**	A	G	0.2	0.187	0.043	0.047	0.032
**rs112883781**	T	C	0.106	-0.242	0.059	-0.02	0.038
**rs116045211**	T	C	0.019	0.804	0.186	-0.071	0.111
**rs116347760**	A	T	0.019	0.63	0.134	0.203	0.091
**rs117310268**	T	C	0.035	0.449	0.097	0.102	0.07
**rs117502683**	T	C	0.014	0.719	0.167	0.064	0.116
**rs118060392**	A	G	0.974	-0.448	0.106	-0.087	0.074
**rs12033048**	T	C	0.844	0.218	0.05	-0.024	0.032
**rs12055962**	A	G	0.673	0.152	0.036	0.057	0.024
**rs12095546**	A	G	0.247	0.175	0.043	-0.002	0.029
**rs12195828**	A	G	0.358	0.145	0.035	0.008	0.024
**rs12504244**	C	G	0.604	-0.169	0.035	0.054	0.024
**rs12568997**	A	G	0.303	-0.293	0.058	-0.017	0.029
**rs12596951**	T	C	0.409	0.156	0.036	0.008	0.025
**rs12638795**	A	G	0.087	0.237	0.058	-0.016	0.041
**rs12804088**	T	C	0.984	-0.602	0.148	0.085	0.107
**rs13107289**	A	C	0.617	-0.145	0.035	-0.026	0.024
**rs13127241**	A	G	0.152	0.187	0.046	0.019	0.032
**rs13141765**	T	C	0.592	-0.266	0.056	0.02	0.028
**rs13256859**	T	C	0.146	0.21	0.05	-0.051	0.035
**rs138445568**	A	T	0.988	-0.928	0.207	0.07	0.14
**rs139286049**	A	G	0.981	-0.601	0.135	-0.272	0.099
**rs139617**	A	C	0.377	0.155	0.036	0.029	0.025
**rs141664172**	A	G	0.021	0.488	0.113	-0.032	0.082
**rs1652783**	A	G	0.781	-0.269	0.059	-0.038	0.032
**rs17748070**	T	C	0.021	0.513	0.126	-0.097	0.088
**rs17756387**	T	C	0.966	-0.396	0.095	-0.036	0.068
**rs17833131**	A	G	0.11	0.214	0.053	0.007	0.037
**rs1918508**	A	G	0.547	0.151	0.036	-0.044	0.024
**rs203665**	T	C	0.112	0.252	0.06	-0.012	0.044
**rs2040325**	T	C	0.922	-0.268	0.062	-0.041	0.043
**rs2200119**	A	T	0.732	0.262	0.062	-0.008	0.031
**rs2253477**	A	T	0.245	-0.173	0.04	-0.006	0.027
**rs2469785**	C	G	0.718	-0.161	0.04	-0.012	0.029
**rs2545802**	A	G	0.08	-0.433	0.102	-0.062	0.056
**rs28599745**	A	G	0.154	-0.363	0.08	-0.01	0.042

**rs28827783**	T	C	0.133	0.23	0.054	0.004	0.038
**rs2927708**	T	C	0.66	-0.148	0.036	-0.035	0.026
**rs2940229**	C	G	0.724	-0.202	0.046	0.015	0.027
**rs3097331**	T	C	0.626	0.157	0.035	-0.07	0.024
**rs35306**	A	G	0.539	-0.143	0.034	0	0.023
**rs35727592**	T	C	0.029	0.469	0.109	-0.085	0.083
**rs35894340**	A	G	0.746	-0.175	0.039	-0.033	0.027
**rs369128**	A	T	0.714	-0.155	0.037	-0.025	0.025
**rs3785423**	T	C	0.037	-0.471	0.107	-0.122	0.066
**rs3787457**	T	C	0.247	0.164	0.039	-0.015	0.028
**rs3935665**	A	G	0.676	0.152	0.036	-0.01	0.025
**rs4074650**	A	G	0.356	0.151	0.036	0.02	0.024
**rs4449480**	T	C	0.65	0.158	0.036	-0.002	0.024
**rs4733767**	A	G	0.27	0.194	0.039	0.022	0.027
**rs4734125**	A	G	0.123	-0.221	0.054	-0.075	0.036
**rs4859478**	A	G	0.225	0.163	0.04	-0.061	0.027
**rs487974**	A	G	0.345	0.175	0.041	0.017	0.03
**rs4903664**	T	C	0.468	0.144	0.033	0.03	0.023
**rs4969099**	A	G	0.67	0.152	0.037	-0.056	0.024
**rs4978387**	T	C	0.815	0.304	0.074	-0.047	0.036
**rs55687617**	A	G	0.114	-0.27	0.058	0.043	0.039
**rs56025909**	T	C	0.03	0.412	0.092	-0.111	0.071
**rs56343802**	A	T	0.718	-0.17	0.037	-0.014	0.025
**rs57514340**	T	C	0.769	0.176	0.042	-0.025	0.029
**rs5757717**	T	C	0.462	-0.146	0.035	0.023	0.024
**rs58776066**	A	C	0.13	0.204	0.05	0.018	0.037
**rs6111584**	T	C	0.973	-0.482	0.11	-0.037	0.079
**rs61146272**	T	C	0.922	0.294	0.067	0.013	0.044
**rs61742168**	A	G	0.987	-0.662	0.161	-0.151	0.116
**rs61933097**	A	G	0.948	-0.314	0.074	0.077	0.056
**rs62249119**	T	C	0.03	-0.484	0.117	-0.047	0.072
**rs639560**	T	C	0.046	-0.416	0.088	0.029	0.056
**rs6580184**	A	G	0.544	0.147	0.034	-0.029	0.023
**rs6871469**	A	C	0.241	-0.169	0.04	-0.003	0.027
**rs6984782**	T	C	0.854	-0.208	0.048	0.031	0.033
**rs6997554**	T	C	0.639	0.151	0.035	0.003	0.024
**rs7124427**	A	G	0.234	0.171	0.039	0.013	0.027
**rs7206058**	T	G	0.821	0.214	0.052	-0.038	0.035
**rs72781967**	T	C	0.657	-0.167	0.035	0.033	0.024
**rs72783425**	A	C	0.055	0.34	0.073	-0.012	0.054
**rs73120742**	A	G	0.023	0.486	0.119	0.025	0.09
**rs73404203**	T	C	0.036	0.393	0.097	0.01	0.066
**rs74156199**	T	C	0.014	0.534	0.125	-0.046	0.099
**rs74750933**	A	G	0.215	-0.168	0.041	0.004	0.029
**rs74993536**	C	G	0.016	0.821	0.201	-0.115	0.118

**rs75740353**	A	G	0.097	-0.433	0.098	0.019	0.059
**rs7658261**	C	G	0.42	0.151	0.036	-0.006	0.024
**rs77885126**	T	C	0.984	-0.603	0.131	0.128	0.101
**rs7790821**	A	G	0.245	0.155	0.038	0.019	0.027
**rs78784799**	A	G	0.013	0.684	0.158	-0.017	0.124
**rs78807573**	T	G	0.129	-0.41	0.1	-0.006	0.047
**rs79306469**	A	G	0.071	0.292	0.069	-0.141	0.052
**rs7937505**	A	C	0.256	0.163	0.039	-0.065	0.027
**rs8030746**	A	C	0.723	-0.151	0.037	0.022	0.027
**rs80344830**	A	G	0.935	-0.3	0.068	-0.028	0.047
**rs909701**	C	G	0.506	-0.155	0.034	-0.008	0.023
**rs9399861**	A	G	0.448	0.144	0.035	0.045	0.024
**rs9544927**	A	G	0.793	0.193	0.043	-0.026	0.028
**rs9733630**	A	G	0.269	0.165	0.039	-0.041	0.029
**rs9836705**	A	G	0.468	0.187	0.043	0.026	0.025
**rs9904844**	T	C	0.128	0.205	0.049	0.016	0.034
**rs9952159**	T	C	0.222	0.182	0.04	0.039	0.027

Table S53. Raw data for the MR analysis of the causal effect of OCD on UC (P<5E-5)							
**SNP**	**Effect allele**	**Other allele**	**Effect allele frequency**	**Beta.exposure**	**Se.exposure**	**Beta.outcome**	**Se.outcome**
**rs10185006**	A	G	0.437	0.141	0.021	0.003	0.034
**rs1030757**	A	C	0.513	-0.165	0.021	-0.013	0.034
**rs10474856**	A	T	0.514	0.148	0.021	0.014	0.034
**rs10479583**	C	G	0.581	-0.144	0.021	0.002	0.034
**rs10773765**	T	C	0.254	0.184	0.025	-0.057	0.04
**rs111926263**	A	G	0.205	0.187	0.029	-0.016	0.043
**rs112883781**	T	C	0.106	-0.242	0.035	-0.009	0.059
**rs116045211**	T	C	0.02	0.804	0.095	-0.029	0.186
**rs116347760**	A	T	0.019	0.63	0.086	0.026	0.134
**rs117310268**	T	C	0.035	0.449	0.063	0.098	0.097
**rs117502683**	T	C	0.014	0.719	0.101	0.185	0.167
**rs118060392**	A	G	0.974	-0.448	0.069	0.031	0.106
**rs12033048**	T	C	0.846	0.218	0.03	0.053	0.05
**rs12055962**	A	G	0.683	0.152	0.023	-0.014	0.036
**rs12095546**	A	G	0.246	0.175	0.027	-0.025	0.043
**rs12195828**	A	G	0.359	0.145	0.022	0.003	0.035
**rs12504244**	C	G	0.602	-0.169	0.022	-0.015	0.035
**rs12568997**	A	G	0.301	-0.293	0.025	-0.032	0.058
**rs12596951**	T	C	0.419	0.156	0.023	0.012	0.036
**rs12638795**	A	G	0.086	0.237	0.038	-0.009	0.058
**rs12804088**	T	C	0.985	-0.602	0.1	-0.069	0.148
**rs13107289**	A	C	0.615	-0.145	0.022	-0.01	0.035
**rs13127241**	A	G	0.154	0.187	0.029	0.024	0.046
**rs13141765**	T	C	0.604	-0.266	0.026	-0.019	0.056
**rs13256859**	T	C	0.144	0.21	0.033	-0.037	0.05
**rs138445568**	A	T	0.988	-0.928	0.134	-0.015	0.207
**rs139286049**	A	G	0.982	-0.601	0.096	-0.023	0.135
**rs139617**	A	C	0.381	0.155	0.023	0.011	0.036
**rs141664172**	A	G	0.022	0.488	0.073	0.038	0.113
**rs1652783**	A	G	0.781	-0.269	0.03	-0.027	0.059
**rs17748070**	T	C	0.02	0.513	0.081	-0.061	0.126
**rs17756387**	T	C	0.964	-0.396	0.059	-0.135	0.095
**rs17833131**	A	G	0.116	0.214	0.033	0.046	0.053
**rs1918508**	A	G	0.549	0.151	0.022	0.053	0.036
**rs203665**	T	C	0.119	0.252	0.036	-0.018	0.06
**rs2040325**	T	C	0.923	-0.268	0.04	-0.009	0.062
**rs2200119**	A	T	0.734	0.262	0.028	-0.01	0.062
**rs2253477**	A	T	0.25	-0.173	0.024	0.052	0.04
**rs2469785**	C	G	0.722	-0.161	0.027	0.005	0.04
**rs2545802**	A	G	0.081	-0.433	0.05	-0.03	0.102
**rs28599745**	A	G	0.155	-0.363	0.038	0.057	0.08
**rs28827783**	T	C	0.128	0.23	0.036	-0.043	0.054

**rs2927708**	T	C	0.676	-0.148	0.024	-0.033	0.036
**rs2940229**	C	G	0.722	-0.202	0.026	0.029	0.046
**rs3097331**	T	C	0.632	0.157	0.022	0.026	0.035
**rs35306**	A	G	0.539	-0.143	0.021	-0.011	0.034
**rs35727592**	T	C	0.029	0.469	0.074	0.028	0.109
**rs35894340**	A	G	0.753	-0.175	0.025	0.005	0.039
**rs369128**	A	T	0.716	-0.155	0.023	0.003	0.037
**rs3785423**	T	C	0.032	-0.471	0.065	-0.056	0.107
**rs3787457**	T	C	0.241	0.164	0.025	0.003	0.039
**rs3935665**	A	G	0.677	0.152	0.023	-0.027	0.036
**rs4074650**	A	G	0.358	0.151	0.023	0.001	0.036
**rs4449480**	T	C	0.645	0.158	0.022	-0.02	0.036
**rs4733767**	A	G	0.274	0.194	0.025	0.016	0.039
**rs4734125**	A	G	0.123	-0.221	0.033	-0.033	0.054
**rs4859478**	A	G	0.228	0.163	0.025	-0.017	0.04
**rs487974**	A	G	0.345	0.175	0.025	0.015	0.041
**rs4903664**	T	C	0.47	0.144	0.021	0.011	0.033
**rs4969099**	A	G	0.671	0.152	0.023	-0.003	0.037
**rs4978387**	T	C	0.807	0.304	0.033	-0.024	0.074
**rs55687617**	A	G	0.118	-0.27	0.036	0.043	0.058
**rs56025909**	T	C	0.03	0.412	0.064	-0.013	0.092
**rs56343802**	A	T	0.716	-0.17	0.023	-0.007	0.037
**rs57514340**	T	C	0.768	0.176	0.028	-0.011	0.042
**rs5757717**	T	C	0.469	-0.146	0.022	0.036	0.035
**rs58776066**	A	C	0.132	0.204	0.035	0.029	0.05
**rs6111584**	T	C	0.973	-0.482	0.068	-0.085	0.11
**rs61146272**	T	C	0.921	0.294	0.04	0.017	0.067
**rs61742168**	A	G	0.987	-0.662	0.105	-0.201	0.161
**rs61933097**	A	G	0.95	-0.314	0.053	-0.065	0.074
**rs62249119**	T	C	0.032	-0.484	0.065	-0.054	0.117
**rs639560**	T	C	0.048	-0.416	0.049	-0.017	0.088
**rs6580184**	A	G	0.533	0.147	0.021	-0.006	0.034
**rs6871469**	A	C	0.244	-0.169	0.025	0.032	0.04
**rs6984782**	T	C	0.858	-0.208	0.031	-0.011	0.048
**rs6997554**	T	C	0.64	0.151	0.022	-0.033	0.035
**rs7124427**	A	G	0.233	0.171	0.025	0.029	0.039
**rs7206058**	T	G	0.82	0.214	0.031	-0.029	0.052
**rs72781967**	T	C	0.655	-0.167	0.022	0.02	0.035
**rs72783425**	A	C	0.056	0.34	0.05	0.015	0.073
**rs73120742**	A	G	0.022	0.486	0.082	0.141	0.119
**rs73404203**	T	C	0.036	0.393	0.061	-0.067	0.097
**rs74156199**	T	C	0.014	0.534	0.09	0.116	0.125
**rs74750933**	A	G	0.22	-0.168	0.026	0.012	0.041
**rs74993536**	C	G	0.016	0.821	0.103	-0.051	0.201
**rs75740353**	A	G	0.095	-0.433	0.055	-0.096	0.098

**rs7658261**	C	G	0.422	0.151	0.023	0.006	0.036
**rs77885126**	T	C	0.984	-0.603	0.089	-0.038	0.131
**rs7790821**	A	G	0.245	0.155	0.025	0.016	0.038
**rs78784799**	A	G	0.013	0.684	0.113	-0.111	0.158
**rs78807573**	T	G	0.121	-0.41	0.042	-0.034	0.1
**rs79306469**	A	G	0.074	0.292	0.044	0.07	0.069
**rs7937505**	A	C	0.261	0.163	0.024	0.024	0.039
**rs8030746**	A	C	0.723	-0.151	0.025	-0.005	0.037
**rs80344830**	A	G	0.937	-0.3	0.045	-0.097	0.068
**rs909701**	C	G	0.506	-0.155	0.022	-0.007	0.034
**rs9399861**	A	G	0.443	0.144	0.022	-0.011	0.035
**rs9544927**	A	G	0.793	0.193	0.026	-0.014	0.043
**rs9733630**	A	G	0.27	0.165	0.025	-0.013	0.039
**rs9836705**	A	G	0.468	0.187	0.023	-0.008	0.043
**rs9904844**	T	C	0.121	0.205	0.033	-0.032	0.049
**rs9952159**	T	C	0.218	0.182	0.026	-0.02	0.04

Table S54. Raw data for the MR analysis of the causal effect of PTSD on IBD (P<5E-5)							
**SNP**	**Effect allele**	**Other allele**	**Effect allele frequency**	**Beta.exposure**	**Se.exposure**	**Beta.outcome**	**Se.outcome**
**rs10012643**	T	C	0.383	0.064	0.015	0.011	0.018
**rs10051297**	T	C	0.948	-0.134	0.032	-0.009	0.046
**rs1020396**	T	C	0.384	-0.062	0.015	0.051	0.018
**rs10228265**	A	G	0.689	0.067	0.016	0.009	0.018
**rs11129618**	A	T	0.161	0.086	0.02	0.005	0.024
**rs11135380**	A	G	0.032	0.163	0.04	-0.061	0.048
**rs11216592**	A	G	0.973	-0.216	0.05	0.006	0.057
**rs112528891**	A	G	0.966	-0.135	0.032	-0.019	0.056
**rs113485448**	T	C	0.035	0.209	0.05	-0.028	0.053
**rs1136201**	A	G	0.781	-0.08	0.017	-0.019	0.022
**rs114120794**	T	C	0.978	-0.238	0.055	0.062	0.068
**rs114514029**	T	G	0.97	-0.204	0.045	-0.026	0.05
**rs114981056**	A	T	0.974	-0.201	0.044	-0.182	0.056
**rs115467410**	T	C	0.979	-0.219	0.053	0.001	0.073
**rs11593164**	A	G	0.982	-0.229	0.056	0.009	0.065
**rs11658294**	C	G	0.503	-0.077	0.018	0.023	0.02
**rs11663467**	T	G	0.153	0.086	0.02	-0.018	0.023
**rs117305550**	A	G	0.943	-0.129	0.031	-0.015	0.04
**rs117405401**	A	G	0.013	0.256	0.055	-0.03	0.103
**rs117453028**	T	C	0.015	0.286	0.063	-0.157	0.091
**rs11852099**	A	G	0.213	-0.077	0.019	-0.048	0.022
**rs12585759**	T	C	0.124	0.09	0.022	-0.005	0.026
**rs12622325**	C	G	0.772	0.072	0.018	-0.031	0.021
**rs1268149**	A	G	0.006	0.38	0.083	-0.048	0.168
**rs12706983**	T	G	0.718	-0.09	0.019	0.032	0.022
**rs13005706**	T	C	0.593	-0.065	0.015	-0.006	0.017
**rs13330653**	A	G	0.771	-0.075	0.017	-0.018	0.021
**rs1363508**	A	G	0.313	-0.072	0.016	0.025	0.018
**rs138642250**	A	G	0.972	-0.189	0.045	0.07	0.055
**rs138823514**	T	C	0.014	0.273	0.067	-0.05	0.078
**rs139533248**	A	G	0.01	0.307	0.074	-0.133	0.127
**rs139591016**	T	C	0.981	-0.249	0.053	0.173	0.074
**rs139862824**	C	G	0.01	0.282	0.069	-0.08	0.103
**rs140281028**	A	C	0.038	0.17	0.038	-0.041	0.049
**rs140928208**	A	G	0.014	0.447	0.093	-0.083	0.08
**rs141291482**	A	G	0.021	0.215	0.051	0.099	0.063
**rs141828260**	T	C	0.053	-0.156	0.038	-0.008	0.043
**rs1420063**	A	G	0.491	0.066	0.015	-0.013	0.017
**rs142333870**	A	T	0.976	-0.222	0.049	0.106	0.071
**rs142491053**	T	C	0.018	0.242	0.056	-0.066	0.069
**rs1444764**	A	G	0.284	0.078	0.016	-0.015	0.019
**rs144835169**	A	C	0.982	-0.277	0.066	-0.083	0.088

**rs145560846**	T	C	0.99	0.396	0.094	-0.045	0.102
**rs146676625**	A	G	0.014	0.233	0.052	0.063	0.073
**rs147714053**	T	C	0.008	0.282	0.07	-0.089	0.131
**rs148445429**	A	G	0.985	-0.281	0.067	0.023	0.09
**rs1486086**	C	G	0.153	-0.095	0.021	-0.009	0.025
**rs148731153**	T	C	0.021	0.236	0.057	-0.038	0.064
**rs149509653**	A	G	0.157	0.09	0.019	0.028	0.024
**rs150653733**	T	C	0.021	0.299	0.073	0.004	0.081
**rs150749132**	T	G	0.027	0.208	0.051	0.053	0.061
**rs1509224**	T	C	0.892	0.103	0.025	0.029	0.027
**rs17041470**	A	G	0.987	-0.298	0.064	-0.19	0.074
**rs1704774**	T	C	0.241	-0.08	0.019	0.018	0.02
**rs17108326**	A	G	0.835	0.106	0.021	-0.011	0.023
**rs17541978**	T	C	0.935	-0.135	0.031	0.023	0.04
**rs180887621**	T	C	0.007	0.22	0.053	-0.025	0.142
**rs181196831**	A	T	0.987	-0.292	0.071	0.195	0.096
**rs184613502**	A	G	0.013	0.295	0.066	-0.081	0.086
**rs186670624**	C	G	0.99	-0.292	0.069	0.01	0.097
**rs1995917**	A	G	0.939	-0.13	0.032	0.001	0.035
**rs2007504**	T	C	0.779	-0.076	0.018	0.009	0.02
**rs2163050**	A	G	0.14	0.1	0.021	0.035	0.024
**rs2177735**	A	G	0.594	0.069	0.016	0.036	0.017
**rs2293995**	T	C	0.949	0.168	0.037	-0.008	0.041
**rs2392362**	T	C	0.308	0.065	0.016	-0.034	0.018
**rs2396726**	T	C	0.533	-0.062	0.015	0.028	0.017
**rs2580277**	C	G	0.507	-0.061	0.015	-0.021	0.017
**rs296935**	A	G	0.95	0.145	0.036	-0.072	0.039
**rs33516**	A	T	0.303	0.072	0.016	0.048	0.018
**rs34517852**	A	T	0.342	0.109	0.019	-0.016	0.021
**rs34763789**	C	G	0.258	0.07	0.017	0.036	0.02
**rs35334143**	C	G	0.764	-0.08	0.017	0.023	0.02
**rs35446081**	T	C	0.966	-0.178	0.041	-0.074	0.048
**rs35775421**	A	G	0.051	0.136	0.033	0.053	0.039
**rs36127550**	T	G	0.163	-0.103	0.02	-0.005	0.027
**rs3886102**	T	C	0.209	0.073	0.018	0.034	0.021
**rs4346247**	A	T	0.168	-0.094	0.022	-0.008	0.023
**rs4400150**	A	G	0.067	0.121	0.03	-0.054	0.034
**rs4449999**	T	C	0.088	0.114	0.027	0.055	0.031
**rs4702059**	A	G	0.094	0.116	0.027	-0.002	0.033
**rs4877586**	A	G	0.572	0.064	0.015	0.027	0.017
**rs55875130**	A	G	0.013	0.25	0.06	0.032	0.092
**rs56340428**	A	T	0.021	0.249	0.058	0.09	0.08
**rs57753395**	A	G	0.014	0.376	0.071	-0.023	0.089
**rs58241853**	C	G	0.149	-0.092	0.021	-0.022	0.024
**rs58701938**	T	C	0.01	0.456	0.111	0.044	0.086

**rs6045841**	T	C	0.697	0.069	0.017	0.016	0.019
**rs60473728**	A	G	0.092	0.12	0.029	0.022	0.037
**rs61879144**	A	C	0.929	-0.117	0.029	-0.007	0.04
**rs61969491**	T	C	0.706	0.069	0.017	0.005	0.02
**rs62108388**	A	G	0.063	0.128	0.03	-0.011	0.039
**rs62583551**	A	G	0.223	-0.097	0.02	0.013	0.023
**rs6943589**	A	C	0.54	-0.062	0.015	-0.016	0.017
**rs72653424**	T	C	0.323	0.076	0.018	-0.025	0.02
**rs72657988**	T	G	0.082	0.147	0.033	-0.018	0.048
**rs72823722**	T	C	0.052	-0.139	0.033	0.029	0.038
**rs72885970**	T	C	0.861	-0.1	0.024	0.018	0.028
**rs72914051**	T	G	0.902	-0.133	0.029	-0.033	0.032
**rs73061109**	T	G	0.187	-0.079	0.019	0.005	0.026
**rs73153506**	T	C	0.021	0.227	0.054	0.007	0.069
**rs73154700**	A	G	0.08	0.126	0.027	0.072	0.032
**rs73157523**	T	C	0.823	-0.086	0.02	0.011	0.022
**rs73176723**	T	C	0.829	-0.086	0.02	0	0.024
**rs73199535**	T	C	0.026	0.195	0.048	-0.03	0.062
**rs73228427**	T	C	0.037	-0.187	0.044	0.048	0.046
**rs74503919**	A	G	0.983	-0.275	0.067	-0.061	0.069
**rs74799611**	A	T	0.024	0.237	0.058	-0.018	0.073
**rs74805019**	C	G	0.033	0.18	0.043	0.04	0.053
**rs74901405**	T	G	0.109	0.117	0.026	-0.012	0.027
**rs7608101**	T	C	0.751	0.075	0.017	0.003	0.02
**rs763753**	A	G	0.14	-0.114	0.022	0.055	0.025
**rs76399807**	T	G	0.009	0.446	0.109	0.28	0.129
**rs76778521**	T	C	0.092	0.109	0.026	-0.007	0.03
**rs77537694**	A	G	0.911	0.13	0.028	0.01	0.03
**rs77681279**	A	G	0.984	-0.325	0.076	0.032	0.083
**rs77941536**	A	G	0.988	-0.339	0.081	-0.057	0.097
**rs780687**	T	C	0.912	-0.105	0.026	-0.06	0.03
**rs7807190**	A	G	0.766	0.083	0.018	0.009	0.021
**rs78333845**	T	C	0.972	-0.18	0.043	0.005	0.054
**rs78455884**	T	C	0.05	0.136	0.034	0.019	0.039
**rs78608260**	A	G	0.987	-0.321	0.069	-0.026	0.096
**rs78791719**	A	G	0.949	-0.137	0.032	0.037	0.038
**rs78990387**	A	G	0.966	-0.175	0.041	0.081	0.048
**rs79177862**	A	C	0.934	-0.128	0.03	0.049	0.035
**rs79246930**	T	C	0.98	-0.252	0.059	0.057	0.064
**rs79317376**	T	G	0.009	0.325	0.073	0.237	0.1
**rs7953905**	A	G	0.76	0.08	0.019	0.031	0.02
**rs79635463**	A	G	0.985	-0.308	0.071	0.079	0.079
**rs79874145**	T	C	0.992	-0.338	0.08	0.015	0.184
**rs80074987**	T	G	0.014	0.238	0.051	-0.123	0.075
**rs8134631**	T	G	0.538	-0.064	0.016	0.011	0.018

**rs9367037**	T	C	0.847	0.085	0.021	0.002	0.023
**rs9544469**	T	C	0.242	-0.077	0.018	-0.037	0.022

Table S55. Raw data for the MR analysis of the causal effect of PTSD on CD (P<5E-5)							
**SNP**	**Effect allele**	**Other allele**	**Effect allele frequency**	**Beta.exposure**	**Se.exposure**	**Beta.outcome**	**Se.outcome**
**rs10012643**	T	C	0.381	0.064	0.015	-0.002	0.024
**rs10051297**	T	C	0.949	-0.134	0.032	0.028	0.063
**rs1020396**	T	C	0.376	-0.062	0.015	0.042	0.024
**rs10228265**	A	G	0.686	0.067	0.016	0.005	0.025
**rs11129618**	A	T	0.162	0.086	0.02	-0.023	0.032
**rs11135380**	A	G	0.032	0.163	0.04	-0.181	0.068
**rs11216592**	A	G	0.974	-0.216	0.05	-0.022	0.079
**rs112528891**	A	G	0.965	-0.135	0.032	0.088	0.076
**rs113485448**	T	C	0.034	0.209	0.05	0.014	0.072
**rs1136201**	A	G	0.791	-0.08	0.017	-0.012	0.031
**rs114120794**	T	C	0.978	-0.238	0.055	-0.03	0.091
**rs114514029**	T	G	0.969	-0.204	0.045	-0.067	0.068
**rs114981056**	A	T	0.976	-0.201	0.044	-0.179	0.076
**rs115467410**	T	C	0.981	-0.219	0.053	0.029	0.106
**rs11593164**	A	G	0.981	-0.229	0.056	-0.074	0.085
**rs11658294**	C	G	0.5	-0.077	0.018	-0.003	0.028
**rs11663467**	T	G	0.155	0.086	0.02	0.015	0.031
**rs117305550**	A	G	0.944	-0.129	0.031	-0.091	0.053
**rs117405401**	A	G	0.013	0.256	0.055	-0.189	0.148
**rs117453028**	T	C	0.015	0.286	0.063	-0.109	0.125
**rs11852099**	A	G	0.214	-0.077	0.019	-0.071	0.03
**rs12585759**	T	C	0.123	0.09	0.022	0.001	0.035
**rs12622325**	C	G	0.768	0.072	0.018	0.001	0.028
**rs1268149**	A	G	0.006	0.38	0.083	-0.08	0.248
**rs12706983**	T	G	0.712	-0.09	0.019	0.043	0.03
**rs13005706**	T	C	0.596	-0.065	0.015	0	0.023
**rs13330653**	A	G	0.771	-0.075	0.017	0.021	0.028
**rs1363508**	A	G	0.313	-0.072	0.016	0.029	0.025
**rs138642250**	A	G	0.972	-0.189	0.045	0.037	0.074
**rs138823514**	T	C	0.014	0.273	0.067	-0.044	0.108
**rs139533248**	A	G	0.01	0.307	0.074	-0.106	0.167
**rs139591016**	T	C	0.983	-0.249	0.053	0.153	0.107
**rs139862824**	C	G	0.009	0.282	0.069	-0.147	0.152
**rs140281028**	A	C	0.039	0.17	0.038	0.086	0.066
**rs140928208**	A	G	0.014	0.447	0.093	-0.135	0.116
**rs141291482**	A	G	0.022	0.215	0.051	0.073	0.086
**rs141828260**	T	C	0.053	-0.156	0.038	0.009	0.059
**rs1420063**	A	G	0.49	0.066	0.015	-0.023	0.023
**rs142333870**	A	T	0.977	-0.222	0.049	0.105	0.099
**rs142491053**	T	C	0.018	0.242	0.056	0.102	0.092
**rs1444764**	A	G	0.285	0.078	0.016	-0.034	0.026
**rs144835169**	A	C	0.982	-0.277	0.066	-0.162	0.12

**rs145560846**	T	C	0.99	0.396	0.094	-0.1	0.144
**rs146676625**	A	G	0.012	0.233	0.052	0.12	0.108
**rs147714053**	T	C	0.008	0.282	0.07	-0.116	0.19
**rs148445429**	A	G	0.986	-0.281	0.067	-0.18	0.129
**rs1486086**	C	G	0.151	-0.095	0.021	-0.018	0.034
**rs148731153**	T	C	0.023	0.236	0.057	-0.096	0.083
**rs149509653**	A	G	0.158	0.09	0.019	0.001	0.033
**rs150653733**	T	C	0.021	0.299	0.073	0.023	0.11
**rs150749132**	T	G	0.024	0.208	0.051	-0.149	0.095
**rs1509224**	T	C	0.894	0.103	0.025	0.067	0.038
**rs17041470**	A	G	0.988	-0.298	0.064	-0.156	0.107
**rs1704774**	T	C	0.242	-0.08	0.019	0.019	0.028
**rs17108326**	A	G	0.832	0.106	0.021	-0.005	0.03
**rs17541978**	T	C	0.938	-0.135	0.031	0.018	0.057
**rs180887621**	T	C	0.007	0.22	0.053	0.014	0.195
**rs181196831**	A	T	0.987	-0.292	0.071	0.008	0.129
**rs184613502**	A	G	0.013	0.295	0.066	-0.132	0.122
**rs186670624**	C	G	0.989	-0.292	0.069	0.041	0.129
**rs1995917**	A	G	0.938	-0.13	0.032	-0.022	0.047
**rs2007504**	T	C	0.777	-0.076	0.018	-0.015	0.027
**rs2163050**	A	G	0.141	0.1	0.021	0.021	0.033
**rs2177735**	A	G	0.588	0.069	0.016	0.028	0.023
**rs2293995**	T	C	0.951	0.168	0.037	0.054	0.057
**rs2392362**	T	C	0.309	0.065	0.016	-0.024	0.025
**rs2396726**	T	C	0.536	-0.062	0.015	0.042	0.023
**rs2580277**	C	G	0.505	-0.061	0.015	-0.018	0.023
**rs296935**	A	G	0.949	0.145	0.036	-0.021	0.053
**rs33516**	A	T	0.3	0.072	0.016	0.018	0.025
**rs34517852**	A	T	0.342	0.109	0.019	-0.015	0.029
**rs34763789**	C	G	0.256	0.07	0.017	0.06	0.027
**rs35334143**	C	G	0.771	-0.08	0.017	0.032	0.027
**rs35446081**	T	C	0.966	-0.178	0.041	-0.027	0.066
**rs35775421**	A	G	0.05	0.136	0.033	0.051	0.053
**rs36127550**	T	G	0.168	-0.103	0.02	-0.019	0.037
**rs3886102**	T	C	0.21	0.073	0.018	0.006	0.028
**rs4346247**	A	T	0.166	-0.094	0.022	-0.005	0.031
**rs4400150**	A	G	0.069	0.121	0.03	-0.076	0.046
**rs4449999**	T	C	0.089	0.114	0.027	0.091	0.042
**rs4702059**	A	G	0.093	0.116	0.027	-0.003	0.045
**rs4877586**	A	G	0.574	0.064	0.015	0.014	0.023
**rs55875130**	A	G	0.014	0.25	0.06	0.058	0.124
**rs56340428**	A	T	0.021	0.249	0.058	0.21	0.111
**rs57753395**	A	G	0.013	0.376	0.071	-0.086	0.128
**rs58241853**	C	G	0.146	-0.092	0.021	-0.073	0.032
**rs58701938**	T	C	0.01	0.456	0.111	0.004	0.118

**rs6045841**	T	C	0.697	0.069	0.017	0.01	0.025
**rs60473728**	A	G	0.095	0.12	0.029	0.008	0.05
**rs61879144**	A	C	0.933	-0.117	0.029	-0.074	0.056
**rs61969491**	T	C	0.699	0.069	0.017	-0.025	0.027
**rs62108388**	A	G	0.06	0.128	0.03	-0.024	0.055
**rs62583551**	A	G	0.227	-0.097	0.02	0.018	0.032
**rs6943589**	A	C	0.543	-0.062	0.015	0.013	0.023
**rs72653424**	T	C	0.318	0.076	0.018	0.008	0.028
**rs72657988**	T	G	0.083	0.147	0.033	-0.002	0.065
**rs72823722**	T	C	0.05	-0.139	0.033	0.034	0.053
**rs72885970**	T	C	0.857	-0.1	0.024	0.031	0.038
**rs72914051**	T	G	0.904	-0.133	0.029	-0.008	0.045
**rs73061109**	T	G	0.182	-0.079	0.019	0.043	0.036
**rs73153506**	T	C	0.021	0.227	0.054	-0.133	0.098
**rs73154700**	A	G	0.081	0.126	0.027	0.045	0.043
**rs73157523**	T	C	0.821	-0.086	0.02	-0.015	0.03
**rs73176723**	T	C	0.827	-0.086	0.02	-0.021	0.033
**rs73199535**	T	C	0.027	0.195	0.048	-0.09	0.085
**rs73228427**	T	C	0.038	-0.187	0.044	0.019	0.062
**rs74503919**	A	G	0.983	-0.275	0.067	-0.046	0.097
**rs74799611**	A	T	0.022	0.237	0.058	-0.016	0.105
**rs74805019**	C	G	0.032	0.18	0.043	-0.008	0.074
**rs74901405**	T	G	0.107	0.117	0.026	-0.018	0.038
**rs7608101**	T	C	0.754	0.075	0.017	0.031	0.028
**rs763753**	A	G	0.141	-0.114	0.022	0.06	0.033
**rs76399807**	T	G	0.009	0.446	0.109	0.473	0.169
**rs76778521**	T	C	0.091	0.109	0.026	-0.016	0.041
**rs77537694**	A	G	0.912	0.13	0.028	0.054	0.042
**rs77681279**	A	G	0.984	-0.325	0.076	0.019	0.118
**rs77941536**	A	G	0.988	-0.339	0.081	-0.076	0.142
**rs780687**	T	C	0.915	-0.105	0.026	-0.05	0.042
**rs7807190**	A	G	0.765	0.083	0.018	0.054	0.028
**rs78333845**	T	C	0.972	-0.18	0.043	-0.005	0.074
**rs78455884**	T	C	0.05	0.136	0.034	0.035	0.053
**rs78608260**	A	G	0.988	-0.321	0.069	0.061	0.14
**rs78791719**	A	G	0.951	-0.137	0.032	-0.008	0.053
**rs78990387**	A	G	0.967	-0.175	0.041	-0.001	0.065
**rs79177862**	A	C	0.935	-0.128	0.03	0.042	0.049
**rs79246930**	T	C	0.978	-0.252	0.059	-0.014	0.084
**rs79317376**	T	G	0.008	0.325	0.073	0.332	0.14
**rs7953905**	A	G	0.764	0.08	0.019	0.003	0.027
**rs79635463**	A	G	0.985	-0.308	0.071	0.158	0.111
**rs79874145**	T	C	0.992	-0.338	0.08	-0.31	0.215
**rs80074987**	T	G	0.013	0.238	0.051	-0.22	0.106
**rs8134631**	T	G	0.538	-0.064	0.016	0.007	0.024

**rs9367037**	T	C	0.846	0.085	0.021	-0.008	0.032
**rs9544469**	T	C	0.24	-0.077	0.018	-0.043	0.031

Table S56. Raw data for the MR analysis of the causal effect of PTSD on UC (P<5E-5)							
**SNP**	**Effect allele**	**Other allele**	**Effect allele frequency**	**Beta.exposure**	**Se.exposure**	**Beta.outcome**	**Se.outcome**
**rs10012643**	T	C	0.385	0.064	0.015	0.022	0.022
**rs10051297**	T	C	0.947	-0.134	0.032	-0.017	0.057
**rs1020396**	T	C	0.387	-0.062	0.015	0.053	0.022
**rs10228265**	A	G	0.69	0.067	0.016	0.018	0.023
**rs11129618**	A	T	0.16	0.086	0.02	0.024	0.03
**rs11135380**	A	G	0.033	0.163	0.04	0.057	0.059
**rs11216592**	A	G	0.973	-0.216	0.05	0.006	0.071
**rs112528891**	A	G	0.965	-0.135	0.032	-0.088	0.07
**rs113485448**	T	C	0.035	0.209	0.05	-0.037	0.067
**rs1136201**	A	G	0.778	-0.08	0.017	-0.009	0.028
**rs114120794**	T	C	0.979	-0.238	0.055	0.074	0.088
**rs114514029**	T	G	0.969	-0.204	0.045	-0.03	0.063
**rs114981056**	A	T	0.974	-0.201	0.044	-0.159	0.068
**rs115467410**	T	C	0.979	-0.219	0.053	-0.038	0.088
**rs11593164**	A	G	0.983	-0.229	0.056	0.135	0.084
**rs11658294**	C	G	0.504	-0.077	0.018	0.04	0.025
**rs11663467**	T	G	0.152	0.086	0.02	-0.038	0.029
**rs117305550**	A	G	0.943	-0.129	0.031	0.045	0.051
**rs117405401**	A	G	0.013	0.256	0.055	0.035	0.126
**rs117453028**	T	C	0.014	0.286	0.063	-0.064	0.115
**rs11852099**	A	G	0.215	-0.077	0.019	-0.039	0.027
**rs12585759**	T	C	0.124	0.09	0.022	-0.01	0.032
**rs12622325**	C	G	0.772	0.072	0.018	-0.041	0.026
**rs1268149**	A	G	0.006	0.38	0.083	-0.041	0.204
**rs12706983**	T	G	0.72	-0.09	0.019	0.025	0.028
**rs13005706**	T	C	0.591	-0.065	0.015	-0.013	0.022
**rs13330653**	A	G	0.771	-0.075	0.017	-0.039	0.026
**rs1363508**	A	G	0.312	-0.072	0.016	0.023	0.023
**rs138642250**	A	G	0.973	-0.189	0.045	0.121	0.07
**rs138823514**	T	C	0.015	0.273	0.067	-0.019	0.099
**rs139533248**	A	G	0.009	0.307	0.074	-0.114	0.17
**rs139591016**	T	C	0.981	-0.249	0.053	0.18	0.091
**rs139862824**	C	G	0.01	0.282	0.069	-0.08	0.123
**rs140281028**	A	C	0.038	0.17	0.038	-0.112	0.063
**rs140928208**	A	G	0.015	0.447	0.093	-0.048	0.098
**rs141291482**	A	G	0.021	0.215	0.051	0.074	0.079
**rs141828260**	T	C	0.053	-0.156	0.038	-0.006	0.054
**rs1420063**	A	G	0.492	0.066	0.015	-0.003	0.021
**rs142333870**	A	T	0.976	-0.222	0.049	0.132	0.089
**rs142491053**	T	C	0.018	0.242	0.056	-0.13	0.088
**rs1444764**	A	G	0.285	0.078	0.016	0.003	0.023
**rs144835169**	A	C	0.982	-0.277	0.066	-0.015	0.112

**rs145560846**	T	C	0.99	0.396	0.094	-0.138	0.128
**rs146676625**	A	G	0.015	0.233	0.052	0.06	0.09
**rs147714053**	T	C	0.008	0.282	0.07	0.01	0.163
**rs148445429**	A	G	0.984	-0.281	0.067	0.12	0.11
**rs1486086**	C	G	0.154	-0.095	0.021	0.01	0.031
**rs148731153**	T	C	0.021	0.236	0.057	-0.018	0.083
**rs149509653**	A	G	0.157	0.09	0.019	0.04	0.03
**rs150653733**	T	C	0.021	0.299	0.073	0.019	0.101
**rs150749132**	T	G	0.029	0.208	0.051	0.173	0.07
**rs1509224**	T	C	0.892	0.103	0.025	0.004	0.034
**rs17041470**	A	G	0.987	-0.298	0.064	-0.243	0.09
**rs1704774**	T	C	0.241	-0.08	0.019	0.003	0.026
**rs17108326**	A	G	0.836	0.106	0.021	-0.012	0.028
**rs17541978**	T	C	0.934	-0.135	0.031	0.031	0.049
**rs180887621**	T	C	0.007	0.22	0.053	-0.075	0.175
**rs181196831**	A	T	0.987	-0.292	0.071	0.249	0.125
**rs184613502**	A	G	0.013	0.295	0.066	0.036	0.104
**rs186670624**	C	G	0.99	-0.292	0.069	-0.037	0.122
**rs1995917**	A	G	0.939	-0.13	0.032	0.008	0.045
**rs2007504**	T	C	0.78	-0.076	0.018	0.032	0.025
**rs2163050**	A	G	0.14	0.1	0.021	0.035	0.03
**rs2177735**	A	G	0.595	0.069	0.016	0.049	0.022
**rs2293995**	T	C	0.948	0.168	0.037	-0.058	0.051
**rs2392362**	T	C	0.308	0.065	0.016	-0.032	0.023
**rs2396726**	T	C	0.53	-0.062	0.015	0.023	0.021
**rs2580277**	C	G	0.509	-0.061	0.015	-0.03	0.021
**rs296935**	A	G	0.949	0.145	0.036	-0.071	0.049
**rs33516**	A	T	0.304	0.072	0.016	0.063	0.023
**rs34517852**	A	T	0.345	0.109	0.019	-0.017	0.025
**rs34763789**	C	G	0.258	0.07	0.017	0.005	0.025
**rs35334143**	C	G	0.761	-0.08	0.017	0.014	0.025
**rs35446081**	T	C	0.966	-0.178	0.041	-0.096	0.06
**rs35775421**	A	G	0.052	0.136	0.033	0.027	0.049
**rs36127550**	T	G	0.161	-0.103	0.02	0.028	0.035
**rs3886102**	T	C	0.208	0.073	0.018	0.045	0.026
**rs4346247**	A	T	0.168	-0.094	0.022	-0.001	0.028
**rs4400150**	A	G	0.067	0.121	0.03	-0.041	0.044
**rs4449999**	T	C	0.087	0.114	0.027	0.046	0.039
**rs4702059**	A	G	0.094	0.116	0.027	0.009	0.041
**rs4877586**	A	G	0.572	0.064	0.015	0.026	0.022
**rs55875130**	A	G	0.013	0.25	0.06	0.029	0.115
**rs56340428**	A	T	0.021	0.249	0.058	-0.001	0.1
**rs57753395**	A	G	0.014	0.376	0.071	-0.018	0.109
**rs58241853**	C	G	0.15	-0.092	0.021	0.002	0.03
**rs58701938**	T	C	0.01	0.456	0.111	0.082	0.107

**rs6045841**	T	C	0.697	0.069	0.017	0.012	0.023
**rs60473728**	A	G	0.092	0.12	0.029	0.016	0.048
**rs61879144**	A	C	0.929	-0.117	0.029	0.004	0.051
**rs61969491**	T	C	0.711	0.069	0.017	0.035	0.025
**rs62108388**	A	G	0.065	0.128	0.03	0.018	0.049
**rs62583551**	A	G	0.221	-0.097	0.02	0.013	0.028
**rs6943589**	A	C	0.538	-0.062	0.015	-0.037	0.021
**rs72653424**	T	C	0.322	0.076	0.018	-0.028	0.025
**rs72657988**	T	G	0.081	0.147	0.033	0.03	0.06
**rs72823722**	T	C	0.052	-0.139	0.033	0.034	0.048
**rs72885970**	T	C	0.862	-0.1	0.024	0.001	0.036
**rs72914051**	T	G	0.9	-0.133	0.029	-0.036	0.04
**rs73061109**	T	G	0.188	-0.079	0.019	-0.001	0.032
**rs73153506**	T	C	0.021	0.227	0.054	0.121	0.084
**rs73154700**	A	G	0.079	0.126	0.027	0.072	0.041
**rs73157523**	T	C	0.825	-0.086	0.02	0.026	0.028
**rs73176723**	T	C	0.83	-0.086	0.02	0.014	0.031
**rs73199535**	T	C	0.026	0.195	0.048	0.003	0.078
**rs73228427**	T	C	0.038	-0.187	0.044	0.076	0.058
**rs74503919**	A	G	0.983	-0.275	0.067	-0.133	0.086
**rs74799611**	A	T	0.025	0.237	0.058	0.092	0.088
**rs74805019**	C	G	0.034	0.18	0.043	0.057	0.066
**rs74901405**	T	G	0.109	0.117	0.026	0	0.034
**rs7608101**	T	C	0.749	0.075	0.017	-0.008	0.026
**rs763753**	A	G	0.139	-0.114	0.022	0.055	0.031
**rs76399807**	T	G	0.009	0.446	0.109	0.163	0.162
**rs76778521**	T	C	0.092	0.109	0.026	0.015	0.037
**rs77537694**	A	G	0.911	0.13	0.028	-0.041	0.038
**rs77681279**	A	G	0.984	-0.325	0.076	0.146	0.102
**rs77941536**	A	G	0.988	-0.339	0.081	-0.125	0.118
**rs780687**	T	C	0.912	-0.105	0.026	-0.069	0.037
**rs7807190**	A	G	0.766	0.083	0.018	-0.026	0.026
**rs78333845**	T	C	0.972	-0.18	0.043	-0.024	0.067
**rs78455884**	T	C	0.05	0.136	0.034	0.013	0.049
**rs78608260**	A	G	0.986	-0.321	0.069	-0.04	0.117
**rs78791719**	A	G	0.948	-0.137	0.032	0.044	0.048
**rs78990387**	A	G	0.966	-0.175	0.041	0.118	0.061
**rs79177862**	A	C	0.933	-0.128	0.03	0.059	0.044
**rs79246930**	T	C	0.98	-0.252	0.059	0.061	0.082
**rs79317376**	T	G	0.008	0.325	0.073	0.273	0.125
**rs7953905**	A	G	0.76	0.08	0.019	0.042	0.025
**rs79635463**	A	G	0.986	-0.308	0.071	0.036	0.099
**rs79874145**	T	C	0.992	-0.338	0.08	0.296	0.261
**rs80074987**	T	G	0.014	0.238	0.051	-0.053	0.092
**rs8134631**	T	G	0.538	-0.064	0.016	0.023	0.022

**rs9367037**	T	C	0.848	0.085	0.021	-0.003	0.03
**rs9544469**	T	C	0.246	-0.077	0.018	-0.031	0.027

Table S57. Raw data for the MR analysis of the causal effect of schizophrenia on IBD (P<5E-5)							
**SNP**	**Effect allele**	**Other allele**	**Effect allele frequency**	**Beta.exposure**	**Se.exposure**	**Beta.outcome**	**Se.outcome**
**rs1000237**	T	A	0.640	-0.073	0.009	-0.030	0.018
**rs10005436**	G	A	0.667	-0.040	0.009	0.017	0.018
**rs10035564**	A	G	0.659	-0.067	0.009	-0.016	0.018
**rs10069930**	T	A	0.487	0.045	0.009	0.004	0.017
**rs10086619**	A	G	0.841	-0.072	0.012	-0.050	0.023
**rs10100465**	G	A	0.784	0.043	0.011	-0.004	0.020
**rs10108980**	C	T	0.805	-0.063	0.011	-0.010	0.021
**rs10117**	G	A	0.595	0.055	0.009	-0.003	0.017
**rs10133619**	T	G	0.609	0.044	0.009	0.008	0.017
**rs10148671**	T	C	0.343	-0.047	0.009	0.001	0.018
**rs10173857**	C	T	0.711	0.051	0.010	-0.018	0.019
**rs10238960**	C	T	0.290	-0.039	0.010	-0.004	0.019
**rs10520163**	T	C	0.499	0.047	0.009	0.014	0.017
**rs10751832**	A	G	0.141	0.057	0.013	-0.002	0.024
**rs10760502**	A	G	0.349	-0.041	0.010	-0.006	0.019
**rs10821976**	G	A	0.754	0.046	0.011	0.003	0.022
**rs10832722**	C	T	0.698	0.049	0.010	0.057	0.019
**rs10861176**	G	A	0.275	-0.056	0.010	-0.002	0.019
**rs10873538**	T	G	0.677	-0.067	0.009	-0.004	0.018
**rs10876446**	G	C	0.686	-0.054	0.009	0.019	0.019
**rs10931083**	T	C	0.053	0.089	0.022	-0.002	0.041
**rs10935477**	G	T	0.759	-0.043	0.010	0.069	0.020
**rs10999895**	C	T	0.613	-0.041	0.009	0.002	0.018
**rs11027839**	A	C	0.501	-0.052	0.009	0.005	0.017
**rs11061973**	G	A	0.848	0.055	0.012	-0.018	0.023
**rs11064676**	C	G	0.792	0.045	0.011	-0.015	0.021
**rs1106478**	T	G	0.794	-0.056	0.011	-0.015	0.021
**rs11105285**	C	T	0.781	0.057	0.011	0.009	0.021
**rs11129511**	G	A	0.661	0.039	0.009	0.030	0.018
**rs111320077**	C	T	0.896	-0.063	0.014	-0.035	0.028
**rs11136325**	G	A	0.419	0.054	0.009	-0.023	0.019
**rs11165690**	T	C	0.812	-0.054	0.011	0.002	0.021
**rs11165867**	C	T	0.836	-0.074	0.012	-0.017	0.023
**rs11191580**	T	C	0.912	0.132	0.016	0.036	0.030
**rs1120004**	T	G	0.256	0.047	0.010	0.021	0.019
**rs11210892**	G	A	0.330	0.064	0.009	0.028	0.018
**rs11217602**	C	T	0.590	0.037	0.009	-0.006	0.017
**rs112177478**	C	T	0.911	0.082	0.015	0.038	0.030
**rs11223774**	A	G	0.294	0.052	0.009	0.006	0.019
**rs11251858**	C	T	0.638	-0.039	0.009	-0.017	0.018
**rs11252007**	C	T	0.739	-0.046	0.010	-0.005	0.020
**rs113264400**	T	C	0.953	-0.112	0.020	-0.070	0.043

**rs113892895**	A	T	0.944	0.093	0.019	0.026	0.037
**rs114702133**	A	G	0.956	-0.101	0.023	0.031	0.045
**rs11487432**	A	G	0.913	0.062	0.015	0.012	0.030
**rs11534045**	G	A	0.665	0.063	0.009	0.023	0.018
**rs115361459**	G	A	0.974	0.136	0.030	0.019	0.058
**rs11587347**	C	G	0.899	-0.104	0.015	0.001	0.029
**rs11590169**	A	G	0.915	-0.067	0.016	-0.076	0.030
**rs11632125**	G	A	0.967	-0.118	0.026	-0.108	0.057
**rs116350522**	G	T	0.961	-0.102	0.023	0.104	0.050
**rs11647188**	A	G	0.602	0.048	0.009	-0.002	0.017
**rs11663962**	G	A	0.717	-0.043	0.010	0.017	0.019
**rs11664298**	G	A	0.805	-0.077	0.011	0.046	0.021
**rs11668896**	T	C	0.358	-0.048	0.010	0.018	0.021
**rs11680328**	T	A	0.126	0.064	0.013	0.013	0.026
**rs11686852**	G	A	0.555	0.046	0.009	0.006	0.017
**rs11687946**	G	T	0.582	0.043	0.009	-0.024	0.018
**rs11690184**	T	C	0.486	0.036	0.009	0.020	0.017
**rs11693094**	C	T	0.553	0.054	0.009	-0.005	0.017
**rs116966156**	C	T	0.983	-0.137	0.033	-0.097	0.068
**rs117170626**	G	A	0.973	-0.110	0.026	0.059	0.063
**rs117178087**	C	T	0.929	0.096	0.018	-0.009	0.033
**rs11719324**	A	C	0.574	-0.038	0.009	-0.011	0.017
**rs11732321**	C	G	0.566	-0.040	0.009	-0.004	0.017
**rs11740474**	A	T	0.592	-0.054	0.009	0.012	0.017
**rs11752044**	G	A	0.777	0.055	0.011	-0.015	0.020
**rs11780451**	T	G	0.085	-0.080	0.016	0.009	0.030
**rs118049780**	G	A	0.920	-0.074	0.018	-0.014	0.033
**rs11811826**	T	A	0.773	-0.045	0.010	-0.004	0.020
**rs118121724**	C	T	0.929	-0.073	0.017	-0.016	0.037
**rs11854073**	G	A	0.678	0.047	0.009	-0.008	0.018
**rs11878234**	A	C	0.874	0.058	0.014	-0.012	0.027
**rs11941714**	G	A	0.643	0.052	0.009	-0.013	0.019
**rs11964401**	A	G	0.485	0.045	0.009	-0.001	0.017
**rs11972718**	C	G	0.699	-0.048	0.009	0.008	0.019
**rs1198588**	A	T	0.197	-0.103	0.011	-0.038	0.021
**rs12029721**	A	G	0.922	-0.081	0.018	0.019	0.037
**rs1203630**	A	G	0.263	0.043	0.010	0.019	0.019
**rs12040431**	C	T	0.332	0.040	0.009	-0.055	0.018
**rs12120481**	A	G	0.860	-0.058	0.013	0.005	0.025
**rs12121218**	T	G	0.812	-0.049	0.011	-0.036	0.022
**rs12129573**	C	A	0.624	-0.078	0.009	0.021	0.017
**rs12138061**	C	A	0.791	-0.045	0.010	0.046	0.021
**rs12138231**	T	A	0.183	-0.067	0.012	-0.021	0.022
**rs12142659**	T	C	0.699	-0.045	0.010	-0.015	0.019
**rs12145726**	G	A	0.371	0.046	0.009	-0.006	0.017

**rs12151767**	G	A	0.509	0.061	0.009	0.035	0.017
**rs12153880**	G	A	0.808	0.052	0.011	0.009	0.022
**rs1217091**	T	C	0.188	-0.059	0.011	-0.015	0.022
**rs12186968**	C	T	0.703	0.047	0.010	0.008	0.019
**rs12190176**	A	G	0.640	-0.038	0.009	0.015	0.018
**rs12285419**	C	A	0.812	-0.085	0.011	-0.012	0.022
**rs12293670**	A	G	0.665	0.070	0.009	-0.003	0.018
**rs12303743**	G	C	0.904	-0.087	0.015	0.034	0.029
**rs12462062**	C	G	0.707	-0.040	0.010	-0.004	0.018
**rs12474906**	A	C	0.798	0.056	0.011	0.016	0.022
**rs12485775**	G	A	0.796	0.047	0.011	-0.010	0.021
**rs12489270**	T	C	0.621	-0.058	0.009	0.000	0.017
**rs12499194**	C	T	0.854	-0.056	0.013	0.004	0.026
**rs1253434**	T	C	0.676	0.043	0.009	0.035	0.019
**rs12577324**	T	C	0.792	0.046	0.011	0.008	0.022
**rs12600720**	C	G	0.683	-0.048	0.010	-0.036	0.020
**rs12615991**	A	G	0.854	-0.055	0.012	-0.021	0.024
**rs12624433**	G	A	0.736	-0.044	0.010	-0.055	0.019
**rs12652777**	T	C	0.475	0.049	0.009	-0.001	0.017
**rs12671608**	T	C	0.879	-0.064	0.013	-0.001	0.029
**rs12712510**	T	C	0.467	0.057	0.009	0.017	0.017
**rs12771371**	G	A	0.691	0.052	0.009	-0.020	0.018
**rs12833624**	C	T	0.651	-0.050	0.009	0.061	0.018
**rs12877581**	G	C	0.732	-0.060	0.010	0.012	0.021
**rs12883788**	C	T	0.570	-0.061	0.009	-0.013	0.018
**rs1289003**	G	T	0.589	-0.048	0.009	0.003	0.017
**rs12943566**	A	G	0.341	-0.050	0.009	-0.001	0.018
**rs13011472**	C	G	0.510	-0.070	0.009	-0.017	0.017
**rs13014947**	G	A	0.437	0.039	0.009	0.005	0.017
**rs13016542**	T	C	0.870	0.088	0.013	0.021	0.026
**rs13025903**	T	A	0.779	-0.051	0.010	-0.002	0.020
**rs13030312**	A	T	0.871	-0.057	0.013	-0.053	0.025
**rs13074132**	T	C	0.646	0.047	0.009	-0.002	0.018
**rs13090130**	G	A	0.624	0.041	0.009	0.017	0.017
**rs13107325**	C	T	0.916	-0.159	0.017	-0.119	0.031
**rs13145146**	A	G	0.948	-0.090	0.019	-0.001	0.038
**rs13150491**	C	T	0.551	-0.037	0.009	-0.029	0.017
**rs13154281**	C	G	0.550	0.039	0.009	-0.032	0.017
**rs13177597**	C	G	0.873	-0.056	0.014	0.013	0.026
**rs1319017**	G	A	0.662	-0.046	0.009	0.021	0.018
**rs13195636**	A	C	0.914	0.211	0.016	0.044	0.030
**rs13233308**	C	T	0.522	0.049	0.009	0.008	0.017
**rs132582**	C	T	0.460	0.051	0.009	0.002	0.017
**rs13273892**	C	A	0.673	-0.040	0.009	-0.026	0.018
**rs13320945**	C	T	0.607	-0.045	0.009	0.019	0.017

**rs13396659**	A	C	0.794	-0.046	0.011	-0.021	0.021
**rs1389718**	G	A	0.769	-0.044	0.010	-0.040	0.020
**rs1392414**	A	G	0.186	0.047	0.011	0.011	0.022
**rs1398541**	G	A	0.683	-0.047	0.009	0.006	0.018
**rs139887650**	C	T	0.988	-0.242	0.055	-0.092	0.099
**rs140150373**	T	A	0.987	-0.158	0.036	0.194	0.086
**rs140769414**	T	G	0.887	0.061	0.014	0.003	0.026
**rs1427633**	G	C	0.411	0.048	0.009	0.025	0.017
**rs143026942**	A	T	0.957	0.110	0.026	0.108	0.048
**rs1430894**	C	T	0.525	-0.053	0.009	-0.017	0.017
**rs143092720**	G	T	0.979	0.161	0.035	-0.104	0.068
**rs1444658**	G	A	0.531	-0.040	0.009	0.003	0.017
**rs144673133**	A	G	0.984	-0.148	0.034	-0.021	0.075
**rs145071536**	T	C	0.807	-0.085	0.012	0.017	0.025
**rs1451488**	A	G	0.447	-0.071	0.009	-0.030	0.017
**rs1452509**	A	G	0.763	-0.045	0.010	0.019	0.020
**rs1462358**	T	C	0.382	-0.043	0.009	0.029	0.017
**rs147169643**	C	T	0.976	-0.146	0.029	-0.031	0.061
**rs1482419**	A	G	0.237	0.052	0.010	-0.004	0.020
**rs1482962**	C	G	0.927	-0.078	0.019	-0.019	0.034
**rs149021237**	A	G	0.951	0.098	0.019	-0.046	0.039
**rs149165**	T	G	0.556	0.048	0.009	0.027	0.017
**rs1495099**	C	G	0.289	0.044	0.010	-0.074	0.019
**rs150751363**	C	G	0.972	-0.135	0.029	0.004	0.060
**rs1510136**	A	G	0.751	0.053	0.010	0.035	0.019
**rs1526803**	C	G	0.721	0.050	0.010	0.008	0.020
**rs1529073**	T	C	0.899	-0.066	0.014	-0.008	0.029
**rs1531167**	A	T	0.752	-0.046	0.010	-0.004	0.020
**rs1534464**	G	T	0.810	-0.054	0.011	-0.013	0.021
**rs1540840**	G	C	0.553	0.056	0.009	0.026	0.020
**rs1593304**	A	G	0.207	-0.064	0.011	0.008	0.022
**rs1604060**	A	G	0.103	-0.077	0.014	0.011	0.027
**rs1615350**	C	T	0.264	0.074	0.010	0.007	0.019
**rs167924**	A	G	0.360	-0.050	0.009	-0.063	0.018
**rs16851048**	T	C	0.799	-0.074	0.011	-0.015	0.021
**rs16867571**	A	G	0.768	0.066	0.010	-0.022	0.020
**rs1693523**	C	T	0.247	-0.045	0.010	-0.033	0.019
**rs16964949**	G	A	0.931	0.087	0.017	-0.042	0.033
**rs17016552**	C	G	0.645	0.052	0.009	0.008	0.018
**rs17119214**	G	C	0.808	0.045	0.011	0.011	0.021
**rs17128077**	C	T	0.860	-0.059	0.012	-0.016	0.024
**rs17194490**	G	T	0.838	-0.078	0.012	0.009	0.023
**rs17242605**	A	G	0.519	-0.040	0.009	0.023	0.017
**rs17384726**	A	G	0.975	-0.126	0.031	-0.084	0.068
**rs17394269**	A	G	0.523	-0.037	0.009	-0.026	0.017

**rs17574718**	C	G	0.936	-0.071	0.017	-0.015	0.037
**rs17598179**	G	A	0.956	-0.098	0.023	0.102	0.046
**rs176648**	T	G	0.478	0.042	0.009	0.026	0.017
**rs17671845**	T	C	0.960	-0.121	0.024	-0.069	0.044
**rs17731**	G	A	0.625	-0.052	0.009	-0.024	0.018
**rs17731604**	C	A	0.523	-0.043	0.009	0.004	0.017
**rs17769886**	T	G	0.860	0.052	0.013	0.052	0.025
**rs17864935**	T	C	0.564	-0.047	0.009	0.026	0.017
**rs1832899**	T	C	0.893	-0.060	0.014	-0.045	0.027
**rs1833193**	T	A	0.544	0.039	0.009	0.034	0.017
**rs1857472**	C	A	0.685	0.046	0.009	-0.002	0.018
**rs1862906**	G	A	0.551	0.047	0.009	0.006	0.018
**rs187557**	C	T	0.158	0.067	0.012	0.038	0.024
**rs1881046**	G	T	0.656	0.051	0.009	0.013	0.018
**rs188987899**	A	T	0.983	-0.183	0.044	-0.069	0.084
**rs1892346**	T	A	0.434	-0.048	0.009	-0.032	0.017
**rs1901512**	T	C	0.312	0.058	0.009	0.001	0.018
**rs1914399**	C	G	0.472	0.049	0.009	-0.003	0.017
**rs1915019**	A	G	0.259	0.057	0.010	0.001	0.019
**rs1953205**	T	A	0.515	-0.050	0.009	-0.020	0.018
**rs1953613**	A	G	0.372	0.045	0.009	-0.003	0.018
**rs1983488**	C	T	0.871	0.060	0.013	0.053	0.026
**rs1994224**	A	G	0.788	-0.055	0.010	-0.032	0.021
**rs2038255**	C	T	0.822	0.055	0.011	0.065	0.022
**rs2053079**	A	G	0.768	-0.060	0.010	-0.024	0.020
**rs207458**	C	T	0.198	-0.045	0.011	-0.026	0.021
**rs2078266**	A	G	0.179	0.070	0.013	0.003	0.028
**rs2121236**	T	C	0.718	-0.043	0.010	-0.017	0.019
**rs2147472**	G	A	0.608	0.042	0.009	-0.004	0.017
**rs2153960**	G	A	0.297	0.045	0.009	0.002	0.018
**rs215412**	G	A	0.677	-0.058	0.009	0.034	0.018
**rs217336**	C	A	0.581	0.050	0.009	-0.016	0.017
**rs2217023**	C	G	0.152	-0.050	0.012	-0.006	0.024
**rs2224086**	C	A	0.296	-0.056	0.010	0.011	0.021
**rs2236270**	G	T	0.608	-0.037	0.009	-0.010	0.017
**rs2238057**	T	G	0.590	-0.084	0.009	-0.031	0.017
**rs2251854**	G	A	0.323	0.043	0.009	-0.004	0.018
**rs2252074**	T	G	0.602	-0.069	0.009	-0.014	0.017
**rs2279311**	T	C	0.805	-0.051	0.011	0.014	0.021
**rs2287921**	T	C	0.495	-0.043	0.009	-0.037	0.017
**rs2297293**	C	G	0.317	0.046	0.009	0.031	0.018
**rs2298450**	C	T	0.638	0.039	0.009	0.047	0.018
**rs2314785**	T	C	0.814	0.056	0.011	0.017	0.022
**rs2325913**	C	T	0.444	-0.045	0.009	0.012	0.017
**rs2332700**	C	G	0.241	0.075	0.010	-0.024	0.020

**rs2333321**	A	G	0.200	0.071	0.011	0.007	0.021
**rs2347256**	A	G	0.525	-0.039	0.009	-0.031	0.017
**rs2381411**	T	C	0.599	-0.050	0.009	-0.024	0.018
**rs2387414**	G	C	0.394	-0.045	0.009	-0.006	0.019
**rs2401925**	A	G	0.370	0.036	0.009	0.001	0.017
**rs2417277**	A	G	0.650	0.042	0.009	0.007	0.018
**rs2420036**	T	C	0.787	0.043	0.011	-0.004	0.021
**rs2455415**	C	T	0.577	-0.049	0.009	0.004	0.017
**rs2456020**	C	T	0.765	0.082	0.010	-0.001	0.020
**rs2470180**	G	A	0.316	-0.040	0.010	0.000	0.019
**rs2514218**	C	T	0.643	0.070	0.009	0.012	0.018
**rs254778**	G	A	0.314	-0.040	0.009	-0.035	0.018
**rs2551445**	C	T	0.099	-0.071	0.014	-0.031	0.028
**rs2635663**	C	A	0.790	0.055	0.011	0.018	0.021
**rs2649999**	T	C	0.344	0.042	0.009	0.025	0.018
**rs2694031**	A	G	0.231	0.049	0.010	0.005	0.020
**rs2710323**	T	C	0.516	0.078	0.009	0.030	0.017
**rs2815731**	C	A	0.660	0.060	0.009	-0.019	0.018
**rs2825395**	T	G	0.737	-0.043	0.010	0.006	0.019
**rs28587597**	T	A	0.665	-0.047	0.009	0.026	0.018
**rs28696126**	A	T	0.141	0.070	0.013	-0.017	0.025
**rs2909457**	G	A	0.444	0.049	0.009	-0.011	0.017
**rs2910591**	T	C	0.064	-0.086	0.018	-0.041	0.035
**rs2938147**	A	G	0.577	-0.038	0.009	0.028	0.017
**rs2949814**	C	G	0.731	0.048	0.010	0.002	0.019
**rs2973043**	A	G	0.226	0.049	0.010	0.018	0.020
**rs2975923**	C	T	0.393	0.039	0.009	0.027	0.018
**rs2999392**	C	T	0.310	-0.052	0.009	-0.005	0.018
**rs3018001**	T	C	0.527	-0.043	0.009	0.004	0.017
**rs3026133**	G	A	0.506	0.043	0.009	0.020	0.017
**rs306206**	A	G	0.730	0.053	0.010	0.007	0.019
**rs308697**	C	A	0.556	0.050	0.009	0.006	0.017
**rs310653**	T	C	0.497	-0.040	0.009	-0.024	0.021
**rs322124**	C	G	0.795	-0.055	0.011	-0.022	0.021
**rs33996476**	A	G	0.891	-0.070	0.014	-0.001	0.028
**rs34103014**	C	T	0.945	0.089	0.020	-0.031	0.037
**rs34111680**	T	C	0.842	-0.049	0.012	0.020	0.023
**rs34275957**	A	G	0.746	-0.041	0.010	0.042	0.020
**rs34665905**	T	C	0.488	-0.038	0.009	0.016	0.017
**rs34768008**	A	T	0.902	0.076	0.015	-0.016	0.030
**rs34777351**	C	T	0.549	0.040	0.009	-0.024	0.017
**rs34888090**	G	A	0.837	-0.058	0.012	-0.051	0.023
**rs352766**	A	G	0.873	-0.060	0.013	-0.064	0.025
**rs35351411**	A	C	0.453	-0.064	0.009	-0.014	0.017
**rs35517955**	G	C	0.976	-0.224	0.051	0.063	0.074

**rs35564002**	C	T	0.985	-0.184	0.037	-0.004	0.074
**rs356183**	G	C	0.452	0.041	0.009	0.002	0.018
**rs35628191**	C	T	0.588	0.039	0.009	0.042	0.017
**rs35734242**	T	C	0.561	-0.051	0.009	0.007	0.018
**rs35780891**	G	A	0.830	-0.055	0.011	-0.017	0.022
**rs35981078**	G	A	0.803	0.055	0.011	0.023	0.022
**rs3739118**	G	A	0.713	0.057	0.010	0.012	0.019
**rs3752827**	T	A	0.691	0.047	0.009	-0.014	0.019
**rs3770754**	C	G	0.639	0.053	0.009	0.038	0.018
**rs3775182**	G	T	0.904	-0.068	0.015	0.036	0.028
**rs3781158**	C	T	0.652	0.047	0.009	0.024	0.018
**rs3788331**	C	T	0.557	-0.040	0.009	-0.008	0.017
**rs3791710**	T	C	0.796	0.060	0.011	-0.036	0.021
**rs3795310**	C	T	0.525	0.051	0.009	0.002	0.017
**rs3802924**	A	C	0.799	0.074	0.011	-0.010	0.021
**rs3812831**	T	C	0.471	-0.044	0.009	-0.002	0.017
**rs3814883**	C	T	0.523	0.067	0.009	0.011	0.017
**rs3824451**	T	C	0.842	-0.066	0.012	-0.043	0.023
**rs3845811**	C	G	0.574	0.038	0.009	0.002	0.018
**rs4129585**	A	C	0.441	0.075	0.009	-0.030	0.017
**rs41297816**	A	G	0.755	-0.051	0.010	-0.011	0.021
**rs41313730**	T	G	0.841	0.055	0.012	0.059	0.023
**rs41314284**	C	T	0.906	-0.081	0.015	0.028	0.029
**rs41475545**	T	C	0.925	-0.083	0.017	-0.021	0.033
**rs4237587**	T	G	0.234	-0.043	0.010	0.006	0.020
**rs4275631**	T	C	0.416	-0.038	0.009	0.012	0.017
**rs428602**	A	G	0.576	0.037	0.009	-0.019	0.017
**rs4308**	A	G	0.388	-0.044	0.009	-0.009	0.018
**rs4341665**	A	G	0.074	-0.090	0.018	-0.038	0.033
**rs4347627**	T	A	0.700	-0.040	0.009	-0.004	0.018
**rs4353292**	A	G	0.586	0.041	0.009	-0.002	0.017
**rs4378995**	G	A	0.524	0.041	0.009	-0.021	0.017
**rs4540690**	A	G	0.480	0.046	0.009	0.013	0.017
**rs4575535**	A	G	0.294	-0.056	0.010	-0.023	0.019
**rs4625037**	A	C	0.124	0.054	0.013	0.002	0.026
**rs4627212**	A	G	0.712	-0.051	0.010	-0.012	0.019
**rs4632195**	C	T	0.484	-0.047	0.009	0.038	0.017
**rs4636654**	G	A	0.593	0.048	0.009	-0.019	0.017
**rs4653164**	C	T	0.329	-0.051	0.009	0.012	0.018
**rs4668193**	G	A	0.386	-0.038	0.009	0.017	0.017
**rs4700418**	C	G	0.504	-0.070	0.009	0.021	0.017
**rs4702**	G	A	0.438	0.084	0.009	-0.033	0.018
**rs4721928**	G	A	0.845	-0.059	0.012	-0.009	0.025
**rs4739612**	C	T	0.489	0.037	0.009	-0.007	0.017
**rs4766428**	C	T	0.561	-0.075	0.009	0.006	0.018

**rs4779050**	T	G	0.370	0.058	0.009	0.012	0.018
**rs4797210**	C	T	0.984	-0.141	0.033	-0.065	0.069
**rs480211**	C	T	0.488	-0.045	0.009	-0.011	0.017
**rs4812325**	G	A	0.387	-0.072	0.009	-0.032	0.018
**rs4813190**	A	G	0.754	0.048	0.010	-0.011	0.020
**rs4837628**	T	C	0.570	0.036	0.009	0.023	0.017
**rs4904476**	T	G	0.739	0.045	0.010	0.011	0.020
**rs4921741**	A	G	0.739	-0.056	0.010	-0.023	0.019
**rs4925114**	A	G	0.368	0.044	0.009	-0.003	0.018
**rs4934962**	T	A	0.394	0.045	0.009	0.018	0.017
**rs4950076**	T	C	0.502	-0.044	0.009	0.018	0.017
**rs495839**	T	C	0.123	0.068	0.016	0.036	0.030
**rs4974598**	C	T	0.439	0.036	0.009	0.041	0.017
**rs498591**	A	T	0.850	-0.072	0.012	-0.015	0.024
**rs500102**	T	C	0.402	0.052	0.009	0.032	0.018
**rs505061**	C	A	0.499	-0.053	0.009	0.009	0.017
**rs508161**	A	C	0.651	-0.045	0.009	-0.015	0.019
**rs528598**	T	C	0.515	-0.044	0.009	0.008	0.017
**rs55648125**	A	G	0.898	-0.065	0.014	-0.009	0.028
**rs55700176**	T	C	0.665	-0.043	0.009	0.002	0.019
**rs55741542**	T	C	0.986	-0.172	0.036	-0.075	0.074
**rs56057437**	G	C	0.842	-0.055	0.012	0.017	0.025
**rs56205728**	G	A	0.710	-0.063	0.010	-0.028	0.020
**rs56286294**	C	T	0.599	0.038	0.009	-0.010	0.017
**rs56335113**	A	G	0.314	0.065	0.009	0.022	0.018
**rs57070985**	A	G	0.650	0.050	0.009	0.006	0.018
**rs57433322**	C	G	0.883	0.083	0.014	-0.017	0.027
**rs5751191**	T	C	0.503	-0.066	0.009	-0.022	0.017
**rs58120505**	T	C	0.576	0.090	0.009	0.030	0.017
**rs58763139**	A	G	0.959	-0.091	0.022	-0.023	0.043
**rs59682551**	T	G	0.765	0.054	0.011	0.146	0.021
**rs60135207**	G	T	0.581	0.050	0.009	0.023	0.017
**rs6037811**	G	A	0.343	-0.039	0.009	0.007	0.018
**rs6125656**	G	A	0.808	-0.064	0.011	0.005	0.022
**rs6132302**	T	C	0.755	-0.048	0.011	0.005	0.021
**rs61820761**	G	C	0.936	0.086	0.019	0.008	0.035
**rs61857878**	A	T	0.770	0.060	0.010	0.002	0.021
**rs61869029**	G	A	0.821	0.058	0.012	-0.029	0.026
**rs61901216**	A	G	0.823	0.048	0.011	0.006	0.022
**rs61920311**	A	C	0.582	0.048	0.009	0.043	0.018
**rs61937595**	C	T	0.913	0.130	0.016	0.077	0.034
**rs61973698**	C	T	0.775	-0.054	0.010	-0.016	0.020
**rs62018952**	T	C	0.283	-0.058	0.010	0.014	0.019
**rs62183855**	A	C	0.793	0.066	0.011	0.030	0.021
**rs62279133**	T	C	0.740	-0.040	0.010	-0.005	0.019

**rs62408211**	A	T	0.651	0.040	0.009	0.076	0.018
**rs62456353**	T	G	0.816	-0.048	0.011	-0.018	0.023
**rs634940**	G	T	0.748	-0.066	0.010	-0.004	0.020
**rs646234**	A	G	0.545	-0.041	0.009	0.002	0.017
**rs6478778**	G	T	0.903	-0.058	0.014	-0.037	0.029
**rs6482437**	A	C	0.104	-0.099	0.014	-0.015	0.028
**rs6499508**	A	G	0.863	0.069	0.013	0.025	0.024
**rs6520064**	A	G	0.792	-0.058	0.011	-0.063	0.021
**rs6538539**	G	T	0.447	0.057	0.009	0.006	0.017
**rs6545708**	T	A	0.550	0.042	0.009	0.009	0.017
**rs6546857**	A	G	0.766	-0.060	0.010	-0.011	0.020
**rs6549963**	T	C	0.585	0.048	0.009	0.012	0.017
**rs6656**	C	T	0.414	-0.044	0.009	-0.011	0.017
**rs6662**	G	T	0.195	-0.059	0.011	-0.001	0.021
**rs6673880**	A	G	0.500	-0.062	0.009	0.002	0.019
**rs66844263**	A	C	0.772	-0.042	0.010	-0.010	0.020
**rs6694677**	C	T	0.843	-0.051	0.012	-0.012	0.023
**rs6709782**	A	G	0.521	-0.036	0.009	-0.005	0.017
**rs6715366**	G	A	0.727	-0.054	0.010	0.005	0.019
**rs67300956**	C	T	0.891	0.059	0.014	0.004	0.027
**rs67627854**	T	G	0.783	-0.056	0.010	-0.026	0.020
**rs67715018**	A	G	0.769	-0.048	0.010	-0.019	0.020
**rs6790697**	C	T	0.965	0.089	0.020	-0.029	0.051
**rs6798742**	A	G	0.693	-0.061	0.009	0.000	0.018
**rs6825268**	A	G	0.525	-0.047	0.009	-0.027	0.018
**rs6851389**	C	T	0.500	0.041	0.009	-0.022	0.017
**rs6943762**	T	C	0.876	0.105	0.013	-0.020	0.026
**rs6974218**	A	C	0.628	0.055	0.009	0.003	0.017
**rs6982908**	C	T	0.262	-0.044	0.010	0.018	0.019
**rs6984242**	G	A	0.406	0.055	0.009	-0.001	0.017
**rs7002992**	T	C	0.633	0.045	0.009	0.000	0.018
**rs7022345**	A	G	0.176	-0.055	0.011	-0.039	0.022
**rs708228**	C	T	0.678	-0.053	0.009	0.036	0.018
**rs7096250**	C	A	0.222	-0.046	0.010	0.008	0.020
**rs710616**	T	A	0.494	0.046	0.009	0.011	0.017
**rs7112616**	T	C	0.510	0.052	0.009	0.011	0.017
**rs7116022**	T	C	0.350	-0.044	0.009	0.009	0.018
**rs7119089**	G	A	0.440	-0.038	0.009	-0.016	0.017
**rs713692**	G	A	0.315	-0.057	0.010	0.006	0.019
**rs7157278**	G	C	0.629	0.046	0.009	0.009	0.018
**rs71573419**	C	A	0.989	0.233	0.055	-0.130	0.105
**rs7162607**	G	A	0.557	-0.040	0.009	-0.032	0.017
**rs7203082**	G	A	0.760	-0.044	0.010	0.046	0.020
**rs7251**	C	G	0.664	0.064	0.009	-0.023	0.019
**rs72694418**	A	G	0.903	-0.072	0.015	-0.033	0.029

**rs72723227**	G	A	0.685	0.048	0.009	-0.025	0.019
**rs72743955**	A	G	0.986	0.169	0.039	0.133	0.083
**rs72802868**	G	T	0.708	0.069	0.010	0.009	0.018
**rs728055**	T	A	0.652	0.067	0.009	0.000	0.018
**rs72854425**	C	A	0.967	-0.112	0.026	-0.043	0.049
**rs72943392**	G	C	0.716	-0.053	0.010	-0.009	0.019
**rs72986630**	C	T	0.942	-0.112	0.018	0.047	0.043
**rs72997862**	A	C	0.958	0.120	0.026	0.030	0.053
**rs73038104**	C	T	0.697	0.040	0.010	-0.009	0.019
**rs73090180**	G	A	0.801	-0.057	0.011	-0.042	0.022
**rs7312697**	T	C	0.372	-0.049	0.009	0.023	0.017
**rs73172807**	G	A	0.849	0.055	0.012	0.014	0.024
**rs73200009**	T	C	0.850	-0.052	0.013	0.024	0.025
**rs73229090**	C	A	0.882	0.103	0.014	-0.027	0.027
**rs73292401**	T	A	0.805	-0.068	0.011	-0.007	0.021
**rs7333701**	G	C	0.933	-0.077	0.017	0.017	0.036
**rs7342307**	C	T	0.879	0.067	0.014	-0.040	0.028
**rs74335775**	C	T	0.975	-0.118	0.028	-0.024	0.059
**rs74379599**	G	A	0.936	0.084	0.019	0.044	0.035
**rs74700062**	C	T	0.962	-0.094	0.022	0.004	0.044
**rs75062856**	A	C	0.943	0.081	0.019	-0.037	0.038
**rs75072290**	T	C	0.831	-0.052	0.012	-0.026	0.025
**rs75072551**	T	C	0.983	-0.126	0.031	-0.091	0.066
**rs7515363**	C	T	0.384	0.054	0.009	-0.016	0.017
**rs75221401**	A	G	0.976	-0.136	0.033	-0.105	0.062
**rs7544605**	T	C	0.613	0.044	0.009	0.004	0.017
**rs755702**	A	G	0.633	-0.042	0.009	-0.017	0.018
**rs75677869**	A	G	0.902	-0.059	0.014	-0.037	0.028
**rs75690664**	T	C	0.943	-0.086	0.020	-0.031	0.041
**rs7575796**	A	G	0.929	0.096	0.017	-0.085	0.041
**rs75792959**	T	C	0.967	-0.119	0.027	-0.026	0.062
**rs76126732**	A	T	0.941	0.086	0.018	-0.010	0.037
**rs7618137**	T	C	0.709	-0.041	0.010	-0.019	0.019
**rs761840**	C	T	0.367	-0.039	0.009	0.018	0.018
**rs76190391**	A	T	0.878	0.057	0.014	0.021	0.026
**rs76243117**	A	G	0.886	-0.058	0.014	0.009	0.027
**rs7634476**	A	G	0.446	-0.058	0.009	-0.011	0.017
**rs76403337**	T	C	0.947	-0.079	0.019	0.066	0.038
**rs7641821**	T	C	0.367	-0.038	0.009	0.000	0.017
**rs7647398**	C	T	0.795	0.077	0.011	0.023	0.021
**rs76503615**	G	A	0.945	-0.096	0.018	-0.019	0.038
**rs7676578**	T	A	0.773	0.045	0.011	-0.029	0.020
**rs7679800**	T	C	0.717	-0.046	0.010	-0.010	0.019
**rs76831675**	T	C	0.980	-0.178	0.038	-0.024	0.075
**rs76838079**	C	T	0.851	-0.078	0.014	-0.009	0.028

**rs7684235**	T	C	0.492	0.045	0.009	0.026	0.017
**rs76852571**	C	G	0.910	0.065	0.015	0.002	0.030
**rs7699241**	C	T	0.909	-0.078	0.015	0.029	0.031
**rs77030891**	C	G	0.921	-0.069	0.016	0.024	0.033
**rs77181200**	C	A	0.969	0.108	0.025	0.023	0.052
**rs77206190**	C	T	0.974	-0.152	0.028	-0.017	0.057
**rs7735021**	A	G	0.178	-0.055	0.011	0.040	0.022
**rs77379825**	G	C	0.855	-0.062	0.012	-0.015	0.024
**rs7739151**	G	T	0.832	-0.056	0.012	-0.002	0.023
**rs7742212**	G	A	0.433	-0.041	0.009	0.005	0.017
**rs77442047**	G	A	0.954	-0.090	0.021	-0.005	0.041
**rs7768725**	T	G	0.865	-0.053	0.012	0.021	0.025
**rs77745530**	A	G	0.978	-0.138	0.028	-0.102	0.065
**rs7779018**	A	G	0.595	0.042	0.009	-0.007	0.019
**rs778371**	A	G	0.719	-0.081	0.010	-0.005	0.019
**rs77947333**	A	T	0.957	-0.106	0.022	-0.017	0.045
**rs7798283**	T	G	0.865	0.074	0.013	-0.011	0.025
**rs7799331**	A	G	0.832	0.054	0.012	-0.015	0.022
**rs7825426**	A	C	0.339	-0.038	0.009	-0.057	0.018
**rs7830315**	T	C	0.493	-0.048	0.009	-0.032	0.017
**rs7847153**	C	G	0.683	-0.051	0.010	-0.026	0.022
**rs78485958**	C	G	0.973	-0.128	0.029	-0.009	0.055
**rs7850322**	A	G	0.845	-0.061	0.012	-0.024	0.023
**rs7856690**	A	T	0.644	-0.047	0.009	-0.011	0.018
**rs7873149**	T	C	0.664	0.039	0.009	0.020	0.018
**rs7902292**	T	C	0.202	0.057	0.011	-0.018	0.022
**rs79073127**	G	C	0.882	0.073	0.014	-0.064	0.027
**rs7915131**	C	T	0.423	0.047	0.009	-0.068	0.017
**rs79169291**	G	T	0.986	-0.192	0.037	0.029	0.080
**rs79210963**	T	C	0.898	-0.086	0.014	-0.019	0.028
**rs79217743**	G	T	0.861	-0.056	0.013	0.014	0.026
**rs793248**	A	G	0.243	-0.042	0.010	0.010	0.020
**rs79415286**	A	G	0.905	0.066	0.014	-0.006	0.031
**rs79445414**	T	C	0.961	-0.123	0.022	-0.055	0.045
**rs79478621**	T	G	0.975	0.117	0.022	-0.100	0.060
**rs7952868**	A	G	0.244	-0.044	0.010	-0.032	0.020
**rs7953300**	G	T	0.386	-0.047	0.009	-0.022	0.017
**rs795587**	A	G	0.544	-0.041	0.009	0.012	0.017
**rs79784950**	G	T	0.754	-0.046	0.010	-0.011	0.019
**rs8007841**	T	C	0.337	0.039	0.009	-0.017	0.018
**rs8012618**	G	A	0.915	-0.067	0.015	-0.014	0.031
**rs8013990**	G	T	0.422	0.040	0.009	-0.028	0.017
**rs8026363**	G	A	0.786	0.054	0.011	0.023	0.022
**rs8055219**	G	A	0.765	-0.067	0.010	-0.008	0.020
**rs8070040**	T	C	0.364	0.041	0.009	-0.001	0.018

**rs8112050**	C	T	0.299	0.048	0.009	0.007	0.018
**rs8119702**	A	T	0.901	-0.058	0.014	-0.028	0.030
**rs834201**	A	G	0.396	-0.042	0.009	-0.028	0.018
**rs862989**	A	G	0.782	-0.042	0.010	-0.008	0.020
**rs886076**	T	C	0.611	-0.037	0.009	-0.011	0.018
**rs891645**	T	A	0.706	0.045	0.010	-0.006	0.019
**rs9304548**	C	A	0.248	0.057	0.010	0.003	0.020
**rs9318627**	A	C	0.597	0.061	0.009	-0.015	0.017
**rs9396665**	A	G	0.876	-0.065	0.013	-0.013	0.027
**rs9403484**	C	T	0.776	-0.053	0.010	-0.007	0.020
**rs9410573**	T	C	0.595	0.040	0.009	-0.007	0.018
**rs9461916**	T	C	0.397	-0.053	0.009	-0.011	0.017
**rs9470670**	T	G	0.830	-0.062	0.011	0.034	0.023
**rs9484329**	A	G	0.880	0.068	0.013	-0.015	0.026
**rs9487653**	A	G	0.226	0.056	0.011	0.032	0.020
**rs949458**	T	C	0.605	-0.036	0.009	-0.016	0.018
**rs950885**	T	C	0.893	0.062	0.014	-0.014	0.027
**rs9554348**	T	A	0.848	0.061	0.013	0.021	0.024
**rs9566131**	G	T	0.838	-0.050	0.012	-0.007	0.023
**rs9605070**	C	T	0.769	-0.048	0.010	-0.014	0.020
**rs9630925**	C	A	0.904	0.065	0.015	0.009	0.028
**rs9636107**	A	G	0.519	-0.070	0.009	-0.011	0.017
**rs9673542**	C	T	0.940	-0.084	0.018	-0.067	0.035
**rs9674069**	A	G	0.217	0.053	0.010	0.008	0.021
**rs9687282**	T	G	0.658	-0.053	0.009	0.004	0.018
**rs979618**	C	T	0.591	0.045	0.009	0.004	0.017
**rs984032**	A	T	0.536	-0.039	0.009	-0.031	0.017
**rs9843908**	T	C	0.448	0.040	0.009	-0.006	0.017
**rs987484**	A	T	0.546	0.037	0.009	-0.043	0.017
**rs9876421**	C	T	0.660	-0.063	0.009	-0.034	0.018
**rs9882339**	A	G	0.956	-0.088	0.021	0.043	0.042
**rs9902772**	T	C	0.920	0.070	0.017	0.049	0.032
**rs9971729**	A	C	0.437	-0.037	0.009	-0.018	0.017
**rs9975024**	A	G	0.535	-0.044	0.009	0.019	0.017
**rs9986069**	T	A	0.684	0.040	0.010	0.000	0.019
**rs9989481**	T	C	0.435	-0.042	0.009	0.014	0.019
**rs999494**	C	T	0.802	0.056	0.011	-0.011	0.021

Table S58. Raw data for the MR analysis of the causal effect of schizophrenia on CD (P<5E-5)							
SNP	Effect allele	Other allele	Effect allele frequency	Beta.exposure	Se.exposure	Beta.outcome	Se.outcome
rs1000237	T	A	0.638	-0.073	0.009	-0.030	0.024
rs10005436	G	A	0.666	-0.040	0.009	-0.033	0.024
rs10035564	A	G	0.654	-0.067	0.009	-0.006	0.024
rs10069930	T	A	0.484	0.045	0.009	-0.007	0.024
rs10086619	A	G	0.843	-0.072	0.012	-0.076	0.031
rs10100465	G	A	0.785	0.043	0.011	0.031	0.028
rs10108980	C	T	0.807	-0.063	0.011	0.003	0.029
rs10117	G	A	0.591	0.055	0.009	-0.034	0.023
rs10133619	T	G	0.605	0.044	0.009	0.022	0.023
rs10148671	T	C	0.341	-0.047	0.009	0.006	0.024
rs10173857	C	T	0.715	0.051	0.010	-0.003	0.026
rs10238960	C	T	0.287	-0.039	0.010	-0.019	0.025
rs10520163	T	C	0.494	0.047	0.009	0.001	0.023
rs10751832	A	G	0.143	0.057	0.013	0.034	0.033
rs10760502	A	G	0.358	-0.041	0.010	0.063	0.025
rs10821976	G	A	0.749	0.046	0.011	0.003	0.029
rs10832722	C	T	0.704	0.049	0.010	-0.003	0.026
rs10861176	G	A	0.272	-0.056	0.010	-0.009	0.026
rs10873538	T	G	0.681	-0.067	0.009	-0.017	0.025
rs10876446	G	C	0.684	-0.054	0.009	-0.010	0.026
rs10931083	T	C	0.051	0.089	0.022	0.001	0.057
rs10935477	G	T	0.759	-0.043	0.010	0.073	0.027
rs10999895	C	T	0.610	-0.041	0.009	0.031	0.025
rs11027839	A	C	0.502	-0.052	0.009	0.017	0.023
rs11061973	G	A	0.847	0.055	0.012	0.001	0.032
rs11064676	C	G	0.792	0.045	0.011	-0.013	0.029
rs1106478	T	G	0.794	-0.056	0.011	0.000	0.028
rs11105285	C	T	0.784	0.057	0.011	0.024	0.029
rs11129511	G	A	0.664	0.039	0.009	0.041	0.025
rs111320077	C	T	0.896	-0.063	0.014	-0.062	0.037
rs11136325	G	A	0.427	0.054	0.009	-0.053	0.026
rs11165690	T	C	0.812	-0.054	0.011	0.031	0.029
rs11165867	C	T	0.837	-0.074	0.012	-0.001	0.031
rs11191580	T	C	0.914	0.132	0.016	0.015	0.040
rs1120004	T	G	0.253	0.047	0.010	0.022	0.026
rs11210892	G	A	0.330	0.064	0.009	0.033	0.024
rs11217602	C	T	0.588	0.037	0.009	-0.040	0.024
rs112177478	C	T	0.913	0.082	0.015	0.065	0.041
rs11223774	A	G	0.295	0.052	0.009	-0.014	0.025
rs11251858	C	T	0.642	-0.039	0.009	-0.015	0.024
rs11252007	C	T	0.740	-0.046	0.010	0.002	0.028
rs113264400	T	C	0.954	-0.112	0.020	-0.049	0.060

**rs113892895**	A	T	0.946	0.093	0.019	0.013	0.052
**rs114702133**	A	G	0.957	-0.101	0.023	0.010	0.061
**rs11487432**	A	G	0.914	0.062	0.015	0.000	0.041
**rs11534045**	G	A	0.663	0.063	0.009	0.026	0.024
**rs115361459**	G	A	0.974	0.136	0.030	0.036	0.079
**rs11587347**	C	G	0.897	-0.104	0.015	0.013	0.039
**rs11590169**	A	G	0.915	-0.067	0.016	-0.082	0.041
**rs11632125**	G	A	0.966	-0.118	0.026	-0.006	0.077
**rs116350522**	G	T	0.961	-0.102	0.023	0.068	0.068
**rs11647188**	A	G	0.604	0.048	0.009	0.007	0.024
**rs11663962**	G	A	0.724	-0.043	0.010	0.039	0.026
**rs11664298**	G	A	0.805	-0.077	0.011	0.052	0.029
**rs11668896**	T	C	0.357	-0.048	0.010	0.021	0.028
**rs11680328**	T	A	0.128	0.064	0.013	0.012	0.035
**rs11686852**	G	A	0.548	0.046	0.009	0.010	0.023
**rs11687946**	G	T	0.590	0.043	0.009	-0.020	0.024
**rs11690184**	T	C	0.489	0.036	0.009	0.016	0.024
**rs11693094**	C	T	0.553	0.054	0.009	0.019	0.023
**rs116966156**	C	T	0.984	-0.137	0.033	-0.124	0.097
**rs117170626**	G	A	0.970	-0.110	0.026	0.024	0.083
**rs117178087**	C	T	0.927	0.096	0.018	-0.070	0.044
**rs11719324**	A	C	0.579	-0.038	0.009	-0.021	0.023
**rs11732321**	C	G	0.565	-0.040	0.009	-0.026	0.023
**rs11740474**	A	T	0.592	-0.054	0.009	0.021	0.024
**rs11752044**	G	A	0.781	0.055	0.011	0.011	0.028
**rs11780451**	T	G	0.083	-0.080	0.016	-0.019	0.042
**rs118049780**	G	A	0.918	-0.074	0.018	0.008	0.045
**rs11811826**	T	A	0.772	-0.045	0.010	0.038	0.028
**rs118121724**	C	T	0.929	-0.073	0.017	0.070	0.051
**rs11854073**	G	A	0.682	0.047	0.009	0.003	0.025
**rs11878234**	A	C	0.873	0.058	0.014	-0.002	0.037
**rs11941714**	G	A	0.665	0.052	0.009	-0.027	0.026
**rs11964401**	A	G	0.489	0.045	0.009	0.006	0.023
**rs11972718**	C	G	0.696	-0.048	0.009	-0.046	0.025
**rs1198588**	A	T	0.196	-0.103	0.011	-0.033	0.029
**rs12029721**	A	G	0.920	-0.081	0.018	0.012	0.051
**rs1203630**	A	G	0.261	0.043	0.010	0.016	0.026
**rs12040431**	C	T	0.334	0.040	0.009	-0.042	0.025
**rs12120481**	A	G	0.856	-0.058	0.013	0.081	0.034
**rs12121218**	T	G	0.815	-0.049	0.011	-0.041	0.029
**rs12129573**	C	A	0.618	-0.078	0.009	0.012	0.024
**rs12138061**	C	A	0.794	-0.045	0.010	0.036	0.028
**rs12138231**	T	A	0.186	-0.067	0.012	0.033	0.030
**rs12142659**	T	C	0.697	-0.045	0.010	-0.020	0.026
**rs12145726**	G	A	0.372	0.046	0.009	-0.015	0.024

**rs12151767**	G	A	0.511	0.061	0.009	0.093	0.023
**rs12153880**	G	A	0.807	0.052	0.011	0.021	0.030
**rs1217091**	T	C	0.188	-0.059	0.011	-0.018	0.029
**rs12186968**	C	T	0.704	0.047	0.010	0.042	0.026
**rs12190176**	A	G	0.637	-0.038	0.009	-0.001	0.024
**rs12285419**	C	A	0.813	-0.085	0.011	-0.044	0.030
**rs12293670**	A	G	0.665	0.070	0.009	0.011	0.025
**rs12303743**	G	C	0.903	-0.087	0.015	0.049	0.039
**rs12462062**	C	G	0.703	-0.040	0.010	0.031	0.025
**rs12474906**	A	C	0.798	0.056	0.011	0.043	0.029
**rs12485775**	G	A	0.800	0.047	0.011	-0.020	0.028
**rs12489270**	T	C	0.621	-0.058	0.009	-0.013	0.024
**rs12499194**	C	T	0.855	-0.056	0.013	0.024	0.036
**rs1253434**	T	C	0.674	0.043	0.009	0.024	0.025
**rs12577324**	T	C	0.792	0.046	0.011	0.015	0.031
**rs12600720**	C	G	0.680	-0.048	0.010	-0.057	0.027
**rs12615991**	A	G	0.856	-0.055	0.012	-0.036	0.033
**rs12624433**	G	A	0.737	-0.044	0.010	-0.057	0.026
**rs12652777**	T	C	0.471	0.049	0.009	-0.019	0.023
**rs12671608**	T	C	0.880	-0.064	0.013	0.021	0.040
**rs12712510**	T	C	0.467	0.057	0.009	0.021	0.023
**rs12771371**	G	A	0.694	0.052	0.009	-0.032	0.025
**rs12833624**	C	T	0.647	-0.050	0.009	0.042	0.024
**rs12877581**	G	C	0.732	-0.060	0.010	-0.033	0.028
**rs12883788**	C	T	0.568	-0.061	0.009	-0.006	0.025
**rs1289003**	G	T	0.583	-0.048	0.009	0.003	0.023
**rs12943566**	A	G	0.341	-0.050	0.009	-0.004	0.024
**rs13011472**	C	G	0.509	-0.070	0.009	0.006	0.023
**rs13014947**	G	A	0.440	0.039	0.009	0.045	0.023
**rs13016542**	T	C	0.871	0.088	0.013	0.024	0.036
**rs13025903**	T	A	0.778	-0.051	0.010	0.001	0.028
**rs13030312**	A	T	0.874	-0.057	0.013	0.015	0.035
**rs13074132**	T	C	0.644	0.047	0.009	-0.014	0.025
**rs13090130**	G	A	0.619	0.041	0.009	0.024	0.023
**rs13107325**	C	T	0.911	-0.159	0.017	-0.222	0.040
**rs13145146**	A	G	0.950	-0.090	0.019	-0.017	0.053
**rs13150491**	C	T	0.552	-0.037	0.009	-0.002	0.023
**rs13154281**	C	G	0.554	0.039	0.009	-0.019	0.023
**rs13177597**	C	G	0.876	-0.056	0.014	0.026	0.036
**rs1319017**	G	A	0.656	-0.046	0.009	0.001	0.024
**rs13195636**	A	C	0.918	0.211	0.016	0.086	0.043
**rs13233308**	C	T	0.525	0.049	0.009	0.016	0.023
**rs132582**	C	T	0.458	0.051	0.009	0.011	0.023
**rs13273892**	C	A	0.674	-0.040	0.009	-0.006	0.024
**rs13320945**	C	T	0.605	-0.045	0.009	0.001	0.023

**rs13396659**	A	C	0.799	-0.046	0.011	-0.005	0.029
**rs1389718**	G	A	0.770	-0.044	0.010	-0.077	0.027
**rs1392414**	A	G	0.186	0.047	0.011	0.020	0.030
**rs1398541**	G	A	0.679	-0.047	0.009	-0.030	0.025
**rs139887650**	C	T	0.989	-0.242	0.055	-0.080	0.150
**rs140150373**	T	A	0.987	-0.158	0.036	0.203	0.116
**rs140769414**	T	G	0.888	0.061	0.014	0.077	0.036
**rs1427633**	G	C	0.410	0.048	0.009	0.023	0.024
**rs143026942**	A	T	0.954	0.110	0.026	0.040	0.064
**rs1430894**	C	T	0.526	-0.053	0.009	-0.029	0.023
**rs143092720**	G	T	0.979	0.161	0.035	-0.125	0.092
**rs1444658**	G	A	0.527	-0.040	0.009	0.004	0.023
**rs144673133**	A	G	0.985	-0.148	0.034	-0.020	0.108
**rs145071536**	T	C	0.811	-0.085	0.012	0.038	0.035
**rs1451488**	A	G	0.450	-0.071	0.009	-0.028	0.023
**rs1452509**	A	G	0.763	-0.045	0.010	0.046	0.027
**rs1462358**	T	C	0.377	-0.043	0.009	0.009	0.024
**rs147169643**	C	T	0.976	-0.146	0.029	-0.057	0.084
**rs1482419**	A	G	0.235	0.052	0.010	0.001	0.027
**rs1482962**	C	G	0.924	-0.078	0.019	-0.037	0.046
**rs149021237**	A	G	0.952	0.098	0.019	-0.013	0.055
**rs149165**	T	G	0.553	0.048	0.009	0.016	0.023
**rs1495099**	C	G	0.294	0.044	0.010	-0.064	0.025
**rs150751363**	C	G	0.971	-0.135	0.029	0.091	0.079
**rs1510136**	A	G	0.747	0.053	0.010	0.035	0.026
**rs1526803**	C	G	0.717	0.050	0.010	-0.003	0.026
**rs1529073**	T	C	0.897	-0.066	0.014	0.001	0.039
**rs1531167**	A	T	0.744	-0.046	0.010	0.023	0.027
**rs1534464**	G	T	0.814	-0.054	0.011	0.025	0.030
**rs1540840**	G	C	0.557	0.056	0.009	0.047	0.027
**rs1593304**	A	G	0.214	-0.064	0.011	0.011	0.029
**rs1604060**	A	G	0.099	-0.077	0.014	0.004	0.038
**rs1615350**	C	T	0.267	0.074	0.010	0.024	0.026
**rs167924**	A	G	0.361	-0.050	0.009	-0.064	0.024
**rs16851048**	T	C	0.796	-0.074	0.011	-0.004	0.028
**rs16867571**	A	G	0.770	0.066	0.010	-0.034	0.028
**rs1693523**	C	T	0.251	-0.045	0.010	-0.042	0.026
**rs16964949**	G	A	0.933	0.087	0.017	-0.061	0.045
**rs17016552**	C	G	0.640	0.052	0.009	-0.012	0.024
**rs17119214**	G	C	0.810	0.045	0.011	0.039	0.029
**rs17128077**	C	T	0.861	-0.059	0.012	-0.007	0.033
**rs17194490**	G	T	0.838	-0.078	0.012	0.017	0.032
**rs17242605**	A	G	0.522	-0.040	0.009	0.020	0.023
**rs17384726**	A	G	0.976	-0.126	0.031	-0.157	0.092
**rs17394269**	A	G	0.527	-0.037	0.009	-0.017	0.023

**rs17574718**	C	G	0.937	-0.071	0.017	-0.012	0.051
**rs17598179**	G	A	0.955	-0.098	0.023	0.061	0.063
**rs176648**	T	G	0.475	0.042	0.009	0.036	0.023
**rs17671845**	T	C	0.959	-0.121	0.024	0.042	0.059
**rs17731**	G	A	0.625	-0.052	0.009	-0.043	0.024
**rs17731604**	C	A	0.518	-0.043	0.009	0.016	0.023
**rs17769886**	T	G	0.857	0.052	0.013	0.049	0.033
**rs17864935**	T	C	0.562	-0.047	0.009	0.029	0.023
**rs1832899**	T	C	0.894	-0.060	0.014	-0.046	0.037
**rs1833193**	T	A	0.544	0.039	0.009	0.030	0.024
**rs1857472**	C	A	0.683	0.046	0.009	-0.013	0.025
**rs1862906**	G	A	0.551	0.047	0.009	-0.002	0.024
**rs187557**	C	T	0.159	0.067	0.012	0.020	0.032
**rs1881046**	G	T	0.655	0.051	0.009	0.008	0.024
**rs188987899**	A	T	0.984	-0.183	0.044	0.136	0.121
**rs1892346**	T	A	0.431	-0.048	0.009	-0.046	0.024
**rs1901512**	T	C	0.315	0.058	0.009	-0.013	0.025
**rs1914399**	C	G	0.470	0.049	0.009	0.006	0.023
**rs1915019**	A	G	0.263	0.057	0.010	-0.007	0.026
**rs1953205**	T	A	0.516	-0.050	0.009	-0.023	0.025
**rs1953613**	A	G	0.364	0.045	0.009	0.003	0.024
**rs1983488**	C	T	0.871	0.060	0.013	0.094	0.035
**rs1994224**	A	G	0.789	-0.055	0.010	-0.066	0.028
**rs2038255**	C	T	0.822	0.055	0.011	0.075	0.031
**rs2053079**	A	G	0.772	-0.060	0.010	-0.018	0.027
**rs207458**	C	T	0.201	-0.045	0.011	-0.016	0.029
**rs2078266**	A	G	0.165	0.070	0.013	0.044	0.041
**rs2121236**	T	C	0.714	-0.043	0.010	-0.014	0.025
**rs2147472**	G	A	0.606	0.042	0.009	-0.016	0.024
**rs2153960**	G	A	0.298	0.045	0.009	0.004	0.025
**rs215412**	G	A	0.683	-0.058	0.009	0.048	0.025
**rs217336**	C	A	0.587	0.050	0.009	-0.006	0.023
**rs2217023**	C	G	0.153	-0.050	0.012	0.013	0.032
**rs2224086**	C	A	0.296	-0.056	0.010	-0.017	0.028
**rs2236270**	G	T	0.609	-0.037	0.009	0.013	0.023
**rs2238057**	T	G	0.590	-0.084	0.009	-0.023	0.023
**rs2251854**	G	A	0.323	0.043	0.009	-0.014	0.024
**rs2252074**	T	G	0.602	-0.069	0.009	-0.005	0.023
**rs2279311**	T	C	0.800	-0.051	0.011	-0.003	0.029
**rs2287921**	T	C	0.495	-0.043	0.009	-0.088	0.024
**rs2297293**	C	G	0.315	0.046	0.009	0.028	0.025
**rs2298450**	C	T	0.643	0.039	0.009	0.055	0.024
**rs2314785**	T	C	0.813	0.056	0.011	0.016	0.029
**rs2325913**	C	T	0.443	-0.045	0.009	0.024	0.023
**rs2332700**	C	G	0.240	0.075	0.010	-0.049	0.027

**rs2333321**	A	G	0.195	0.071	0.011	-0.015	0.029
**rs2347256**	A	G	0.522	-0.039	0.009	-0.023	0.023
**rs2381411**	T	C	0.598	-0.050	0.009	-0.031	0.024
**rs2387414**	G	C	0.389	-0.045	0.009	-0.010	0.025
**rs2401925**	A	G	0.367	0.036	0.009	0.020	0.024
**rs2417277**	A	G	0.651	0.042	0.009	0.006	0.024
**rs2420036**	T	C	0.789	0.043	0.011	0.001	0.029
**rs2455415**	C	T	0.577	-0.049	0.009	0.025	0.024
**rs2456020**	C	T	0.765	0.082	0.010	0.003	0.027
**rs2470180**	G	A	0.314	-0.040	0.010	0.022	0.025
**rs2514218**	C	T	0.633	0.070	0.009	-0.004	0.024
**rs254778**	G	A	0.316	-0.040	0.009	-0.063	0.025
**rs2551445**	C	T	0.097	-0.071	0.014	-0.021	0.039
**rs2635663**	C	A	0.791	0.055	0.011	-0.021	0.029
**rs2649999**	T	C	0.343	0.042	0.009	0.015	0.025
**rs2694031**	A	G	0.227	0.049	0.010	0.002	0.027
**rs2710323**	T	C	0.515	0.078	0.009	0.018	0.023
**rs2815731**	C	A	0.667	0.060	0.009	-0.013	0.024
**rs2825395**	T	G	0.734	-0.043	0.010	0.013	0.026
**rs28587597**	T	A	0.669	-0.047	0.009	0.000	0.024
**rs28696126**	A	T	0.144	0.070	0.013	-0.018	0.034
**rs2909457**	G	A	0.444	0.049	0.009	0.003	0.023
**rs2910591**	T	C	0.062	-0.086	0.018	-0.037	0.049
**rs2938147**	A	G	0.573	-0.038	0.009	0.019	0.023
**rs2949814**	C	G	0.733	0.048	0.010	0.016	0.026
**rs2973043**	A	G	0.227	0.049	0.010	0.008	0.027
**rs2975923**	C	T	0.395	0.039	0.009	0.032	0.024
**rs2999392**	C	T	0.313	-0.052	0.009	-0.013	0.025
**rs3018001**	T	C	0.525	-0.043	0.009	0.002	0.024
**rs3026133**	G	A	0.510	0.043	0.009	0.022	0.023
**rs306206**	A	G	0.728	0.053	0.010	-0.007	0.026
**rs308697**	C	A	0.556	0.050	0.009	0.030	0.023
**rs310653**	T	C	0.490	-0.040	0.009	-0.038	0.029
**rs322124**	C	G	0.795	-0.055	0.011	0.001	0.029
**rs33996476**	A	G	0.893	-0.070	0.014	-0.002	0.038
**rs34103014**	C	T	0.946	0.089	0.020	-0.033	0.050
**rs34111680**	T	C	0.842	-0.049	0.012	-0.016	0.031
**rs34275957**	A	G	0.743	-0.041	0.010	0.076	0.028
**rs34665905**	T	C	0.489	-0.038	0.009	0.026	0.023
**rs34768008**	A	T	0.901	0.076	0.015	0.006	0.040
**rs34777351**	C	T	0.549	0.040	0.009	-0.022	0.023
**rs34888090**	G	A	0.838	-0.058	0.012	-0.081	0.031
**rs352766**	A	G	0.872	-0.060	0.013	-0.065	0.034
**rs35351411**	A	C	0.452	-0.064	0.009	-0.011	0.023
**rs35517955**	G	C	0.977	-0.224	0.051	0.008	0.104

**rs35564002**	C	T	0.985	-0.184	0.037	0.001	0.100
**rs356183**	G	C	0.451	0.041	0.009	-0.037	0.024
**rs35628191**	C	T	0.588	0.039	0.009	0.012	0.023
**rs35734242**	T	C	0.559	-0.051	0.009	0.044	0.025
**rs35780891**	G	A	0.831	-0.055	0.011	0.006	0.031
**rs35981078**	G	A	0.806	0.055	0.011	0.018	0.030
**rs3739118**	G	A	0.712	0.057	0.010	0.013	0.025
**rs3752827**	T	A	0.692	0.047	0.009	-0.006	0.026
**rs3770754**	C	G	0.636	0.053	0.009	-0.006	0.024
**rs3775182**	G	T	0.904	-0.068	0.015	-0.003	0.038
**rs3781158**	C	T	0.649	0.047	0.009	0.062	0.024
**rs3788331**	C	T	0.563	-0.040	0.009	0.003	0.023
**rs3791710**	T	C	0.796	0.060	0.011	-0.034	0.029
**rs3795310**	C	T	0.519	0.051	0.009	0.057	0.023
**rs3802924**	A	C	0.807	0.074	0.011	-0.051	0.029
**rs3812831**	T	C	0.471	-0.044	0.009	-0.008	0.024
**rs3814883**	C	T	0.525	0.067	0.009	0.019	0.024
**rs3824451**	T	C	0.841	-0.066	0.012	-0.068	0.031
**rs3845811**	C	G	0.567	0.038	0.009	-0.014	0.024
**rs4129585**	A	C	0.442	0.075	0.009	-0.036	0.023
**rs41297816**	A	G	0.752	-0.051	0.010	0.019	0.029
**rs41313730**	T	G	0.842	0.055	0.012	0.056	0.032
**rs41314284**	C	T	0.903	-0.081	0.015	0.071	0.040
**rs41475545**	T	C	0.921	-0.083	0.017	0.004	0.044
**rs4237587**	T	G	0.232	-0.043	0.010	0.013	0.027
**rs4275631**	T	C	0.414	-0.038	0.009	0.045	0.024
**rs428602**	A	G	0.576	0.037	0.009	-0.036	0.023
**rs4308**	A	G	0.391	-0.044	0.009	-0.018	0.024
**rs4341665**	A	G	0.077	-0.090	0.018	-0.015	0.044
**rs4347627**	T	A	0.698	-0.040	0.009	0.001	0.025
**rs4353292**	A	G	0.589	0.041	0.009	0.019	0.023
**rs4378995**	G	A	0.526	0.041	0.009	-0.020	0.023
**rs4540690**	A	G	0.478	0.046	0.009	-0.003	0.023
**rs4575535**	A	G	0.288	-0.056	0.010	-0.047	0.026
**rs4625037**	A	C	0.127	0.054	0.013	0.045	0.034
**rs4627212**	A	G	0.713	-0.051	0.010	-0.024	0.025
**rs4632195**	C	T	0.483	-0.047	0.009	0.045	0.023
**rs4636654**	G	A	0.591	0.048	0.009	-0.013	0.023
**rs4653164**	C	T	0.331	-0.051	0.009	0.012	0.024
**rs4668193**	G	A	0.382	-0.038	0.009	0.023	0.024
**rs4700418**	C	G	0.502	-0.070	0.009	0.000	0.023
**rs4702**	G	A	0.443	0.084	0.009	-0.035	0.024
**rs4721928**	G	A	0.844	-0.059	0.012	-0.009	0.034
**rs4739612**	C	T	0.490	0.037	0.009	-0.036	0.023
**rs4766428**	C	T	0.566	-0.075	0.009	0.033	0.024

**rs4779050**	T	G	0.369	0.058	0.009	0.043	0.024
**rs4797210**	C	T	0.984	-0.141	0.033	-0.070	0.091
**rs480211**	C	T	0.486	-0.045	0.009	-0.014	0.023
**rs4812325**	G	A	0.391	-0.072	0.009	-0.005	0.024
**rs4813190**	A	G	0.755	0.048	0.010	-0.022	0.027
**rs4837628**	T	C	0.571	0.036	0.009	0.020	0.023
**rs4904476**	T	G	0.744	0.045	0.010	0.034	0.028
**rs4921741**	A	G	0.734	-0.056	0.010	-0.021	0.026
**rs4925114**	A	G	0.371	0.044	0.009	0.009	0.024
**rs4934962**	T	A	0.394	0.045	0.009	0.024	0.023
**rs4950076**	T	C	0.506	-0.044	0.009	0.013	0.023
**rs495839**	T	C	0.116	0.068	0.016	0.029	0.041
**rs4974598**	C	T	0.442	0.036	0.009	0.036	0.024
**rs498591**	A	T	0.849	-0.072	0.012	0.009	0.032
**rs500102**	T	C	0.394	0.052	0.009	0.024	0.024
**rs505061**	C	A	0.495	-0.053	0.009	-0.041	0.023
**rs508161**	A	C	0.652	-0.045	0.009	-0.008	0.026
**rs528598**	T	C	0.514	-0.044	0.009	0.030	0.023
**rs55648125**	A	G	0.899	-0.065	0.014	0.021	0.039
**rs55700176**	T	C	0.667	-0.043	0.009	0.001	0.025
**rs55741542**	T	C	0.986	-0.172	0.036	-0.114	0.100
**rs56057437**	G	C	0.843	-0.055	0.012	0.019	0.036
**rs56205728**	G	A	0.712	-0.063	0.010	-0.049	0.027
**rs56286294**	C	T	0.602	0.038	0.009	0.015	0.024
**rs56335113**	A	G	0.309	0.065	0.009	0.044	0.025
**rs57070985**	A	G	0.648	0.050	0.009	0.005	0.025
**rs57433322**	C	G	0.884	0.083	0.014	-0.019	0.037
**rs5751191**	T	C	0.506	-0.066	0.009	-0.031	0.023
**rs58120505**	T	C	0.574	0.090	0.009	0.057	0.023
**rs58763139**	A	G	0.958	-0.091	0.022	-0.070	0.056
**rs59682551**	T	G	0.766	0.054	0.011	0.127	0.028
**rs60135207**	G	T	0.575	0.050	0.009	0.012	0.023
**rs6037811**	G	A	0.345	-0.039	0.009	0.021	0.024
**rs6125656**	G	A	0.806	-0.064	0.011	0.042	0.029
**rs6132302**	T	C	0.755	-0.048	0.011	0.039	0.029
**rs61820761**	G	C	0.937	0.086	0.019	-0.007	0.048
**rs61857878**	A	T	0.773	0.060	0.010	-0.041	0.029
**rs61869029**	G	A	0.827	0.058	0.012	-0.070	0.037
**rs61901216**	A	G	0.827	0.048	0.011	0.011	0.030
**rs61920311**	A	C	0.580	0.048	0.009	0.057	0.025
**rs61937595**	C	T	0.915	0.130	0.016	0.022	0.047
**rs61973698**	C	T	0.775	-0.054	0.010	0.001	0.028
**rs62018952**	T	C	0.289	-0.058	0.010	0.060	0.025
**rs62183855**	A	C	0.790	0.066	0.011	0.005	0.029
**rs62279133**	T	C	0.748	-0.040	0.010	0.002	0.027

**rs62408211**	A	T	0.649	0.040	0.009	0.099	0.024
**rs62456353**	T	G	0.820	-0.048	0.011	0.006	0.032
**rs634940**	G	T	0.750	-0.066	0.010	0.012	0.027
**rs646234**	A	G	0.544	-0.041	0.009	0.018	0.023
**rs6478778**	G	T	0.901	-0.058	0.014	0.013	0.039
**rs6482437**	A	C	0.104	-0.099	0.014	0.001	0.038
**rs6499508**	A	G	0.863	0.069	0.013	0.008	0.033
**rs6520064**	A	G	0.790	-0.058	0.011	-0.074	0.028
**rs6538539**	G	T	0.449	0.057	0.009	0.015	0.023
**rs6545708**	T	A	0.549	0.042	0.009	-0.004	0.024
**rs6546857**	A	G	0.769	-0.060	0.010	-0.015	0.027
**rs6549963**	T	C	0.584	0.048	0.009	0.010	0.023
**rs6656**	C	T	0.411	-0.044	0.009	-0.017	0.024
**rs6662**	G	T	0.197	-0.059	0.011	0.020	0.029
**rs6673880**	A	G	0.499	-0.062	0.009	-0.023	0.027
**rs66844263**	A	C	0.775	-0.042	0.010	0.003	0.028
**rs6694677**	C	T	0.841	-0.051	0.012	0.003	0.031
**rs6709782**	A	G	0.520	-0.036	0.009	-0.005	0.024
**rs6715366**	G	A	0.730	-0.054	0.010	-0.008	0.026
**rs67300956**	C	T	0.888	0.059	0.014	0.030	0.037
**rs67627854**	T	G	0.782	-0.056	0.010	-0.059	0.028
**rs67715018**	A	G	0.769	-0.048	0.010	0.011	0.028
**rs6790697**	C	T	0.966	0.089	0.020	0.051	0.072
**rs6798742**	A	G	0.694	-0.061	0.009	0.033	0.025
**rs6825268**	A	G	0.524	-0.047	0.009	-0.026	0.025
**rs6851389**	C	T	0.494	0.041	0.009	-0.010	0.023
**rs6943762**	T	C	0.881	0.105	0.013	0.029	0.036
**rs6974218**	A	C	0.628	0.055	0.009	-0.008	0.024
**rs6982908**	C	T	0.265	-0.044	0.010	0.007	0.027
**rs6984242**	G	A	0.409	0.055	0.009	-0.012	0.023
**rs7002992**	T	C	0.631	0.045	0.009	-0.041	0.024
**rs7022345**	A	G	0.175	-0.055	0.011	-0.030	0.030
**rs708228**	C	T	0.680	-0.053	0.009	0.031	0.024
**rs7096250**	C	A	0.219	-0.046	0.010	-0.004	0.027
**rs710616**	T	A	0.488	0.046	0.009	-0.009	0.023
**rs7112616**	T	C	0.515	0.052	0.009	-0.021	0.023
**rs7116022**	T	C	0.351	-0.044	0.009	0.009	0.024
**rs7119089**	G	A	0.440	-0.038	0.009	-0.015	0.023
**rs713692**	G	A	0.310	-0.057	0.010	0.005	0.026
**rs7157278**	G	C	0.630	0.046	0.009	-0.017	0.024
**rs71573419**	C	A	0.989	0.233	0.055	-0.099	0.148
**rs7162607**	G	A	0.557	-0.040	0.009	-0.018	0.024
**rs7203082**	G	A	0.755	-0.044	0.010	0.028	0.027
**rs7251**	C	G	0.668	0.064	0.009	-0.014	0.026
**rs72694418**	A	G	0.903	-0.072	0.015	-0.093	0.039

**rs72723227**	G	A	0.684	0.048	0.009	-0.009	0.026
**rs72743955**	A	G	0.987	0.169	0.039	0.050	0.119
**rs72802868**	G	T	0.712	0.069	0.010	0.015	0.025
**rs728055**	T	A	0.647	0.067	0.009	0.004	0.024
**rs72854425**	C	A	0.968	-0.112	0.026	0.017	0.069
**rs72943392**	G	C	0.720	-0.053	0.010	-0.032	0.026
**rs72986630**	C	T	0.940	-0.112	0.018	-0.002	0.057
**rs72997862**	A	C	0.959	0.120	0.026	0.100	0.073
**rs73038104**	C	T	0.699	0.040	0.010	-0.004	0.026
**rs73090180**	G	A	0.797	-0.057	0.011	-0.070	0.029
**rs7312697**	T	C	0.367	-0.049	0.009	0.055	0.024
**rs73172807**	G	A	0.850	0.055	0.012	0.039	0.032
**rs73200009**	T	C	0.846	-0.052	0.013	0.022	0.034
**rs73229090**	C	A	0.880	0.103	0.014	-0.001	0.036
**rs73292401**	T	A	0.806	-0.068	0.011	-0.018	0.029
**rs7333701**	G	C	0.932	-0.077	0.017	0.088	0.049
**rs7342307**	C	T	0.874	0.067	0.014	-0.032	0.038
**rs74335775**	C	T	0.976	-0.118	0.028	-0.052	0.084
**rs74379599**	G	A	0.935	0.084	0.019	0.020	0.048
**rs74700062**	C	T	0.964	-0.094	0.022	0.007	0.062
**rs75062856**	A	C	0.943	0.081	0.019	-0.048	0.052
**rs75072290**	T	C	0.832	-0.052	0.012	-0.042	0.033
**rs75072551**	T	C	0.984	-0.126	0.031	-0.080	0.094
**rs7515363**	C	T	0.382	0.054	0.009	-0.059	0.024
**rs75221401**	A	G	0.976	-0.136	0.033	-0.035	0.086
**rs7544605**	T	C	0.610	0.044	0.009	-0.026	0.024
**rs755702**	A	G	0.634	-0.042	0.009	0.015	0.024
**rs75677869**	A	G	0.904	-0.059	0.014	0.015	0.039
**rs75690664**	T	C	0.943	-0.086	0.020	-0.007	0.059
**rs7575796**	A	G	0.932	0.096	0.017	-0.114	0.057
**rs75792959**	T	C	0.966	-0.119	0.027	0.000	0.085
**rs76126732**	A	T	0.942	0.086	0.018	-0.018	0.051
**rs7618137**	T	C	0.708	-0.041	0.010	0.001	0.026
**rs761840**	C	T	0.367	-0.039	0.009	-0.006	0.024
**rs76190391**	A	T	0.873	0.057	0.014	0.008	0.035
**rs76243117**	A	G	0.883	-0.058	0.014	-0.012	0.036
**rs7634476**	A	G	0.441	-0.058	0.009	-0.005	0.024
**rs76403337**	T	C	0.949	-0.079	0.019	0.066	0.053
**rs7641821**	T	C	0.368	-0.038	0.009	0.053	0.024
**rs7647398**	C	T	0.790	0.077	0.011	-0.012	0.028
**rs76503615**	G	A	0.945	-0.096	0.018	0.003	0.052
**rs7676578**	T	A	0.773	0.045	0.011	-0.042	0.027
**rs7679800**	T	C	0.716	-0.046	0.010	-0.018	0.025
**rs76831675**	T	C	0.980	-0.178	0.038	-0.009	0.106
**rs76838079**	C	T	0.850	-0.078	0.014	0.053	0.037

**rs7684235**	T	C	0.492	0.045	0.009	0.025	0.023
**rs76852571**	C	G	0.909	0.065	0.015	-0.039	0.040
**rs7699241**	C	T	0.906	-0.078	0.015	-0.006	0.041
**rs77030891**	C	G	0.921	-0.069	0.016	0.077	0.045
**rs77181200**	C	A	0.970	0.108	0.025	-0.101	0.071
**rs77206190**	C	T	0.973	-0.152	0.028	0.051	0.077
**rs7735021**	A	G	0.177	-0.055	0.011	0.038	0.030
**rs77379825**	G	C	0.856	-0.062	0.012	-0.045	0.033
**rs7739151**	G	T	0.830	-0.056	0.012	-0.002	0.031
**rs7742212**	G	A	0.430	-0.041	0.009	-0.017	0.023
**rs77442047**	G	A	0.952	-0.090	0.021	-0.033	0.054
**rs7768725**	T	G	0.862	-0.053	0.012	-0.001	0.033
**rs77745530**	A	G	0.978	-0.138	0.028	-0.136	0.087
**rs7779018**	A	G	0.600	0.042	0.009	-0.016	0.026
**rs778371**	A	G	0.719	-0.081	0.010	-0.010	0.026
**rs77947333**	A	T	0.957	-0.106	0.022	-0.005	0.062
**rs7798283**	T	G	0.864	0.074	0.013	-0.040	0.034
**rs7799331**	A	G	0.832	0.054	0.012	-0.030	0.030
**rs7825426**	A	C	0.343	-0.038	0.009	-0.038	0.024
**rs7830315**	T	C	0.493	-0.048	0.009	-0.021	0.023
**rs7847153**	C	G	0.685	-0.051	0.010	-0.003	0.029
**rs78485958**	C	G	0.974	-0.128	0.029	-0.041	0.077
**rs7850322**	A	G	0.847	-0.061	0.012	-0.029	0.032
**rs7856690**	A	T	0.641	-0.047	0.009	-0.006	0.024
**rs7873149**	T	C	0.664	0.039	0.009	0.015	0.024
**rs7902292**	T	C	0.205	0.057	0.011	-0.019	0.030
**rs79073127**	G	C	0.880	0.073	0.014	-0.058	0.036
**rs7915131**	C	T	0.419	0.047	0.009	-0.131	0.023
**rs79169291**	G	T	0.986	-0.192	0.037	0.146	0.116
**rs79210963**	T	C	0.901	-0.086	0.014	-0.007	0.038
**rs79217743**	G	T	0.860	-0.056	0.013	0.009	0.035
**rs793248**	A	G	0.241	-0.042	0.010	0.007	0.027
**rs79415286**	A	G	0.905	0.066	0.014	0.005	0.042
**rs79445414**	T	C	0.963	-0.123	0.022	-0.050	0.063
**rs79478621**	T	G	0.977	0.117	0.022	-0.174	0.082
**rs7952868**	A	G	0.247	-0.044	0.010	-0.042	0.027
**rs7953300**	G	T	0.391	-0.047	0.009	-0.009	0.024
**rs795587**	A	G	0.546	-0.041	0.009	-0.020	0.024
**rs79784950**	G	T	0.756	-0.046	0.010	0.029	0.026
**rs8007841**	T	C	0.336	0.039	0.009	-0.038	0.024
**rs8012618**	G	A	0.914	-0.067	0.015	-0.050	0.041
**rs8013990**	G	T	0.429	0.040	0.009	-0.048	0.023
**rs8026363**	G	A	0.778	0.054	0.011	0.052	0.030
**rs8055219**	G	A	0.765	-0.067	0.010	-0.007	0.027
**rs8070040**	T	C	0.369	0.041	0.009	0.006	0.025

**rs8112050**	C	T	0.302	0.048	0.009	0.008	0.025
**rs8119702**	A	T	0.905	-0.058	0.014	-0.044	0.042
**rs834201**	A	G	0.396	-0.042	0.009	-0.021	0.025
**rs862989**	A	G	0.785	-0.042	0.010	-0.018	0.028
**rs886076**	T	C	0.616	-0.037	0.009	0.024	0.025
**rs891645**	T	A	0.710	0.045	0.010	-0.004	0.025
**rs9304548**	C	A	0.248	0.057	0.010	0.017	0.028
**rs9318627**	A	C	0.593	0.061	0.009	-0.046	0.023
**rs9396665**	A	G	0.873	-0.065	0.013	0.003	0.036
**rs9403484**	C	T	0.777	-0.053	0.010	-0.014	0.027
**rs9410573**	T	C	0.598	0.040	0.009	-0.035	0.025
**rs9461916**	T	C	0.396	-0.053	0.009	-0.038	0.024
**rs9470670**	T	G	0.829	-0.062	0.011	0.008	0.030
**rs9484329**	A	G	0.883	0.068	0.013	-0.013	0.036
**rs9487653**	A	G	0.234	0.056	0.011	0.041	0.027
**rs949458**	T	C	0.611	-0.036	0.009	-0.068	0.024
**rs950885**	T	C	0.895	0.062	0.014	0.000	0.037
**rs9554348**	T	A	0.844	0.061	0.013	-0.020	0.033
**rs9566131**	G	T	0.841	-0.050	0.012	-0.013	0.032
**rs9605070**	C	T	0.768	-0.048	0.010	-0.019	0.027
**rs9630925**	C	A	0.902	0.065	0.015	-0.002	0.038
**rs9636107**	A	G	0.516	-0.070	0.009	-0.026	0.023
**rs9673542**	C	T	0.940	-0.084	0.018	-0.074	0.047
**rs9674069**	A	G	0.216	0.053	0.010	0.017	0.028
**rs9687282**	T	G	0.654	-0.053	0.009	0.010	0.024
**rs979618**	C	T	0.594	0.045	0.009	0.010	0.023
**rs984032**	A	T	0.537	-0.039	0.009	-0.025	0.024
**rs9843908**	T	C	0.451	0.040	0.009	-0.024	0.023
**rs987484**	A	T	0.545	0.037	0.009	-0.089	0.023
**rs9876421**	C	T	0.664	-0.063	0.009	0.008	0.024
**rs9882339**	A	G	0.957	-0.088	0.021	0.045	0.059
**rs9902772**	T	C	0.920	0.070	0.017	0.042	0.044
**rs9971729**	A	C	0.438	-0.037	0.009	-0.011	0.023
**rs9975024**	A	G	0.541	-0.044	0.009	0.032	0.024
**rs9986069**	T	A	0.683	0.040	0.010	-0.012	0.027
**rs9989481**	T	C	0.439	-0.042	0.009	0.019	0.027
**rs999494**	C	T	0.800	0.056	0.011	-0.008	0.028

Table S59. Raw data for the MR analysis of the causal effect of schizophrenia on UC (P<5E-5)							
SNP	Effect allele	Other allele	Effect allele frequency	Beta.exposure	Se.exposure	Beta.outcome	Se.outcome
**rs1000237**	T	A	0.642	-0.073	0.009	-0.030	0.022
**rs10005436**	G	A	0.668	-0.040	0.009	0.059	0.023
**rs10035564**	A	G	0.660	-0.067	0.009	-0.020	0.023
**rs10069930**	T	A	0.489	0.045	0.009	0.007	0.022
**rs10086619**	A	G	0.842	-0.072	0.012	-0.042	0.029
**rs10100465**	G	A	0.782	0.043	0.011	-0.027	0.026
**rs10108980**	C	T	0.804	-0.063	0.011	-0.022	0.027
**rs10117**	G	A	0.598	0.055	0.009	0.023	0.022
**rs10133619**	T	G	0.611	0.044	0.009	-0.012	0.022
**rs10148671**	T	C	0.343	-0.047	0.009	-0.004	0.022
**rs10173857**	C	T	0.710	0.051	0.010	-0.028	0.024
**rs10238960**	C	T	0.291	-0.039	0.010	0.007	0.023
**rs10520163**	T	C	0.502	0.047	0.009	0.013	0.021
**rs10751832**	A	G	0.139	0.057	0.013	-0.013	0.031
**rs10760502**	A	G	0.345	-0.041	0.010	-0.036	0.024
**rs10821976**	G	A	0.755	0.046	0.011	0.024	0.028
**rs10832722**	C	T	0.696	0.049	0.010	0.083	0.024
**rs10861176**	G	A	0.276	-0.056	0.010	0.010	0.024
**rs10873538**	T	G	0.675	-0.067	0.009	0.007	0.023
**rs10876446**	G	C	0.687	-0.054	0.009	0.037	0.024
**rs10931083**	T	C	0.053	0.089	0.022	0.003	0.052
**rs10935477**	G	T	0.757	-0.043	0.010	0.056	0.025
**rs10999895**	C	T	0.613	-0.041	0.009	-0.012	0.022
**rs11027839**	A	C	0.498	-0.052	0.009	0.000	0.021
**rs11061973**	G	A	0.848	0.055	0.012	-0.037	0.029
**rs11064676**	C	G	0.791	0.045	0.011	-0.012	0.026
**rs1106478**	T	G	0.794	-0.056	0.011	-0.012	0.026
**rs11105285**	C	T	0.779	0.057	0.011	-0.006	0.027
**rs11129511**	G	A	0.660	0.039	0.009	0.030	0.022
**rs111320077**	C	T	0.897	-0.063	0.014	-0.001	0.035
**rs11136325**	G	A	0.417	0.054	0.009	-0.001	0.023
**rs11165690**	T	C	0.813	-0.054	0.011	-0.022	0.027
**rs11165867**	C	T	0.836	-0.074	0.012	-0.032	0.029
**rs11191580**	T	C	0.912	0.132	0.016	0.041	0.037
**rs1120004**	T	G	0.257	0.047	0.010	0.022	0.024
**rs11210892**	G	A	0.329	0.064	0.009	0.020	0.022
**rs11217602**	C	T	0.591	0.037	0.009	0.021	0.022
**rs112177478**	C	T	0.910	0.082	0.015	0.013	0.037
**rs11223774**	A	G	0.295	0.052	0.009	-0.003	0.023
**rs11251858**	C	T	0.636	-0.039	0.009	-0.002	0.022
**rs11252007**	C	T	0.738	-0.046	0.010	-0.010	0.026
**rs113264400**	T	C	0.954	-0.112	0.020	-0.080	0.054

**rs113892895**	A	T	0.943	0.093	0.019	0.040	0.046
**rs114702133**	A	G	0.955	-0.101	0.023	0.015	0.057
**rs11487432**	A	G	0.913	0.062	0.015	0.004	0.038
**rs11534045**	G	A	0.666	0.063	0.009	0.009	0.023
**rs115361459**	G	A	0.974	0.136	0.030	0.036	0.073
**rs11587347**	C	G	0.900	-0.104	0.015	-0.018	0.037
**rs11590169**	A	G	0.915	-0.067	0.016	-0.079	0.038
**rs11632125**	G	A	0.968	-0.118	0.026	-0.158	0.071
**rs116350522**	G	T	0.961	-0.102	0.023	0.109	0.062
**rs11647188**	A	G	0.602	0.048	0.009	-0.007	0.022
**rs11663962**	G	A	0.714	-0.043	0.010	0.009	0.023
**rs11664298**	G	A	0.803	-0.077	0.011	0.033	0.027
**rs11668896**	T	C	0.358	-0.048	0.010	0.018	0.027
**rs11680328**	T	A	0.125	0.064	0.013	0.022	0.033
**rs11686852**	G	A	0.556	0.046	0.009	0.012	0.022
**rs11687946**	G	T	0.580	0.043	0.009	-0.029	0.022
**rs11690184**	T	C	0.485	0.036	0.009	0.014	0.022
**rs11693094**	C	T	0.551	0.054	0.009	-0.006	0.022
**rs116966156**	C	T	0.983	-0.137	0.033	-0.120	0.084
**rs117170626**	G	A	0.974	-0.110	0.026	0.029	0.083
**rs117178087**	C	T	0.931	0.096	0.018	0.038	0.043
**rs11719324**	A	C	0.573	-0.038	0.009	-0.014	0.021
**rs11732321**	C	G	0.567	-0.040	0.009	0.005	0.022
**rs11740474**	A	T	0.592	-0.054	0.009	0.004	0.022
**rs11752044**	G	A	0.776	0.055	0.011	-0.028	0.026
**rs11780451**	T	G	0.086	-0.080	0.016	0.031	0.038
**rs118049780**	G	A	0.920	-0.074	0.018	-0.014	0.043
**rs11811826**	T	A	0.773	-0.045	0.010	-0.031	0.025
**rs118121724**	C	T	0.928	-0.073	0.017	-0.080	0.046
**rs11854073**	G	A	0.677	0.047	0.009	-0.024	0.023
**rs11878234**	A	C	0.874	0.058	0.014	-0.001	0.034
**rs11941714**	G	A	0.610	0.052	0.009	0.007	0.024
**rs11964401**	A	G	0.483	0.045	0.009	-0.011	0.021
**rs11972718**	C	G	0.700	-0.048	0.009	0.036	0.023
**rs1198588**	A	T	0.199	-0.103	0.011	-0.040	0.027
**rs12029721**	A	G	0.922	-0.081	0.018	0.014	0.047
**rs1203630**	A	G	0.265	0.043	0.010	0.020	0.024
**rs12040431**	C	T	0.332	0.040	0.009	-0.052	0.023
**rs12120481**	A	G	0.861	-0.058	0.013	-0.045	0.031
**rs12121218**	T	G	0.812	-0.049	0.011	-0.025	0.027
**rs12129573**	C	A	0.626	-0.078	0.009	0.019	0.022
**rs12138061**	C	A	0.791	-0.045	0.010	0.038	0.026
**rs12138231**	T	A	0.182	-0.067	0.012	-0.068	0.028
**rs12142659**	T	C	0.699	-0.045	0.010	-0.010	0.024
**rs12145726**	G	A	0.372	0.046	0.009	0.003	0.022

**rs12151767**	G	A	0.507	0.061	0.009	-0.019	0.021
**rs12153880**	G	A	0.808	0.052	0.011	-0.009	0.028
**rs1217091**	T	C	0.188	-0.059	0.011	-0.007	0.027
**rs12186968**	C	T	0.702	0.047	0.010	-0.016	0.024
**rs12190176**	A	G	0.641	-0.038	0.009	0.036	0.022
**rs12285419**	C	A	0.812	-0.085	0.011	0.004	0.028
**rs12293670**	A	G	0.662	0.070	0.009	0.003	0.022
**rs12303743**	G	C	0.903	-0.087	0.015	0.024	0.036
**rs12462062**	C	G	0.708	-0.040	0.010	-0.018	0.023
**rs12474906**	A	C	0.798	0.056	0.011	0.004	0.027
**rs12485775**	G	A	0.796	0.047	0.011	-0.003	0.026
**rs12489270**	T	C	0.620	-0.058	0.009	0.026	0.022
**rs12499194**	C	T	0.854	-0.056	0.013	0.012	0.032
**rs1253434**	T	C	0.677	0.043	0.009	0.043	0.023
**rs12577324**	T	C	0.791	0.046	0.011	0.019	0.027
**rs12600720**	C	G	0.685	-0.048	0.010	-0.016	0.026
**rs12615991**	A	G	0.854	-0.055	0.012	-0.015	0.030
**rs12624433**	G	A	0.739	-0.044	0.010	-0.056	0.024
**rs12652777**	T	C	0.477	0.049	0.009	0.018	0.021
**rs12671608**	T	C	0.877	-0.064	0.013	-0.016	0.036
**rs12712510**	T	C	0.465	0.057	0.009	0.015	0.021
**rs12771371**	G	A	0.691	0.052	0.009	-0.015	0.023
**rs12833624**	C	T	0.651	-0.050	0.009	0.073	0.022
**rs12877581**	G	C	0.734	-0.060	0.010	0.056	0.026
**rs12883788**	C	T	0.572	-0.061	0.009	-0.021	0.022
**rs1289003**	G	T	0.592	-0.048	0.009	0.007	0.021
**rs12943566**	A	G	0.341	-0.050	0.009	0.005	0.022
**rs13011472**	C	G	0.510	-0.070	0.009	-0.036	0.021
**rs13014947**	G	A	0.435	0.039	0.009	-0.012	0.022
**rs13016542**	T	C	0.869	0.088	0.013	0.046	0.032
**rs13025903**	T	A	0.779	-0.051	0.010	-0.026	0.026
**rs13030312**	A	T	0.871	-0.057	0.013	-0.106	0.031
**rs13074132**	T	C	0.645	0.047	0.009	0.011	0.023
**rs13090130**	G	A	0.627	0.041	0.009	0.020	0.022
**rs13107325**	C	T	0.919	-0.159	0.017	-0.031	0.040
**rs13145146**	A	G	0.948	-0.090	0.019	0.006	0.048
**rs13150491**	C	T	0.551	-0.037	0.009	-0.040	0.021
**rs13154281**	C	G	0.550	0.039	0.009	-0.048	0.022
**rs13177597**	C	G	0.871	-0.056	0.014	0.005	0.033
**rs1319017**	G	A	0.665	-0.046	0.009	0.021	0.023
**rs13195636**	A	C	0.911	0.211	0.016	0.019	0.037
**rs13233308**	C	T	0.521	0.049	0.009	0.004	0.021
**rs132582**	C	T	0.460	0.051	0.009	-0.010	0.021
**rs13273892**	C	A	0.673	-0.040	0.009	-0.032	0.023
**rs13320945**	C	T	0.608	-0.045	0.009	0.037	0.022

**rs13396659**	A	C	0.791	-0.046	0.011	-0.029	0.026
**rs1389718**	G	A	0.771	-0.044	0.010	-0.003	0.025
**rs1392414**	A	G	0.186	0.047	0.011	-0.003	0.027
**rs1398541**	G	A	0.685	-0.047	0.009	0.032	0.023
**rs139887650**	C	T	0.988	-0.242	0.055	-0.099	0.117
**rs140150373**	T	A	0.987	-0.158	0.036	0.166	0.111
**rs140769414**	T	G	0.884	0.061	0.014	-0.033	0.033
**rs1427633**	G	C	0.413	0.048	0.009	0.027	0.022
**rs143026942**	A	T	0.958	0.110	0.026	0.133	0.062
**rs1430894**	C	T	0.526	-0.053	0.009	-0.014	0.021
**rs143092720**	G	T	0.980	0.161	0.035	-0.105	0.086
**rs1444658**	G	A	0.534	-0.040	0.009	-0.005	0.022
**rs144673133**	A	G	0.984	-0.148	0.034	-0.074	0.094
**rs145071536**	T	C	0.805	-0.085	0.012	-0.009	0.031
**rs1451488**	A	G	0.446	-0.071	0.009	-0.031	0.021
**rs1452509**	A	G	0.762	-0.045	0.010	0.008	0.025
**rs1462358**	T	C	0.384	-0.043	0.009	0.039	0.022
**rs147169643**	C	T	0.976	-0.146	0.029	0.006	0.077
**rs1482419**	A	G	0.237	0.052	0.010	-0.007	0.025
**rs1482962**	C	G	0.928	-0.078	0.019	0.010	0.044
**rs149021237**	A	G	0.951	0.098	0.019	-0.055	0.049
**rs149165**	T	G	0.556	0.048	0.009	0.038	0.021
**rs1495099**	C	G	0.288	0.044	0.010	-0.082	0.024
**rs150751363**	C	G	0.973	-0.135	0.029	-0.071	0.077
**rs1510136**	A	G	0.751	0.053	0.010	0.021	0.024
**rs1526803**	C	G	0.723	0.050	0.010	0.018	0.025
**rs1529073**	T	C	0.899	-0.066	0.014	-0.013	0.036
**rs1531167**	A	T	0.755	-0.046	0.010	-0.013	0.026
**rs1534464**	G	T	0.808	-0.054	0.011	-0.036	0.027
**rs1540840**	G	C	0.550	0.056	0.009	0.002	0.025
**rs1593304**	A	G	0.203	-0.064	0.011	0.005	0.028
**rs1604060**	A	G	0.105	-0.077	0.014	0.017	0.034
**rs1615350**	C	T	0.263	0.074	0.010	0.001	0.024
**rs167924**	A	G	0.362	-0.050	0.009	-0.056	0.022
**rs16851048**	T	C	0.800	-0.074	0.011	-0.033	0.026
**rs16867571**	A	G	0.766	0.066	0.010	-0.005	0.026
**rs1693523**	C	T	0.246	-0.045	0.010	-0.023	0.025
**rs16964949**	G	A	0.931	0.087	0.017	-0.042	0.041
**rs17016552**	C	G	0.646	0.052	0.009	0.018	0.022
**rs17119214**	G	C	0.807	0.045	0.011	-0.004	0.027
**rs17128077**	C	T	0.859	-0.059	0.012	-0.034	0.030
**rs17194490**	G	T	0.838	-0.078	0.012	-0.007	0.029
**rs17242605**	A	G	0.516	-0.040	0.009	0.024	0.021
**rs17384726**	A	G	0.975	-0.126	0.031	-0.030	0.087
**rs17394269**	A	G	0.522	-0.037	0.009	-0.026	0.022

**rs17574718**	C	G	0.934	-0.071	0.017	-0.032	0.046
**rs17598179**	G	A	0.956	-0.098	0.023	0.115	0.058
**rs176648**	T	G	0.477	0.042	0.009	0.026	0.021
**rs17671845**	T	C	0.960	-0.121	0.024	-0.133	0.055
**rs17731**	G	A	0.627	-0.052	0.009	-0.008	0.022
**rs17731604**	C	A	0.524	-0.043	0.009	0.006	0.021
**rs17769886**	T	G	0.861	0.052	0.013	0.049	0.031
**rs17864935**	T	C	0.564	-0.047	0.009	0.007	0.021
**rs1832899**	T	C	0.894	-0.060	0.014	-0.044	0.034
**rs1833193**	T	A	0.542	0.039	0.009	0.037	0.022
**rs1857472**	C	A	0.686	0.046	0.009	0.004	0.023
**rs1862906**	G	A	0.552	0.047	0.009	0.017	0.022
**rs187557**	C	T	0.156	0.067	0.012	0.048	0.030
**rs1881046**	G	T	0.656	0.051	0.009	0.022	0.022
**rs188987899**	A	T	0.983	-0.183	0.044	-0.213	0.101
**rs1892346**	T	A	0.436	-0.048	0.009	-0.023	0.022
**rs1901512**	T	C	0.311	0.058	0.009	0.018	0.023
**rs1914399**	C	G	0.472	0.049	0.009	-0.007	0.021
**rs1915019**	A	G	0.257	0.057	0.010	-0.008	0.024
**rs1953205**	T	A	0.515	-0.050	0.009	-0.023	0.022
**rs1953613**	A	G	0.374	0.045	0.009	0.006	0.022
**rs1983488**	C	T	0.868	0.060	0.013	0.021	0.032
**rs1994224**	A	G	0.789	-0.055	0.010	0.005	0.026
**rs2038255**	C	T	0.822	0.055	0.011	0.047	0.028
**rs2053079**	A	G	0.768	-0.060	0.010	-0.032	0.025
**rs207458**	C	T	0.198	-0.045	0.011	-0.033	0.027
**rs2078266**	A	G	0.188	0.070	0.013	-0.018	0.033
**rs2121236**	T	C	0.720	-0.043	0.010	-0.011	0.024
**rs2147472**	G	A	0.610	0.042	0.009	0.009	0.022
**rs2153960**	G	A	0.297	0.045	0.009	-0.002	0.023
**rs215412**	G	A	0.673	-0.058	0.009	0.018	0.023
**rs217336**	C	A	0.579	0.050	0.009	-0.020	0.021
**rs2217023**	C	G	0.152	-0.050	0.012	-0.022	0.030
**rs2224086**	C	A	0.296	-0.056	0.010	0.009	0.026
**rs2236270**	G	T	0.609	-0.037	0.009	-0.028	0.022
**rs2238057**	T	G	0.589	-0.084	0.009	-0.043	0.021
**rs2251854**	G	A	0.324	0.043	0.009	0.008	0.023
**rs2252074**	T	G	0.602	-0.069	0.009	-0.034	0.022
**rs2279311**	T	C	0.808	-0.051	0.011	0.027	0.027
**rs2287921**	T	C	0.493	-0.043	0.009	-0.011	0.021
**rs2297293**	C	G	0.317	0.046	0.009	0.032	0.023
**rs2298450**	C	T	0.634	0.039	0.009	0.025	0.022
**rs2314785**	T	C	0.815	0.056	0.011	0.026	0.027
**rs2325913**	C	T	0.443	-0.045	0.009	0.010	0.022
**rs2332700**	C	G	0.244	0.075	0.010	-0.007	0.025

**rs2333321**	A	G	0.202	0.071	0.011	0.007	0.026
**rs2347256**	A	G	0.526	-0.039	0.009	-0.034	0.021
**rs2381411**	T	C	0.600	-0.050	0.009	-0.022	0.022
**rs2387414**	G	C	0.396	-0.045	0.009	-0.004	0.023
**rs2401925**	A	G	0.370	0.036	0.009	-0.005	0.022
**rs2417277**	A	G	0.651	0.042	0.009	0.009	0.022
**rs2420036**	T	C	0.785	0.043	0.011	-0.002	0.027
**rs2455415**	C	T	0.576	-0.049	0.009	-0.004	0.022
**rs2456020**	C	T	0.764	0.082	0.010	0.010	0.025
**rs2470180**	G	A	0.315	-0.040	0.010	-0.010	0.024
**rs2514218**	C	T	0.648	0.070	0.009	0.016	0.022
**rs254778**	G	A	0.316	-0.040	0.009	-0.028	0.023
**rs2551445**	C	T	0.100	-0.071	0.014	-0.028	0.035
**rs2635663**	C	A	0.790	0.055	0.011	0.057	0.027
**rs2649999**	T	C	0.345	0.042	0.009	0.034	0.023
**rs2694031**	A	G	0.232	0.049	0.010	-0.002	0.025
**rs2710323**	T	C	0.514	0.078	0.009	0.039	0.021
**rs2815731**	C	A	0.658	0.060	0.009	-0.025	0.022
**rs2825395**	T	G	0.737	-0.043	0.010	-0.006	0.024
**rs28587597**	T	A	0.663	-0.047	0.009	0.034	0.022
**rs28696126**	A	T	0.140	0.070	0.013	-0.024	0.032
**rs2909457**	G	A	0.445	0.049	0.009	-0.024	0.021
**rs2910591**	T	C	0.065	-0.086	0.018	-0.056	0.043
**rs2938147**	A	G	0.578	-0.038	0.009	0.028	0.022
**rs2949814**	C	G	0.729	0.048	0.010	0.006	0.024
**rs2973043**	A	G	0.225	0.049	0.010	0.024	0.025
**rs2975923**	C	T	0.392	0.039	0.009	0.018	0.022
**rs2999392**	C	T	0.309	-0.052	0.009	0.007	0.023
**rs3018001**	T	C	0.525	-0.043	0.009	0.007	0.022
**rs3026133**	G	A	0.505	0.043	0.009	0.014	0.021
**rs306206**	A	G	0.731	0.053	0.010	0.019	0.024
**rs308697**	C	A	0.557	0.050	0.009	-0.009	0.021
**rs310653**	T	C	0.502	-0.040	0.009	-0.016	0.027
**rs322124**	C	G	0.795	-0.055	0.011	-0.035	0.027
**rs33996476**	A	G	0.889	-0.070	0.014	0.013	0.035
**rs34103014**	C	T	0.946	0.089	0.020	-0.043	0.047
**rs34111680**	T	C	0.842	-0.049	0.012	0.029	0.029
**rs34275957**	A	G	0.746	-0.041	0.010	0.020	0.025
**rs34665905**	T	C	0.487	-0.038	0.009	0.005	0.021
**rs34768008**	A	T	0.902	0.076	0.015	-0.025	0.037
**rs34777351**	C	T	0.550	0.040	0.009	-0.021	0.021
**rs34888090**	G	A	0.838	-0.058	0.012	-0.047	0.029
**rs352766**	A	G	0.874	-0.060	0.013	-0.051	0.032
**rs35351411**	A	C	0.454	-0.064	0.009	-0.013	0.021
**rs35517955**	G	C	0.976	-0.224	0.051	0.066	0.091

**rs35564002**	C	T	0.985	-0.184	0.037	0.006	0.094
**rs356183**	G	C	0.453	0.041	0.009	0.034	0.022
**rs35628191**	C	T	0.587	0.039	0.009	0.078	0.022
**rs35734242**	T	C	0.560	-0.051	0.009	-0.014	0.022
**rs35780891**	G	A	0.827	-0.055	0.011	-0.031	0.028
**rs35981078**	G	A	0.802	0.055	0.011	0.020	0.027
**rs3739118**	G	A	0.714	0.057	0.010	0.012	0.024
**rs3752827**	T	A	0.691	0.047	0.009	-0.011	0.024
**rs3770754**	C	G	0.641	0.053	0.009	0.064	0.022
**rs3775182**	G	T	0.905	-0.068	0.015	0.055	0.036
**rs3781158**	C	T	0.650	0.047	0.009	0.006	0.022
**rs3788331**	C	T	0.555	-0.040	0.009	-0.019	0.021
**rs3791710**	T	C	0.796	0.060	0.011	-0.045	0.026
**rs3795310**	C	T	0.526	0.051	0.009	-0.039	0.022
**rs3802924**	A	C	0.798	0.074	0.011	0.005	0.027
**rs3812831**	T	C	0.469	-0.044	0.009	0.013	0.022
**rs3814883**	C	T	0.521	0.067	0.009	-0.004	0.022
**rs3824451**	T	C	0.843	-0.066	0.012	-0.032	0.029
**rs3845811**	C	G	0.576	0.038	0.009	0.018	0.022
**rs4129585**	A	C	0.443	0.075	0.009	-0.012	0.021
**rs41297816**	A	G	0.755	-0.051	0.010	-0.044	0.026
**rs41313730**	T	G	0.840	0.055	0.012	0.063	0.029
**rs41314284**	C	T	0.905	-0.081	0.015	-0.009	0.037
**rs41475545**	T	C	0.927	-0.083	0.017	-0.032	0.042
**rs4237587**	T	G	0.234	-0.043	0.010	-0.008	0.025
**rs4275631**	T	C	0.414	-0.038	0.009	-0.002	0.022
**rs428602**	A	G	0.578	0.037	0.009	0.002	0.021
**rs4308**	A	G	0.387	-0.044	0.009	0.000	0.022
**rs4341665**	A	G	0.073	-0.090	0.018	-0.052	0.042
**rs4347627**	T	A	0.701	-0.040	0.009	-0.007	0.023
**rs4353292**	A	G	0.584	0.041	0.009	-0.018	0.022
**rs4378995**	G	A	0.524	0.041	0.009	-0.018	0.021
**rs4540690**	A	G	0.481	0.046	0.009	0.046	0.021
**rs4575535**	A	G	0.298	-0.056	0.010	-0.021	0.023
**rs4625037**	A	C	0.123	0.054	0.013	-0.035	0.033
**rs4627212**	A	G	0.711	-0.051	0.010	-0.016	0.023
**rs4632195**	C	T	0.483	-0.047	0.009	0.037	0.021
**rs4636654**	G	A	0.593	0.048	0.009	-0.015	0.022
**rs4653164**	C	T	0.329	-0.051	0.009	0.023	0.023
**rs4668193**	G	A	0.387	-0.038	0.009	0.022	0.022
**rs4700418**	C	G	0.504	-0.070	0.009	0.040	0.021
**rs4702**	G	A	0.440	0.084	0.009	-0.030	0.022
**rs4721928**	G	A	0.846	-0.059	0.012	-0.004	0.031
**rs4739612**	C	T	0.491	0.037	0.009	0.006	0.021
**rs4766428**	C	T	0.558	-0.075	0.009	-0.011	0.022

**rs4779050**	T	G	0.368	0.058	0.009	-0.010	0.022
**rs4797210**	C	T	0.984	-0.141	0.033	-0.070	0.088
**rs480211**	C	T	0.489	-0.045	0.009	-0.011	0.021
**rs4812325**	G	A	0.385	-0.072	0.009	-0.050	0.022
**rs4813190**	A	G	0.754	0.048	0.010	-0.004	0.024
**rs4837628**	T	C	0.569	0.036	0.009	0.021	0.021
**rs4904476**	T	G	0.737	0.045	0.010	0.003	0.025
**rs4921741**	A	G	0.742	-0.056	0.010	-0.013	0.024
**rs4925114**	A	G	0.366	0.044	0.009	-0.016	0.022
**rs4934962**	T	A	0.395	0.045	0.009	0.015	0.022
**rs4950076**	T	C	0.499	-0.044	0.009	0.025	0.021
**rs495839**	T	C	0.124	0.068	0.016	0.039	0.038
**rs4974598**	C	T	0.436	0.036	0.009	0.047	0.022
**rs498591**	A	T	0.850	-0.072	0.012	-0.032	0.030
**rs500102**	T	C	0.404	0.052	0.009	0.036	0.022
**rs505061**	C	A	0.500	-0.053	0.009	0.040	0.021
**rs508161**	A	C	0.651	-0.045	0.009	-0.032	0.024
**rs528598**	T	C	0.515	-0.044	0.009	-0.001	0.021
**rs55648125**	A	G	0.896	-0.065	0.014	-0.033	0.035
**rs55700176**	T	C	0.665	-0.043	0.009	0.010	0.023
**rs55741542**	T	C	0.986	-0.172	0.036	-0.074	0.095
**rs56057437**	G	C	0.841	-0.055	0.012	0.030	0.032
**rs56205728**	G	A	0.710	-0.063	0.010	-0.019	0.025
**rs56286294**	C	T	0.598	0.038	0.009	-0.023	0.022
**rs56335113**	A	G	0.313	0.065	0.009	0.007	0.023
**rs57070985**	A	G	0.650	0.050	0.009	0.010	0.023
**rs57433322**	C	G	0.882	0.083	0.014	-0.016	0.033
**rs5751191**	T	C	0.504	-0.066	0.009	-0.014	0.021
**rs58120505**	T	C	0.575	0.090	0.009	0.021	0.022
**rs58763139**	A	G	0.960	-0.091	0.022	0.022	0.055
**rs59682551**	T	G	0.762	0.054	0.011	0.159	0.026
**rs60135207**	G	T	0.582	0.050	0.009	0.023	0.021
**rs6037811**	G	A	0.343	-0.039	0.009	-0.002	0.022
**rs6125656**	G	A	0.808	-0.064	0.011	-0.027	0.027
**rs6132302**	T	C	0.753	-0.048	0.011	-0.037	0.026
**rs61820761**	G	C	0.936	0.086	0.019	0.028	0.044
**rs61857878**	A	T	0.768	0.060	0.010	0.027	0.026
**rs61869029**	G	A	0.819	0.058	0.012	-0.007	0.032
**rs61901216**	A	G	0.820	0.048	0.011	0.013	0.028
**rs61920311**	A	C	0.581	0.048	0.009	0.031	0.023
**rs61937595**	C	T	0.911	0.130	0.016	0.123	0.043
**rs61973698**	C	T	0.775	-0.054	0.010	-0.030	0.025
**rs62018952**	T	C	0.279	-0.058	0.010	-0.016	0.024
**rs62183855**	A	C	0.795	0.066	0.011	0.033	0.027
**rs62279133**	T	C	0.736	-0.040	0.010	-0.002	0.024

**rs62408211**	A	T	0.649	0.040	0.009	0.054	0.022
**rs62456353**	T	G	0.813	-0.048	0.011	-0.026	0.028
**rs634940**	G	T	0.747	-0.066	0.010	-0.019	0.024
**rs646234**	A	G	0.545	-0.041	0.009	-0.020	0.021
**rs6478778**	G	T	0.904	-0.058	0.014	-0.049	0.036
**rs6482437**	A	C	0.104	-0.099	0.014	-0.027	0.035
**rs6499508**	A	G	0.864	0.069	0.013	0.044	0.031
**rs6520064**	A	G	0.794	-0.058	0.011	-0.045	0.026
**rs6538539**	G	T	0.447	0.057	0.009	0.005	0.021
**rs6545708**	T	A	0.550	0.042	0.009	0.015	0.022
**rs6546857**	A	G	0.766	-0.060	0.010	-0.008	0.025
**rs6549963**	T	C	0.585	0.048	0.009	0.021	0.022
**rs6656**	C	T	0.416	-0.044	0.009	-0.007	0.022
**rs6662**	G	T	0.193	-0.059	0.011	-0.038	0.027
**rs6673880**	A	G	0.506	-0.062	0.009	0.005	0.024
**rs66844263**	A	C	0.770	-0.042	0.010	-0.021	0.025
**rs6694677**	C	T	0.844	-0.051	0.012	-0.012	0.029
**rs6709782**	A	G	0.521	-0.036	0.009	-0.010	0.021
**rs6715366**	G	A	0.727	-0.054	0.010	0.005	0.024
**rs67300956**	C	T	0.891	0.059	0.014	-0.003	0.034
**rs67627854**	T	G	0.786	-0.056	0.010	-0.011	0.026
**rs67715018**	A	G	0.769	-0.048	0.010	-0.042	0.026
**rs6790697**	C	T	0.964	0.089	0.020	-0.092	0.063
**rs6798742**	A	G	0.691	-0.061	0.009	-0.018	0.023
**rs6825268**	A	G	0.527	-0.047	0.009	-0.021	0.022
**rs6851389**	C	T	0.502	0.041	0.009	-0.026	0.021
**rs6943762**	T	C	0.874	0.105	0.013	-0.055	0.032
**rs6974218**	A	C	0.628	0.055	0.009	0.010	0.022
**rs6982908**	C	T	0.261	-0.044	0.010	0.010	0.024
**rs6984242**	G	A	0.406	0.055	0.009	0.011	0.022
**rs7002992**	T	C	0.635	0.045	0.009	0.027	0.022
**rs7022345**	A	G	0.176	-0.055	0.011	-0.043	0.028
**rs708228**	C	T	0.676	-0.053	0.009	0.032	0.023
**rs7096250**	C	A	0.224	-0.046	0.010	0.012	0.025
**rs710616**	T	A	0.496	0.046	0.009	0.022	0.021
**rs7112616**	T	C	0.509	0.052	0.009	0.028	0.021
**rs7116022**	T	C	0.350	-0.044	0.009	-0.002	0.022
**rs7119089**	G	A	0.439	-0.038	0.009	-0.015	0.021
**rs713692**	G	A	0.323	-0.057	0.010	0.007	0.024
**rs7157278**	G	C	0.629	0.046	0.009	0.024	0.022
**rs71573419**	C	A	0.989	0.233	0.055	-0.135	0.131
**rs7162607**	G	A	0.557	-0.040	0.009	-0.034	0.022
**rs7203082**	G	A	0.762	-0.044	0.010	0.063	0.025
**rs7251**	C	G	0.664	0.064	0.009	-0.033	0.024
**rs72694418**	A	G	0.904	-0.072	0.015	-0.016	0.037

**rs72723227**	G	A	0.685	0.048	0.009	-0.046	0.024
**rs72743955**	A	G	0.985	0.169	0.039	0.212	0.102
**rs72802868**	G	T	0.706	0.069	0.010	0.005	0.023
**rs728055**	T	A	0.653	0.067	0.009	0.003	0.023
**rs72854425**	C	A	0.966	-0.112	0.026	-0.112	0.060
**rs72943392**	G	C	0.716	-0.053	0.010	0.005	0.024
**rs72986630**	C	T	0.943	-0.112	0.018	0.095	0.056
**rs72997862**	A	C	0.958	0.120	0.026	-0.045	0.066
**rs73038104**	C	T	0.697	0.040	0.010	-0.031	0.024
**rs73090180**	G	A	0.804	-0.057	0.011	-0.020	0.028
**rs7312697**	T	C	0.373	-0.049	0.009	0.000	0.022
**rs73172807**	G	A	0.848	0.055	0.012	-0.009	0.030
**rs73200009**	T	C	0.851	-0.052	0.013	0.031	0.032
**rs73229090**	C	A	0.882	0.103	0.014	-0.024	0.034
**rs73292401**	T	A	0.805	-0.068	0.011	0.005	0.027
**rs7333701**	G	C	0.933	-0.077	0.017	-0.014	0.045
**rs7342307**	C	T	0.880	0.067	0.014	-0.039	0.035
**rs74335775**	C	T	0.975	-0.118	0.028	-0.023	0.072
**rs74379599**	G	A	0.937	0.084	0.019	0.058	0.045
**rs74700062**	C	T	0.961	-0.094	0.022	-0.009	0.054
**rs75062856**	A	C	0.944	0.081	0.019	-0.043	0.048
**rs75072290**	T	C	0.831	-0.052	0.012	-0.025	0.031
**rs75072551**	T	C	0.983	-0.126	0.031	-0.118	0.081
**rs7515363**	C	T	0.386	0.054	0.009	0.008	0.022
**rs75221401**	A	G	0.976	-0.136	0.033	-0.148	0.077
**rs7544605**	T	C	0.617	0.044	0.009	0.019	0.022
**rs755702**	A	G	0.632	-0.042	0.009	-0.041	0.022
**rs75677869**	A	G	0.901	-0.059	0.014	-0.065	0.035
**rs75690664**	T	C	0.942	-0.086	0.020	-0.055	0.050
**rs7575796**	A	G	0.929	0.096	0.017	-0.050	0.052
**rs75792959**	T	C	0.968	-0.119	0.027	-0.061	0.078
**rs76126732**	A	T	0.941	0.086	0.018	-0.004	0.046
**rs7618137**	T	C	0.710	-0.041	0.010	-0.034	0.024
**rs761840**	C	T	0.368	-0.039	0.009	0.034	0.022
**rs76190391**	A	T	0.880	0.057	0.014	0.045	0.033
**rs76243117**	A	G	0.887	-0.058	0.014	0.043	0.034
**rs7634476**	A	G	0.449	-0.058	0.009	-0.012	0.022
**rs76403337**	T	C	0.947	-0.079	0.019	0.066	0.048
**rs7641821**	T	C	0.364	-0.038	0.009	-0.040	0.022
**rs7647398**	C	T	0.797	0.077	0.011	0.046	0.027
**rs76503615**	G	A	0.945	-0.096	0.018	-0.029	0.047
**rs7676578**	T	A	0.774	0.045	0.011	-0.020	0.025
**rs7679800**	T	C	0.718	-0.046	0.010	-0.007	0.024
**rs76831675**	T	C	0.980	-0.178	0.038	-0.076	0.093
**rs76838079**	C	T	0.851	-0.078	0.014	-0.034	0.035

**rs7684235**	T	C	0.493	0.045	0.009	0.027	0.021
**rs76852571**	C	G	0.910	0.065	0.015	0.040	0.038
**rs7699241**	C	T	0.909	-0.078	0.015	0.049	0.038
**rs77030891**	C	G	0.920	-0.069	0.016	0.009	0.041
**rs77181200**	C	A	0.968	0.108	0.025	0.069	0.066
**rs77206190**	C	T	0.975	-0.152	0.028	-0.020	0.071
**rs7735021**	A	G	0.177	-0.055	0.011	0.052	0.028
**rs77379825**	G	C	0.856	-0.062	0.012	0.006	0.030
**rs7739151**	G	T	0.833	-0.056	0.012	-0.002	0.028
**rs7742212**	G	A	0.433	-0.041	0.009	0.010	0.022
**rs77442047**	G	A	0.954	-0.090	0.021	0.032	0.051
**rs7768725**	T	G	0.865	-0.053	0.012	0.026	0.031
**rs77745530**	A	G	0.978	-0.138	0.028	-0.081	0.081
**rs7779018**	A	G	0.595	0.042	0.009	-0.015	0.024
**rs778371**	A	G	0.719	-0.081	0.010	-0.003	0.024
**rs77947333**	A	T	0.957	-0.106	0.022	0.023	0.057
**rs7798283**	T	G	0.867	0.074	0.013	0.019	0.032
**rs7799331**	A	G	0.833	0.054	0.012	-0.003	0.028
**rs7825426**	A	C	0.339	-0.038	0.009	-0.063	0.022
**rs7830315**	T	C	0.494	-0.048	0.009	-0.046	0.021
**rs7847153**	C	G	0.682	-0.051	0.010	-0.038	0.027
**rs78485958**	C	G	0.972	-0.128	0.029	-0.023	0.068
**rs7850322**	A	G	0.845	-0.061	0.012	-0.022	0.029
**rs7856690**	A	T	0.645	-0.047	0.009	-0.013	0.022
**rs7873149**	T	C	0.664	0.039	0.009	0.025	0.023
**rs7902292**	T	C	0.200	0.057	0.011	0.000	0.028
**rs79073127**	G	C	0.884	0.073	0.014	-0.066	0.034
**rs7915131**	C	T	0.429	0.047	0.009	-0.017	0.021
**rs79169291**	G	T	0.986	-0.192	0.037	-0.070	0.098
**rs79210963**	T	C	0.896	-0.086	0.014	-0.026	0.035
**rs79217743**	G	T	0.862	-0.056	0.013	0.018	0.033
**rs793248**	A	G	0.242	-0.042	0.010	0.021	0.025
**rs79415286**	A	G	0.904	0.066	0.014	-0.023	0.039
**rs79445414**	T	C	0.960	-0.123	0.022	-0.061	0.055
**rs79478621**	T	G	0.974	0.117	0.022	0.002	0.074
**rs7952868**	A	G	0.243	-0.044	0.010	-0.031	0.025
**rs7953300**	G	T	0.385	-0.047	0.009	-0.029	0.022
**rs795587**	A	G	0.543	-0.041	0.009	0.024	0.022
**rs79784950**	G	T	0.752	-0.046	0.010	-0.037	0.024
**rs8007841**	T	C	0.338	0.039	0.009	0.008	0.022
**rs8012618**	G	A	0.916	-0.067	0.015	0.032	0.039
**rs8013990**	G	T	0.420	0.040	0.009	-0.007	0.022
**rs8026363**	G	A	0.788	0.054	0.011	0.007	0.028
**rs8055219**	G	A	0.765	-0.067	0.010	-0.012	0.025
**rs8070040**	T	C	0.363	0.041	0.009	-0.016	0.023

**rs8112050**	C	T	0.297	0.048	0.009	0.016	0.023
**rs8119702**	A	T	0.902	-0.058	0.014	-0.020	0.037
**rs834201**	A	G	0.396	-0.042	0.009	-0.020	0.023
**rs862989**	A	G	0.781	-0.042	0.010	0.012	0.026
**rs886076**	T	C	0.609	-0.037	0.009	-0.025	0.022
**rs891645**	T	A	0.704	0.045	0.010	-0.008	0.024
**rs9304548**	C	A	0.247	0.057	0.010	-0.006	0.026
**rs9318627**	A	C	0.601	0.061	0.009	0.005	0.022
**rs9396665**	A	G	0.877	-0.065	0.013	-0.014	0.034
**rs9403484**	C	T	0.775	-0.053	0.010	-0.014	0.025
**rs9410573**	T	C	0.595	0.040	0.009	0.017	0.022
**rs9461916**	T	C	0.400	-0.053	0.009	-0.003	0.022
**rs9470670**	T	G	0.831	-0.062	0.011	0.053	0.029
**rs9484329**	A	G	0.878	0.068	0.013	-0.015	0.032
**rs9487653**	A	G	0.223	0.056	0.011	0.023	0.025
**rs949458**	T	C	0.606	-0.036	0.009	-0.010	0.022
**rs950885**	T	C	0.892	0.062	0.014	-0.048	0.034
**rs9554348**	T	A	0.851	0.061	0.013	0.070	0.031
**rs9566131**	G	T	0.839	-0.050	0.012	0.000	0.029
**rs9605070**	C	T	0.769	-0.048	0.010	-0.022	0.026
**rs9630925**	C	A	0.905	0.065	0.015	0.021	0.036
**rs9636107**	A	G	0.521	-0.070	0.009	-0.006	0.021
**rs9673542**	C	T	0.940	-0.084	0.018	-0.075	0.044
**rs9674069**	A	G	0.217	0.053	0.010	0.005	0.026
**rs9687282**	T	G	0.659	-0.053	0.009	0.009	0.023
**rs979618**	C	T	0.590	0.045	0.009	0.005	0.022
**rs984032**	A	T	0.538	-0.039	0.009	-0.034	0.022
**rs9843908**	T	C	0.448	0.040	0.009	0.002	0.021
**rs987484**	A	T	0.548	0.037	0.009	-0.005	0.021
**rs9876421**	C	T	0.658	-0.063	0.009	-0.061	0.022
**rs9882339**	A	G	0.956	-0.088	0.021	0.004	0.053
**rs9902772**	T	C	0.920	0.070	0.017	0.059	0.041
**rs9971729**	A	C	0.436	-0.037	0.009	-0.039	0.022
**rs9975024**	A	G	0.530	-0.044	0.009	0.018	0.022
**rs9986069**	T	A	0.685	0.040	0.010	0.010	0.024
**rs9989481**	T	C	0.434	-0.042	0.009	0.010	0.023
**rs999494**	C	T	0.802	0.056	0.011	-0.005	0.027
