# Supplementary material for: Causal atlas between inflammatory bowel disease and mental disorders: a bi-directional 2-sample Mendelian randomization study
Source: Front Immunol. 2023 Oct 13;14:1267834. doi: 10.3389/fimmu.2023.1267834 (PMC10611497; doi:10.3389/fimmu.2023.1267834)
Supplement: Supplementary file 2 [file Presentation_1.pdf]

STROBE-MR checklist of recommended items to address in reports of Mendelian randomization studies[1 2]

Item No.	Section		Checklist item	Page No.	Relevant text from manuscript
1	**TITLE and ABSTRACT**		Indicate Mendelian randomization (MR) as the study's design in the title and/or the abstract if that is a main purpose of the study	1	Causal Atlas Between Inflammatory Bowel Disease and Mental Disorders: A Bi-directional 2-Sample Mendelian Randomization Study
	**INTRODUCTION**				
2	**Background**		Explain the scientific background and rationale for the reported study. What is the exposure? Is a potential causal relationship between exposure and outcome plausible? Justify why MR is a helpful method to address the study question	2	However, the current studies are mostly limited to the observational correlation, and the cause and effect between IBD and mental disorders are still unknown.<br>The Mendelian randomization (MR) method is a common approach in causality research, which is similar to randomized controlled trials and is a natural randomized trial.
3	**Objectives**		State specific objectives clearly, including pre-specified causal hypotheses (if any). State that MR is a method that, under specific assumptions, intends to estimate causal effects	2	The application of the MR method has three basic prerequisites: (a) instrumental variables, namely gene polymorphism, are closely related to exposure factors; (b) instrumental variables should be independent of any exposure confounders; (c) the association between instrumental variables and research outcomes only exists through exposure factors.
	**METHODS**				
4	**Study design and data sources**		Present key elements of the study design early in the article. Consider including a table listing sources of data for all phases of the study. For each data source contributing to the analysis, describe the following:	2-4	Figure 1; Study design and Data sources
		a)	Setting: Describe the study design and the underlying population, if possible. Describe the setting, locations, and relevant dates, including periods of recruitment, exposure, follow-up, and data collection, when available.	2-4	Figure 1; Study design and Data sources
		b)	Participants: Give the eligibility criteria, and the sources and methods of selection of participants. Report the sample size, and whether any power or sample size calculations were carried out prior to the main analysis	3-4	Figure 1B; Table 1; Study design and Data sources
		c)	Describe measurement, quality control and selection of genetic variants	3-4	Figure 1B; Table 1; Instruments Selection

		d)	For each exposure, outcome, and other relevant variables, describe methods of assessment and diagnostic criteria for diseases	3-4	Figure 1B; Table 1; Cited articles
		e)	Provide details of ethics committee approval and participant informed consent, if relevant	2	Ethics considerations<br>This current study is based on the summary results from the published studies. Ethical approval for each of the included studies can be found in the original publication. Because our study uses summary-level data and ethical approval and informed consent were obtained in all original studies, ethical approval is exempted in our hospital.
5	**Assumptions**		Explicitly state the three core IV assumptions for the main analysis (relevance, independence and exclusion restriction) as well assumptions for any additional or sensitivity analysis	3-5	Figure 1B; Table 1; Instruments Selection and Statistical analyses
6	**Statistical methods: main analysis**		Describe statistical methods and statistics used	3-5	Figure 1B; Statistical Analyses
		a)	Describe how quantitative variables were handled in the analyses (i.e., scale, units, model)	3-5	Figure 1B; Statistical Analyses
		b)	Describe how genetic variants were handled in the analyses and, if applicable, how their weights were selected	3-5	Figure 1B; Statistical Analyses
		c)	Describe the MR estimator (e.g. two-stage least squares, Wald ratio) and related statistics. Detail the included covariates and, in case of two-sample MR, whether the same covariate set was used for adjustment in the two samples	3-5	Figure 1B; Statistical Analyses
		d)	Explain how missing data were addressed	3-5	Figure 1B; Statistical Analyses
		e)	If applicable, indicate how multiple testing was addressed	3-5	In order to initially control the risk of potential multiple tests in our study, the $P < 0.01$ was a significant correlation, and P values between 0.01 and 0.05 were considered suggestive correlation.
7	**Assessment of assumptions**		Describe any methods or prior knowledge used to assess the assumptions or justify their validity	2-5	Methods
8	**Sensitivity analyses and additional analyses**		Describe any sensitivity analyses or additional analyses performed (e.g. comparison of effect estimates from different approaches, independent replication, bias analytic techniques, validation of instruments, simulations)	2-5	Statistical Analyses; Sensitivity Analysis

9	**Software and pre-registration**			5	We used R software (Version 4.1.0; https://www.r-project.org/) to perform all statistical analyses and graph presentations. The R-based "TwoSampleMR" package was applied to conduct our MR analysis.
		a)	Name statistical software and package(s), including version and settings used	5	R software (Version 4.1.0）
		b)	State whether the study protocol and details were pre-registered (as well as when and where)		Not applicable
	**RESULTS**				
10	**Descriptive data**				
		a)	Report the numbers of individuals at each stage of included studies and reasons for exclusion. Consider use of a flow diagram	3	Figure 1
		b)	Report summary statistics for phenotypic exposure(s), outcome(s), and other relevant variables (e.g. means, SDs, proportions)	4-5	Table 1; Assessment of genetic instruments, Supplementary Tables
		c)	If the data sources include meta-analyses of previous studies, provide the assessments of heterogeneity across these studies	4-5	Table 1; Assessment of genetic instruments, Supplementary Tables
		d)	For two-sample MR:<br>i. Provide justification of the similarity of the genetic variant-exposure associations between the exposure and outcome samples<br>ii. Provide information on the number of individuals who overlap between the exposure and outcome studies	4-5	Table 1, Supplementary Tables
11	**Main results**				
		a)	Report the associations between genetic variant and exposure, and between genetic variant and outcome, preferably on an interpretable scale	4-7	Figure 2-3; Table 1, Supplementary Tables
		b)	Report MR estimates of the relationship between exposure and outcome, and the measures of uncertainty from the MR analysis, on an interpretable scale, such as odds ratio or relative risk per SD difference	4-7	Figure 2-3; Results, Supplementary Tables
		c)	If relevant, consider translating estimates of relative risk into absolute risk for a meaningful time period	4-7	Figure 2-3; Results, Supplementary Tables
		d)	Consider plots to visualize results (e.g. forest plot, scatterplot of associations between genetic variants and outcome versus between genetic variants and exposure)	4-7	Figure 2-3
12	**Assessment of assumptions**				

		a)	Report the assessment of the validity of the assumptions	4-7	Figure 2-3; Table 1, Supplementary Tables
		b)	Report any additional statistics (e.g., assessments of heterogeneity across genetic variants, such as I^2, Q statistic or E-value)	4-7	Figure 2-3; Table 1, Supplementary Tables
13	**Sensitivity analyses and additional analyses**				
		a)	Report any sensitivity analyses to assess the robustness of the main results to violations of the assumptions	5	Table S3-S5; Sensitivity Analysis
		b)	Report results from other sensitivity analyses or additional analyses	5	Table S3-S5; Sensitivity Analysis
		c)	Report any assessment of direction of causal relationship (e.g., bidirectional MR)	5	Table S3-S5; Results
		d)	When relevant, report and compare with estimates from non-MR analyses	5	Table S3-S5.
		e)	Consider additional plots to visualize results (e.g., leave-one-out analyses)	5	Sensitivity Analysis
	**DISCUSSION**				
14	**Key results**		Summarize key results with reference to study objectives	5-6	The first paragraph of the discussion
15	**Limitations**		Discuss limitations of the study, taking into account the validity of the IV assumptions, other sources of potential bias, and imprecision. Discuss both direction and magnitude of any potential bias and any efforts to address them	8	The main limitations of our study have been stated below.…
16	**Interpretation**				
		a)	Meaning: Give a cautious overall interpretation of results in the context of their limitations and in comparison with other studies	6-8	Discussion
		b)	Mechanism: Discuss underlying biological mechanisms that could drive a potential causal relationship between the investigated exposure and the outcome, and whether the gene-environment equivalence assumption is reasonable. Use causal language carefully, clarifying that IV estimates may provide causal effects only under certain assumptions	6-8	Discussion
		c)	Clinical relevance: Discuss whether the results have clinical or public policy relevance, and to what extent they inform effect sizes of possible interventions	6-8	Discussion
17	**Generalizability**		Discuss the generalizability of the study results (a) to other populations, (b) across other exposure periods/timings, and (c) across other levels of exposure	6-8	Discussion
	**OTHER INFORMATION**				

18	**Funding**	Describe sources of funding and the role of funders in the present study and, if applicable, sources of funding for the databases and original study or studies on which the present study is based	9	Funding
19	**Data and data sharing**	Provide the data used to perform all analyses or report where and how the data can be accessed, and reference these sources in the article. Provide the statistical code needed to reproduce the results in the article, or report whether the code is publicly accessible and if so, where	8	Data accessibility statement
20	**Conflicts of Interest**	All authors should declare all potential conflicts of interest	9	Conflict of interest<br>The authors declare that the research was conducted in the absence of any commercial or financial relationships that could be construed as a potential conflict of interest.

This checklist is copyrighted by the Equator Network under the Creative Commons Attribution 3.0 Unported (CC BY 3.0) license.

1. Skrivankova VW, Richmond RC, Woolf BAR, Yarmolinsky J, Davies NM, Swanson SA, et al. Strengthening the Reporting of Observational Studies in Epidemiology using Mendelian Randomization (STROBE-MR) Statement. JAMA. 2021;under review.

2. Skrivankova VW, Richmond RC, Woolf BAR, Davies NM, Swanson SA, VanderWeele TJ, et al. Strengthening the Reporting of Observational Studies in Epidemiology using Mendelian Randomisation (STROBE-MR): Explanation and Elaboration. BMJ. 2021;375:n2233.
